# C1′-Substituted Penta- and Heptamethine Cyanines: Synthetic Scope and Properties

**DOI:** 10.1021/acs.joc.6c00573

**Published:** 2026-07-06

**Authors:** Ottavio Bedocchi, Jana Okoročenkova, Petr Klán

**Affiliations:** † Department of Chemistry, Faculty of Science, 37748 Masaryk University, Kamenice 5, 625 00 Brno, Czech Republic; ‡ RECETOX, Faculty of Science, Masaryk University, Kamenice 5, 625 00 Brno, Czech Republic

## Abstract

Cyanine dyes are of particular importance in contemporary bioimaging and fluorescence-based applications. The structure of these dyes features a polymethine linker, which exerts a substantial influence on their spectroscopic properties. Despite the extensive use of cyanines, synthetic methods for the functionalization of their polymethine chain remain limited. This study presents a comprehensive methodology for the synthesis of penta- and heptamethine cyanines bearing diverse substituents in the C1′ (chain) position. It is a follow-up to our recent report ( J. Org. Chem. 2025, 90, 17797 41359637 10.1021/acs.joc.5c02283PMC12723678), which explained why cyanines with bulky and electron-withdrawing groups in this position exhibit significantly altered absorption properties. The synthetic protocol demonstrates a high functional-group tolerance, moderate to excellent yields, and flexibility toward the reaction partners, thus establishing itself as a valuable tool for the preparation of new cyanine dyes. The steady-state absorption and emission spectra, singlet-oxygen production and photooxygenation efficiencies, and photostabilities of the synthesized derivatives are presented herein. Furthermore, our results indicate that the selected cyanines exhibit a high degree of sensitivity to pH variations and to nucleophilic addition, thus illustrating the potential of the novel cyanine derivatives to serve as sensors and probes in a variety of biological applications.

## Graphical abstract

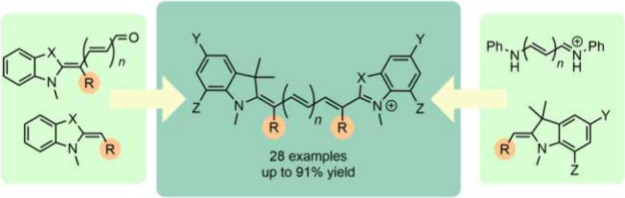

## Introduction

Cyanines (Cy) are a diverse group of organic dyes that contain a polymethine chain, which connects two heteroatom-containing end groups.^1^ The length of this chain determines its unique photophysical properties and chemical reactivity.^2–4^ Pentamethine (Cy5) and, in particular, heptamethine (Cy7) cyanines have attracted significant attention in biology due to their absorption and emission close to or within the phototherapeutic window.^5^ Cyanines have found widespread use in bioimaging,^6–8^ sensing,^9,10^ and drug delivery.^11,12^ The medical significance of Cy7 derivatives is evident in the case of indocyanine green (ICG), a substance approved by the Food and Drug Administration.^13^

The demand for new fluorescent dyes with specific optical properties remains strong. In this context, the functionalization of polymethine chains of cyanines plays an important role.^10,14,15^ Chain substituents can modulate the photophysical properties of the dye, such as emission efficiency,^16^ solubility,^17^ photostability,^18^ and singlet oxygen sensitization.^14,19^ Despite the importance of chain functionalization in tuning the properties of Cy dyes, only a few methodologies are available for their synthesis.

The scope of *meso*-position (central-carbon) functionalization of the polymethine chain is well-established for Cy5s^20,21^ and Cy7s.^22,23^ Our group has reported a general method for the functionalization of the C3′, C4′, and C5′ positions of Cy7s by the direct transformation of Zincke salts to cyanine dyes under mild conditions.^15^ An alternative procedure has recently been developed using substituted furfural derivatives.^24^ The synthesis of several 1′-substituted trimethine cyanines (Cy3s) has been reported,^25,26^ while examples of analogous 1′-substituted Cy5s are very limited.^27,28^ Functionalizing the C1′ positions of Cy5s and especially Cy7s remained elusive until our recent report of the preparation of C1′-substituted derivatives bearing alkyl and cyano groups. In this work, we investigated symmetry breaking in cyanines with substituents in the C1′ position and its impact on bond length alternation and out-of-plane rotation of the heterocyclic end group.^29^ We concluded that the significant steric and to some extent electronic effects of these substituents can induce electronic and geometric distortions of the entire molecule. These distortions were manifested by significant hypsochromic shifts in the absorption spectra and an increase in the intensity of the S_0_→S_2_ absorption band relative to that of S_0_→S_1_.

Considering the significant influence of C1′ substituents on the properties of cyanine dyes, the present study has sought to expand the scope of this novel synthetic methodology, and a general approach for their preparation has been formulated. Specifically, the synthesis of different heterocyclic (indolenine and benzothiazole) end groups, the introduction of a diverse group at the C1′ position, and the preparation of 1′,5′- and 1′,7′-disubstituted Cy5 and Cy7 derivatives were the focus of our work (Figure 1 lists all Cy5s (**Cy5–1**–**Cy5–12**) and Cy7s (**Cy7–1**–**Cy7–18**) derivatives that appear in the paper. Moreover, we demonstrate that some cyanine derivatives exhibit a specific and tunable pH response and sensitivity to nucleophilic attack in their absorption and emission spectra, which could further lead to specific applications in medicine and biology.

**Figure 1:**
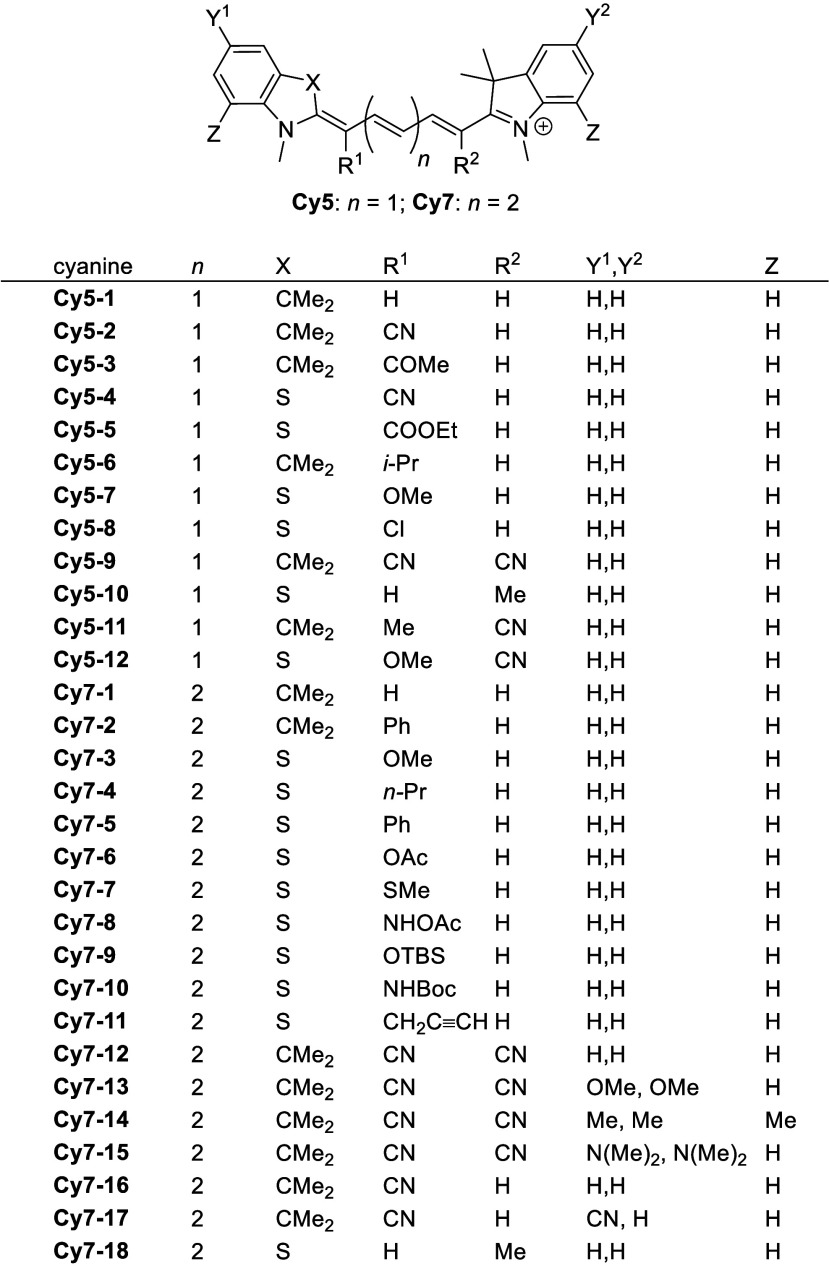
Cyanine derivatives prepared and discussed in this work.

## Results and Discussion

### Synthesis

In our previous work, several 1′-substituted Cy5 and Cy7 derivatives (**Cy5–2**, **Cy5–4**, **Cy5–6**, **Cy5–9**, **Cy7–4**, **Cy7–12**, and **Cy7–16**; Schemes 1–3) were synthesized by condensation of 2-substituted Fischer’s bases **1** or **2**, prepared in advance by deprotonation of quaternary nitrogen salts **3** or **4**, and chloropolyene derivatives **5** and **6** generated in situ from the corresponding enaminaldehydes **7** and **8**, respectively.^29^

**Scheme 1:**
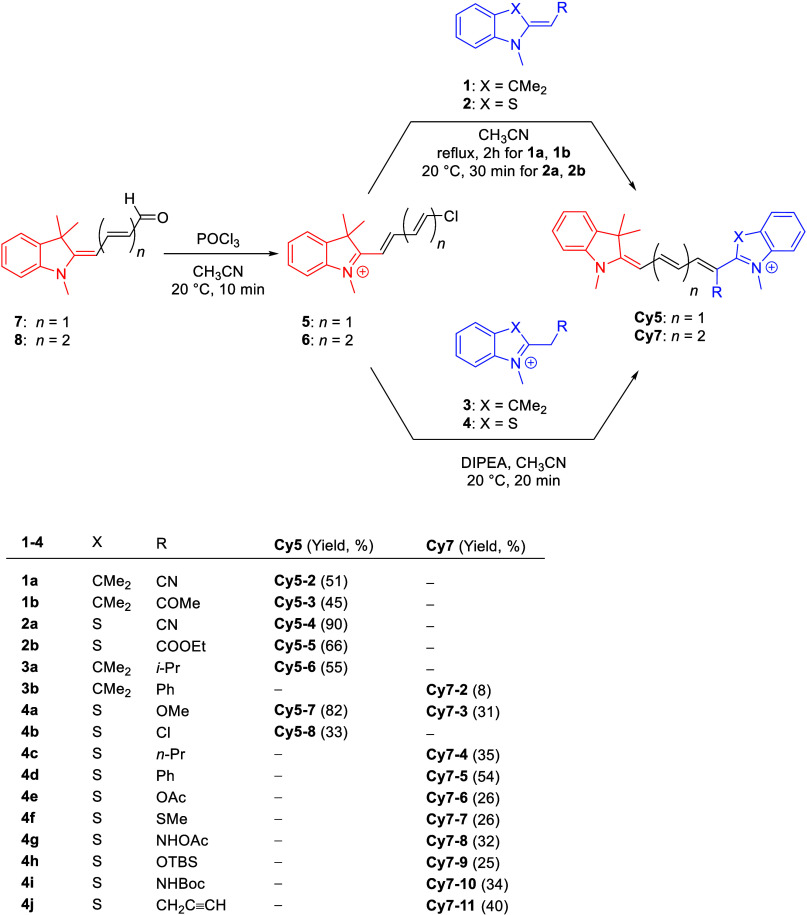
Synthesis of 1′-Substituted Cy5s and Cy7s

The direct employment of **1** or **2** bypassed the utilization of a base that can cause partial degradation of **5** during the final step. 1′,5′-Dicyano (**Cy5–9**) and 1′,7′-dicyano (**Cy7–12**) derivatives were obtained in good yields by the condensation of 2-(1,3,3-trimethylindolin-2-ylidene)­acetonitrile **1a** ^18^ with commercially available dianilide hydrochloride derivatives of malonaldehyde (**9** *n* = 1) or glutaconaldehyde (**10** *n* = 1), respectively, in acetic anhydride at elevated temperatures (Scheme 2).

**Scheme 2:**
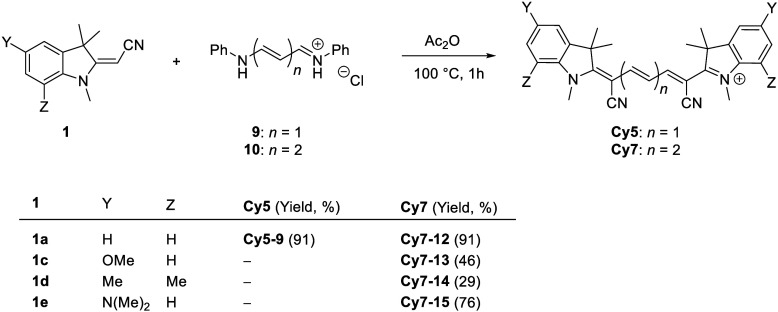
Synthesis of 1′,5′- and 1′,7′-Disubstituted Cy5s and Cy7s

In the initial phase of our present research, we focused on the development of substituted 2-methylene-1-indolenine and 2-methylene-benzothiazole precursors (Fischer’s bases) to demonstrate the robustness of the synthetic method for a wide range of substituents. It has been demonstrated that some Fischer’s bases **1** or **2**, which bear electron-withdrawing groups at the 2-methylene position, were reported to be stable in solutions.^30^ 2-Indolenine derivatives **1a** ^18^ or **1b** ^31^ (Scheme 1) were obtained through the cyanation or acetylation, respectively, of commercially available 1,3,3-trimethyl-2-methyleneindoline in good yields. The benzothiazole analog **2a** ^26^ was prepared by cyanation of 3-methyl-2-(methylthio)­benzo­[*d*]­thiazol-3-ium, while the ethyl ester derivative **2b** ^32^ was obtained by a three-step sequence starting with the condensation of 2-aminothiophenol and ethyl cyanoacetate.

Finally, the prepared Fischer’s bases reacted with chloroallylidene **5** to give the corresponding Cy5s (**Cy5–2**, **Cy5–3**, **Cy5–4**, and **Cy5–5**) in good yields (Scheme 1). It is noteworthy that benzothiazole derivatives yielded the corresponding cyanine derivatives in high yields under mild conditions. Conversely, the utilization of indolenine Fischer’s bases necessitated elevated temperatures and prolonged reaction times. Additionally, Fischer’s base derivatives **1c**, **1d** and **1e** successfully afforded the corresponding 1,7-dicyano Cy7 dyes in moderate to good yields when reacted with **10** (Scheme 2).

However, no Cy7 formation was observed upon the reaction of these bases with a chlorohexatrienyl derivative **6**. One possible explanation for the reduced reactivity is the suppression of the activation of the vinylic halide end due to a longer conjugated polyene system. A variety of agents activating enaminaldehyde **8**, such as Ac_2_O, trifluoracetic acid, or Tf_2_O, were examined. However, no significant improvement in reactivity was observed. POBr_3_ resulted in the partial decomposition of the starting material. To circumvent this limitation, an alternative synthetic approach was investigated. The reaction of α-(1,1,3,3-tetramethylindan-2-ylidene)­acetonitriles with *n*-BuLi at – 78 °C was shown to give the corresponding α-cyanovinyl carbanion, which can subsequently be trapped by various aldehydes or ketones.^33^ We assumed that the lithiation of Fischer’s base derivatives **1a** would lead to the formation of an intermediate **11**, and after addition of aldehyde **8** followed by subsequent elimination under acidic conditions, Cy7 can be obtained (Scheme 3). Fischer’s base **1a** and **1f** facilitated the efficient production of **Cy7–16** and **Cy7–17**, while the use of other Fischer’s bases resulted only in marginal formation of **11**.

**Scheme 3:**
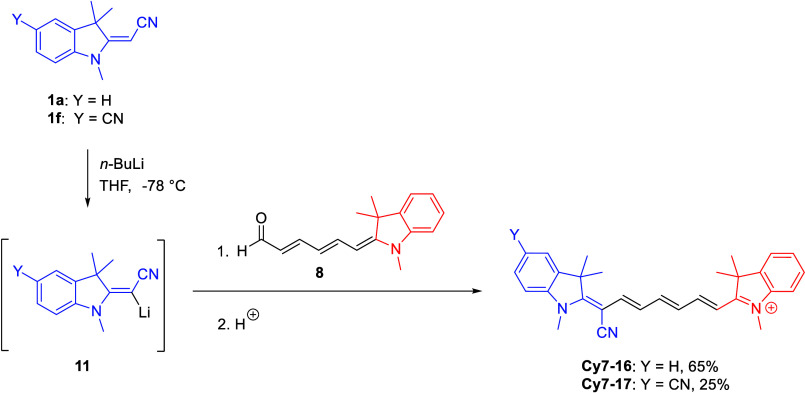
Synthesis of 1′-Substituted Cy7s by Lithiation of Fischer’s Base

The presence of electron-donating substituents on Fischer’s bases results in rapid decomposition, making those compounds challenging to manipulate.^34^ However, the use of quaternary salts **3** or **4** in the presence of a base was found to be an effective approach for the preparation of 1′-substituted Cy5s and Cy7s.^29^ Their use is advised due to their stability and uncomplicated preparation. For instance, indolenine-based quaternary salts **3a** ^29^ and **3b** (Scheme 1) were obtained by Fischer’s indolization^35^ of phenylhydrazine with a suitable ketone, followed by *N*-methylation. Benzothiazole analogs **4a**, **4b**, **4c**,^29^ **4d**,^36^ **4e**, **4f**, **4g**, **4h**, **4i**, and **4j** (Scheme 1) were synthesized through a two-step process involving the condensation of 2-aminothiophenol with α-substituted acyl chloride, subsequently followed by methylation, or through functional group manipulation from 2-(bromomethyl)­benzo­[*d*]­thiazole.

The condensation of 2-substituted heterocycles with **5** or **6** (Scheme 1), or with dianilide hydrochloride glutaconaldehyde **10** (Scheme 2), resulted in the preparation of 21 novel 1′-substituted Cy5 and Cy7 derivatives (Figure 1). This procedure demonstrates broad group tolerance, accommodating bulky 1′-substituents such as the phenyl group in **Cy7–5**, electron-withdrawing (ethoxycarbonyl, methylcarbonyl) groups in **Cy5–5** and **Cy5–3**, or an electron-donating (methoxy) group in **Cy5–7**. Furthermore, *t*-butyl­(dimethyl)­silyl and *t*-butyloxycarbonyl protecting groups exhibited stability throughout the reaction sequence, thereby enabling the isolation of **Cy7–9** and **Cy7–10** in moderate yields (Scheme 1). **Cy7–9** degrades in a methanol solution within a few hours. In general, the employment of Fischer’s base **1** or **2** resulted in higher yields of Cy5s. Conversely, the use of Fischer’s bases for the synthesis of Cy7s proved to be rather unproductive. The preparation of 1′-substituted Cy7s bearing electron-withdrawing groups with this method is challenging. Lithiation of Fischer’s bases **1a** or **1f** led to the partial resolution of the issue, resulting in the formation of **Cy7–16** and **C7–17**, respectively (Scheme 3). Unfortunately, this approach was not applicable in general. We found that other Fischer’s bases, namely **2b** and **1b**, did not undergo lithiation.

To expand the scope of this methodology, we focused on developing new synthetic aldehyde precursors. Despite the established knowledge concerning the reaction of 1,3,3-trimethyl-2-methyleneindoline with commercially available 3-dimethylaminoacrolein to form **7** (Scheme 1),^37^ there is a notable absence of literature documenting syntheses starting from other heterocyclic analogs such as benzothiazolium derivatives or the employment of 2-substituted methylideneindoline for the preparation of enaminaldehydes. 3-Methyl-2-methylbenzothiazolium **2c** exists exclusively as a dimer,^38,39^ but its reactivity toward an electrophile was maintained in the reaction of **2c** with **12**, yielding **13**, or with pyrylium tetrafluoroborate salt^40^ **14**, affording **15** in moderate yields (Scheme 4A).

**Scheme 4:**
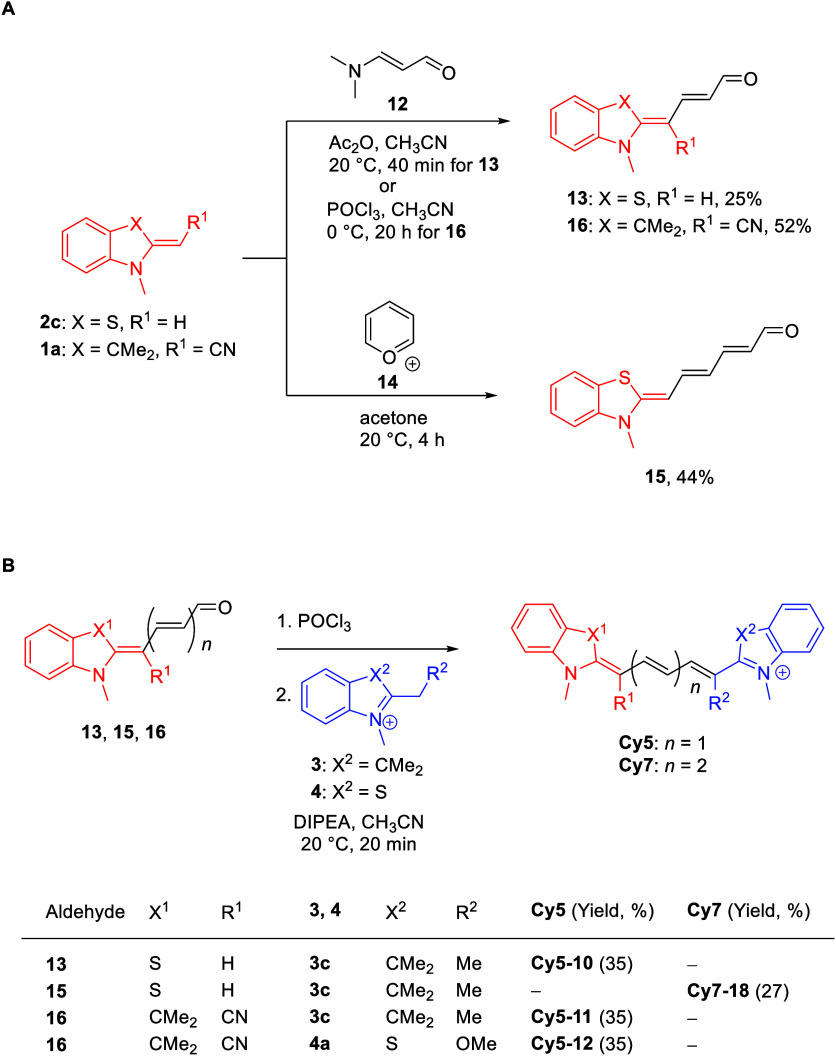
Synthesis of (A) Aldehyde Precursors **13**, **15**, and **16** and (B) the Corresponding Cy5s and Cy7s

Subsequently, the utilization of modified Fischer’s bases was examined. For instance, the cyano derivative **1a** reacted with **12** to yield the desired **16** (Scheme 4A) in a 52% yield. A series of experiments were conducted to facilitate the opening of the pyrylium ring of **14** using **1a**. However, these attempts led to the decomposition of the reactive precursor **14** after a prolonged reaction time. Furthermore, the use of heterocycles other than **1a** for the preparation of 1′-substituted aldehydes resulted in significant degradation of the starting material. Three new aldehyde derivatives (**13**, **15**, **16**) were employed in the synthesis of Cy5s **Cy5–10**, **Cy5–11**, **Cy5–12**, and one Cy7 **Cy7–18** (Scheme 4B, Figure 1). The compounds were obtained in similar yields to those made using the derivatives **7** and **8** (Figure 1). **16** was obtained as a 1:1 mixture of *E* and *Z* isomers (^1^H NMR; Figure S30). This mixture was used in the next step, affording **Cy5–11** and **Cy5–12** exclusively as the *E*-isomers (Scheme 4B). The introduction of unsymmetrical cyanines with various heterocyclic end groups, such as indolenine and benzothiazole moieties in **Cy5–10** and **Cy7–18**, was driven by interest in their potentially unique spectroscopic properties, already observed on other cyanine derivatives.^10,41–43^

The preparation of 1′,5′-disubstituted Cy5s and 1′,7′-disubstituted Cy7s has not been documented in the scientific literature, except for dicyano derivatives **Cy5–9** and **Cy7–12** reported by us.^29^ However, the synthesis of 1′-substituted aldehyde precursors continued to pose a substantial challenge. The sensitivity of chloroiminium salt derived from **12** and pyrilium ion **14** toward bases rendered the use of quaternary nitrogen salts impractical. The usefulness of Fischer’s base was demonstrated by its ability to form the corresponding 1′-substituted aldehyde, albeit in low yields and at trace levels in most instances. It is noteworthy that Fischer’s base **1a** played a fundamental role in the synthesis of 1′-substituted aldehyde **16**.

### Absorption and Emission Properties

The absorption maxima of Cy5 and Cy7 derivatives with high molar absorption coefficients (*ε* > 200,000 M^–1^ cm^–1^; S_0_→S_1_) are typically found in the ranges of 520–640 nm and 655–740 nm, respectively.^14,16,44^ The impact of electron-withdrawing substituents on the absorption spectra of chain-substituted Cy dyes was reported^14,45,46^ and attributed to the alteration of charge delocalization. Recently, we demonstrated that not only the electronic nature but especially the steric demands of bulky 1′-substituents can influence the absorption properties of Cys.^29^

The absorption spectra and additional physicochemical data of the reported and currently prepared 1′-substituted and disubstituted Cy5s (Figure 2A) and Cy7s (Figure 2B) are presented in Table 1. The major absorption maxima of Cys bearing electron-donating methyl (**Cy5–10**), methoxy (**Cy5–7**, **Cy7–3**), and methylthio (**Cy7–7**) substituents in methanol were found in the same region as those of the parent **Cy5–1** and **Cy7–1** derivatives. A more pronounced hypsochromic shift compared to that of **Cy5–1** was found for Cy5s bearing an electron-withdrawing group, such as methylcarbonyl (**Cy5–3**) and ethoxycarbonyl (**Cy5–5**) (λ_max_ ∼ 530 nm). The replacement of the methoxy group of **Cy7–3** with the bulkier *t*-butyldimethylsilyloxy (OTBS) group in **Cy7–9** resulted in a hypsochromic shift and band broadening of ∼ 100 nm. In contrast, the nature of the heterocyclic end group played a marginal role on the absorption spectra.

**Table 1:**
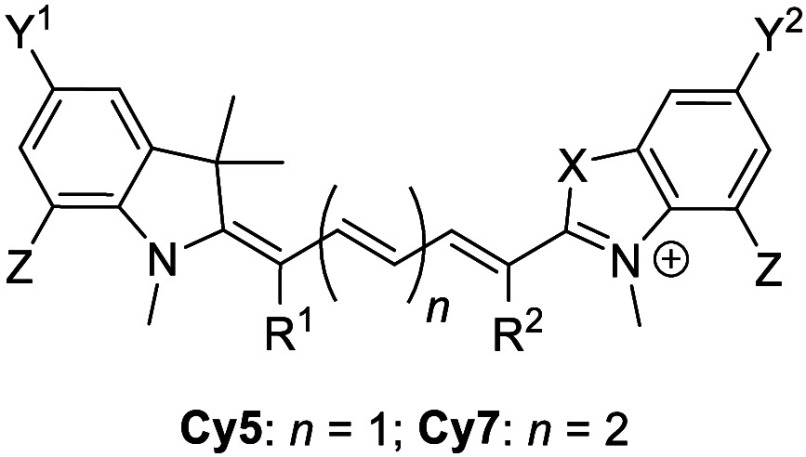
Photophysical Properties of the Studied Cyanines

Pentamethine Cyanines (*n* = 1)						
Cy	λ_abs_/nm^a^	λ_em_/nm^a^	ε/10^5^ ^b^	Φ_F_ ^c^	Φ_dec_/10^–6^ ^d^	Φ_Δ_/10^–3^ ^e^
**Cy5–1** ^f^	638	665	1.96	0.17 ± 0.01	0.37 ± 0.01	1.9 ± 0.3
**Cy5–2** ^f^	599	645	0.69	0.047 ± 0.002	0.26 ± 0.02	0.48 ± 0.06
**Cy5–3**	531	nonemissive	0.41		1.9 ± 0.2	1.9 ± 0.2
**Cy5–4** ^f^	616	652	1.14	0.037 ± 0.003	0.8 ± 0.10	1.3 ± 0.1
**Cy5–5**	600	656	0.59	0.0075 ± 0.0001	13 ± 2	2.5 ± 0.7
**Cy5–6** ^f^	374, 652	688	0.39	0.001 ± 0.000	0.62 ± 0.06	n.d.^g^
**Cy5–7**	649	693	0.87	0.0030 ± 0.0003	50 ± 5	0.32 ± 0.07
**Cy5–8**	649	681	1.30	0.011 ± 0.002	78 ± 7	2.1 ± 0.2
**Cy5–9** ^f^	610	671	1.06	0.007 ± 0.001	0.31 ± 0.06	2.1 ± 0.2
**Cy5–10**	653	684	1.28	0.0042 ± 0.0002	1.0 ± 0.2	n.d.^h^
**Cy5–11**	347, 537	nonemissive	0.30		n.d.^g^	n.d.^h^
**Cy5–12**	340, 519	nonemissive	0.61		n.d.^g^	n.d.^h^

Heptamethine Cyanines (*n* = 2)						
Cy	λ_abs_/nm^a^	λ_em_/nm^a^	ε/10^5^ ^b^	Φ_F_ ^c^	Φ_dec_/10^–6^ ^d^	Φ_Δ_/10^–3^ ^e^
**Cy7–1**	740	769	2.39	0.16 ± 0.02	3.1 ± 0.2	8.9 ± 0.1
**Cy7–2**	747	795	1.05	0.018 ± 0.002	2.5 ± 0.3	2.6 ± 0.1
**Cy7–3**	745	806	0.98	0.0227 ± 0.0070	6.0 ± 0.3	1.3 ± 0.2
**Cy7–4** ^f^	638	790	0.51	0.052 ± 0.009	19 ± 3	5.9 ± 0.5
**Cy7–5**	744	806	0.44	0.0256 ± 0.0010	7.3 ± 0.3	3.4 ± 0.5
**Cy7–6**	753	793	1.18	0.033 ± 0.002	8.3 ± 0.4	10.8 ± 0.7
**Cy7–7**	741	791	1.78	0.0027 ± 0.0004	0.70 ± 0.07	1.00 ± 0.05
**Cy7–8**	741	788	1.20	0.037 ± 0.001	8.0 ± 0.3	7.1 ± 0.6
**Cy7–9**	375, 655	817	0.20	0.0020 ± 0.0002	32 ± 7	1.1 ± 0.1
**Cy7–10**	737	794	0.79	0.024 ± 0.001	7.4 ± 0.3	5.0 ± 0.4
**Cy7–11**	738	788	0.58	0.045 ± 0.004	73 ± 3	7.7 ± 1.5
**Cy7–12** ^f^ ^,^ ^i^	704	744	1.50	0.122 ± 0.005	0.54 ± 0.1	6.3 ± 0.9
**Cy7–13** ^i^	731	788	1.53	0.003 ± 0.001	0.12 ± 0.01	3.21 ± 0.43
**Cy7–14** ^i^	714	773	1.18	0.011 ± 0.001	0.32 ± 0.01	5.27 ± 0.52
**Cy7–15** ^i^	701	738	0.68	0.061 ± 0.001	0.41 ± 0.06	1.11 ± 0.10
**Cy7–16** ^f^	620	744	0.57	0.098 ± 0.014	0.7 ± 0.3	5.2 ± 0.8
**Cy7–17**	577	739	0.87	0.007 ± 0.001	0.70 ± 0.02	n.d.^g^
**Cy7–18**	754	798	1.25	0.017 ± 0.001	22.2 ± 3.4	5.0 ± 0.4

a Absorption (λ_abs_) and emission (λ_em_) maxima obtained in methanol at room temperature.

b Molar absorption coefficients, ε_max_/mol^–1^ dm^3^ cm^–1^, determined in methanol.

c Fluorescence quantum yield (Φ_F_) measured in methanol and obtained as an absolute value using an integrating sphere.

d Quantum yield of photodecomposition (Φ_dec_) calculated relative to that of **Cy5–1** (Φ_dec_ = 3.7 × 10^–7^) or **Cy7–1** (Φ_dec_ = 3.1 × 10^–6^).^14^

e Quantum yield of the singlet oxygen production (Φ_Δ_) determined relative to that of methylene blue (Φ_Δ_ = 0.49)^47^ for Cy5s or **Cy7–1** (Φ_Δ_ = 8.9 × 10^–3^)^14^ for Cy7s.

f Data for these compounds have already been reported^29^ and are provided for comparison.

g Not determined.

h Under the detection limit.

i Measured in 0.1% HCl in methanol.

**Figure 2:**
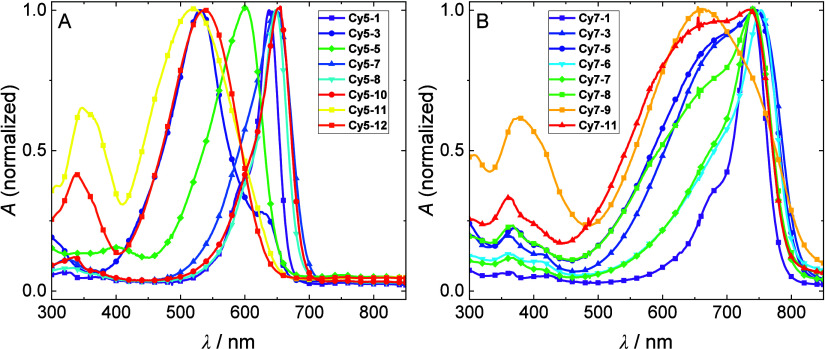
Absorption spectra of 1′-substituted (A) Cy5s and (B) Cy7s in methanol.

The incorporation of a second 5′-group into the 1′-cyano **Cy5–11** and **Cy5–12** derivatives resulted in significant bathochromic shifts of the absorption maxima, along with a reduction in the corresponding molar absorption coefficients. Less intense secondary absorption bands at ∼350 nm were attributed to the S_0_ → S_2_ electronic transition.^29^ This transition was also pronounced in 1′-monosubstituted Cy7 derivatives with bulky and/or electron-withdrawing groups (**Cy7–9**, **Cy7–11**, **Cy7–8**, or **Cy7–5**).

Cys are generally not very bright fluorophores (the fluorescence quantum yield, Φ_F_, is ∼0.16 for both unsubstituted **Cy5–1** and **Cy7–1** in methanol; Table 1)^14,29^ due to radiationless decay and photoisomerization deactivation channels.^48,49^ Structural modifications of Cys can greatly affect Φ_F_.^14,50^ The Φ_F_ values of both previously^29^ and newly synthesized 1′-substituted Cy5s and Cy7s were found to be very small (Φ_F_ < 0.01). No fluorescence was detected for **Cy5–3** and the 1′,5′-disubstituted derivatives **Cy5–11** and **Cy5–12** (Table 1). The emission maxima of Cy5s and Cy7s were found to be less affected by the 1′-substituents than the absorption maxima. A notable increase in Stokes shifts was observed in **Cy7–9**, which exhibited an emission maximum at 817 nm, resulting in a Stokes shift greater than 150 nm.

### Photostability and Formation of Singlet Oxygen

Cyanines with enhanced photostability are highly sought after for their application in biological contexts.^51^ As previously demonstrated, chain substitution can affect the photostability of Cy7s.^14^ While chain substitutions with electron withdrawing amide,^52^ triazole,^53^ fluoride,^54^ or cyano^14^ groups was shown to improve the photostability of Cys, the heavy halogen substituents exhibit an opposite effect, attributable to the elevated production of singlet oxygen (^1^O_2_) via triplet-state sensitization.^10,14,19,55^ The structure of the heterocyclic end groups can also influence the photostability of the dyes. A 7-fold increase in photostability was observed for benzoxazole-based Cy in comparison with benzothiazole-based dye,^56^ which exhibit reduced photostability compared to indole-based cyanines.^57^

In this work, the photostability in methanol was evaluated as the quantum yield of photodecomposition, Φ_dec_, using Φ_dec_ of **Cy5–1** ^29^ and **Cy7–1** ^14^ as references (Table 1). In general, comparable or improved photostability (i.e., lower Φ_dec_ values) was observed for most 1′-substituted Cy5s and Cy7s when compared to that of the parent derivatives. The enhancement was most evident in monocyano and dicyano cyanines, whereas **Cy5–7** and **Cy5–8** were found to be photochemically more reactive. Surprisingly, benzothiazole-based monocyano **Cy5–4** was found to be more photostable than the corresponding indoline-based derivative, **Cy5–2**.

Subsequently, we investigated whether enhanced photostability is associated with decreased production of ^1^O_2_ and subsequent cyanine oxygenation. The production of ^1^O_2_ is utilized for biological applications, such as photodynamic therapy^58^ or NIR-light-triggered uncaging,^59^ but it is recognized as the primary cause of photodecomposition of Cy7.^55^

The influence of the chain substitution pattern on ^1^O_2_ production quantum yield (Φ_Δ_) has already been established for some Cy5s^21^ and Cy7s.^14^ The Φ_Δ_ values for 1′-substituted Cys in methanol were obtained using methylene blue (Φ_Δ_ = 0.49)^60^ and **Cy7–1** ^14^ as references, for Cy5s and Cy7s, respectively (Table 1). Diphenylisobenzofuran (DPBF)^61^ was used as an ^1^O_2_ trap for all the derivatives. The ^1^O_2_ production efficiency is orders of magnitude higher than Φ_dec_; therefore, it was not surprising that the magnitudes of both Φ_Δ_ for Φ_dec_ do not correlate in a simple manner. The maximum values of Φ_Δ_ approached 1%, indicating inefficient formation of the triplet state in the evaluated cyanines, with variations within an order of magnitude. No ^1^O_2_ production was observed for the 1′,5′-disubstituted Cy5s **Cy5–11**, **Cy5–12**, and monosubstituted **Cy5–10**.

### Case Study: 1′-Substituted Cyanines as pH Probes

Numerous case reports have documented the sensitivity of Cy7s to pH variations. For example, pH variability can be detected through the protonation of nonalkylated heterocyclic nitrogen^62–64^ or using a nitrogen-containing substituent incorporated into the Cy scaffold.^65,66^

During the course of our investigation, it was observed that dicyano Cy7 **Cy7–12** exhibited a pH-dependent behavior in protic media such as methanol or water. The distinctive absorption band of Cy7 dyes at ∼700 nm disappeared upon dissolving the compound in a 95:5 (*v*/*v*) water–methanol mixture, yielding a pale-yellow solution with a new absorption maximum at 430 nm. Acidification of the solution by aqueous HCl restored the original band. The p*K*_a_ of the corresponding acid was obtained and recalculated for pure water by an Yasuda-Shedlovsky extrapolation^67,68^ to be at 4.1 ± 0.3 (Figure 3A). Furthermore, the repetitive addition of acid (HCl) and base (NaOH) solutions in five subsequent cycles resulted in the complete reversibility of the process, with no observed decomposition (Figure S134). The fluorescence response of **Cy7–12** to pH variation is demonstrated in Figure 3B. Excitation at 690 nm induced an 11-fold decrease in the emission intensity of **C7–12** at 744 nm when the pH was adjusted from 3.5 to 6.5.

**Figure 3:**
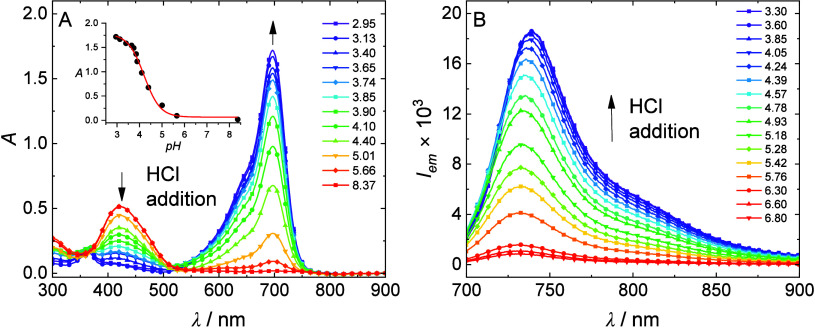
(A) Absorption spectra of **Cy7–12** at different pH values (inset: the pH titration curve). (B) Fluorescence emission (λ_ex_ = 690 nm) of **Cy7–12** at different pH values. All experiments were conducted in a water/methanol (95:5, *v/v*) solution of **Cy7–12** (*c* ∼ 2.7 × 10^–5^ mol L^–1^). The pH was adjusted by consecutive additions of aqueous HCl. The total volume change during titration was corrected for the effects of dilution.

A series of electron-deficient 1′-cyano and 1′,7′-dicyano Cy7 derivatives were tested for their ability to monitor the pH change (Table 2). The heterocyclic methyl substituents in 1′,7′-dicyano **Cy7–14** exhibited a small effect on the p*K*_a_ value when compared to unsubstituted **Cy7–12**, while the dimethoxy derivative **Cy7–13** manifested a higher p*K*_a_ of 6.1. An even higher value of p*K*_a_ ∼ 10 was found for 1′-cyano **Cy7–16** and **Cy7–17** dyes. Practically the same p*K*_a_ values for **Cy7–12**, **Cy7–13**, and **Cy7–16** were obtained using Britton-Robinson buffered solutions with the controlled ionic strength^69^ (*I* = 0.1 M). In all cases, the application of basic pH resulted in a hypsochromic shift of the absorption maxima and quenched the fluorescence emission (Figures S126–S131). Acidification of the solution led to the restoration of the original absorption and emission properties.

**Table 2:** p*K*_a_ Values of Selected Derivatives^a^

Cy	p*K*_a_ ^b^
**Cy7–12**	4.1 ± 0.3 (4.2 ± 0.2^c^)
**Cy7–13**	6.1 ± 0.3 (6.0 ± 0.1^c^)
**Cy7–14**	3.5 ± 0.2
**Cy7–15**	2.5 ± 0.2
	4.4 ± 0.2
	6.8 ± 0.2
**Cy7–16**	10.3 ± 0.2 (10.1 ± 0.1^c^)
**Cy7–17**	10.5 ± 0.2

a Measured in a mixture of water/methanol (95:5, *v/v*; UV–vis spectroscopy); averages of three independent measurements; standard deviation of the mean is given.

b The p*K*_a_ values were obtained by deconvolution of the spectra according to the previously published method^49^ and are recalculated for pure water by an Yasuda-Shedlovsky extrapolation.^67,68^

c The p*K*_a_ values were obtained using Britton-Robinson buffered solutions with the controlled ionic strength *I* = 0.1 M^69^ (see the Supporting Information).

It has previously been demonstrated that the presence of strong alkaline media can promote the reversible attack of hydroxide on the electron-deficient iminium carbon of the indolinium moiety of Cy, leading to a significant hypsochromic shift of the absorption maxima.^70^ To rationalize the pH sensing in our derivatives, we found by ^1^H NMR that the addition of NaOD (3 equiv) to a methanolic solution of **Cy7–17** resulted in the formation of a new species. ^1^H–^1^H COSY and ^1^H–^1^H NOESY experiments then provided evidence that the hydroxide was added to the iminium carbon (Scheme 5, Figures S137–140). This is consistent with the fact that the presence of two cyano moieties in **Cy7–12**, **Cy7–13**, and **Cy7–14** results in a substantial decrease in p*K*_a_, indicating higher sensitivity to hydroxide attack. In contrast, the titration of the dimethylamino derivative **Cy7–15** revealed the concurrent existence of multiple acid–base species, attributable to protonation of several basic sites in the molecule (Figure S129).

**Scheme 5:**
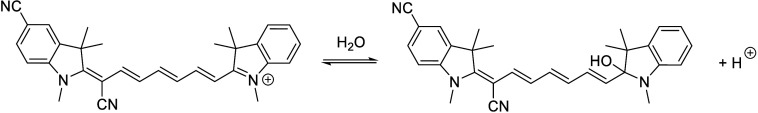
Reversible Addition of Water to the Iminium Carbon Atom of **Cy7–17**

### Sensing of Nucleophiles

The final aim of this study was to test the ability of 1′,7′-dicyano Cy7s to sense nucleophiles. Nucleophiles such as cyanide and fluoride,^71^ or azide,^72^ can irreversibly attack the iminium carbon of Cys. In contrast, soft nucleophiles such as primary thiols and phosphines undergo photoinduced reversible nucleophilic attack to the C2′-position of the Cy5s polymethine chain.^73,74^ In the present study, **Cy7–13** was selected as the model compound. With the p*K*_a_ value of 6.1, a Britton-Robinson buffer^69^ solution (5% of methanol; pH = 5.1) ensured that the probe’s functionality was not compromised by the hydroxide attack. Upon the gradual addition of a NaCN solution (up to 180 equiv), the original absorption band of **Cy7–13** at 722 nm gradually disappeared while a new band at 455 nm emerged (Figure 4A). The ^1^H NMR analysis of the addition of NaCN (3 equiv) to in a methanolic solution of **Cy7–13** containing DCl (2 equiv; to avoid the probe deactivation) revealed that nucleophilic attack of the cyanide to the iminium carbon occurred (Figure S141). Subsequent acidification of the solution by aqueous DCl did not reverse the addition process. This finding is consistent with previous reports of cyanide addition to Cy and hemicyanine derivatives.^75,76^ HPLC analysis showed that the same adduct was formed in Britton-Robinson buffer (Figure S135).

**Figure 4:**
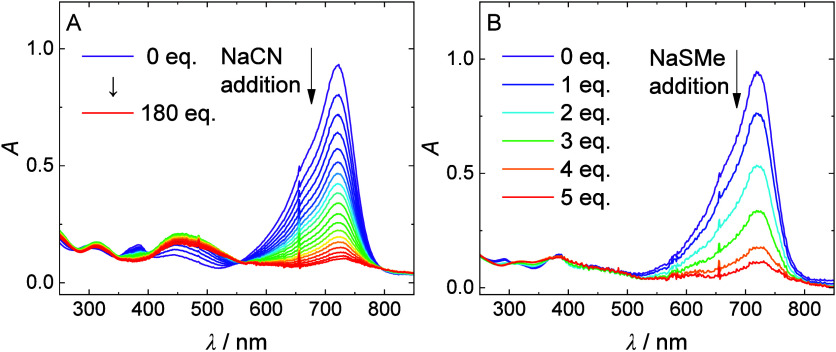
Absorption spectra of **Cy7–13** upon addition of A) NaCN in water (*c* ∼ 2.0 × 10^–1^ mol L^–1^) and B) NaSMe in methanol (*c* ∼ 2.0 × 10^–1^ mol L^–1^). All experiments were conducted in a Britton-Robinson buffer/methanol (95:5, *v/v*) solution of **Cy7–13** (*c* ∼ 2.0 × 10^–5^ mol L^–1^) at pH = 5.1.

Furthermore, **Cy7–13** exhibited a notable sensitivity to methanethiol. The addition of 5 equiv of HSMe to **Cy7–13** in Britton-Robinson buffer at pH 5.1 resulted in the disappearance of the major absorption band of the dye at 700 nm (Figure 4B). The ^1^H NMR (MeOD, DCl) and HPLC analyses showed a mixture of products, and after additional acidification of the solution with DCl, the initial **Cy7–13** spectrum was restored (Figure S142). Supported by ^1^H–^1^H COSY and ^1^H–^1^H NOESY measurement of the mixture, we hypothesize a simultaneous nucleophilic attack of methanethiol at C2′ and C4′ positions of **Cy7–13** (Scheme 6, Figures S143–147). The attack on the iminium carbon is probably sterically hindered.

**Scheme 6:**
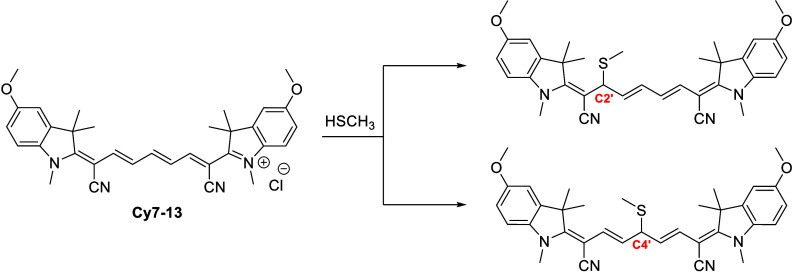
Suggested Products of a Nucleophilic Attack of Methanethiol to C2′ and C4′ of **Cy7-13**

Nucleophiles, such as NCS^–^, and H_2_S, required more than 100 equiv to produce observable spectral changes when **Cy7–13** was used as a probe (Figure S132). No response was observed in the presence of amines, halides, phosphites, or amino acids. Thanks to their p*K*_a_ values, the 1′-cyano Cy7 analogs **Cy7–16** and **Cy7–17** were tested at neutral pH. However, they were found to be considerably less reactive toward methanethiol attack than **Cy7–13**, so they were not used further (Figure S133).

## Conclusions

The condensation of chloroiminium derivatives and dianilide hydrochloride glutaconaldehyde with diverse Fischer’s bases facilitates a straightforward and universally applicable method for the preparation of chain 1′-functionalized Cy5 and Cy7 dyes. This approach offers a high degree of flexibility, enabling the introduction of a broad range of substituents in the chain and heterocyclic end groups. Additionally, it provides access to previously unavailable 1′,5′- and 1′,7′-disubstituted pentamethine and heptamethine cyanines, respectively.

Among the twenty-one new cyanine derivatives introduced, those bearing bulky and/or electron-withdrawing substituents at the C1′ position exhibit significantly altered absorption properties, the phenomenon that has been explained by symmetry breaking, accompanied by bond length alternation and out-of-plane rotation of the end group previously.^29^ This phenomenon is manifested by the appearance of the secondary absorption band around 350 nm, attributed to the S_0_→S_2_ transition (e.g., **Cy5–11** or **Cy7–9**), or by the nearly 100 nm hypsochromic shift in the major absorption maxima (e.g., **Cy5–3**, **Cy7–4**, and **Cy7–16**).

We demonstrate that cyano-substituted cyanines combine excellent photostability with satisfactory fluorescence efficiency, making them highly suitable for diagnostic imaging applications. They exemplify previously undocumented pH sensitivity, reversibly modulating both absorption and emission properties. The p*K*_a_s of the studied cyanines in the broad range of 2.5–10.5 are achievable through targeted structural modification. Such flexibility can be advantageous for the monitoring of pH fluctuations in biological environments with pronounced acidic conditions, such as lysosomes and endosomes. Finally, some of the synthesized cyanines, such as **Cy7–13**, demonstrated sensitivity to the presence of nucleophiles, such as cyanide or methanethiol, suggesting their potential application as probes for selective detection of nucleophilic species in chemical or biological systems.

## Experimental Section

### Synthesis

Pentamethine cyanines **Cy5–2**, **Cy5–4**, **Cy5–6**, and **Cy5–9**, heptamethine cyanines **Cy7–4**, **Cy7–12**, and **Cy7–16**, aldehydes **7** and **8**, Fischer’s base **1a**, and heterocyclic salts **3a**, **3c**, and **4c** were prepared according to our previously published procedures.^29^

### Substituted Benzothiazoles, Indoles, and Aldehyde Precursors

#### (*E*)-1-(1,3,3-Trimethylindolin-2-ylidene)­propan-2-one (**1b**)

The title compound was prepared according to a reported procedure.^31^ 1,3,3-Trimethyl-2-methyleneindoline (Fischer’s base; 1.8 mL, 10.4 mmol) was added to 4 mL of freshly distilled acetic anhydride and heated (heating mantle) at 110 °C for 2 h. After cooling, ethyl acetate (10 mL) was added to a pink solution, which was then poured into 50 mL of water. The organic phase was extracted and then washed with a 10% NaOH solution. After the removal of the solvent, the title compound was purified using column chromatography (silica gel; toluene/ethyl acetate, 85:15). Yield: 1.5 g (67%). Orange solid. ^1^H NMR (300 MHz, CDCl_3_) δ (ppm): 7.24–7.16 (m, 2H), 7.01 (m, 1H), 6.78 (m, 1H), 5.31 (s, 1H), 3.25 (s, 3H), 2.22 (s, 3H), 1.70 (s, 6H). The NMR data are consistent with those reported in the literature.^31^ 
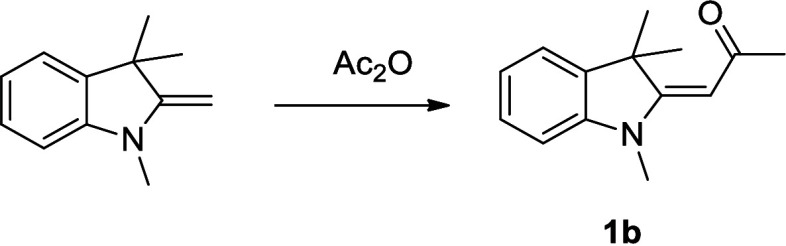


#### (*Z*)-2-(3-Methylbenzo­[*d*]­thiazol-2­(3*H*)-ylidene)­acetonitrile (**2a**)

The title compound was prepared according to the reported procedure.^26^ 3-Methyl-2-methylthio-benzothazolium tosylate^26^ (0.8 g; 2.3 mmol) was dissolved in pyridine (6 mL). Cyanoacetic acid (0.23 g; 2.7 mmol) was added, followed by the addition of triethylamine (0.38 mL; 5.2 mmol). The reaction was stirred overnight at room temperature. The solvent was removed under reduced pressure, and water (∼30 mL) was added. The product was collected by filtration. Yield: 0.24 g (55%). Brown solid. ^1^H NMR (300 MHz, *d*_6_-DMSO) δ (ppm): 7.66 (m, 1H), 7.39–7.21 (m, 2H), 7.11 (s, 1H), 4.71 (s, 1H), 3.38 (s, 3H). The NMR data are consistent with those reported in the literature.^26^ 
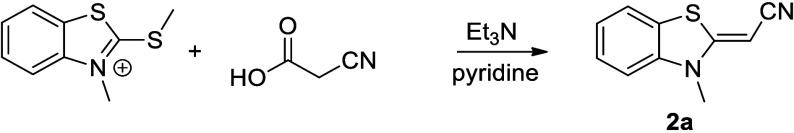


#### Ethyl (*Z*)-2-(3-methylbenzo­[*d*]­thiazol-2­(3*H*)-ylidene)­acetate (**2b**)

2-Ethoxycarbonylmethyl-3-methyl-benzothiazolium^77^ (0.3 g, 1.0 mmol) was suspended in 5 mL of aq NaOH (30%). Diethyl ether (10 mL) was added, and the reaction was stirred at room temperature for 2 h. Water was removed, and an organic phase was distilled under reduced pressure to afford the title compound. Yield: 168 mg (92%). Off-white solid. ^1^H NMR (300 MHz, *d*_6_-DMSO) δ (ppm): 7.67 (d, *J* = 7.7 Hz, 1H), 7.34 (d, *J* = 7.7 Hz, 2H), 7.18–7.07 (m, 1H), 5.31 (s, 1H), 4.09 (q, *J* = 7.1 Hz, 2H), 3.48 (s, 3H), 1.21 (t, *J* = 7.1 Hz, 3H). The NMR data are consistent with the literature.^32^ 
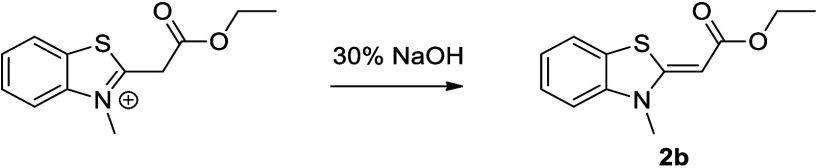


#### (*Z*)-2,3-Dimethyl-2-((3-methylbenzo­[*d*]­thiazol-2­(3*H*)-ylidene)­methyl)-2,3-dihydrobenzo­[*d*]­thiazole (**2c**)

2,3-Dimethylbenzo­[*d*]­thiazol-3-ium methosulfate^78^ (2 g; 7.3 mmol) was dissolved in dichloromethane (30 mL), and a 10% KOH solution (30 mL) was added dropwise. After stirring for 20 min, the reaction mixture was extracted with diethyl ether. The solvent was removed under reduced pressure to afford the title compound. Yield: 0.64 g (54%). Pink crystals. ^1^H NMR (300 MHz, CDCl_3_) δ (ppm): 7.24–7.00 (m, 4H), 6.78 (m, 3H), 6.40 (d, *J* = 7.8 Hz, 1H), 4.48 (d, *J* = 7.8 Hz, 1H), 3.28 (s, 3H), 2.66 (s, 3H), 1.71 (d, *J* = 6.0 Hz, 3H). The NMR data are consistent with the literature.^38^ 
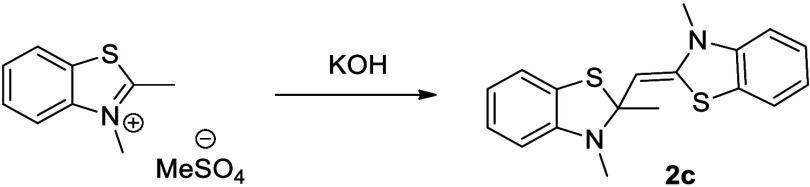


#### 2-(But-3-yn-1-yl)-3-methylbenzo­[*d*]­thiazol-3-ium Triflate (**3b**)

Phenylhydrazine (0.1 g, 0.92 mmol) and 3-methyl-1-phenylbutan-2-one^79^ (0.22 g, 1.4 mmol) were dissolved in acetic acid (2 mL), and the solution was refluxed (heating mantle) for 2 h. After cooling the reaction mixture, a saturated solution of NaHCO_3_ (10 mL) was added, and the solution was extracted 2× with ethyl acetate (5 mL). Product was purified by silica gel chromatography (*n*-hexane/ethyl acetate, 10:1). The corresponding 2-benzyl-3,3-dimethyl-3*H*-indole^80^ was obtained as yellow oil. Subsequently, this compound (0.14 g; 0.60 mmol) was dissolved in diethyl ether (25 mL). Methyl trifluoromethanesulfonate (0.19 g, 1.2 mmol) was added dropwise under vigorous stirring, and the reaction was stirred overnight under the N_2_ atmosphere at room temperature. The resulting precipitate was filtered off and washed several times with diethyl ether and *n*-pentane. Yield: 0.09 g (37%). White solid. The compound was used in the next step without further purification.
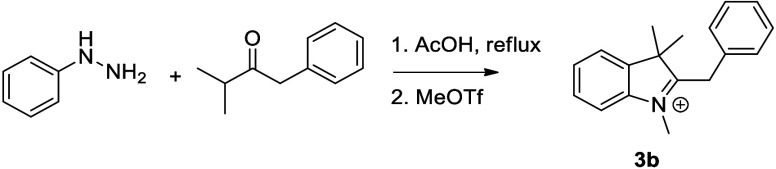


#### (2*E*,4*Z*)-4-(3-Methylbenzo­[*d*]­thiazol-2­(3*H*)-ylidene)­but-2-enal (**13**)

3-Dimethylaminoacrolein (1.0 g; 10.1 mmol) was added dropwise to acetic anhydride (5 mL) under stirring. After stirring for additional 20 min, a solution of **2c** (0.65 g, 1.9 mmol) in acetonitrile (5 mL) was added dropwise using a syringe pump over 20 min. After stirring for additional 20 min, the reaction mixture was poured into a solution of 20% NaOH (20 mL) and extracted with dichloromethane (30 mL). After the solvent was removed under reduced pressure, the product was purified by silica gel chromatography (*n*-hexane/ethyl acetate, 1:1). Yield: 0.11 g (25%). Yellow solid. ^1^H NMR (500 MHz, *d*_6_-DMSO) δ (ppm): 9.32 (d, *J* = 8.3 Hz, 1H), 7.67 (d, *J* = 8.3 Hz, 1H), 7.37–7.27 (m, 3H), 7.10–7.15 m, 1H), 5.78–5.90 (m, 2H), 3.49 (s, 3H). ^13^C­{^1^H} NMR (126 MHz, *d*_6_-DMSO) δ (ppm): 191.3, 157.6, 151.1, 142.7, 127.5, 123.8, 122.9, 122.5, 120.6, 111.2, 91.4, 32.3 (Figures S26 and S27). HRMS (APCI-TOF) *m*/*z*: [M + H]^+^ calcd for C_12_H_11_NOS^+^ 218.0634; found 218.0632.
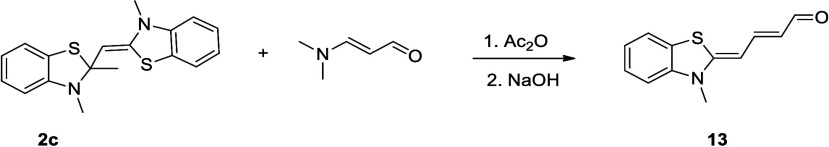


#### (2*E*,4*E*,6*Z*)-6-(3-Methylbenzo­[*d*]­thiazol-2­(3*H*)-ylidene)­hexa-2,4-dienal (**15**)

**2c** (0.5 g; 1.5 mmol) was dissolved in acetone (250 mL). Under vigorous stirring, finely ground pyrylium tetrafluoroborate^40^ (0.20 g; 1.2 mmol) was added. The reaction was stirred at room temperature for 4 h and on an hourly basis. After the solvent was removed, the crude material was dissolved in dichloromethane (30 mL) and extracted with a 1% KOH solution (50 mL). Dichloromethane was removed and methanol (5 mL) was added. The product was collected by filtration. Yield: 0.13 g (44%). Red solid. ^1^H NMR (500 MHz, CD_2_Cl_2_) δ (ppm): 9.47 (d, *J* = 8.1 Hz, 1H), 7.41 (d, *J* = 8.1 Hz, 1H), 7.32–7.19 (m, 3H), 7.06 (t, *J* = 8.1 Hz, 1H), 6.95 (d, *J* = 8.1 Hz, 1H), 6.88 (dd, *J* = 11.6 Hz, 1H), 6.27 (d, *J* = 11.6 Hz, 1H), 6.02–5.95 (m, 1H), 5.61 (d, *J* = 11.6 Hz, 1H), 3.39 (s, 3H). ^13^C­{^1^H} NMR (126 MHz, CD_2_Cl_2_) δ (ppm): 192.5, 154.0, 153.4, 142.8, 141.5, 126.5, 124.9, 124.2, 121.7, 121.5, 119.8, 109.1, 92.0, 31.4 (Figures S28 and S29). HRMS (APCI-TOF) *m*/*z*: [M + H]^+^ calcd for C_14_H_14_NOS^+^ 244.0791; found 244.0792.
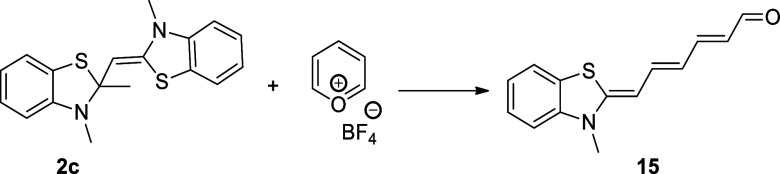


#### (*E*)-5-Oxo-2-((*Z*)-1,3,3-trimethylindolin-2-ylidene)­pent-3-enenitrile (**16**)

Dimethylaminoacrolein (0.425 g; 4.3 mmol) was added to dry acetonitrile (2.5 mL). Then, POCl_3_ (0.4 mL; 4.3 mmol) was added to the stirred solution at 0 °C. After 10 min, a solution of (*Z*)-2-(1,3,3-trimethylindolin-2-ylidene)­acetonitrile^18^ (**1a**; 0.3 g; 1.7 mmol) in dry acetonitrile (2 mL) was added dropwise at 0 °C. After 20 min, the ice bath was removed, and the reaction was stirred overnight. Subsequently, the mixture was poured into a 5% NaOH solution (20 mL) in small portions and was stirred for 20 min. Ethyl acetate (∼30 mL) was added, and the organic phase was washed two times with water (50 mL). After the solvent was removed under reduced pressure, the product was purified by silica gel chromatography (*n*-hexane/ethyl acetate, 2:1). The product was obtained as a mixture of isomers (1:1). Yield: 0.2 g (52%). Pale yellow solid. ^1^H NMR (500 MHz, *d*_6_-DMSO) δ (ppm): 9.69 (d, *J* = 8.0 Hz, 1H), 9.58 (d, *J* = 8.0 Hz, 1H), 8.01 (m, 2H), 7.50 (dd, *J* = 13.9, 8.0 Hz, 2H), 7.36 (t, *J* = 7.3 Hz, 2H), 7.26 (dd, *J* = 13.9, 8.0 Hz, 2H), 7.19 (dd, *J* = 13.9, 8.0 Hz, 2H), 6.00 (m, 2H), 3.79 (d, *J* = 6.2 Hz, 3H), 3.72 (s, 3H), 1.65 (d, *J* = 12.6 Hz, 12H). ^13^C­{^1^H} NMR (126 MHz, *d*_6_-DMSO) δ (ppm): 193.1, 192.9, 149.2, 148.7, 128.7, 124.6, 124.3, 123.0, 122.4, 122.4, 110.52, 40.82, 39.9, 39.8, 39.6, 39.5, 36.4, 33.7, 26.9, 25.2 (Figures S30 and S31). HRMS (ESI-TOF) *m*/*z*: [M + H]^+^ calcd for C_16_H_17_N_2_O^+^ 253.1335; found 253.1333.
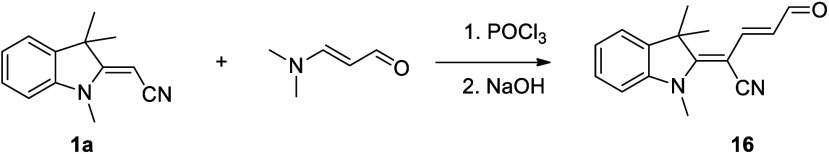


#### 2-(Methoxymethyl)­benzo­[*d*]­thiazole (**17**)

The title compound was prepared according to a reported procedure.^81^ 2-Aminothiophenol (0.6 g; 4.8 mmol) was suspended in 50 mL of water. Methoxyacetyl chloride (0.52 g; 5.8 mmol) was added dropwise. The resulting mixture was stirred at 35 °C (heating mantle) for 2 h. After cooling, the reaction mixture was extracted with dichloromethane (20 mL). After the solvent was removed under reduced pressure, the product was purified by silica gel chromatography (*n*-hexane/ethyl acetate, 10:1). Yellow oil. Yield: 0.53 g (61%). ^1^H NMR (300 MHz, *d*_6_-DMSO) δ (ppm): 8.12 (d, *J* = 8.0 Hz, 1H), 7.98 (d, *J* = 8.0 Hz, 1H), 7.56–7.41 (m, 2H), 4.86 (s, 2H), 3.46 (s, 3H). The NMR data are consistent with those reported in the literature.^81^ 
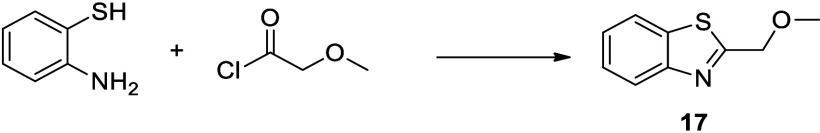


#### 2-(Chloromethyl)­benzo­[*d*]­thiazole (**18**)

The title compound was prepared according to the reported procedure.^82^ 2-Aminothiophenol (1.0 g; 8.1 mmol) was dissolved in acetic acid (15 mL). After 10 min, chloroacetyl chloride (1.35 g; 12.0 mmol) was added dropwise under vigorous stirring. The reaction was stirred for 3 h at room temperature, and then ethyl acetate (30 mL) was added. The organic phase was washed 3× with water (∼50 mL). After the solvent was removed under reduced pressure, the product was purified by silica gel chromatography (*n*-hexane/ethyl acetate, 10:1). Yield: 1.2 g (80%). Yellow oil. ^1^H NMR (300 MHz, *d*_6_-DMSO) δ (ppm): 8.16 (t, *J* = 10.8 Hz, 1H), 8.04 (t, *J* = 10.8 Hz, 1H), 7.53 (m, 2H), 5.24 (s, 2H). The NMR data are consistent with those reported in the literature.^82^ 
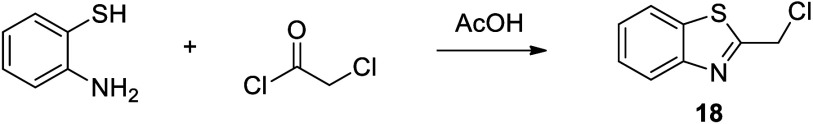


#### 5-Methoxy-2,3,3-trimethyl-3*H*-indole (**19**)

The title compound was prepared by the modification of a known procedure.^83^ 4-Methoxyphenylhydrazine hydrochloride (2.5 g; 14.5 mmol) and methyl isopropyl ketone (3.01 mL; 28.0 mmol) are dissolved in acetic acid (50 mL) and the solution was refluxed (heating mantle) for 2 h. After cooling the reaction mixture, a solution of 10% NaOH (100 mL) was added, and the solution was extracted 2× with ethyl acetate (30 mL). The removal of the solvent under reduced pressure gave the product. Yield: 2.55 g (94%). Dark oil. ^1^H NMR (300 MHz, CDCl_3_) δ (ppm): 7.48 (d, *J* = 8.1 Hz, 1H), 6.85 (s, 2H), 3.85 (s, 3H), 2.28 (s, 3H), 1.31 (s, 6H). The NMR data are consistent with those reported in the literature.^83^ 
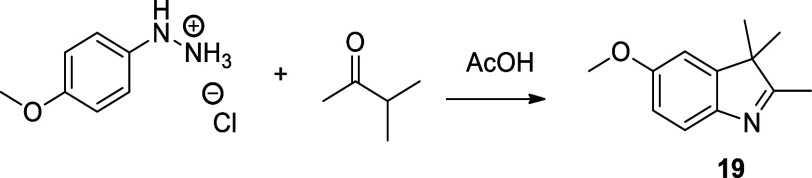


#### 2,3,3,5,7-Pentamethyl-3*H*-indole (**20**)

The title compound was prepared by the modification of a known procedure.^84^ 2,4-Dimethylphenylhydrazine hydrochloride (3.4 g; 20.0 mmol) and methyl isopropyl ketone (3.22 mL; 30.0 mmol) were dissolved in acetic acid (30 mL), and the solution was refluxed (heating mantle) for 3 h. After cooling, the reaction mixture was poured into a 10% NaOH solution (100 mL), and the mixture was extracted 2× with ethyl acetate (30 mL). The removal of the solvent under reduced pressure gave the product. Yield: 3.1 g (83%). Dark oil. ^1^H NMR (300 MHz, CDCl_3_) δ (ppm): 6.95 (d, *J* = 4.6 Hz, 2H), 2.57 (s, 3H), 2.38 (s, 3H), 2.34 (s, 3H), 1.30 (s, 6H). The NMR data are consistent with those reported in the literature.^84^ 
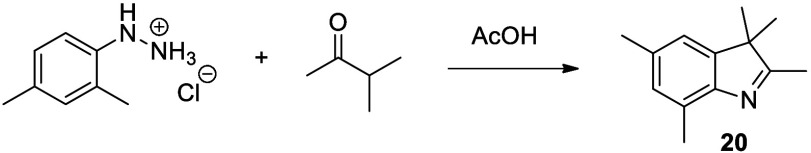


#### 2,3,3-Trimethyl-3*H*-indol-5-amine (**21**)

The title compound was prepared by the modification of a known procedure.^85^ 2,3,3-Trimethyl-3*H*-indole (3.0 g; 18.9 mmol) was dissolved in 96% H_2_SO_4_ (12 mL). KNO_3_ (2.0 g; 19.8 mmol) suspended in 96% H_2_SO_4_ (12 mL) was added dropwise at 0 °C under vigorous stirring. After 30 min, the resulting red solution was added slowly to water (100 mL) and neutralized at 0 °C with a 20% NaOH solution (150 mL). The mixture was extracted 2× with ethyl acetate (30 mL). The solvent was removed under reduced pressure to give 2,3,3-trimethyl-5-nitro-3*H*-indole, which was used without further purification in the next step. 3.6 g (93%). Dark solid. ^1^H NMR (300 MHz, *d*_6_-DMSO) δ (ppm): 8.41 (s, 1H), 8.23 (d, *J* = 8.5 Hz, 1H), 7.65 (d, *J* = 8.5 Hz, 1H), 2.30 (s, 3H), 1.33 (s, 6H).

2,3,3-Trimethyl-5-nitro-3*H*-indole (2.0 g; 9.8 mmol) and stannous chloride (SnCl_2_; 12 g, 65.3 mmol) were suspended in water (30 mL). 35% HCl (30 mL) was added, and the reaction was stirred (heating mantle) at 100 °C for 2 h. After cooling, a 10% NaOH solution was added dropwise until the basic pH was reached. The mixture was extracted 2× with ethyl acetate (30 mL). The solvent was removed under reduced pressure. Yield: 1.25 g (73%). Brown solid. ^1^H NMR (300 MHz, CDCl_3_) δ (ppm): 7.36 (d, *J* = 7.9 Hz, 1H), 6.65 (s, 2H), 2.30 (s, 3H), 1.30 (s, 6H). The NMR data are consistent with those reported in the literature.^86^ 
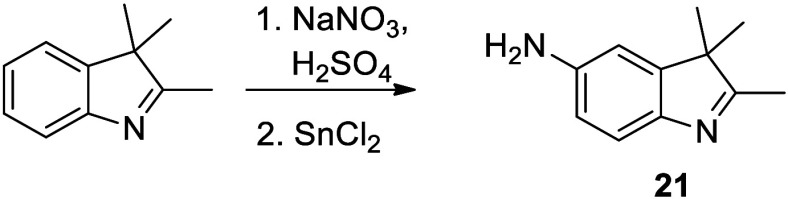


#### 2,3,3-Trimethyl-3*H*-indole-5-carbonitrile (**22**)

The title compound was prepared by the modification of a known procedure.^87^ 4-Cyanophenylhydrazine hydrochloride (2 g; 11.2 mmol) and methyl isopropyl ketone (4 mL; 37.2 mmol) was dissolved in acetic acid (20 mL) and the reaction was stirred at reflux (heating mantle) overnight. After cooling the reaction mixture, a saturated solution of NaHCO_3_ (100 mL) was added, and the solution was extracted 2× with ethyl acetate (30 mL). The solvent was removed under reduced pressure, and the product was purified by chromatography on silica gel (*n*-hexane/ethyl acetate, 3:1). Yield: 1.13 g (51%). Red solid. ^1^H NMR (300 MHz, *d*_6_-DMSO) δ (ppm): 7.98 (s, 1H), 7.77 (d, *J* = 9.4 Hz, 1H), 7.60 (d, *J* = 9.4 Hz, 1H), 2.27 (s, 3H), 1.28 (s, 6H). The NMR data are consistent with those reported in the literature.^38^ 
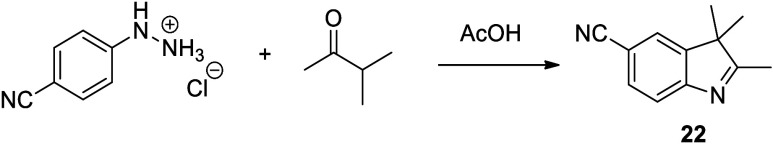


#### 2-Benzylbenzo­[*d*]­thiazole (**23**)

The title compound was prepared according to the reported procedure.^88^ 2-Aminothiophenol (1.2 g; 9.6 mmol) and phenylacetic acid (1.7 g; 12.5 mmol) were dissolved in methanesulfonic acid (9 mL). After the addition of silica (2.8 g), the reaction was stirred at 140 °C (heating mantle) for 3 h. After cooling, the crude mixture was added dropwise to a saturated solution of NaHCO_3_ (50 mL) at 0 °C. The mixture was extracted with ethyl acetate (50 mL), the solvent was removed under reduced pressure, and the product was purified by chromatography on silica gel (*n*-hexane/ethyl acetate, 10:1). Yield: 1.1 g (51%). Yellow oil. ^1^H NMR (300 MHz, *d*_6_-DMSO) δ (ppm): 7.98 (m, 2H), 7.56–7.25 (m, 7H), 4.47 (s, 2H). The NMR data are consistent with those reported in the literature.^88^ 
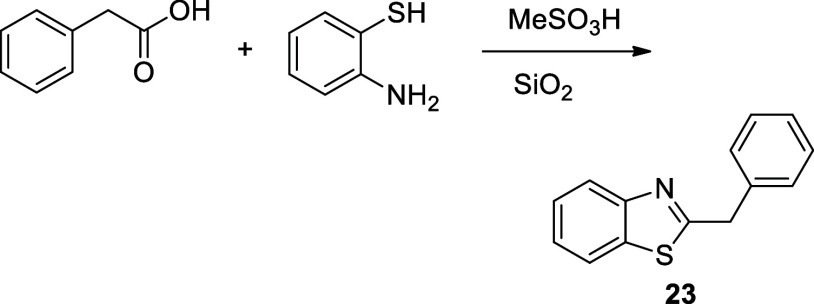


#### 2-((Methylthio)­methyl)­benzo­[*d*]­thiazole (**24**)

The title compound was prepared by the modification of a known procedure.^89^ 2-(Bromomethyl)­benzo­[*d*]­thiazole^90^ (0.27 g; 1.2 mmol) was suspended in methanol (3 mL). A solution of sodium thiomethoxide (0.45 g; 6.4 mmol) in methanol (5 mL) was added dropwise at 0 °C under stirring. The reaction was refluxed (heating mantle) for 3 h and then stirred at room temperature overnight. The reaction was quenched with water (10 mL) and extracted with diethyl ether (10 mL). The organic phase was washed 5× with water (10 mL). The solvent was removed under reduced pressure. Yield: 0.18 g (78%). Yellow oil. ^1^H NMR (500 MHz, *d*_6_-DMSO) δ (ppm): 8.08 (d, *J* = 7.5 Hz, 1H), 7.95 (d, *J* = 7.5 Hz, 1H), 7.51 (t, *J* = 8.3 Hz, 1H), 7.43 (t, *J* = 8.3 Hz, 1H), 4.20 (s, 2H), 2.14 (s, 3H). ^13^C­{^1^H} NMR (126 MHz, *d*_6_-DMSO) δ (ppm): 170.9, 153.23, 135.74, 126.6, 125.6, 122.9, 122.7, 35.4. HRMS (ESI-TOF) *m*/*z*: [M + H]^+^ calcd for C_9_H_9_NS_2_ 196.0249; found 196.0247. The NMR data are consistent with those reported in the literature.^89^ 
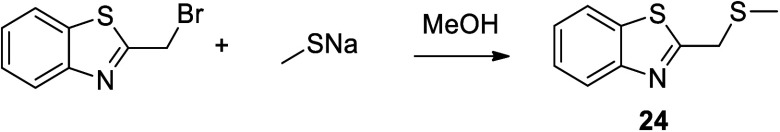


#### Benzo­[*d*]­thiazol-2-ylmethyl Acetate (**25**)

The title compound was prepared by the modification of a known procedure.^89^ 2-(Bromomethyl)­benzo­[*d*]­thiazole^90^ (0.3 g; 1.3 mmol) was dissolved in DMSO (5 mL). Sodium acetate (0.27 g; 3.3 mmol) was added. The reaction was stirred at 70 °C (heating mantle) overnight. After cooling, water (25 mL) was added, and the reaction mixture was extracted with dichloromethane (20 mL). The solvent was removed under reduced pressure. Yield: 0.25 g (92%). Beige solid. ^1^H NMR (300 MHz, *d*_6_-DMSO) δ (ppm): 8.13 (d, *J* = 7.9 Hz, 1H), 8.02 (d, *J* = 7.9 Hz, 1H), 7.51 (m, 2H), 5.50 (s, 2H), 2.17 (s, 3H). The NMR data are consistent with those reported in the literature.^89^ 
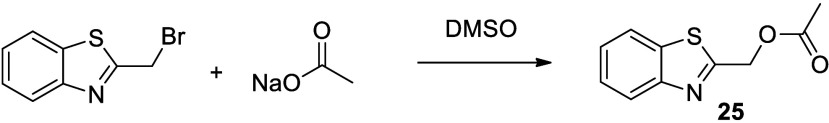


#### *N*-(Benzo­[*d*]­thiazol-2-ylmethyl)­acetamide (**26**)

2-(Azidomethyl)­benzo­[*d*]­thiazole^91^ (1.1 g; 5.8 mmol) was dissolved in methanol (55 mL). Palladium on carbon (0.4 g; 4 mol %) was added to reaction mixture under stirring. The reaction was stirred overnight under a positive pressure of hydrogen. The reaction was filtered on a Celite pad, and the solvent was removed under reduced pressure to give benzo­[*d*]­thiazol-2-ylmethanamine. Yield: 0.72 g (76%). Red oil. ^1^H NMR (300 MHz, D_2_O) δ (ppm): 7.99 (d, *J* = 8.2 Hz, 1H), 7.50 (dt, *J* = 23.1, 8.2 Hz, 2H), 4.62 (s, 2H). The NMR data are consistent with those reported in the literature.^92^

Benzo­[*d*]­thiazol-2-ylmethanamine (0.3 g; 1.8 mmol) was suspended in dry dichloromethane (10 mL). 4-Dimethylaminopyridine (DMAP) (0.27 g; 2.2 mmol) was added under stirring. After 10 min, acetic anhydride (0.2 mL; 2.1 mmol) was added dropwise. The reaction was stirred at room temperature for 1 h. Dichloromethane (10 mL) was added, and the reaction mixture was extracted 2× with water (20 mL) and brine (20 mL). The organic phase was distilled under reduced pressure to afford the title compound. Yield: 0.26 g (70%). Beige solid. ^1^H NMR (300 MHz, *d*_6_-DMSO) δ (ppm): 8.85 (s, 1H), 8.06 (d, *J* = 7.8 Hz, 1H), 7.94 (d, *J* = 7.8 Hz, 1H), 7.46 (m, 2H), 4.65 (d, *J* = 6.0 Hz, 2H), 1.94 (s, 3H). The NMR data are consistent with the literature.^93^ 
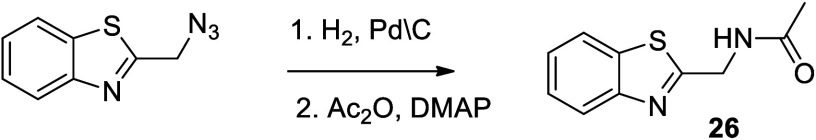


#### 2-(((*tert*-Butyldimethylsilyl)­oxy)­methyl)­benzo­[*d*]­thiazole (**27**)

The title compound was prepared by the modification of a known procedure.^94^ Benzo­[*d*]­thiazol-2-ylmethanol^95^ (0.5 g; 3.0 mmol) was dissolved in pyridine (10 mL), and *tert*-butyldimethylsilyl chloride (TBDMSCl) (1.0 g; 6.6 mmol) was added to the reaction mixture. After stirring the reaction overnight, a saturated solution of NaHCO_3_ (30 mL) was added. The title product was extracted with ethyl acetate (10 mL) and, after the solvent was removed under reduced pressure, it was purified using column chromatography (silica gel; *n*-hexane/ethyl acetate, 10:1). Yield: 0.56 g (67%). Pale pink oil. ^1^H NMR (300 MHz, CDCl_3_) δ (ppm): 7.95 (m, 2H), 7.44 (m, 2H), 5.11 (s, 2H), 0.98 (d, *J* = 16.3 Hz, 9H), 0.19 (s, 6H). The NMR data are consistent with the literature.^96^ 
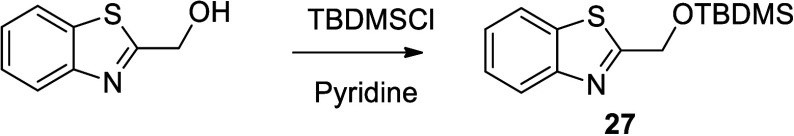


#### *tert*-Butyl­(benzo­[*d*]­thiazol-2-ylmethyl)­carbamate (**28**)

2-(Azidomethyl)­benzo­[*d*]­thiazole^91^ (1.1 g; 5.8 mmol) was dissolved in methanol (55 mL). Palladium on carbon (0.4 g; 4 mol %) was added to the reaction mixture under stirring. The reaction was stirred overnight under a positive pressure of hydrogen. The reaction was filtered on a Celite pad, and the solvent was removed under reduced pressure to give the corresponding benzo­[*d*]­thiazol-2-ylmethanamine. Yield: 0.72 g (76%). Red oil. ^1^H NMR (300 MHz, D_2_O) δ (ppm): 7.99 (d, *J* = 8.2 Hz, 1H), 7.50 (dt, *J* = 23.1, 8.2 Hz, 2H), 4.62 (s, 2H). The NMR data are consistent with those reported in the literature.^92^

Benzo­[*d*]­thiazol-2-ylmethanamine (0.54 g; 2.2 mmol) was dissolved in tetrahydrofuran (10 mL). Di-*tert*-butyl decarbonate (Boc)_2_O (0.5 g; 2.5 mmol) was added, and the reaction mixture was heated at 40 °C (heating mantle) for 1 h. After cooling, the solvent was removed under reduced pressure, and the crude compound was dissolved in dichloromethane (10 mL) and extracted with water (30 mL). Removal of the solvent under reduced pressure afforded the title compound. Yield: 0.8 g (69%). Red oil. The compound was used in the next step without further purification. ^1^H NMR (300 MHz, CDCl_3_) δ (ppm): 8.01 (d, *J* = 8.1 Hz, 1H), 7.89 (d, *J* = 8.1 Hz, 1H), 7.46 (m, 2H), 4.77 (d, *J* = 5.2 Hz, 2H), 1.53 (s, 9H). The NMR data are consistent with the literature.^97^ 
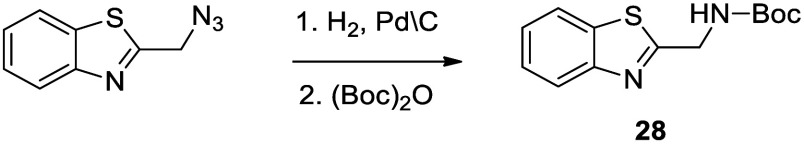


#### 2-(But-3-yn-1-yl)­benzo­[*d*]­thiazole (**29**)

Pent-4-ynoic acid (1.0 g; 10.2 mmol) was added to dichloromethane (30 mL). After the addition of dimethylformamide (0.05 mL), oxalyl chloride (2.5 g; 19.8 mmol) was added dropwise. The reaction mixture was stirred at room temperature for 1.5 h. The solvent was removed under reduced pressure to afford yellowish oily crystals. This material was added dropwise to a solution of 2-aminothiophenol (2.3 g; 18.4 mmol) in toluene (25 mL). After stirring the reaction at room temperature for 3 h, a saturated solution of NaHCO_3_ (30 mL) was added. The title product was extracted with ethyl acetate (10 mL), and after the solvent was removed under reduced pressure, it was purified using column chromatography (silica gel; *n*-hexane/ethyl acetate, 10:1). Yield: 0.86 g (25%). Pale yellow oil. ^1^H NMR (300 MHz, CDCl_3_) δ (ppm): 8.02 (d, *J* = 8.2 Hz, 1H), 7.88 (d, *J* = 8.2 Hz, 1H), 7.54–7.46 (m, 1H), 7.40 (m, 1H), 3.38 (m, 2H), 2.81 (m, 2H), 2.06 (t, *J* = 2.6 Hz, 1H). The NMR data are consistent with the literature.^98^ 
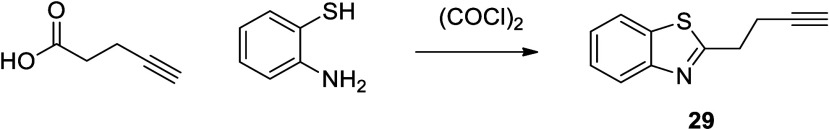


### General Procedure for the Synthesis of Quaternary Salts

The corresponding heterocycle (1 equiv) was dissolved in diethyl ether (10 mL). Methyl trifluoromethanesulfonate (1.5 equiv) was added dropwise under vigorous stirring, and the reaction was stirred overnight under the N_2_ atmosphere at room temperature. The resulting precipitate was filtered off and washed several times with diethyl ether and *n*-pentane.
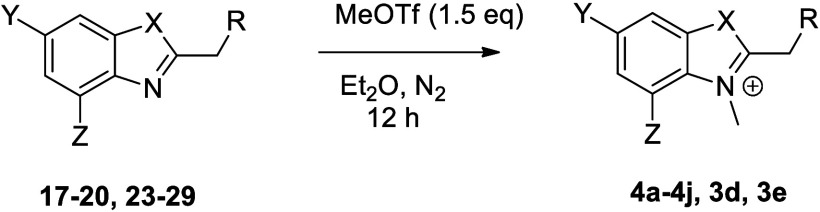


#### 2-(Methoxymethyl)-3-methylbenzo­[*d*]­thiazol-3-ium Triflate (**4a**)

Prepared according to the general procedure above from **17** (0.5 g; 2.8 mmol) and methyl trifluoromethanesulfonate (0.69 g; 4.2 mmol). Yield: 0.76 g (80%). White solid. ^1^H NMR (500 MHz, *d*_6_-DMSO) δ (ppm): 8.47 (d, *J* = 8.1 Hz, 1H), 8.34 (d, *J* = 8.1 Hz, 1H), 7.94 (t, *J* = 7.4 Hz, 1H), 7.84 (t, *J* = 7.4 Hz, 1H), 5.29 (s, 2H), 4.17 (s, 3H), 3.63 (s, 3H). ^13^C­{^1^H} NMR (126 MHz *d*_6_-DMSO) δ (ppm): 180.5, 142.7, 129.9, 129.5, 128.5, 125.2, 117.0, 69.2, 60.4, 37.0 (Figures S12 and S13). HRMS (ESI-TOF) *m*/*z*: [M – OTf ^–^]^+^ calcd for C_10_H_12_NOS^+^ 194.0634; found 194.0636. The NMR data are consistent with the literature.^89^ 
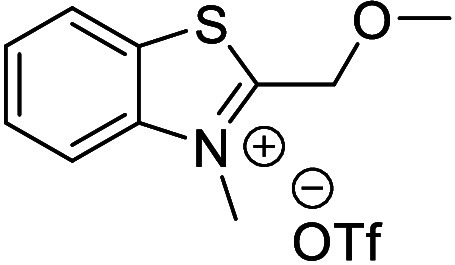


#### 2-(Chloromethyl)-3-methylbenzo­[*d*]­thiazol-3-ium Triflate (**4b**)

Prepared according to the general procedure above from **18** (0.87g; 4.7 mmol) and methyl trifluoromethanesulfonate (1.17 g; 7.1 mmol). Yield: 1.32 g (80%). White solid. ^1^H NMR (500 MHz, *d*_4_-CD_3_OD) δ (ppm): 8.28 (d, *J* = 8.6 Hz, 1H), 8.20 (d, *J* = 8.6 Hz, 1H), 7.89 (t, *J* = 7.8 Hz, 1H), 7.79 (t, *J* = 7.8 Hz, 1H), 5.48 (m, 2H), 4.26 (s, 3H). ^13^C­{^1^H} NMR (126 MHz, *d*_4_-CD_3_OD) δ (ppm): 142.7, 130.2, 129.7, 129.0, 124.2, 121.7, 119.1 116.6, 35.9 (Figures S14 and S15). HRMS (ESI-TOF) *m*/*z*: [M – OTf ^–^]^+^ calcd for C_9_H_9_ClNS^+^ 198.0139; found 198.0140. The NMR data are consistent with the literature.^89^ 
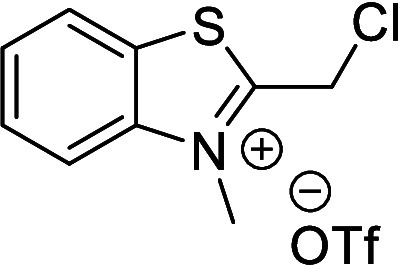


#### 2-Benzyl-3-methylbenzo­[*d*]­thiazol-3-ium Triflate (**4d**)

Prepared according to the general procedure above from **23** (0.45 g; 2.0 mmol) and methyl trifluoromethanesulfonate (0.49 g; 3.0 mmol). White solid. Yield: 0.65 g (83%). ^1^H NMR (300 MHz, *d*_6_-DMSO) δ (ppm): 8.33 (m, 2H), 7.91 (s, 1H), 7.79 (s, 1H), 7.52 (m, 5H), 4.87 (s, 2H), 4.31 (s, 3H). The NMR data are consistent with the literature.^36^ 
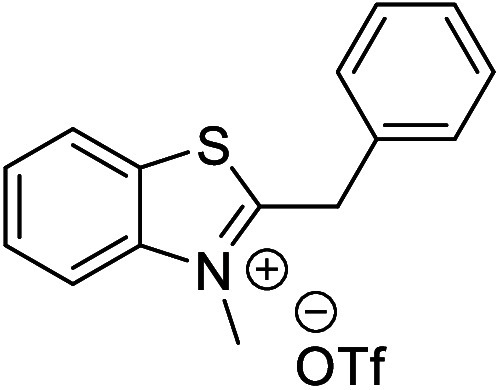


#### 3-Methyl-2-((methylthio)­methyl)­benzo­[*d*]­thiazol-3-ium Triflate (**4f**)

Prepared according to the general procedure above from **24** (0.18 g; 0.92 mmol) and methyl trifluoromethanesulfonate (0.23 g; 1.4 mmol). Yield: 0.13 g (40%). White solid. ^1^H NMR (300 MHz, *d*_4_-CD_3_OD) δ (ppm): 8.34 (d, *J* = 7.7 Hz, 1H), 8.26 (d, *J* = 7.7 Hz, 1H), 7.99–7.83 (m, 2H), 4.31 (s, 3H), 3.11 (s, 2H), 2.38 (s, 3H). The compound was used in the next step without further purification. The NMR data are consistent with the literature.^89^ 
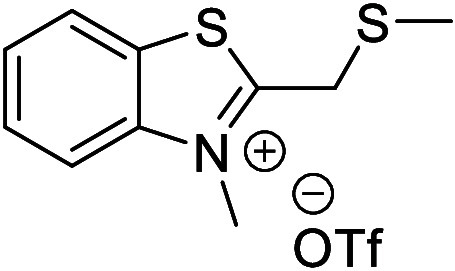


#### 2-(Acetoxymethyl)-3-methylbenzo­[*d*]­thiazol-3-ium Triflate (**4e**)

Prepared according to the general procedure above from **25** (0.28 g; 1.35 mmol) and methyl trifluoromethanesulfonate (0.33 g; 2.0 mmol). Yield: 0.32 g (64%). Beige solid. ^1^H NMR (300 MHz, *d*_6_-DMSO) δ (ppm): 8.42 (m, 2H), 7.91 (m, 2H), 5.91 (s, 2H), 4.27 (s, 3H), 2.24 (s, 3H). ^13^C­{^1^H} NMR (126 MHz, *d*_6_-DMSO) δ (ppm): 185.9, 172.4, 143.2, 129.8, 129.4, 128.2, 125.2, 116.8, 60.1, 36.7, 21.5 (Figures S16 and S17). HRMS (ESI-TOF) *m*/*z*: [M – OTf ^–^]^+^ calcd for C_11_H_12_NO_2_S^+^ 222.0583; found 222.0583.
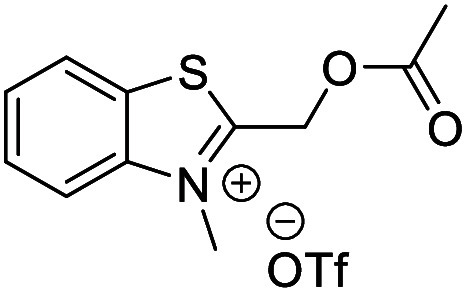


#### 2-(Acetamidomethyl)-3-methylbenzo­[*d*]­thiazol-3-ium Triflate (**4g**)

Prepared according to the general procedure above from **26** (0.26 g; 1.26 mmol) and methyl trifluoromethanesulfonate (0.31 g; 1.9 mmol). Yield: 0.32 g (68%). Brown solid. ^1^H NMR (300 MHz, *d*_6_-DMSO) δ (ppm): 9.18 (s, 1H), 8.42 (d, *J* = 8.1 Hz, 1H), 8.31 (d, *J* = 8.1 Hz, 1H), 7.92 (m, 1H), 7.83 (m, 1H), 5.09 (m, 2H), 4.31 (m, 3H), 2.04 (s, 3H). ^13^C­{^1^H} NMR (126 MHz, *d*_6_-DMSO) δ (ppm): 181.6, 171.2, 143.0, 130.0, 129.0, 128.7, 125.4, 125.1, 117.3, 37.1, 22.6 (Figures S18 and S19). HRMS (ESI-TOF) *m*/*z*: [M – OTf ^–^]^+^ calcd for C_11_H_13_N_2_OS^+^ 221.0743; found 221.0744.
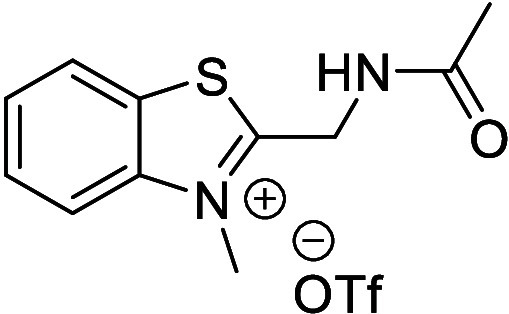


#### 2-(((*tert*-Butyldimethylsilyl)­oxy)­methyl)-3-methylbenzo­[*d*]­thiazol-3-ium Triflate (**4h**)

Prepared according to the general procedure above from **27** (0.20 g; 0.7 mmol) and methyl trifluoromethanesulfonate (0.17 g; 1.0 mmol). Yield: 0.24 g (75%). White solid. ^1^H NMR (300 MHz, *d*_4_-CD_3_OD) δ (ppm): 8.36 (d, *J* = 8.2 Hz, 1H), 8.25 (d, *J* = 8.2 Hz, 1H), 7.95 (t, *J* = 7.9 Hz, 1H), 7.84 (t, *J* = 7.9 Hz, 1H), 5.53 (s, 2H), 4.22 (s, 3H), 1.08 (s, 9H), 0.31 (s, 6H). ^13^C­{^1^H} NMR (126 MHz, *d*_4_-CD_3_OD) δ (ppm): 184.1, 143.0, 129.5, 128.0, 124.1, 124.0, 115.8, 61.4, 59.6, 35.5, 24.7, 17.7, −6.9 (Figures S20 and S21). HRMS (ESI-TOF) *m*/*z*: [M – OTf ^–^]^+^ calcd for C_15_H_24_NOSSi^+^ 294.1342; found 294.1341.
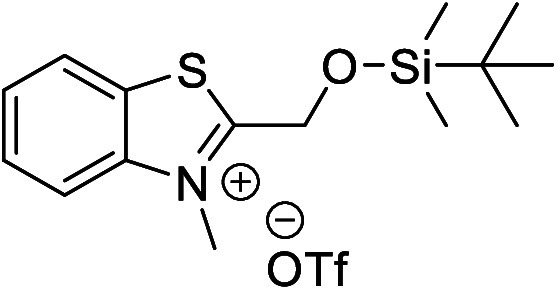


#### 2-(((*tert*-Butoxycarbonyl)­amino)­methyl)-3-methylbenzo­[*d*]­thiazol-3-ium Triflate (**4i**)

Prepared according to the general procedure above from **28** (0.37 g; 1.4 mmol) and methyl trifluoromethanesulfonate (0.34 g; 2.1 mmol). Yield: 0.51 g (85%). White solid. ^1^H NMR (300 MHz, *d*_6_-DMSO) δ (ppm): (d, *J* = 34.1 Hz, 2H), 7.87 (d, *J* = 24.3 Hz, 2H), 4.99 (s, 2H), 4.24 (s, 3H), 1.45 (s, 9H). ^13^C {^1^H} NMR (126 MHz, *d*_6_-DMSO) δ (ppm): 182.9, 143.2, 129.5, 128.9, 128.7, 126.3, 125.9, 125.1, 122.7, 117.3, 80.4, 37.1, 31.7, 28.5 (Figures S22 and S23). HRMS (ESI-TOF) *m*/*z*: [M – OTf ^–^]^+^ calcd for C_14_H_19_N_2_O_2_S^+^ 202.0685; found 202.0683.
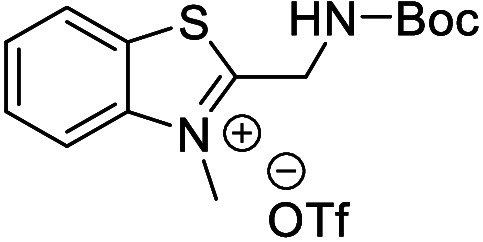


#### 2-(But-3-yn-1-yl)-3-methylbenzo­[*d*]­thiazol-3-ium Triflate (**4j**)

Prepared according to the general procedure above from **29** (0.23 g; 1.3 mmol) and methyl trifluoromethanesulfonate (0.30 g; 1.8 mmol). Yield: 0.2 g (46%). Pink oil that crystallizes on standing. ^1^H NMR (300 MHz, *d*_4_-CD_3_OD) δ (ppm): 8.35 (d, *J* = 8.1 Hz, 1H), 8.28 (d, *J* = 8.1 Hz, 1H), 7.97 (t, *J* = 7.8 Hz, 1H), 7.87 (t, *J* = 7.8 Hz, 1H), 4.36 (s, 3H), 3.80 (t, *J* = 6.7 Hz, 2H), 2.98 (t, *J* = 6.7 Hz, 2H), 2.63 (s, 1H) ^13^C­{^1^H} NMR (126 MHz, *d*_4_-CD_3_OD) δ (ppm): 129.7, 129.1, 128.5, 127.0, 123.8, 118.7, 116.5, 79.7, 72.2, 35.8, 29.5, 16.6 (Figures S24 and S25). HRMS (ESI-TOF) *m*/*z*: [M – OTf ^–^]^+^ calcd for C_12_H_12_NS^+^ 279.1162; found 279.1165.
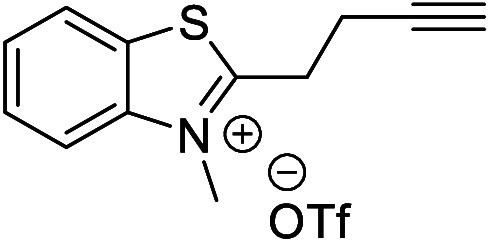


#### 5-Methoxy-1,2,3,3-tetramethyl-3*H*-indol-1-ium Triflate (**3d**)

Prepared according to the general procedure above from **19** (0.47 g; 2.5 mmol) and methyl trifluoromethanesulfonate (0.61 g; 3.7 mmol). Yield: 0.53 g (60%). White solid. ^1^H NMR (300 MHz, *d*_4_-CD_3_OD) δ (ppm): 7.73 (d, *J* = 8.5 Hz, 1H), 7.34 (s, 1H), 7.16 (d, *J* = 8.5 Hz, 1H), 4.01 (s, 3H), 3.92 (s, 3H), 2.75 (s, 3H), 1.59 (s, 6H) (Figure S9). The NMR data are consistent with the literature.^99^ 
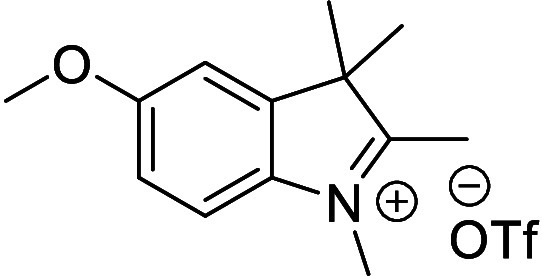


#### 1,2,3,3,5,7-Hexamethyl-3*H*-indol-1-ium Triflate (**3e**)

Prepared according to the general procedure above from **20** (1.45 g; 7.7 mmol) and methyl trifluoromethanesulfonate (1.90 g; 11.6 mmol). Yield: 2.27 g (83%). White solid. ^1^H NMR (300 MHz, *d*_6_-DMSO) δ (ppm): 7.45 (s, 1H), 7.19 (s, 1H), 4.08 (s, 3H), 2.70 (d, *J* = 4.0 Hz, 6H), 2.37 (s, 3H), 1.47 (s, 6H) (Figure S10). The NMR data are consistent with the literature.^84^ 
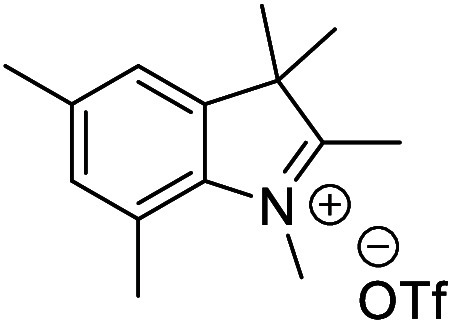


#### 5-(Dimethylamino)-1,2,3,3-tetramethyl-3*H*-indol-1-ium Iodide (**3f**)

**21** (1.0 g, 5.7 mmol) was dissolved in acetonitrile (20 mL), and methyl iodine (12 mL, 0.17 mol) was added. The reaction was heated at 90 °C (heat mantle) in a pressure vial for 48 h. After cooling, the resulting precipitate was filtered off and washed several times with diethyl ether and *n*-pentane. Yield: 1.26 g (64%). White solid. ^1^H NMR (300 MHz, *d*_6_-DMSO) δ (ppm): 7.64 (d, *J* = 8.7 Hz, 1H), 7.10 (s, 1H), 6.81 (d, *J* = 8.7 Hz, 1H), 3.89 (s, 3H), 3.01 (s, 6H), 2.64 (s, 3H), 1.47 (s, 6H) (Figure S11). The NMR data are consistent with the literature.^86^ 
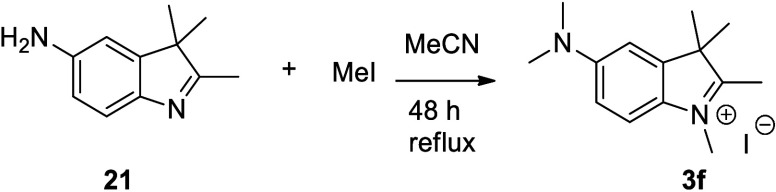


#### 5-Cyano-1,2,3,3-tetramethyl-3*H*-indol-1-ium Triflate (**3g**)

**22** (1.0 g, 5.4 mmol) was dissolved in acetonitrile (15 mL). Methyl trifluoromethanesulfonate (1.2 mL, 11.0 mmol) was added dropwise. The reaction was heated at 90 °C (heating mantle) in a pressure vial overnight. After cooling, solvent was removed under reduced pressure. Methanol (3 mL) was added to the residue, then diethyl ether (20 mL) was added dropwise. The flask was left at – 20 °C overnight. The precipitate was filtered and washed 3× with diethyl ether (5 mL). Yield: 1.60 g. (85%). Red solid which becomes honey tar on standing. ^1^H NMR (300 MHz, *d*_6_-DMSO) δ (ppm): 7.57 (s, 2H), 6.84 (s, 1H), 3.98 (s, 3H), 3.07 (m, 3H), 1.30 (s, 6H). The product was used in the next step without further purification.
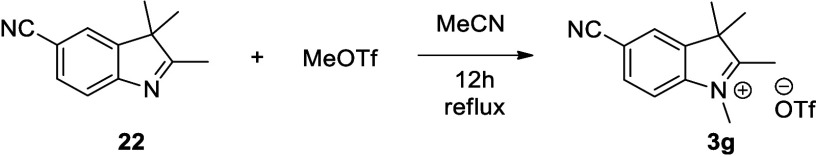


### General Procedure for the Synthesis of Fischer’s Base Derivatives

Nitrogen quaternary salt **3d**–**3g** (2.9 mmol) was suspended in dichloromethane (15 mL). 10% NaOH solution (20 mL) was added slowly under stirring. The mixture was allowed to stir at room temperature for 1 h. A saturated solution of NaHCO_3_ (30 mL) was added, and the mixture was extracted 2× with dichloromethane (10 mL). The solvent was removed under reduced pressure to afford a dark oil which was used directly in the next step.
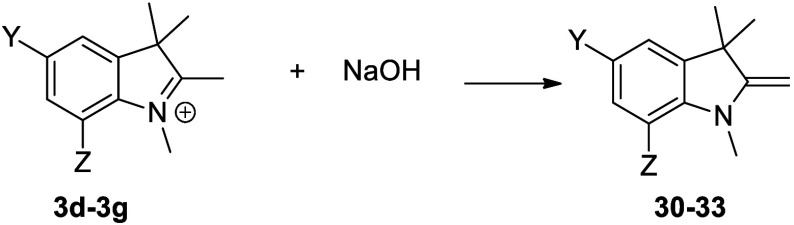


#### 5-Methoxy-1,3,3-trimethyl-2-methyleneindoline (**30**)

Prepared according to the general procedure above from **3d** (0.4 g; 1.3 mmol). Yield: 0.2 g (95%). Dark oil. ^1^H NMR (300 MHz, CDCl_3_) δ (ppm): 6.73 (m, 2H), 6.44 (d, *J* = 8.4 Hz, 1H), 3.79 (s, 5H), 3.02 (s, 3H), 1.36 (s, 6H). The compound was used in the next step without further purification.
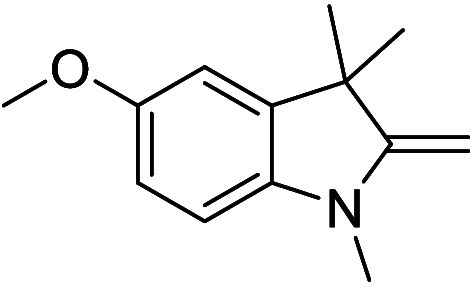


#### 1,3,3,5,7-Pentamethyl-2-methyleneindoline (**31**)

Prepared according to the general procedure above from **3e** (1.6 g; 4.6 mmol). Yield: 0.82 g (90%). Dark oil. The compound was used in the next step without further purification.
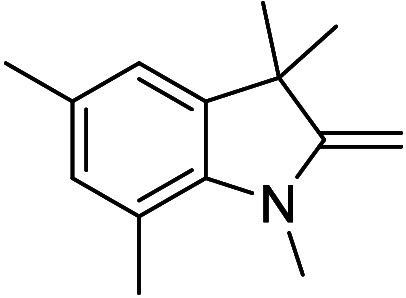


#### *N,N*,1,3,3-Pentamethyl-2-methyleneindolin-5-amine (**32**)

Prepared according to the general procedure above from **3f** (50 mg; 0.14 mmol). Yield: 30 mg (95%). Dark oil. ^1^H NMR (300 MHz, *d*_6_-DMSO) δ (ppm): 6.76 (s, 1H), 6.51 (s, 2H), 3.73 (d, *J* = 7.3 Hz, 2H), 2.92 (d, *J* = 7.3 Hz, 3H), 2.75 (d, *J* = 10.0 Hz, 6H), 1.25 (d, *J* = 10.0 Hz, 6H). The compound was used in the next step without further purification.
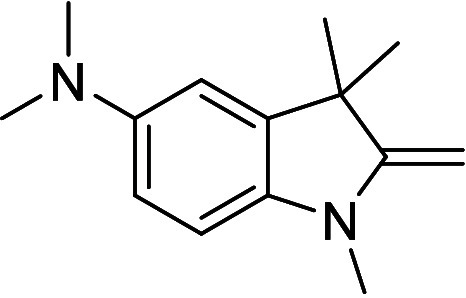


#### 1,3,3-Trimethyl-2-methyleneindoline-5-carbonitrile (**33**)

Prepared according to the general procedure above from **3g** (2.4 g; 6.9 mmol). Yield: 1.0 g (73%). Dark oil. The compound was used in the next step without further purification.
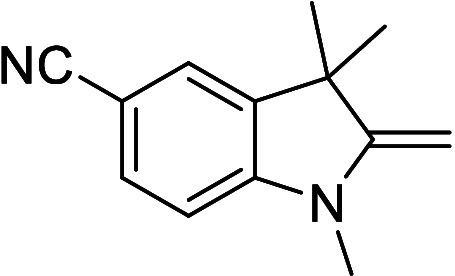


### General Procedure for the Cyanation of Fischer’s Base Derivatives^18^

Sodium thiocyanate (0.40 g; 4.9 mmol) was dissolved in acetone (6 mL). Benzoyl chloride (0.83 g; 5.9 mmol) was added dropwise under vigorous stirring and a white precipitate was formed. The reaction was refluxed (heating mantle) for 30 min. After cooling, Fischer’s base derivative **30**–**33** (0.8 g, 4.0 mmol) was added dropwise. After the addition, the reaction mixture turned deep red. After refluxing for 30 min and subsequent cooling, the reaction mixture was poured in water (∼25 mL) and extracted 3× with dichloromethane (10 mL). The solvent was removed under reduced pressure, and the crude product was dissolved in a 3% sodium methoxide solution (10 mL) and refluxed for 2 h. After cooling, the solvent was removed under reduced pressure, and the product was purified using column chromatography (silica gel; *n*-hexane/ethyl acetate, 3:1).
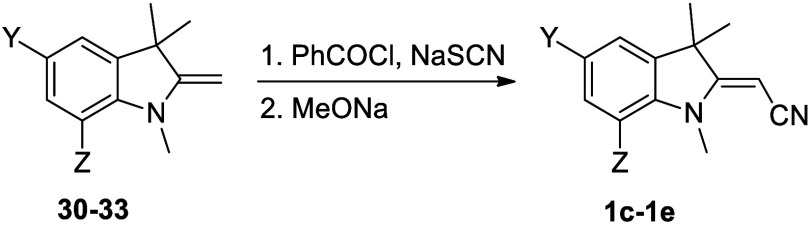


#### (*Z*)-2-(5-Methoxy-1,3,3-trimethylindolin-2-ylidene)­acetonitrile (**1c**)

Prepared according to the general procedure above from **30** (0.4 g; 2.0 mmol). Yield: 0.2 g (45%). Beige solid. The title compound was obtained as a 2:1 mixture of *E*/*Z* isomers, which were directly used in the next step. ^1^H NMR (300 MHz, *d*_6_-DMSO) δ (ppm): 7.08–6.74 (m, 5H), 4.36 (s, 1H), 4.33 (s, 0.5H), 3.72 (s, 5H), 3.52 (s, 2H), 3.10 (s, 3H), 1.55 (s, 6H), 1.29 (s, 4H). ^13^C­{^1^H} NMR (126, MHz, *d*_6_-DMSO) δ (ppm): 172.7, 171.8, 155.7, 155.6, 139.1, 138.9, 138.8, 137.9, 121.2, 112.8, 112.7, 110.0, 109.8 108.3, 108.2, 56.8, 56.1, 54.8, 47.8, 47.2, 31.1, 29.6, 28.6, 25.8 (Figures S1 and S2). HRMS (APCI-TOF) *m*/*z*: [M + H]^+^ calcd for C_14_H_16_N_2_O 229.1335; found 229.1333.
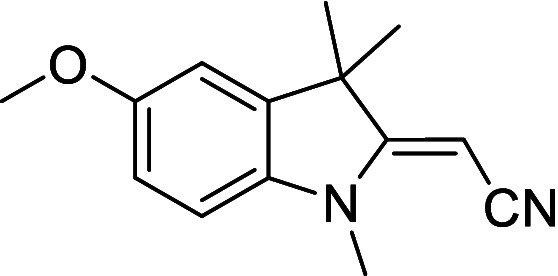


#### (*Z*)-2-(1,3,3,5,7-Pentamethylindolin-2-ylidene)­acetonitrile (**1d**)

Prepared according to the general procedure above from **31** (0.8 g; 4.0 mmol). Yield: 0.8 g (90%). Dark oil that solidifies on standing. ^1^H NMR (300 MHz, CDCl_3_) δ (ppm): 6.84 (s, 1H), 6.76 (s, 1H), 4.06 (s, 1H), 3.38 (s, 3H), 2.54 (d, *J* = 9.8 Hz, 3H), 2.29 (s, 3H), 1.65 (s, 6H). ^13^C­{^1^H} NMR (126 MHz, CDCl_3_) δ (ppm): 173.9, 139.5, 138.89, 132.5, 132.4, 131.4, 120.7, 117.9, 57.6, 46.5, 32.9, 29.2, 26.2, 20.6, 19.6 (Figures S3 and S4). HRMS (APCI-TOF) *m*/*z*: [M + H]^+^ calcd for C_15_H_18_N_2_ 227.1543; found 227.1540.
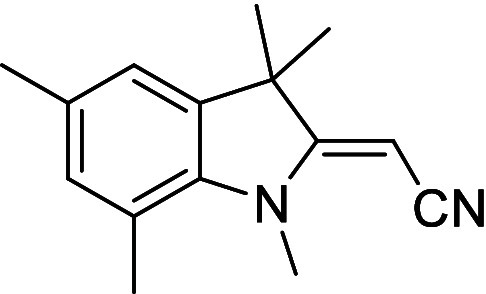


#### (*Z*)-2-(5-(Dimethylamino)-1,3,3-trimethylindolin-2-ylidene)­acetonitrile (**1e**)

Prepared according to the general procedure above from **32** (0.4 g; 1.8 mmol). Yield: 50 mg (11%). Brown solid. ^1^H NMR (300 MHz, *d*_6_-DMSO) δ (ppm): 6.86 (d, *J* = 2.2 Hz, 1H), 6.78 (d, *J* = 8.6 Hz, 1H), 6.57 (dd, *J* = 8.6, 2.2 Hz, 1H), 4.28 (s, 1H), 3.08 (s, 3H), 2.83 (s, 6H), 1.55 (s, 6H). ^13^C­{^1^H} NMR (126, MHz, *d*_6_-DMSO) δ (ppm): 172.5, 147.5, 138.7, 135.4, 121.5, 112.2, 108.9, 108.2, 60.2, 55.8, 47.4, 41.7, 29.6, 28.8, 26.0 (Figures S5 and S6). HRMS (APCI-TOF) *m*/*z*: [M + H]^+^ calcd for C_15_H_19_N_3_ 242.1652; found 242.1654.
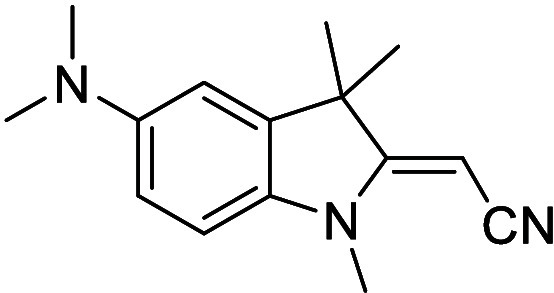


#### (*Z*)-2-(Cyanomethylene)-1,3,3-trimethylindoline-5-carbonitrile (**1f**)

Prepared according to the general procedure above from **33** (0.8 g; 4.0 mmol). Yield: 0.34 g (42%). Dark solid. The title compound was obtained as a 1:1 mixture of *E*/*Z* isomers, which were directly used in the next step. ^1^H NMR (500 MHz, *d*_6_-DMSO) δ (ppm): 7.83 (dd, *J* = 7.5, 1.4 Hz, 2H), 7.72 (dd, *J* = 7.5, 1.4 Hz, 2H), 7.15 (d, *J* = 8.3 Hz, 1H), 7.11 (d, *J* = 8.3 Hz, 1H), 4.74 (s, 1H), 4.69 (s, 1H), 3.60 (s, 3H), 3.19 (s, 3H), 1.59 (s, 6H), 1.34 (s, 6H). ^13^C­{^1^H} NMR (126, MHz, *d*_6_-DMSO) δ (ppm): 172.1, 171.0, 148.9, 148.0, 138.9, 138.6, 134.1, 134.0, 126.2, 126.0, 120.1, 120.0, 119.9, 119.7, 108.9, 108.8, 103.4, 103.2, 61.7, 59.6, 47.2, 46.6, 31.3, 29.8, 28.3, 25.6 (Figures S7 and S8). HRMS (APCI-TOF) *m*/*z*: [M + H]^+^ calcd for C_14_H_13_N_3_ 224.1182; found 224.1184.
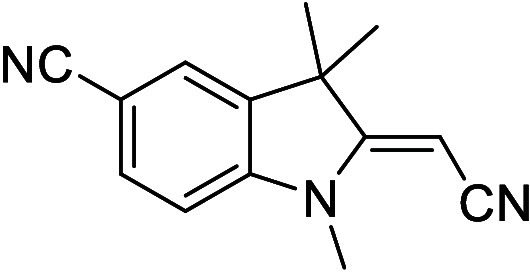


### Synthesis of Pentamethine Cyanines (Cy5)

#### General Procedure for the Synthesis of Pentamethine Cyanine Dyes from Fischer’s Bases

Aldehyde **7** (1 equiv; 0.39 mM) was dissolved in acetonitrile (4 mL). After 10 min, POCl_3_ (44 μL; 0.47 mM, 1.2 equiv) was added dropwise under stirring. After 15 min, a heterocycle (**1a**, **1b**, **2a**, or **2b**; 1–3 equiv) was added to the solution. The reaction mixture was stirred at room temperature or under reflux (heating mantle) until completion (monitored by HPLC). The solvent was removed under reduced pressure, and the crude product was dissolved in dichloromethane (∼20 mL) and extracted several times with an HCl aqueous solution (pH = 4). The solvent was removed, and the pure compound was obtained using chromatography on silica gel (*n*-hexane/ethyl acetate, 1:1, then dichloromethane/methanol, 100:0 to 96:4). The product was obtained as a dark solid.
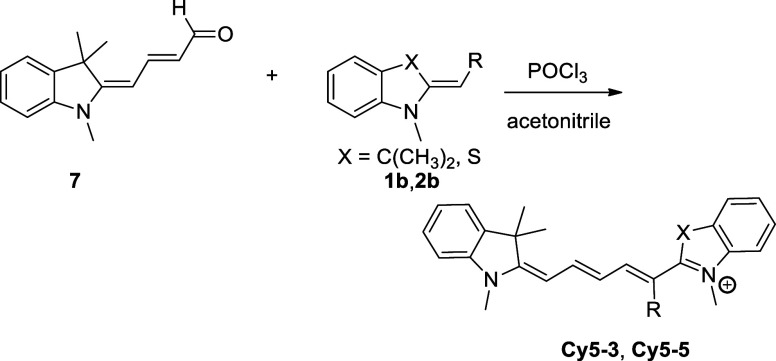


#### 1,3,3-Trimethyl-2-((3*Z*,5*E*)-2-oxo-7-((*E*)-1,3,3-trimethylindolin-2-ylidene)­hepta-3,5-dien-3-yl)-3*H*-indol-1-ium Chloride (**Cy5–3**)

Prepared according to the general procedure above from **1b** (0.19 g; 0.88 mmol) and **7** (0.10 g; 0.44 mmol). The reaction was stirred at reflux (heating mantle) for 2 h. Yield: 90 mg (45%). Blue solid. Mp: 143–148 °C. ^1^H NMR (500 MHz, *d*_4_-CD_3_OD) δ (ppm): 8.07 (d, *J* = 12.8 Hz, 1H), 7.88 (t, *J* = 12.8 Hz, 1H), 7.79 (dd, *J* = 6.0, 3.0 Hz, 1H), 7.67 (dd, *J* = 6.0, 3.0 Hz, 1H), 7.59 (dd, *J* = 6.0, 3.0 Hz, 2H), 7.26 (d, *J* = 7.4 Hz, 1H), 7.19 (t, *J* = 7.2 Hz, 1H), 6.99 (t, *J* = 7.2 Hz, 1H), 6.94 (d, *J* = 7.4 Hz, 1H), 5.78 (m, 2H), 3.83 (s, 3H), 3.24 (s, 3H), 2.44 (s, 3H), 1.52 (d, *J* = 30.4 Hz, 12H). ^13^C­{^1^H} NMR (126 MHz, *d*_4_-CD_3_OD) δ (ppm): 195.0, 193.1, 169.6, 151.4, 151.2, 143.4, 142.9, 141.6, 139.9, 129.9, 129.0, 127.9, 122.8, 121.5, 116.3, 115.2, 113.8, 108.6, 98.6, 56.6, 34.8, 29.1, 26.9, 24.8, 23.0 (Figures S32–S34). HRMS (ESI-TOF) *m*/*z*: [M – Cl ^–^]^+^ calcd for C_29_H_33_N_2_O^+^ 425.2587; found 425.2584 (Figure S101).
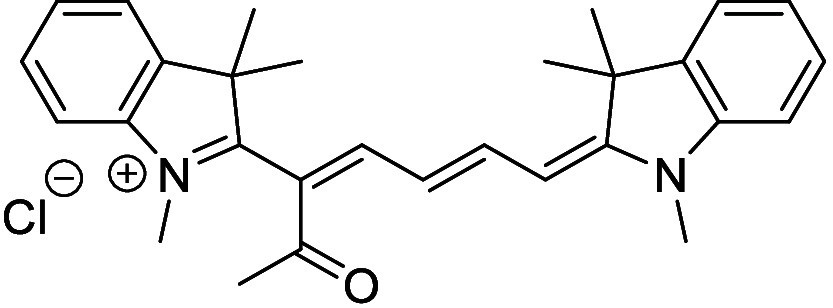


#### 2-((2*E*,4*E*)-1-Ethoxy-1-oxo-6-((*E*)-1,3,3-trimethylindolin-2-ylidene)­hexa-2,4-dien-2-yl)-3-methylbenzo­[*d*]­thiazol-3-ium Chloride (**Cy5–5**)

Prepared according to the general procedure above from **7** (0.05 g; 0.22 mmol) and **2b** (0.05 g; 0.21 mmol). The reaction was stirred at room temperature for 30 min. Yield: 70 mg (66%). Blue solid. Mp: 153–158 °C. ^1^H NMR (500 MHz, *d*_3_-CD_3_CN) δ (ppm): 8.17 (d, *J* = 7.8 Hz, 1H), 8.09–7.98 (m, 3H), 7.85 (t, *J* = 7.8 Hz, 1H), 7.74 (t, *J* = 7.7 Hz, 1H), 7.45 (d, *J* = 7.8 Hz, 1H), 7.39 (t, *J* = 7.7 Hz, 1H), 7.21 (t, *J* = 7.7 Hz, 1H), 7.16 (d, *J* = 7.8 Hz, 1H), 6.58 (t, *J* = 12.9 Hz, 1H), 6.06 (d, *J* = 12.9 Hz, 1H), 4.34 (q, *J* = 7.1 Hz, 2H), 4.02 (s, 3H), 3.45 (s, 3H), 1.67 (s, 6H), 1.37 (t, *J* = 7.1 Hz, 3H). ^13^C­{^1^H} NMR (126 MHz, *d*_3_-CD_3_CN) δ (ppm): 172.9, 172.0, 165.1, 154.9, 153.5, 143.9, 142.6, 141.3, 130.1, 129.6, 128.9, 128.2, 124.7, 123.8, 122.6, 118.8, 116.7, 110.6, 101.3, 61.7, 49.1, 39.6, 31.1, 27.4, 14.3 (Figures S35–S38). HRMS (ESI-TOF) *m*/*z*: [M – OTf ^–^]^+^ calcd for C_27_H_29_N_2_O_2_S^+^ 445.1944; found 445.1947 (Figure S102).
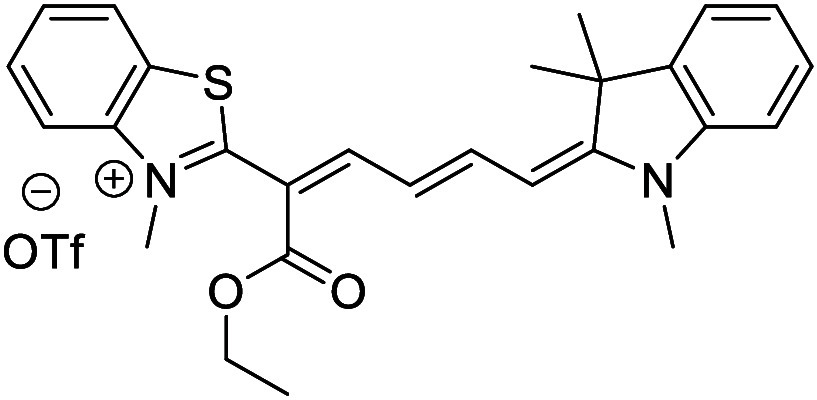


#### General Procedure for the Synthesis of Pentamethine Cyanine Dyes from Quaternary Salts

An aldehyde derivative (**7**, **13**, or **16**; 1 equiv; 0.39 mM) was dissolved in acetonitrile (5 mL). After 15 min, POCl_3_ (44 μL; 0.47 mM, 1.2 equiv) was added dropwise under stirring. After 10 min, the corresponding quaternary salt (**3c**, **4a**, or **4b**; 1.1–3 equiv) was added to the solution. A solution of *N*, *N*-diisopropylethylamine (DIPEA; 0.24 mL; 1.38 mM, 3.5 equiv) in acetonitrile (3 mL) was added dropwise over 20 min using a syringe pump. The solvent was removed under reduced pressure, and the crude material was dissolved in dichloromethane (∼20 mL) and extracted several times with an HCl aqueous solution (pH = 4). The solvent was evaporated under reduced pressure, and the pure product was obtained by chromatography on silica gel (*n*-hexane/ethyl acetate, 1:1, then dichloromethane/methanol, 100:0 to 96:4). The product was obtained as a dark solid.
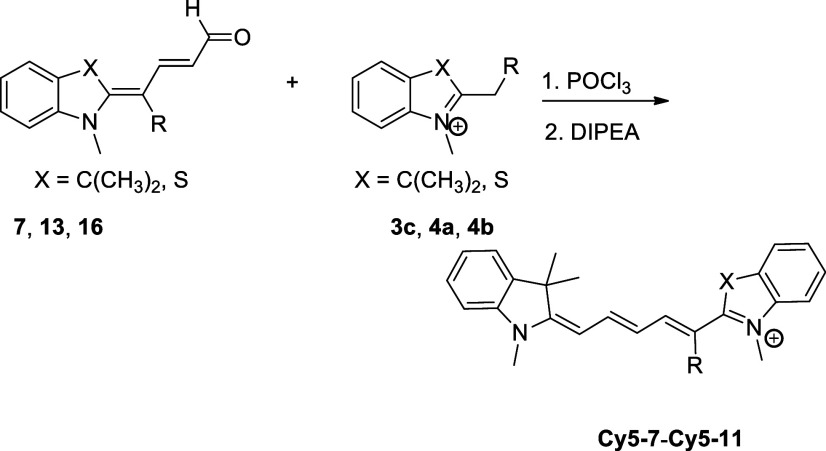


#### 2-((1*Z*,3*E*)-1-Methoxy-5-((*E*)-1,3,3-trimethylindolin-2-ylidene)­penta-1,3-dien-1-yl)-3-methylbenzo­[*d*]­thiazol-3-ium Triflate (**Cy5–7**)

Prepared according to the general procedure above from **7** (0.085 g; 0.37 mmol) and **4a** (0.21 g; 0.61 mmol). Yield: 170 mg (82%). Blue solid. Mp: 143–146 °C. ^1^H NMR (500 MHz, *d*_6_-DMSO) δ (ppm): 8.19 (d, *J* = 7.9 Hz, 1H), 8.03 (m, 2H), 7.80 (d, *J* = 7.9 Hz, 1H), 7.74 (t, *J* = 7.8 Hz, 1H), 7.60 (t, *J* = 7.8 Hz, 1H), 7.48 (d, *J* = 7.2 Hz, 1H), 7.32 (t, *J* = 7.8 Hz, 1H), 7.17 (d, *J* = 7.2 Hz, 1H), 7.09 (t, *J* = 7.8 Hz, 1H), 6.50 (t, *J* = 12.5 Hz, 1H), 6.09 (d, *J* = 12.5 Hz, 1H), 4.20 (s, 3H), 3.82 (s, 3H), 3.43 (s, 3H), 1.64 (s, 6H). ^13^C­{^1^H} NMR (126 MHz, *d*_6_-DMSO) δ (ppm): 168.2, 166.0, 165.6, 148.5, 138.5, 136.2, 129.1, 128.8, 128.4, 127.3, 126.7, 124.0, 123.1, 122.5, 117.6, 115.6, 109.7, 101.0, 91.6, 61.4, 47.8, 38.2, 28.0 (Figures S39–S42). HRMS (ESI-TOF) *m*/*z*: [M – OTf ^–^]^+^ calcd for C_23_H_27_N_2_OS^+^ 403.1839; found 403.1838 (Figure S103).
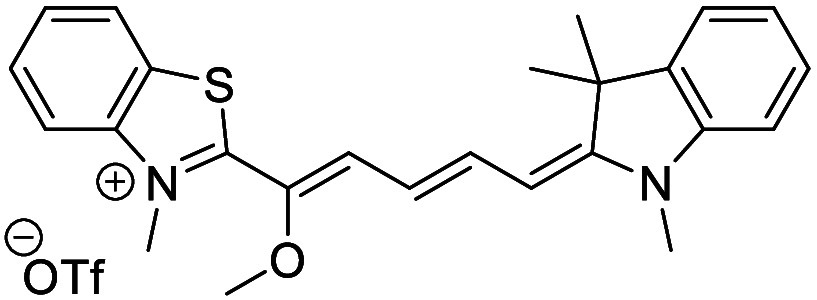


#### 2-((1*Z*,3*E*)-1-Chloro-5-((*E*)-1,3,3-trimethylindolin-2-ylidene)­penta-1,3-dien-1-yl)-3-methylbenzo­[*d*]­thiazol-3-ium Triflate (**Cy5–8**)

Prepared according to the general procedure above from **7** (0.085 g; 0.37 mmol) and **4b** (0.21 g; 0.60 mmol). Yield: 65 mg (33%). Blue solid. Mp: 118–121 °C. ^1^H NMR (300 MHz, *d*_6_-DMSO) δ (ppm): 8.32–8.19 (m, 2H), 8.14 (d, *J* = 7.8 Hz, 1H), 7.99 (d, *J* = 7.8 Hz, 1H), 7.72 (t, *J* = 7.8 Hz, 1H), 7.56 (m, 2H), 7.46–7.32 (m, 2H), 7.22 (m, 1H), 6.55 (t, *J* = 12.0 Hz, 1H), 6.39 (d, *J* = 12.0 Hz, 1H), 4.19 (s, 3H), 3.57 (s, 3H), 1.66 (s, 6H). ^13^C­{^1^H} NMR (126 MHz, *d*_6_-DMSO) δ (ppm): 172.6, 165.2, 153.2, 144.9, 144.9, 143.4, 141.3, 129.1, 128.8, 126.8, 126.4, 124.8, 123.6, 122.7, 122.5, 119.9 119.8, 115.2, 111.2, 104.4, 103.7, 49.0, 31.4, 27.6 (Figures S43–S46). HRMS (ESI-TOF) *m*/*z*: [M – OTf ^–^]^+^ calcd for C_24_H_24_ClN_2_S^+^ 407.1343; found 407.1341 (Figure S104).
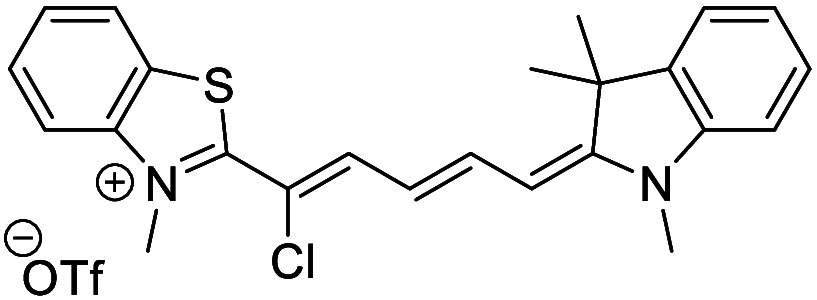


#### 1,3,3-Trimethyl-2-((2*E*,4*E*,6*Z*)-6-(3-methylbenzo­[d]­thiazol-2­(3*H*)-ylidene)­hexa-2,4-dien-2-yl)-3*H*-indol-1-ium Triflate (**Cy5–10**)

Prepared according to the general procedure above from **13** (0.015 g; 0.07 mmol) **3c** (0.09 g; 0.26 mmol). Yield: 13 mg (35%). Blue solid. Mp: 158–161 °C. ^1^H NMR (500 MHz, *d*_4_-CD_3_OD) δ (ppm): 7.83–7.68 (m, 3H), 7.61 (d, *J* = 8.3 Hz, 1H), 7.52 (t, *J* = 7.8 Hz, 1H), 7.37 (t, *J* = 7.8 Hz, 1H), 7.28 (m, 2H), 7.10 (t, *J* = 7.8 Hz, 1H), 7.06 (d, *J* = 7.8 Hz, 1H), 6.57 (d, *J* = 12.5 Hz, 1H), 6.51 (t, *J* = 12.5 Hz, 1H), 3.82 (s, 3H), 3.65 (s, 3H), 2.16 (s, 3H), 1.59 (s, 6H). ^13^C­{^1^H} NMR (126 MHz, *d*_4_-CD_3_OD) δ (ppm): 174.8, 166.7, 152.5, 152.1, 144.6, 142.1, 140.7, 128.1, 128.1, 125.9, 125.6, 124.1, 122.4, 121.5, 118.4, 113.3, 110.0, 108.1, 102.8, 49.9, 36.7, 32.8, 26.4, 14.1 (Figures S47–S50). HRMS (ESI-TOF) *m*/*z*: [M – OTf ^–^]^+^ calcd for C_25_H_27_N_2_S^+^ 387.1889; found 387.1889 (Figure S105).
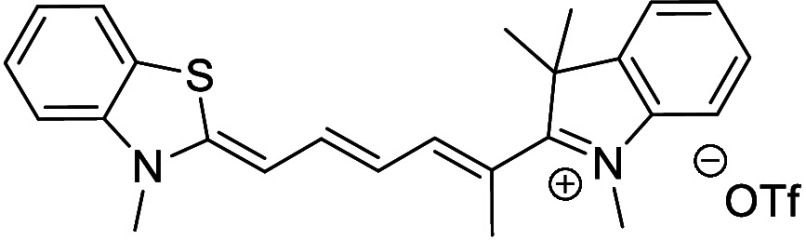


#### 2-((1*Z*,3*E*)-1-Cyano-5-((*E*)-1,3,3-trimethylindolin-2-ylidene)­hexa-1,3-dien-1-yl)-1,3,3-trimethyl-3*H*-indol-1-ium Triflate (**Cy5–11**)

Prepared according to the general procedure above from **16** (38 mg; 0.15 mmol) and **3c** (0.1 g; 0.29 mmol). Yield: 30 mg (35%). Blue solid. Mp: 136–138 °C. ^1^H NMR (500 MHz, *d*_6_-DMSO) δ (ppm): 7.86 (d, *J* = 8.0 Hz, 1H), 7.83–7.79 (m, 1H), 7.71–7.58 (m, 3H), 7.50 (d, *J* = 8.0 Hz, 1H), 7.43 (d, *J* = 8.0 Hz, 1H), 7.36 (t, *J* = 7.7 Hz, 1H), 7.24 (d, *J* = 7.7 Hz, 1H), 7.17 (t, *J* = 7.7 Hz, 1H), 6.62 (m, 1H), 4.01 (s, 3H), 3.74 (s, 3H), 2.27 (s, 3H), 1.66 (d, *J* = 6.2 Hz, 12H). ^13^C­{^1^H} NMR (126 MHz, *d*_6_-DMSO) δ (ppm): 189.8, 171.1, 146.4, 144.2, 142.6, 140.8, 139.3, 129.4, 128.7, 124.3, 123.2, 122.5, 122.4, 119.8, 118.8, 118.7, 117.8, 115.4, 110.3, 78.9, 54.3, 49.7, 37.9, 26.0, 24.4, 15.1 (Figures S51–S54). HRMS (ESI-TOF) *m*/*z*: [M – OTf ^–^]^+^ calcd for C_29_H_32_N_3_^+^ 422.2591; found 422.2587 (Figure S106).
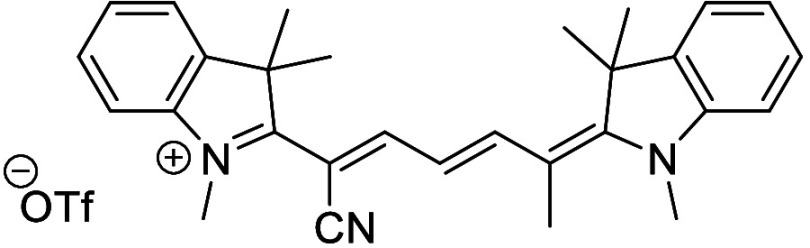


#### 2-((1*Z*,3*E*)-5-Cyano-1-methoxy-5-((*Z*)-1,3,3-trimethylindolin-2-ylidene)­penta-1,3-dien-1-yl)-3-methylbenzo­[*d*]­thiazol-3-ium Triflate (**Cy5–12**)

Prepared according to the general procedure above from **16** (40 mg; 0.16 mmol) and **4a** (0.1 g; 0.29 mmol). Yield: 30 mg (35%). Blue solid. Mp: 121–124 °C. ^1^H NMR (500 MHz, *d*_6_-DMSO) δ (ppm): 8.38 (d, *J* = 8.0 Hz, 1H), 8.28 (d, *J* = 8.0 Hz, 1H), 7.89 (dd, *J* = 10.5, 5.5 Hz, 2H), 7.77 (t, *J* = 7.6 Hz, 2H), 7.52 (d, *J* = 7.2 Hz, 1H), 7.37 (t, *J* = 7.5 Hz, 1H), 7.27 (d, *J* = 7.2 Hz, 1H), 7.19 (t, *J* = 7.5 Hz, 1H), 6.67 (m, 1H), 4.34 (s, 3H), 3.86 (s, 3H), 3.78 (s, 3H), 1.68 (s, 6H). ^13^C­{^1^H} NMR (126 MHz, *d*_6_-DMSO) δ (ppm): 171.2, 169.3, 144.0, 140.9, 139.6, 135.1, 129.8, 128.7, 128.6, 128.2, 124.6, 124.4, 122.5, 122.4, 119.8, 117.0, 115.9, 110.4, 79.4, 62.0, 49.9, 38.8 (Figures S55–S58). HRMS (ESI-TOF) *m*/*z*: [M – OTf ^–^]^+^ calcd for C_26_H_26_N_3_OS^+^ 428.1791; found 428.1788 (Figure S107).
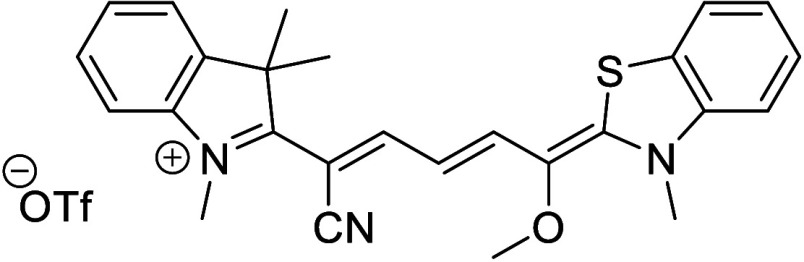


#### General Procedure for the Synthesis of Heptamethine Cyanine Dyes from Quaternary Salts

A, aldehyde derivative (**8** or **15**; 1 eq; 0.39 mM) was dissolved in acetonitrile (5 mL). After 15 min, POCl_3_ (44 μL; 0.47 mM, 1.2 equiv) was added dropwise under stirring. After 10 min, the corresponding quaternary salt (**3b**, **3c**, **4a**, **4d**–**4j**; 1.1–3 equiv) was added to the solution. A solution of DIPEA (0.24 mL; 1.38 mM, 3.5 equiv) in acetonitrile (3 mL) was added dropwise over 20 min (using syringe pump). Solvent was removed under reduced pressure, and the crude was dissolved in dichloromethane (∼20 mL) and extracted several times with an HCl aqueous solution (pH = 4). The solvent was evaporated, and the pure compound was obtained by chromatography on silica gel (*n*-hexane/ethyl acetate, 1:1, then dichloromethane/methanol, 100:0 to 96:4). The product was obtained as a dark solid.
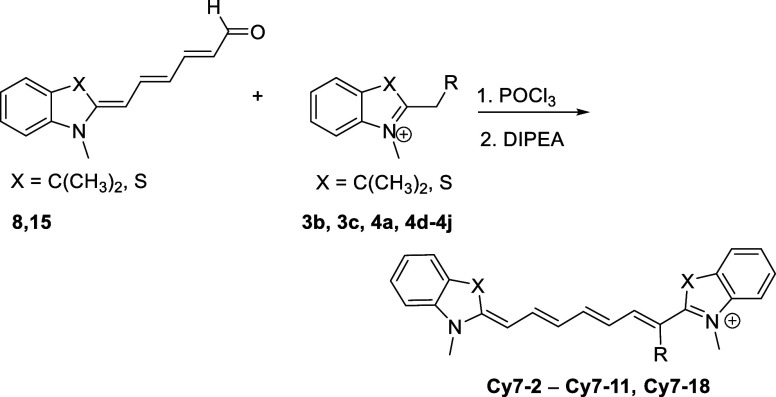


#### 1,3,3-Trimethyl-2-((1*E*,3*E*,5*E*)-1-phenyl-7-((*E*)-1,3,3-trimethylindolin-2-ylidene)­hepta-1,3,5-trien-1-yl)-3*H*-indol-1-ium Triflate (**Cy7–2**)

Prepared according to the general procedure above from **8** (50 mg; 0.20 mmol) and **3b** (0.15 g; 0.40 mmol). Yield: 10 mg (8%). Green solid. Mp: 166–168 °C. ^1^H NMR (500 MHz, *d*_4_-CD_3_OD) δ (ppm): 8.01 (d, *J* = 12.8 Hz, 1H), 7.89 (d, *J* = 12.8 Hz, 1H), 7.60 (t, *J* = 12.8 Hz, 1H), 7.55–7.45 (m, 5H), 7.41 (dd, *J* = 16.0, 8.0 Hz, 2H), 7.34 – 7.29 (m, 3H), 7.23 (dd, *J* = 16.0, 8.0 Hz, 3H), 6.37 (t, *J* = 12.8 Hz, 1H), 6.19 (d, *J* = 12.8 Hz, 1H), 6.05 (t, *J* = 12.8 Hz, 1H), 3.56 (s, 3H), 3.16 (s, 3H), 1.75 (s, 6H), 1.69 (s, 6H). ^13^C­{^1^H} NMR (126 MHz, *d*_4_-CD_3_OD) δ (ppm): 173.9, 172.0, 155.4, 151.5, 150.8, 143.9, 143.0, 141.3, 140.9, 137.1, 131.4, 131.2, 128.7, 128.3, 128.0, 127.9, 125.7, 125.3, 124.5, 122.7, 121.8, 121.7, 119.1, 117.9, 116.6, 110.8, 110.1, 103.3, 50.7, 48.7, 35.1, 29.9, 26.7, 26.5 (Figures S59–S62). HRMS (ESI-TOF) *m*/*z*: [M – OTf^–^]^+^ calcd for C_35_H_37_N_2_^+^ 485.2951; found 485.2953 (Figure S108).
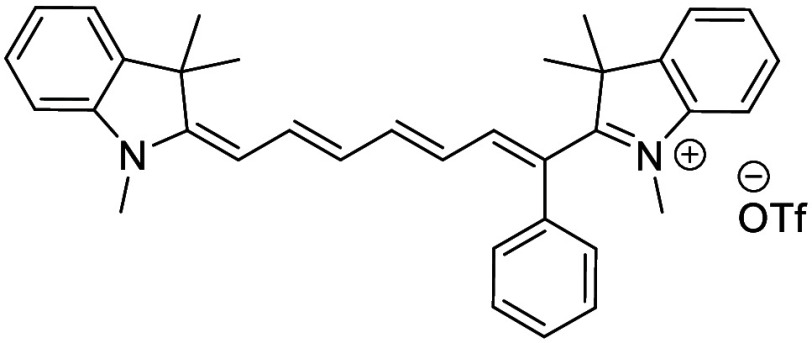


#### 2-((1*Z*,3*E*,5*E*)-1-Methoxy-7-((*E*)-1,3,3-trimethylindolin-2-ylidene)­hepta-1,3,5-trien-1-yl)-3-methylbenzo­[*d*]­thiazol-3-ium Triflate (**Cy7–3**)

Prepared according to the general procedure above from **8** (50 mg; 0.20 mmol) and **4a** (0.12 g; 0.29 mmol). Yield: 35 mg (31%). Green solid. Mp: 105–108 °C. ^1^H NMR (500 MHz, *d*_3_-CD_3_CN) δ (ppm): 8.08 (d, *J* = 7.9 Hz, 1H), 7.89 (d, *J* = 7.9 Hz, 1H), 7.80 (t, *J* = 7.9 Hz, 1H), 7.67 (t, *J* = 7.9 Hz, 1H), 7.49 (t, *J* = 7.9 Hz, 1H), 7.36 – 7.23 (m, 4H), 7.03 (t, *J* = 7.9 Hz, 1H), 6.94 (d, *J* = 7.9 Hz, 1H), 6.64–6.57 (m, 1H), 6.46–6.36 (m, 1H), 5.77 (d, *J* = 12.7 Hz, 1H), 4.20 (s, 3H), 3.86 (s, 3H), 3.32 (s, 3H), 1.62 (s, 6H). (Figure S63). It was not possible to record the ^13^C NMR spectrum due to the instability of the compound in solution. HRMS (ESI-TOF) *m*/*z*: [M – OTf ^–^]^+^ calcd for C_27_H_29_N_2_OS^+^ 429.1995; found 429.1994 (Figure S109).
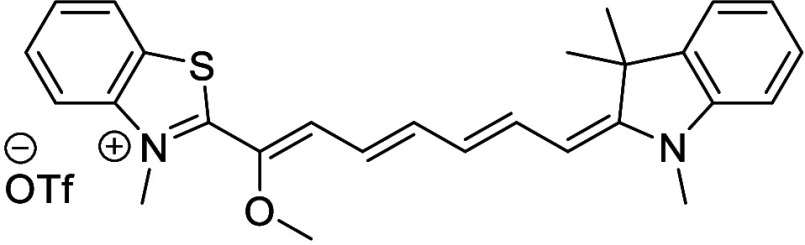


#### 3-Methyl-2-((1*E*,3*E*,5*E*)-1-phenyl-7-((*E*)-1,3,3-trimethylindolin-2-ylidene)­hepta-1,3,5-trien-1-yl)­benzo­[*d*]­thiazol-3-ium Triflate (**Cy7–5**)

Prepared according to the general procedure above from **8** (50 mg; 0.20 mmol) and **4d** (0.1 g; 0.26 mmol). Yield: 60 mg (54%). Green solid. Mp: 186–188 °C. ^1^H NMR (500 MHz, *d*_3_-CD_3_CN) δ (ppm): 8.08 (s, 1H), 7.86 (m, 2H), 7.71 (s, 1H), 7.55 (d, *J* = 6.6 Hz, 4H), 7.44 (d, *J* = 6.6 Hz, 3H), 7.37–7.24 (m, 3H), 7.01 (t, *J* = 7.0 Hz, 1H), 6.91 (d, *J* = 7.0 Hz, 1H), 6.32 – 6.18 (m, 2H), 5.67 (d, *J* = 7.0 Hz, 1H), 3.89 (s, 3H), 3.27 (s, 3H), 1.60 (s, 6H). ^13^C NMR gave an inconsistent result due to the aggregation of the compound. (Figures S64–S67). HRMS (ESI-TOF) *m*/*z*: [M – OTf ^–^]^+^ calcd for C_32_H_31_N_2_S^+^ 475.2202; found 475.2199 (Figure S110).
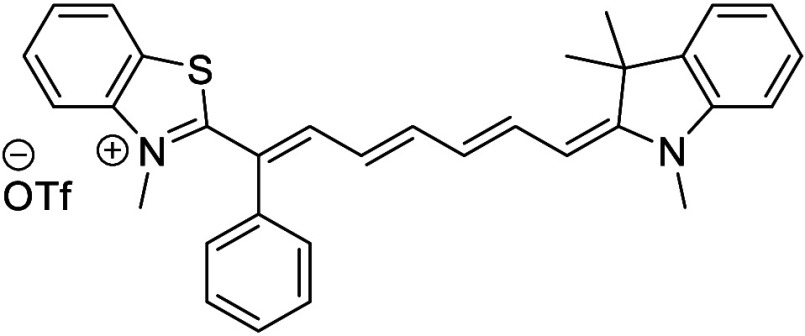


#### 2-((1*Z*,3*E*,5*E*)-1-Acetoxy-7-((*E*)-1,3,3-trimethylindolin-2-ylidene)­hepta-1,3,5-trien-1-yl)-3-methylbenzo­[*d*]­thiazol-3-ium Triflate (**Cy7–6**)

Prepared according to the general procedure above from **8** (80 mg; 0.31 mmol) and **4e** (0.2 g; 0.54 mmol). Yield: 50 mg (26%). Green solid. Mp: 124–126 °C. ^1^H NMR (500 MHz, *d*_4_-CD_3_OD) δ (ppm): 7.91 (d, *J* = 8.0 Hz, 1H), 7.78 (d, *J* = 8.0 Hz, 1H), 7.64 (t, *J* = 13.1 Hz, 1H), 7.57 (t, *J* = 13.1 Hz, 1H), 7.49 (m, 2H), 7.33 (d, *J* = 13.1 Hz, 1H), 7.25 (d, *J* = 7.4 Hz, 1H), 7.20 (t, *J* = 7.4 Hz, 1H), 7.02–6.94 (m, 2H), 6.36 (t, *J* = 12.7 Hz, 1H), 6.20 (t, *J* = 12.7 Hz, 1H), 5.85 (d, *J* = 12.7 Hz, 1H), 4.10 (s, 3H), 3.33 (s, 3H), 2.35 (s, 3H), 1.54 (s, 6H). ^13^C­{^1^H} NMR (126 MHz, *d*_4_-CD_3_OD) δ (ppm): 168.9, 153.4, 146.7, 143.7, 143.4, 140.0, 138.4, 129.0, 128.0, 126.8, 126.7, 126.3, 124.9, 122.8, 122.8, 121.7, 121.6, 119.2, 117.6, 117.5, 114.5, 108.6, 100.4, 100.2, 36.9, 29.2, 26.8, 18.8 (Figures S68–S71). HRMS (ESI-TOF) *m*/*z*: [M – OTf ^–^]^+^ calcd for C_28_H_29_N_2_O_2_S^+^ 457.1944; found 457.1946 (Figure S111).
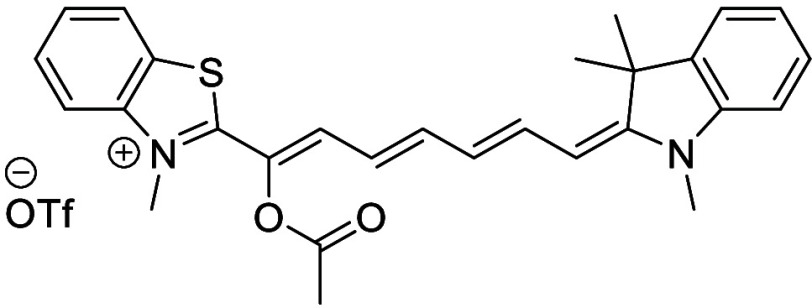


#### 3-Methyl-2-((1*Z*,3*E*,5*E*)-1-(methylthio)-7-((*E*)-1,3,3-trimethylindolin-2-ylidene)­hepta-1,3,5-trien-1-yl)­benzo­[*d*]­thiazol-3-ium Triflate (**Cy7–7**)

Prepared according to the general procedure above from **8** (80 mg; 0.31 mmol) and **4f** (0.13 g; 0.36 mmol). Yield: 50 mg (26%). Green solid. Mp: 130–133 °C. ^1^H NMR (500 MHz, *d*_4_-CD_3_OD) δ (ppm): 7.91 (d, *J* = 8.0 Hz, 1H), 7.76 (d, *J* = 8.0 Hz, 1H), 7.68–7.54 (m, 3H), 7.45 (m, 2H), 7.28–7.15 (m, 2H), 6.98 (dd, *J* = 12.4, 8.0 Hz, 2H), 6.79 (t, *J* = 12.4 Hz, 1H), 6.47 – 6.35 (m, 1H), 5.87 (d, *J* = 12.4 Hz, 1H), 4.10 (s, 3H), 3.33 (s, 3H), 2.30 (s, 3H), 1.54 (s, 6H). ^13^C­{^1^H} NMR (126 MHz, *d*_4_-CD_3_OD) δ (ppm): 173.8, 168.0, 167.9, 154.5, 150.4, 147.0, 144.5, 143.6, 140.0, 126.6, 128.6, 128.0, 126.5, 124.9, 122.8, 122.7, 122.3, 121.7, 121.6, 119.2, 114.6, 108.6, 100.3, 38.2, 29.2, 26.9, 17.2 (Figures S72–S75). HRMS (ESI-TOF) *m*/*z*: [M – OTf ^–^]^+^ calcd for C_27_H_29_N_2_S_2_^+^ 445.1767; found 445.1771 (Figure S112).
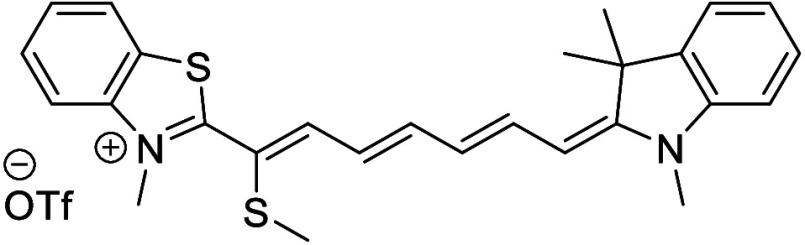


#### 2-((1*Z*,3*E*,5*E*)-1-Acetamido-7-((*E*)-1,3,3-trimethylindolin-2-ylidene)­hepta-1,3,5-trien-1-yl)-3-methylbenzo­[*d*]­thiazol-3-ium Triflate (OB206 **Cy7–8**)

Prepared according to the general procedure above from **8** (80 mg; 0.31 mmol) and **4g** (0.23 g; 0.62 mmol). Yield: 60 mg (32%). Green solid. Mp: 145–147 °C. ^1^H NMR (500 MHz, *d*_4_-CD_3_OD) δ (ppm): 7.98 (d, *J* = 7.7 Hz, 1H), 7.87 (d, *J* = 7.7 Hz, 1H), 7.70 (t, *J* = 7.7 Hz, 1H), 7.57 (d, *J* = 7.7 Hz, 1H), 7.43 (t, *J* = 12.4 Hz, 1H), 7.26 – 7.12 (m, 4H), 6.90 (t, *J* = 7.7 Hz, 1H), 6.84 (d, *J* = 7.7 Hz, 1H), 6.39–6.26 (m, 2H), 5.68 (d, *J* = 12.4 Hz, 1H), 4.10 (s, 3H), 3.24 (s, 3H), 2.12 (s, 3H), 1.51 (s, 6H). ^13^C {^1^H} NMR (126 MHz, *d*_4_-CD_3_OD) δ (ppm): 171.6, 170.5, 165.2, 151.3, 144.0, 143.7, 143.1, 141.5, 139.5, 129.1, 127.8, 127.3, 124.4, 123.0, 121.7, 121.4, 119.2, 118.8, 115.2, 113.3, 107.7, 98.7, 37.0, 28.8, 27.0, 20.9 (Figures S76–S79). HRMS (ESI-TOF) *m*/*z*: [M – OTf^–^]^+^ calcd for C_28_H_30_N_3_OS^+^ 456.2104; found 456.2108 (Figure S113).
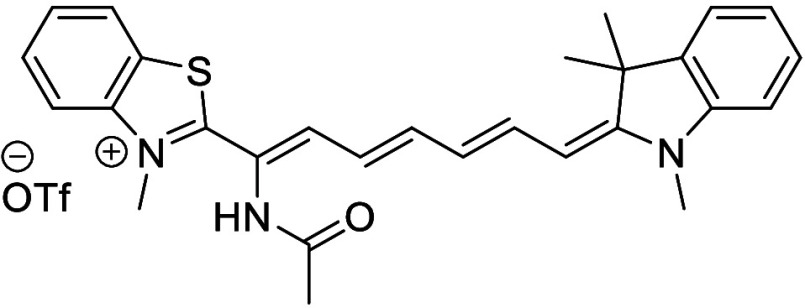


#### 2-((1*Z*,3*E*,5*E*)-1-((*tert*-Butyldimethylsilyl)­oxy)-7-((*E*)-1,3,3-trimethylindolin-2-ylidene)­hepta-1,3,5-trien-1-yl)-3-methylbenzo­[*d*]­thiazol-3-ium Triflate (**Cy7–9**)

Prepared according to the general procedure from **8** (60 mg; 0.24 mmol) and **4h** (0.23 g; 0.67 mmol). Yield: 40 mg (25%). Green solid. Mp: 124–127 °C. ^1^H NMR (300 MHz, *d*_4_-CD_3_OD) δ (ppm): 8.19 (d, *J* = 8.0 Hz, 1H), 8.06 (d, *J* = 8.0 Hz, 1H), 7.92–7.83 (m, 1H), 7.73 (t, *J* = 12.9 Hz, 1H), 7.41 (t, *J* = 12.9 Hz, 1H), 7.29 – 7.12 (m, 4H), 7.00–6.86 (m, 2H), 6.56–6.45 (m, 1H), 6.40–6.27 (m, 1H), 5.70 (d, *J* = 12.9 Hz, 1H), 4.34 (s, 3H), 3.29 (s, 3H), 1.61 (s, 6H), 1.20–1.07 (m, 9H), 0.24 (s, 6H) (Figure S80). It was not possible to record the ^13^C NMR and HRMS measurements properly due to the instability of the compound in solution.
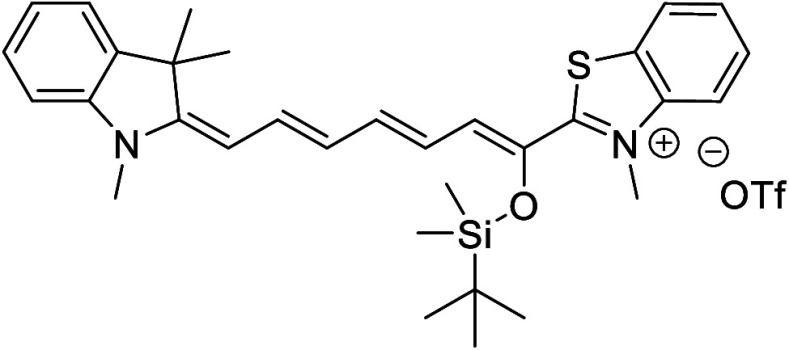


#### 2-((1*Z*,3*E*,5*E*)-1-((*tert*-Butoxycarbonyl)­amino)-7-((*E*)-1,3,3-trimethylindolin-2-ylidene)­hepta-1,3,5-trien-1-yl)-3-methylbenzo­[*d*]­thiazol-3-ium Triflate (**Cy7–10**)

Prepared according to the general procedure above from **8** (0.1 g; 0.40 mmol) and **4i** (0.42 g; 0.98 mmol). Yield: 90 mg (34%). Green solid. Mp: 153–15 °C. ^1^H NMR (500 MHz, *d*_4_-CD_3_OD) δ (ppm): 8.13 (d, *J* = 7.8 Hz, 1H), 8.02 (d, *J* = 8.4 Hz, 1H), 7.84 (t, *J* = 7.8 Hz, 1H), 7.71 (d, *J* = 7.8 Hz, 1H), 7.58 – 7.48 (m, 1H), 7.28 (m, 4H), 7.04–6.93 (m, 2H), 6.53–6.39 (m, 2H), 5.79 (d, *J* = 8.4 Hz, 1H), 4.26 (s, 3H), 3.35 (s, 3H), 1.63 (s, 6H), 1.54 (s, 9H). ^13^C NMR gave an inconsistent result due to the aggregation of the compound. (Figures S81–S84). HRMS (ESI-TOF) *m*/*z*: [M – OTf ^–^]^+^ calcd for C_31_H_36_N_3_O_2_S^+^ 514.2523; found 514.2526 (Figure S114).
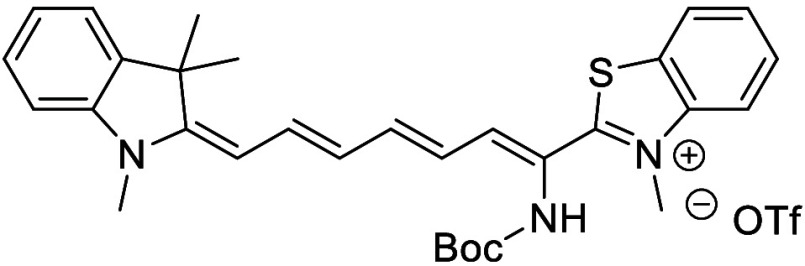


#### 1,3,3-Trimethyl-2-((1*E*,3*E*,5*E*,7*Z*)-7-(3-methylbenzo­[*d*]­thiazol-2­(3*H*)-ylidene)­deca-1,3,5-trien-9-yn-1-yl)-3*H*-indol-1-ium Triflate (**Cy7–11**)

Prepared according to the general procedure above from **8** (50 mg; 0.20 mmol) and **4j** (0.14 g; 0.40 mmol). Green solid. Yield: 46 mg (40%). Mp: 105–109 °C. ^1^H NMR (500 MHz, *d*_4_-CD_3_OD) δ (ppm): 8.06 (d, *J* = 7.9 Hz, 1H), 7.95 (d, *J* = 7.9 Hz, 1H), 7.75 (t, *J* = 7.9 Hz, 1H), 7.63 (t, *J* = 7.9 Hz, 1H), 7.44–7.25 (m, 2H), 7.21 – 7.10 (m, 3H), 6.92 – 6.78 (m, 2H), 6.37 (d, *J* = 7.9 Hz, 2H), 5.62 (d, *J* = 7.9 Hz, 1H), 4.17 (s, 3H), 3.61 (d, *J* = 2.4 Hz, 2H), 2.51 (d, *J* = 2.4 Hz, 1H), 1.50 (s, 6H).^13^C­{^1^H} NMR (126 MHz, *d*_4_-CD_3_OD) δ (ppm): 144.2, 142.9, 139.4, 129.3, 129.2, 127.8, 127.5, 126.4, 125.6, 123.1, 123.0, 121.4, 115.7, 115.3, 107.4, 79.3, 71.2, 70.8, 55.3, 40.8, 37.9, 35.8, 28.7, 27.1, 21.8, 20.8, 20.6 (Figures S85–S88). HRMS (ESI-TOF) *m*/*z*: [M – OTf ^–^]^+^ calcd for C_29_H_29_N_2_S^+^ 437.2046; found 437.2044 (Figure S115).
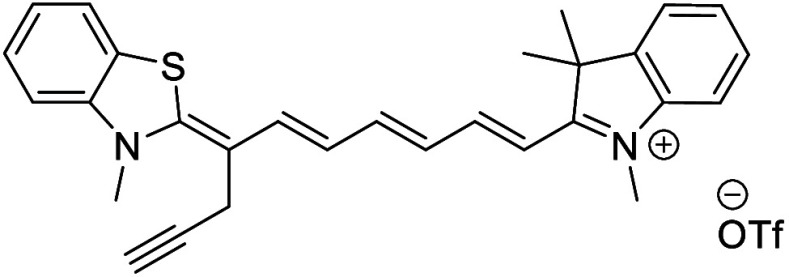


#### 1,3,3-Trimethyl-2-((2*E*,4*E*,6*E*,8*Z*)-8-(3-methylbenzo­[*d*]­thiazol-2­(3*H*)-ylidene)­octa-2,4,6-trien-2-yl)-3*H*-indol-1-ium Triflate (OB281 **Cy7–18**)

Prepared according to the general procedure above from **15** (50 mg; 0.20 mmol) and **3c** (0.2 g; 0.59 mmol). Green solid. Yield: 30 mg (27%). Mp: 164–166 °C. ^1^H NMR (300 MHz, *d*_4_-CD_3_OD) δ (ppm): 7.92 (d, *J* = 8.1 Hz, 1H), 7.75 (d, *J* = 8.1 Hz, 1H), 7.70–7.62 (m, 2H), 7.50 (t, *J* = 8.1 Hz, 1H), 7.44–7.31 (m, 3H), 7.19–7.07 (m, 2H), 6.67 (dd, *J* = 18.7, 13.2 Hz, 2H), 6.51 (dd, *J* = 18.7, 13.2 Hz, 2H), 3.95 (s, 3H), 3.70 (s, 3H), 2.23 (s, 3H), 1.66 (s, 6H).^13^C­{^1^H} NMR (126 MHz, *d*_4_-CD_3_OD) δ (ppm): 171.9, 166.2, 153.2, 149.8, 149.0, 145.1, 142.2, 140.5, 128.3, 128.0, 126.2, 125.8, 124.5, 123.5, 122.5, 121.4, 120.1, 113.5, 109.6, 108.4, 104.2, 49.3, 36.9, 32.9, 26.5, 14.1 (Figures S97–S100). HRMS (ESI-TOF) *m*/*z*: [M – OTf ^–^]^+^ calcd for C_27_H_29_N_2_S^+^ 413.2046; found 413.2043 (Figure S120).
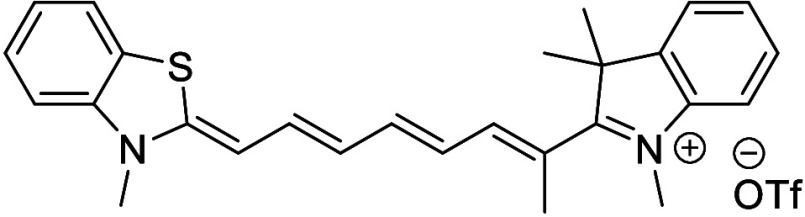


#### General Procedure for the Synthesis of Dicyano Heptamethine Cyanine Dyes from Fischer’s Bases

Heterocycle **1a**, **1c**, **1d**, or **1e** (0.50 mmol) and glutaconaldehydedianil hydrochloride **10** (0.25 mmol) were added to fresh distilled acetic anhydride (0.5 mL). The reaction was heated to 95–100 °C (heating mantle) for approximately 1 h (monitored by a UV–vis spectrometer). After cooling, methanol (2 mL) was added, and then diethyl ether (∼15 mL) was added dropwise. The flask was left at – 20 °C overnight. The precipitate was filtered and washed 3× times with diethyl ether (∼5 mL) and *n*-pentane (∼5 mL). Product was obtained as a dark solid.
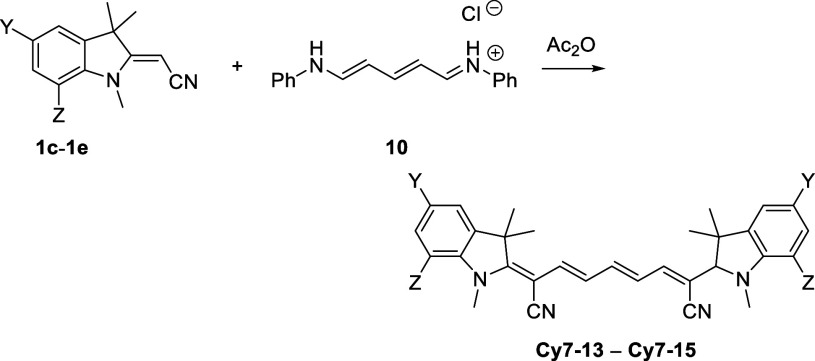


#### 2-((1*Z*,3*E*,5*E*)-1,7-Dicyano-7-((*Z*)-5-methoxy-1,3,3-trimethylindolin-2-ylidene)­hepta-1,3,5-trien-1-yl)-5-methoxy-1,3,3-trimethyl-3*H*-indol-1-ium Chloride (**Cy7–13**)

Prepared according to the general procedure above from **10** (30 mg; 0.10 mmol) and **1c** (46 mg; 0.20 mmol). Yield: 25 mg (46%). Green solid. Mp: 168–173 °C. ^1^H NMR (500 MHz, *d*_4_-CD_3_OD) δ (ppm): 8.10 (d, *J* = 12.8 Hz, 2H), 7.98 (t, *J* = 12.8 Hz, 1H), 7.43 (d, *J* = 8.8 Hz, 2H), 7.20 (s, 2H), 7.07 (d, *J* = 8.8 Hz, 2H), 6.90 (t, *J* = 12.8 Hz, 2H), 4.03 (s, 6H), 3.90 (s, 6H), 1.77 (s, 12H).^13^C­{^1^H} NMR (126 MHz, *d*_4_-CD_3_OD) δ (ppm): 175.4, 160.5, 160.0, 153.9, 142.7, 136.3, 123.0, 115.9, 113.9, 113.1, 108.3, 85.2, 55.1, 51.9, 35.2, 24.2 (Figures S89 and S90). HRMS (ESI-TOF) *m*/*z*: [M – Cl^–^]^+^ calcd for C_33_H_35_N_4_O_2_^+^ 519.2755; found 519.2758 (Figure S116).
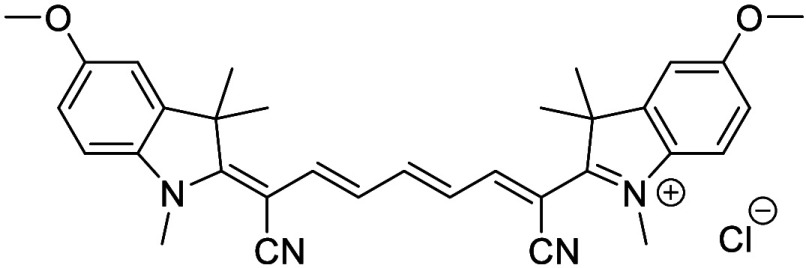


#### 2-((1*Z*,3*E*,5*E*)-1,7-Dicyano-7-((*Z*)-1,3,3,5,7-pentamethylindolin-2-ylidene)­hepta-1,3,5-trien-1-yl)-1,3,3,5,7-pentamethyl-3*H*-indol-1-ium Chloride (**Cy7–14**)

Prepared according to the general procedure above from **10** (73 mg; 0.26 mmol) and **1d** (0.11 g; 0.50 mmol). Yield: 40 mg (29%). Green solid. Mp: 144–145 °C. ^1^H NMR (500 MHz, *d*_4_-CD_3_OD) δ (ppm): 8.00 (d, *J* = 12.8 Hz, 1H), 7.90 (t, *J* = 12.8 Hz, 1H), 7.24 (s, 1H), 7.11 (s, 1H), 6.90 (t, *J* = 12.8 Hz, 1H), 4.15 (s, 3H), 2.71 (s, 3H), 2.40 (s, 3H), 1.73 (s, 6H).). ^13^C­{^1^H} NMR (126 MHz, *d*_4_-CD_3_OD) δ (ppm): 178.9, 160.1, 154.2, 141.9, 139.3, 137.8, 133.1, 124.1, 122.9, 120.7, 115.8, 85.0, 51.0, 40.7, 24.2, 19.6, 18.1 (Figures S91 and S92). HRMS (ESI-TOF) *m*/*z*: [M – Cl^–^]^+^ calcd for C_35_H_39_N_4_^+^ 515.3169; found 515.3166 (Figure S117).
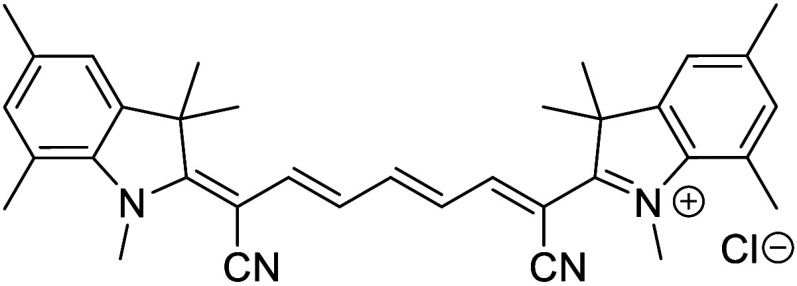


#### 2-((1*Z*,3*E*,5*E*)-1,7-Dicyano-7-((*Z*)-5-(dimethylamino)-1,3,3-trimethylindolin-2-ylidene)­hepta-1,3,5-trien-1-yl)-5-(dimethylamino)-1,3,3-trimethyl-3*H*-indol-1-ium Chloride (**Cy7–15**)

Prepared according to the general procedure above from **10** (25 mg; 0.09 mmol) and **1e** (36 mg; 0.15 mmol). Yield: 33 mg (76%). Green solid. Mp: 177–180 °C. ^1^H NMR (500 MHz, *d*_4_-CD_3_OD) δ (ppm): 8.09 (d, *J* = 12.7 Hz, 2H), 8.00 (t, *J* = 12.7 Hz, 1H), 7.65 (s, 2H), 7.57 (d, *J* = 8.8 Hz, 2H), 7.50 (d, *J* = 8.8 Hz, 2H), 6.96 (t, *J* = 12.7 Hz, 2H), 4.04 (s, 6H), 3.29 (s, 12H), 1.81 (s, 12H). ^13^C­{^1^H} NMR (126 MHz, *d*_4_-CD_3_OD) δ (ppm): 174.5, 158.8, 143.2, 124.8, 120.7, 118.4, 115.5, 144.3, 113.4, 111.7, 87.2, 51.8, 46.2, 43.9, 35.5, 24.6, 24.2 (Figures S93 and S94). HRMS (ESI-TOF) *m*/*z*: [M – Cl^–^]^+^ calcd for C_35_H_41_N_6_^+^ 545.3384; found 545.3384 (Figure S118).
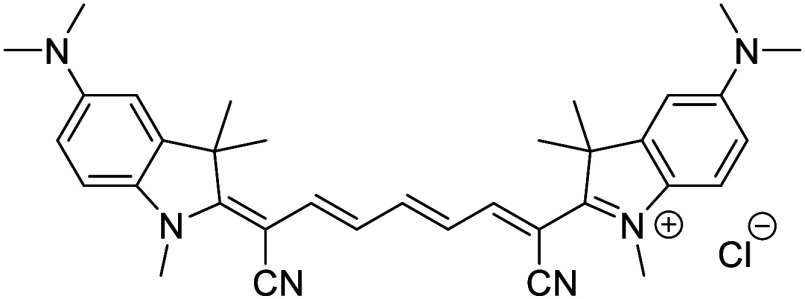


#### 5-Cyano-2-((1*Z*,3*E*,5*E*)-1-cyano-7-((*E*)-1,3,3-trimethylindolin-2-ylidene)­hepta-1,3,5-trien-1-yl)-1,3,3-trimethyl-3*H*-indol-1-ium Chloride (**Cy7–17**)

In a dry Schlenk flask, **1f** (0.12 g; 0.55 mmol) was added to dry tetrahydrofuran (1.6 mL). *n*-Butyllithium (a 6 M *n*-hexane solution; 0.72 mmol) was added dropwise at – 78 °C. After 15 min, a yellow precipitate appeared. **8** (50 mg; 0.2 mmol) was dissolved in dry tetrahydrofuran (0.4 mL), and the mixture was added dropwise. The reaction was stirred at – 78 °C for 20 min and then at room temperature for 1 h. Dichloromethane (∼10 mL) was added, and then water (3 mL) was added dropwise to quench the reaction. The organic phase was washed 2× with an aqueous HCl solution (pH = 3). The product was purified by chromatography on silica (*n*-hexane/ethyl acetate, 1:1, then dichloromethane/methanol, 100:0 to 96:4). Yield: 20 mg (25%). Green solid. Mp: 147–150 °C. ^1^H NMR (500 MHz, *d*_4_-CD_3_OD) δ (ppm): 8.19 (m, 1H), 7.79–7.70 (m, 4H), 7.59 (m, 4H), 7.26 (d, *J* = 8.3 Hz, 1H), 7.01 (d, *J* = 8.3 Hz, 1H), 6.91–6.86 (m, 1H), 6.79–6.71 (m, 1H), 4.00 (s, 3H), 3.82 (s, 3H), 1.79 (s, 6H), 1.75 (s, 6H). ^13^C­{^1^H} NMR (126 MHz, *d*_4_-CD_3_OD) δ (ppm): 180.7, 155.3, 151.2, 143.0, 142.0, 138.0, 137.9, 137.8, 133.4, 130.4, 130.2, 130.2, 129.0, 128.7, 125.4, 122.4, 118.6, 113.8, 112.8, 109.9, 51.6, 49.1, 32.4, 25.2 (Figures S95 and S96). HRMS (ESI-TOF) *m*/*z*: [M – Cl^–^]^+^ calcd for C_31_H_31_N_4_^+^ 459.2543; found 459.2541 (Figure S119).
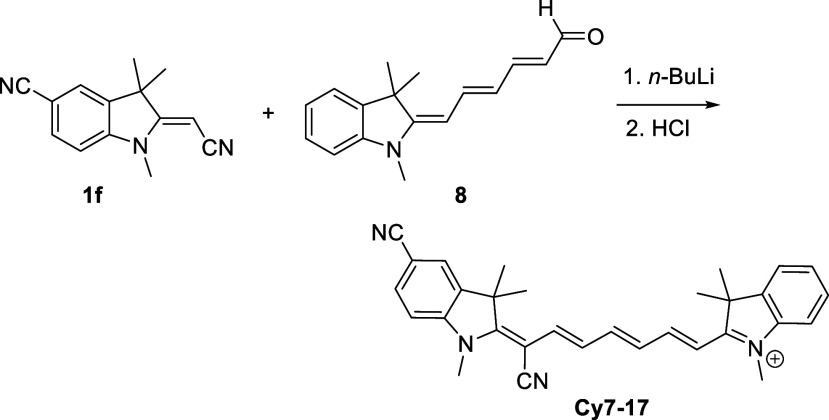


## Supplementary Material



## Data Availability

The data underlying this study are available in this manuscript, the Supporting Information, and on Zenodo (https://zenodo.org/records/20119205).

## Notes

Materials and methods, ^1^H, ^13^C­{1H}, gHSQC, gHMBC, COSY, and NOESY NMR spectra, MS data, p*K*_a_ measurements, HPLC data, and absorption and emission spectra (PDF)
