# Supplementary material for: C1′-Substituted Penta- and Heptamethine Cyanines: Synthetic Scope and Properties
Source: J Org Chem. 2026 Jul 6;91(28):9718–38. doi: 10.1021/acs.joc.6c00573 (PMC13386540; doi:10.1021/acs.joc.6c00573)
Supplement: Supplementary file 1 [file jo6c00573_si_001.pdf]

Supporting Information

C1´-Substituted Penta- and Heptamethine Cyanines: Synthetic Scope and Properties

Ottavio Bedocchi,[†,‡] *Jana Okoročenkova,*[†,‡] *Petr Klán*[†,‡,*]

[†] Department of Chemistry, Faculty of Science, Masaryk University, Kamenice 5, 625 00, Brno, Czech Republic.

[‡] RECETOX, Faculty of Science, Masaryk University, Kamenice 5, 625 00, Brno, Czech Republic.

AUTHOR EMAIL ADDRESS: *klan@sci.muni.cz*

Content

Materials and Methods ____ S2

NMR Spectra ____ S4

MS Spectra ____ S104

Absorption and Emission Spectra ____ S111

Spectroscopic Determination of pK_a ____ S116

Nucleophile Sensing ____ S119

HPLC Measurements ____ S122

Fluorescence Lifetimes ____ S138

References ____ S139

Materials and Methods

Reagents and solvents of the highest purity available were used as purchased unless stated otherwise. Compounds **Cy5-1**, **9**, and **10** were purchased used without further purification. Compounds **Cy5-2**, **Cy5-4**, **Cy5-6**, **Cy5-9**, **Cy7-1**, **Cy7-4**, **Cy7-12**, and **Cy7-16** were prepared as previously described.[1] The synthetic procedures were performed under an ambient atmosphere unless stated otherwise. Column chromatography was performed using silica gel 60. ^{1}H NMR spectra were recorded on 300, 400 or 500 MHz spectrometers and ^{13}C NMR were obtained on 125 MHz or 75 MHz instruments in $CDCl_3$, CD_3OD, or $(CD_3)_2SO$. ^{1}H chemical shifts are reported in ppm relative to tetramethylsilane (δ = 0.00 ppm) using the residual solvent signal as an internal reference. ^{13}C chemical shifts are reported in ppm with $CDCl_3$ (δ = 77.67 ppm), CD_3OD (δ = 49.30 ppm), or $(CD_3)_2SO$ (δ = 39.52 ppm) as internal references. Structural assignments were made using additional information from gHSQC, gHMBC, COSY and NOESY experiments. The deuterated solvents were kept under a nitrogen atmosphere.

Absorption spectra were obtained with matched 1.0 cm quartz cuvettes using a UV-vis spectrometer. Molar absorption coefficients were determined from the absorption spectra (the average values were obtained from three independent measurements with solutions of different concentrations). Fluorescence was measured on an automated luminescence spectrometer in 1.0 cm quartz fluorescence cuvettes at 23 ± 1 °C; sample concentrations with an absorbance below 0.1 at the excitation wavelength were used. Fluorescence quantum yields were determined on a fluorimeter as absolute values using an integrating sphere. The quantum yields were measured five times and were averaged for each sample. The solution concentrations were adjusted to have absorbance below 0.15.

The exact masses of the synthesized compounds were obtained using a triple quadrupole electrospray ionization (ESI) mass spectrometer in a positive or negative mode coupled with direct inlet or liquid chromatography (nitrogen flow 5 L min^{-1}, gas temperature: 325 °C, nebulizer 45 psig, skimmer 65 V, V_{cap} –2500 V, fragmentor: 60 V; in methanol) and an atmospheric pressure chemical ionization (APCI) mass spectrometer in a positive or negative mode coupled with direct inlet or liquid chromatography (nitrogen flow 5 L min^{-1}, gas temperature: 325 °C, nebulizer 45 psig, skimmer 65 V, vaporizer 200 °C, fragmentor: 60 V; in dichloromethane).

HPLC analyses were carried out on a chromatograph equipped with a photodiode array detector (200–900 nm) using Zorbax SB-Aq C18 column at 30 °C and flow rate of 1 mL min^{-}1 (3.5 µm, 4.6 × 150 mm). The mobile phase was 0.1% trifluoroacetic acid in water (A) and acetonitrile (B), programmed from 10% to 90% of B over 6 min, from 90% to 95% of B over 0.5 min, kept 95% B for 0.5 min, and re-equilibrated with 10% of solution B for 3 min.

Singlet Oxygen Production. Determination of the singlet oxygen production quantum yield (Φ_Δ): A solution of 1,3-diphenylisobenzofuran (DPBF; $c = 5 \times 10^{-5}$) and a Cy5 or Cy7 derivative ($c \sim 1 \times 10^{-5}$ M) in methanol was prepared. The stirred solution (3.0 mL) in a quartz cell (1 cm) was irradiated using LEDs at 730 nm for Cy7s and 638 nm for all Cy5s, **Cy7-16**, and **Cy7-17**, and the UV-vis spectra were recorded periodically. The irradiation period was selected to reach a ∼10% consumption of DPBF. The procedure was repeated three times. The decomposition of DPBF monitored at 411 nm was fitted with a pseudo-first-order rate law, and the singlet oxygen formation quantum yield (Φ_Δ) was calculated using that of **Cy7-1** as a reference (Φ_Δ = 0.009)[2] for **Cy7-2**–**Cy7-11**, **Cy7-13**–**Cy7-15**, and **Cy7-18**. Methylene blue (Φ_Δ = 0.49)[3] was used as a reference for compounds **Cy5-2**–**Cy5-12**, **Cy7-16**, and **Cy7-17**.

Photostability. The decomposition quantum yields (Φ_{dec}) of **Cy7-2–Cy7-11**, **Cy7-13–Cy7-15**, and **Cy7-18** in an aerated methanol solution were determined using an LED light source (λ_{irr} = 730 nm). The quantum yields were measured three times and were averaged for each sample. Unsubstituted (parent) Cy7 (Φ_{dec} = 3.1 × 10^{-6})[2] was used as a reference. The decomposition quantum yields of **Cy5-2–Cy5-12**, **Cy7-16**, and **Cy7-17** in an aerated methanol solution were determined using LED (λ_{irr} = 638 nm). The Φ_{dec} values were measured three times and were averaged for each sample. The quantum yield of decomposition of unsubstituted (parent) **Cy5-1** at 640 nm (Φ_{dec} = 3.7 × 10^{-7}) was determined using a photodiode and was used as a reference.[1]

Determination of p*K*a. *Method A*: A freshly prepared solution of a Cy7 derivative (**Cy7-12–Cy7-17**; *c* ~ 2.0 × 10^{-5} M) in water/methanol (95 : 5, *v/v*) was transferred into a matched 1.0 cm quartz cuvette. After the addition of aq. NaOH (0.01 M) to reach pH~ 12, the UV-vis absorption spectrum was recorded. The solution was acidified by the addition of small aliquots of aq. HCl, and the pH and UV-vis absorption spectra were recorded upon each addition. The total volume change during titration was corrected for dilution effects. *Method B:* The p*K*a values of **Cy7-12**, **Cy7-13**, and **Cy7-16** were also determined by the following procedure: A compound was dissolved in a series of buffered solutions (Britton-Robinson buffer (containing 5% methanol; ionic strength = 0.1 M adjusted with KCl)[4] across the range of pH = 2.2 to 11.5, which was followed by titration monitored using UV-vis spectroscopy. The results were consistent across both methods A and B.

Nucleophile Sensing. A freshly prepared solution of a **Cy7-13** (*c* ~1.3 × 10^{-5} M) in Britton-Robinson buffer/methanol (95 : 5, *v/v*) at pH = 5.1 was transferred into a matched 1.0 cm quartz cuvette, and the UV-vis absorption spectrum was recorded. The solution was treated by the addition of small aliquots of a methanol solution of the nucleophile (*c* ~3 × 10^{-2} M), and the UV-vis absorption spectra were recorded upon each addition. A similar procedure was performed for **Cy7-16** (*c* ~2 × 10^{-5} M) in PBS/methanol (95 : 5, *v/v*) and for **Cy7-17** (*c* ~2 × 10^{-5} M) in Britton-Robinson buffer/methanol (95 : 5, *v/v*) both at pH = 7.4.

NMR Spectra

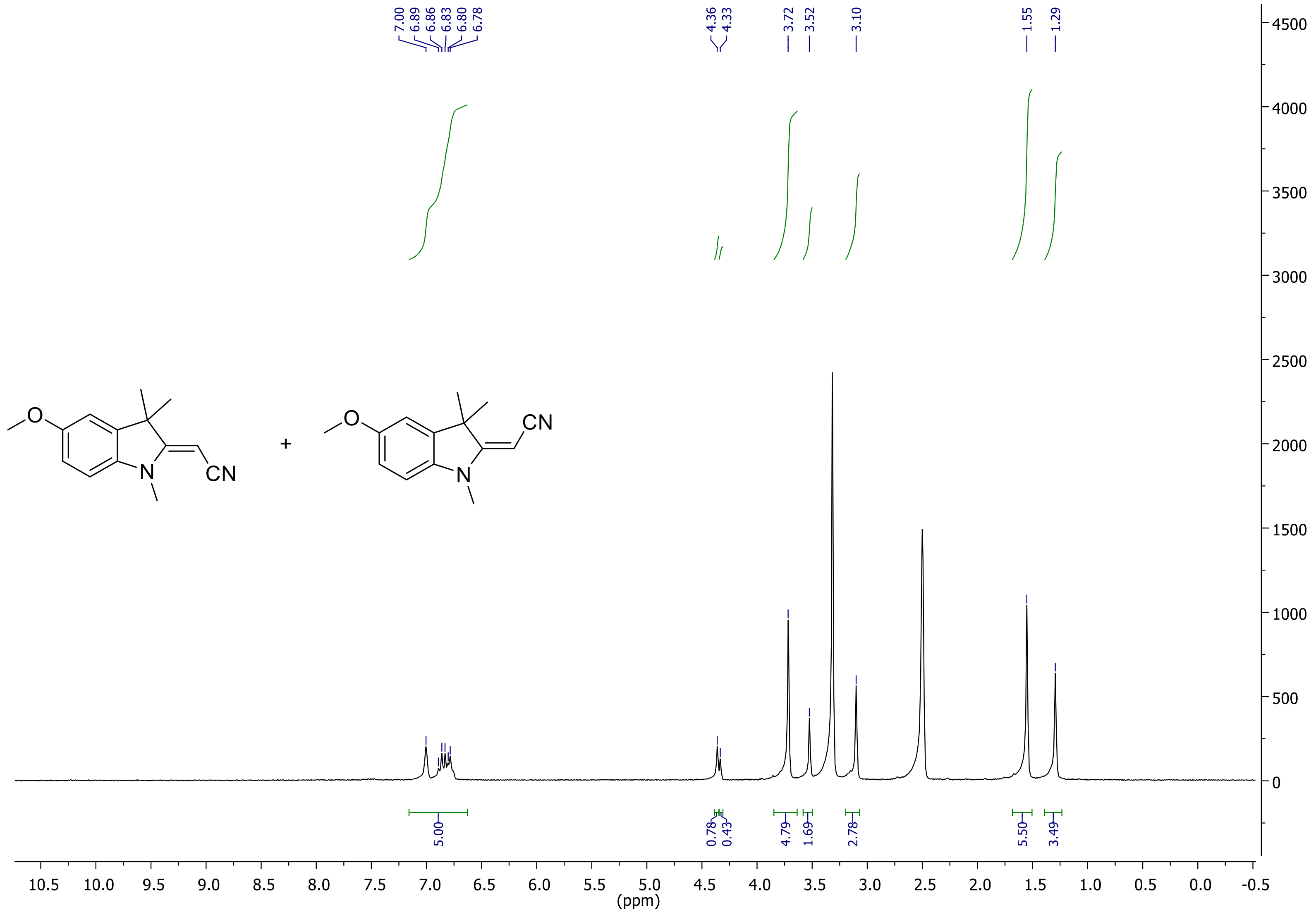

Figure S1. ^{1}H NMR (300 MHz, d_6-DMSO): **1c**.

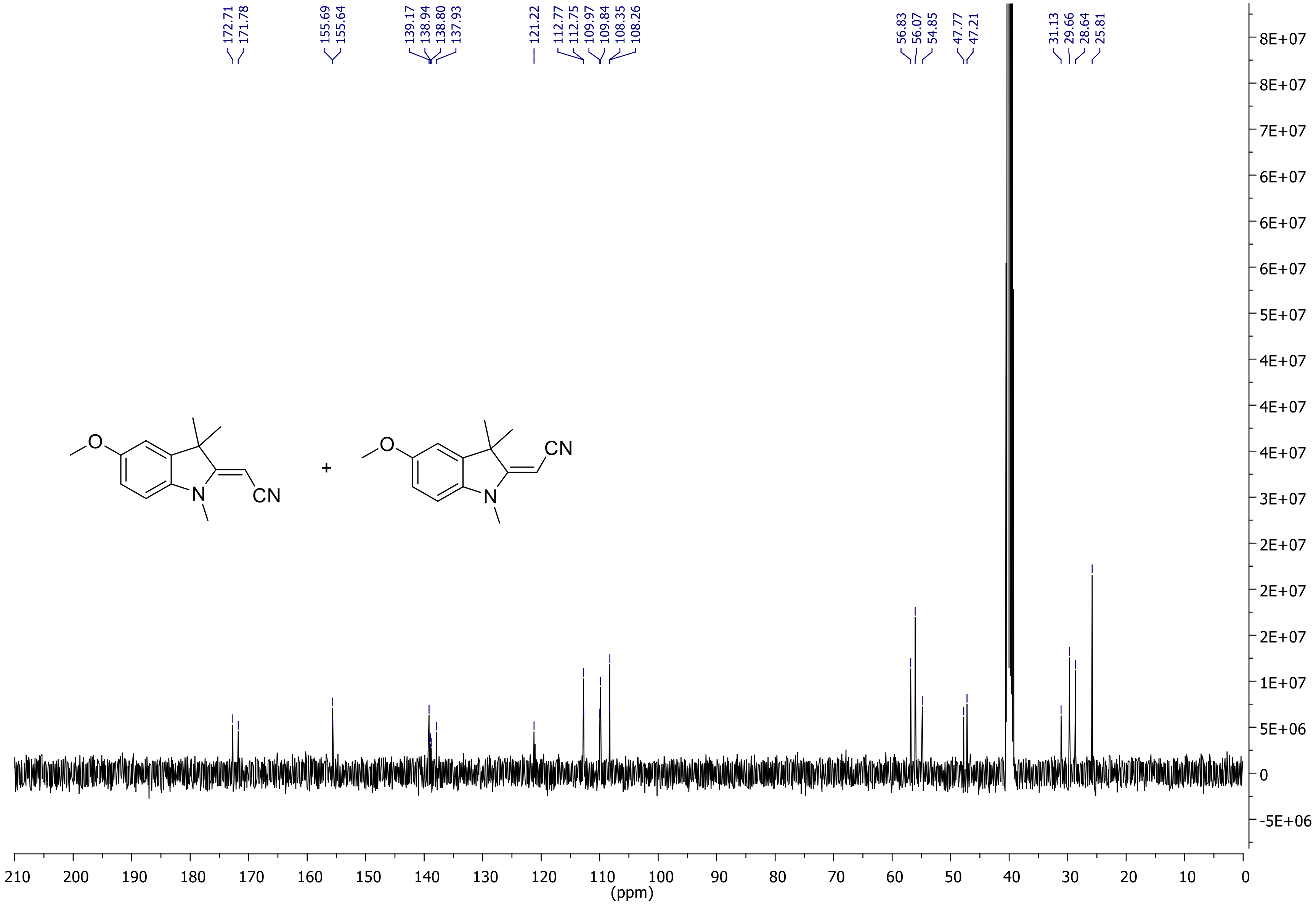

Figure S2. $^{13}C\{^{1}H\}$ NMR (126 MHz, d_6-DMSO): **1c**.

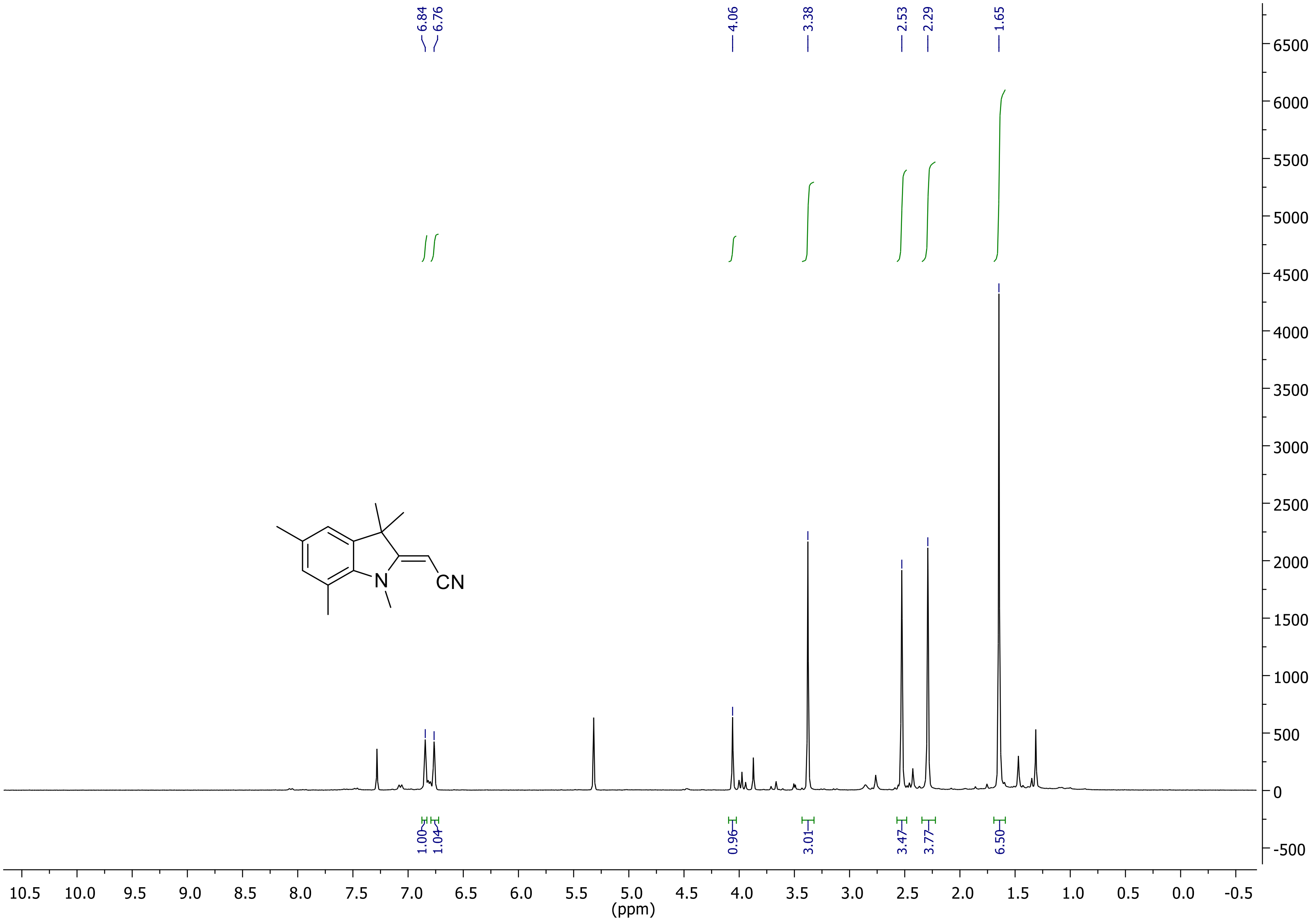

Figure S3. ^1H NMR (300 MHz, $CDCl_3$): **1d** (* dichloromethane).

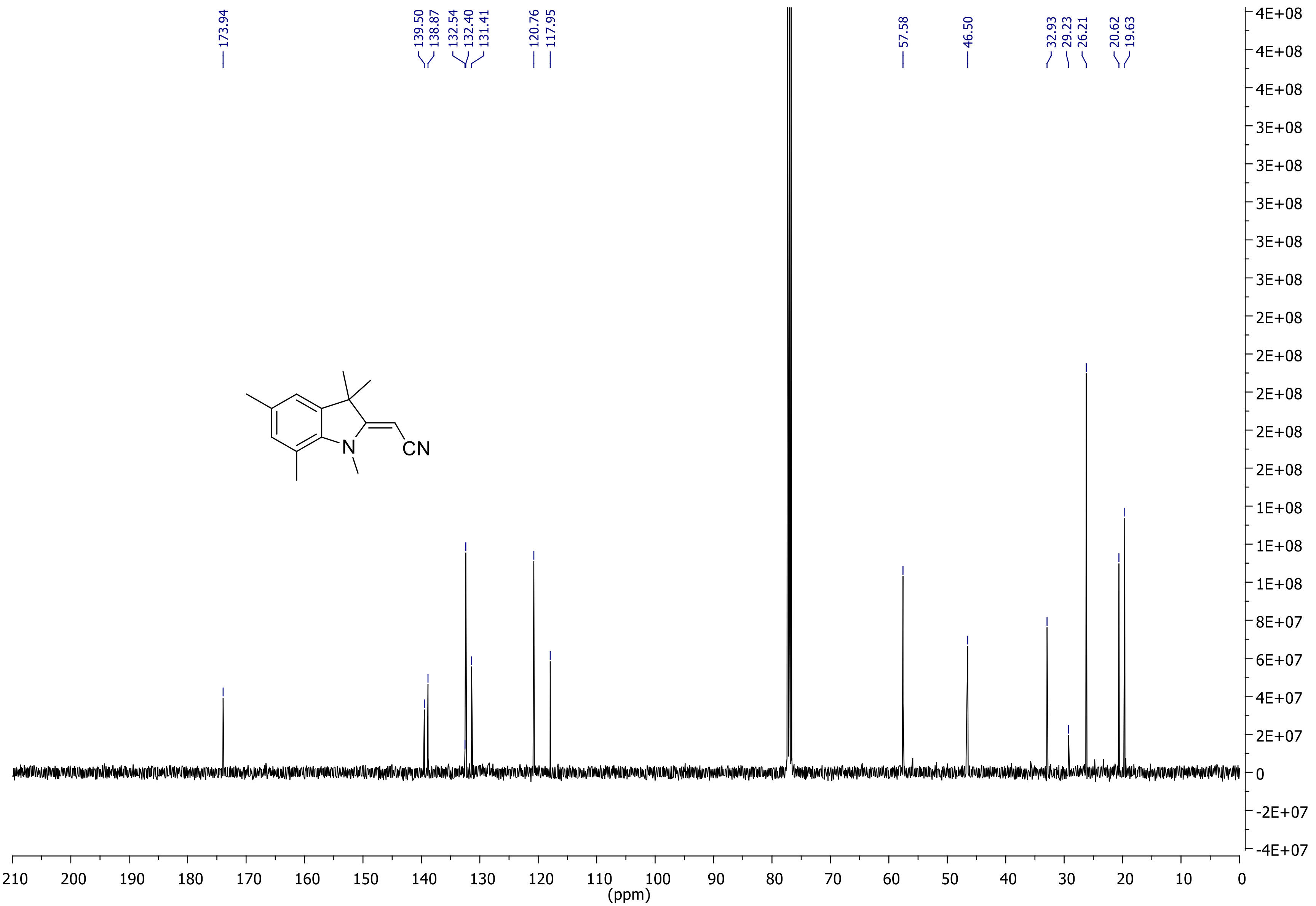

Figure S4. $^{13}C\{^{1}H\}$ NMR (126 MHz, $CDCl_3$): **1d**.

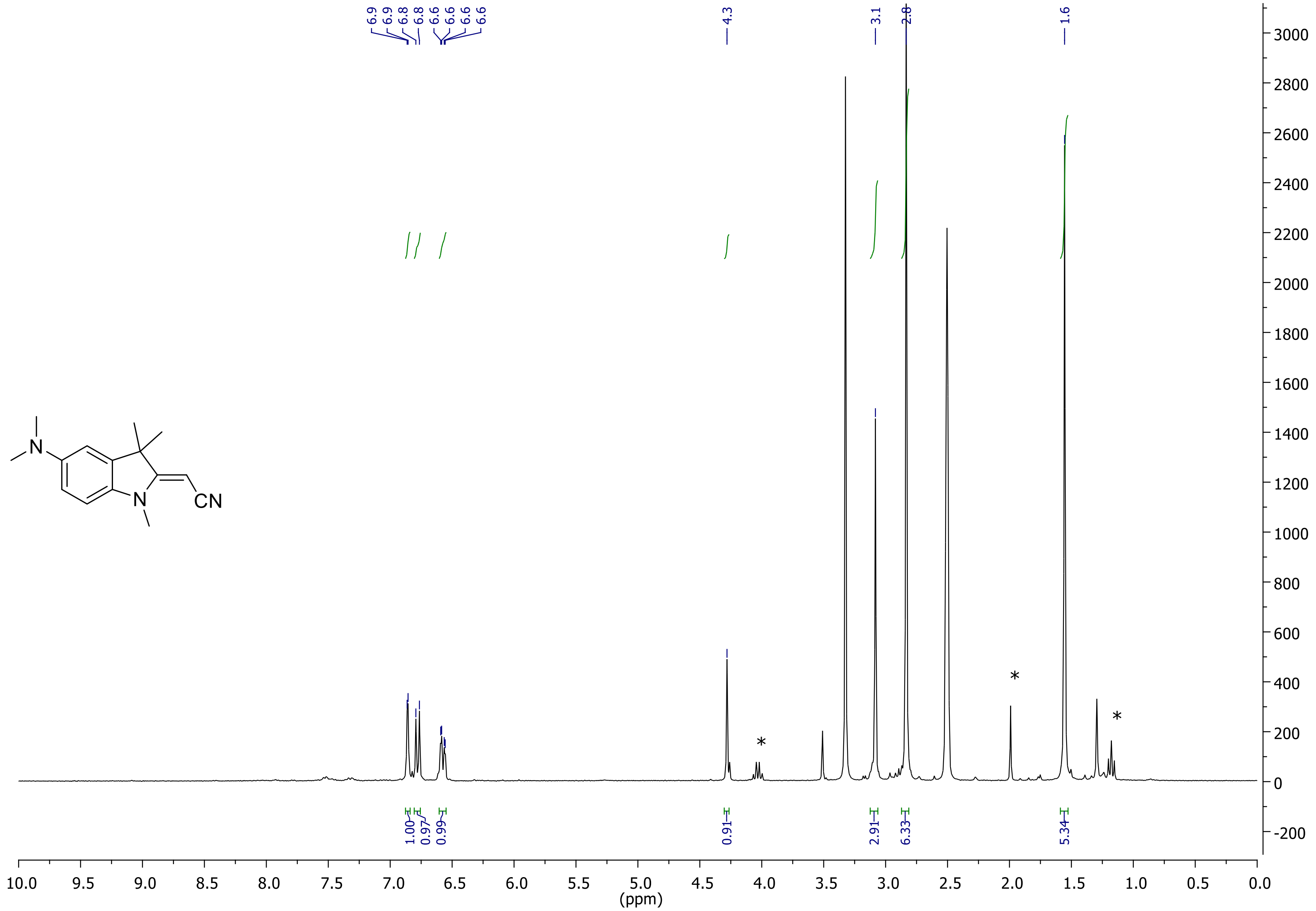

Figure S5. ^{1}H NMR (300 MHz, d_6-DMSO): **1e** (* ethyl acetate).

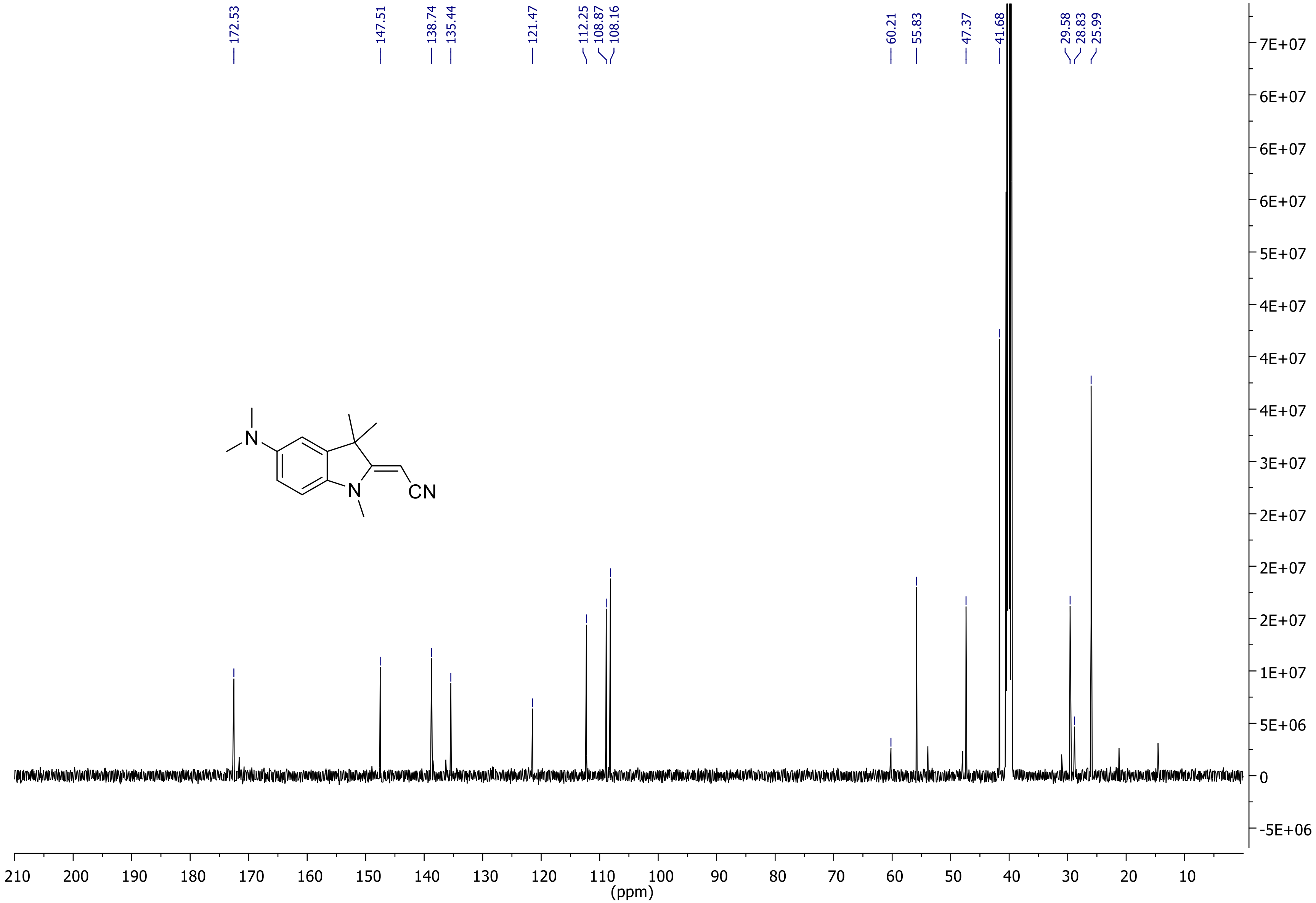

Figure S6. $^{13}C\{^1H\}$ NMR (126 MHz, d_6-DMSO): **1e**.

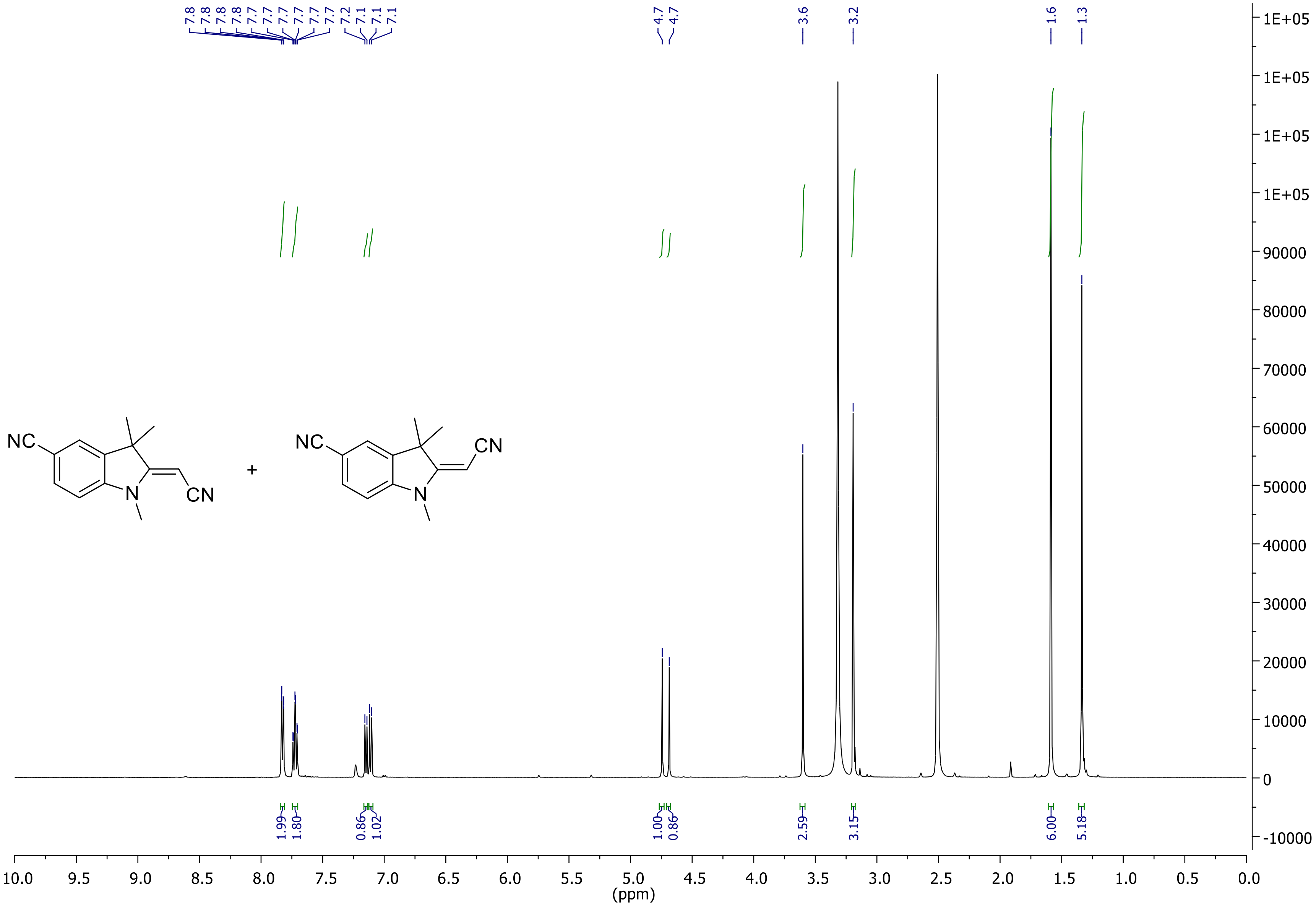

Figure S7. ^{1}H NMR (500 MHz, d_6-DMSO): **1f**.

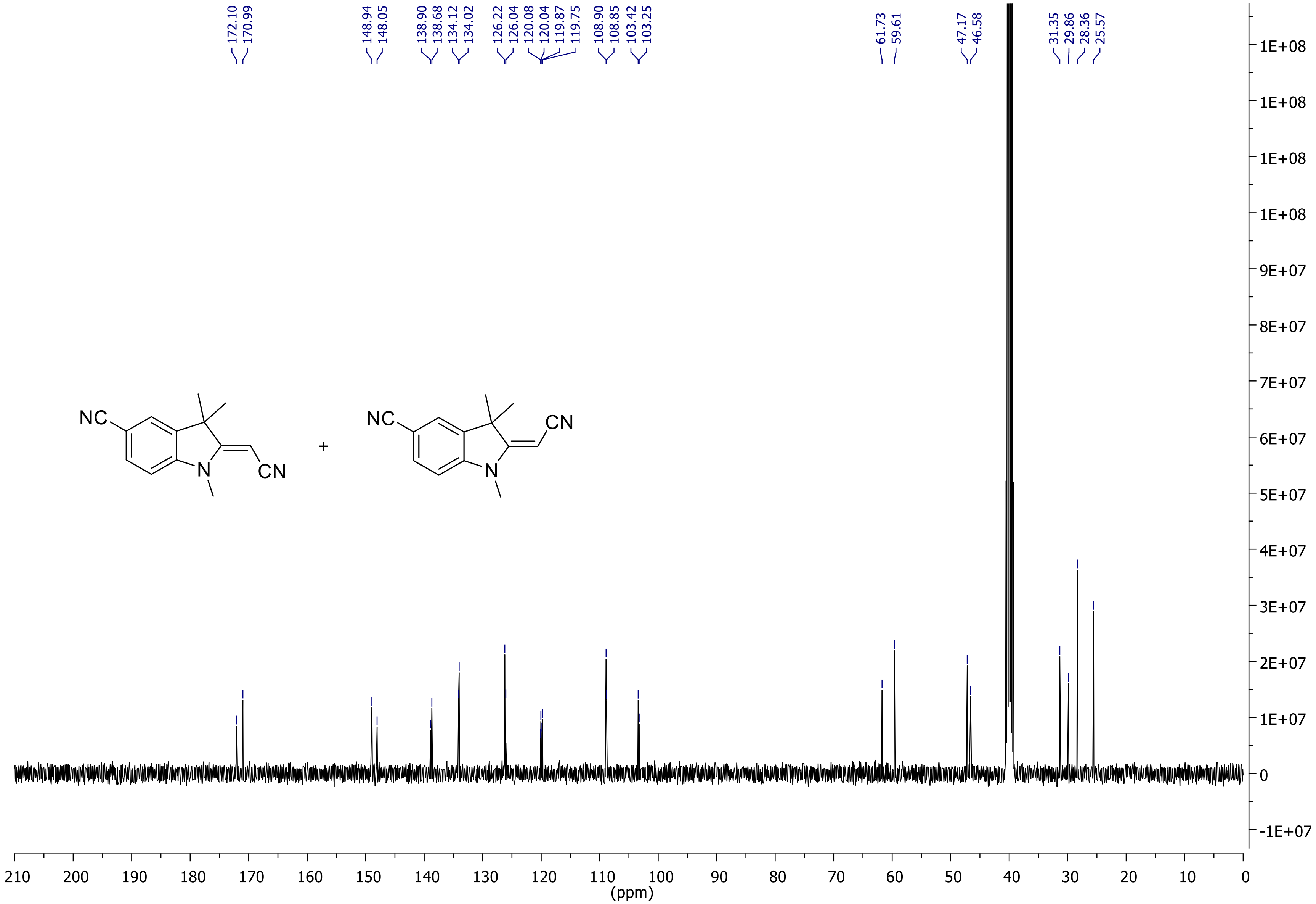

Figure S8. $^{13}C\{^{1}H\}$ NMR (126 MHz, d_6-DMSO): **1f**.

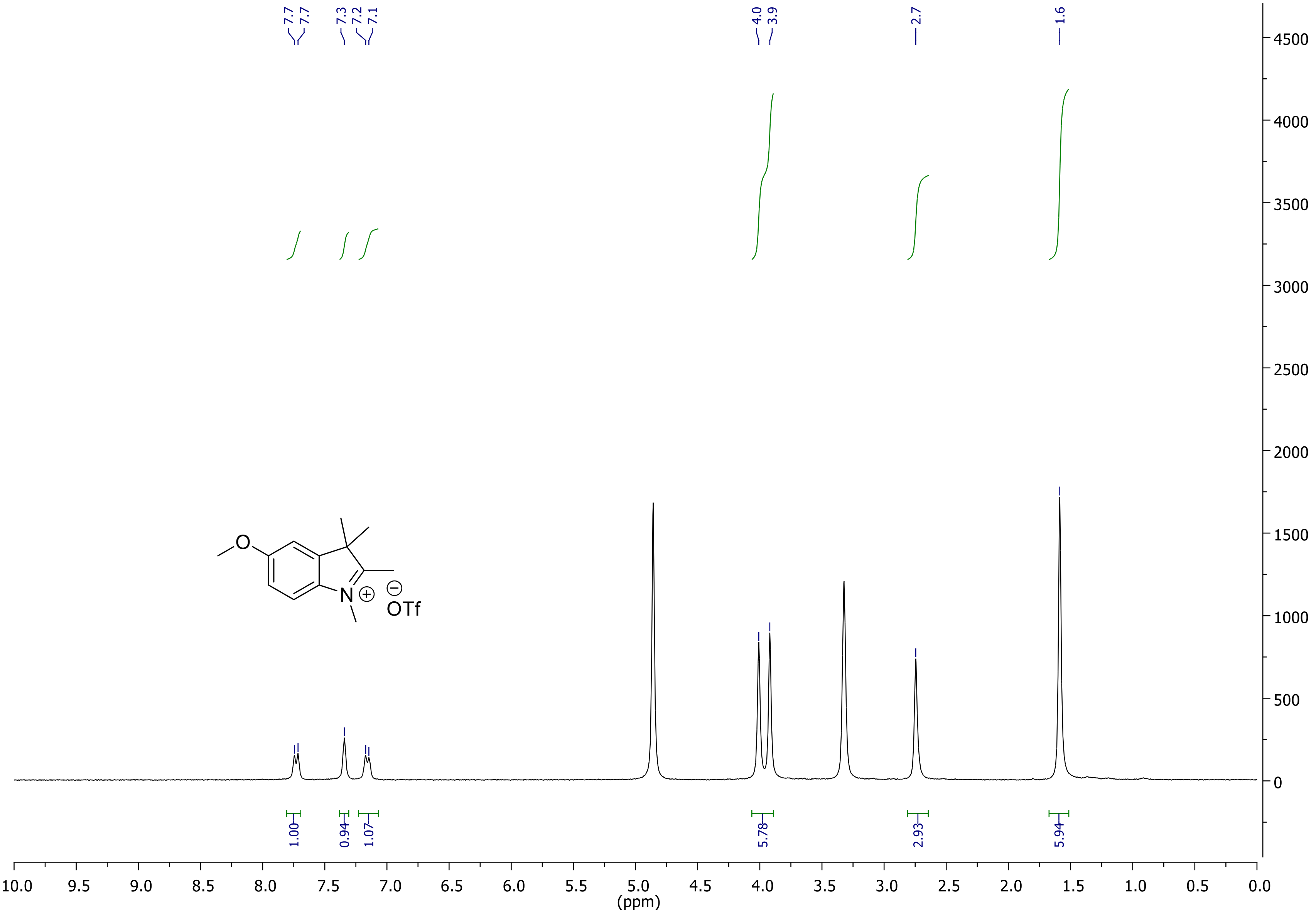

Figure S9. ^{1}H NMR (300 MHz, d_4–CD_3OD): **3d**.

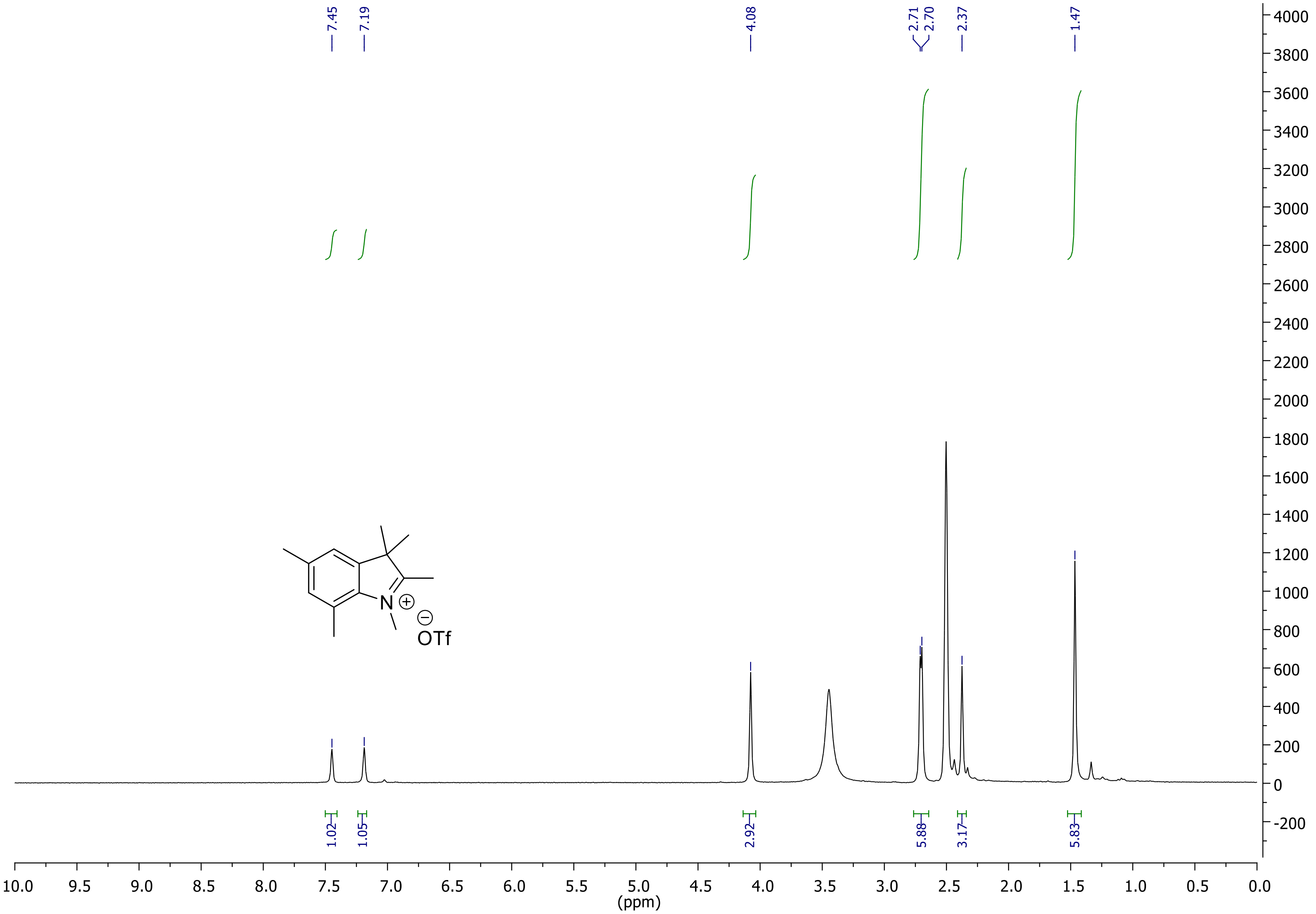

Figure S10. ^{1}H NMR (300 MHz, d_6-DMSO): **3e**.

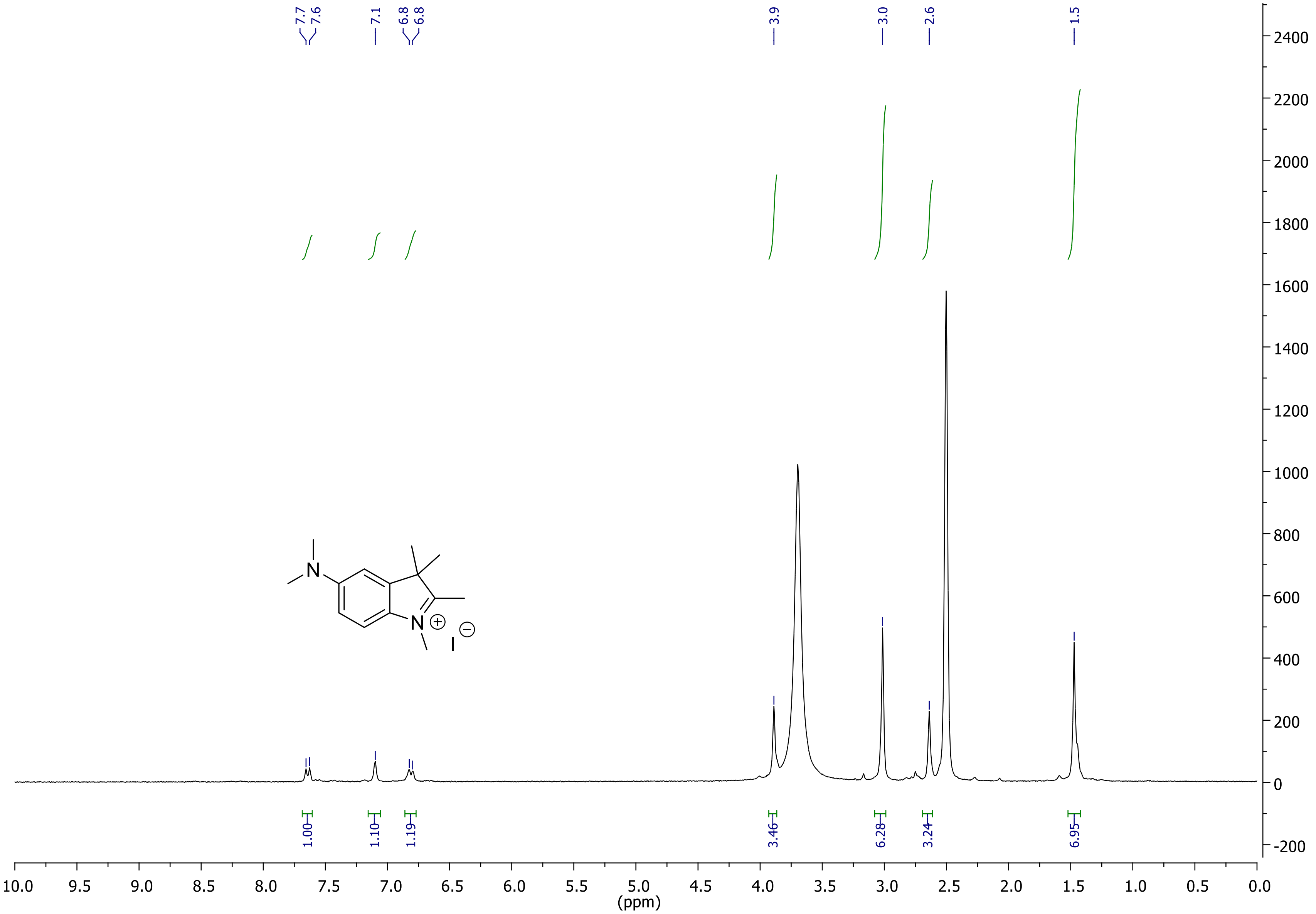

Figure S11. ^{1}H NMR (300 MHz, d_6-DMSO): **3f**.

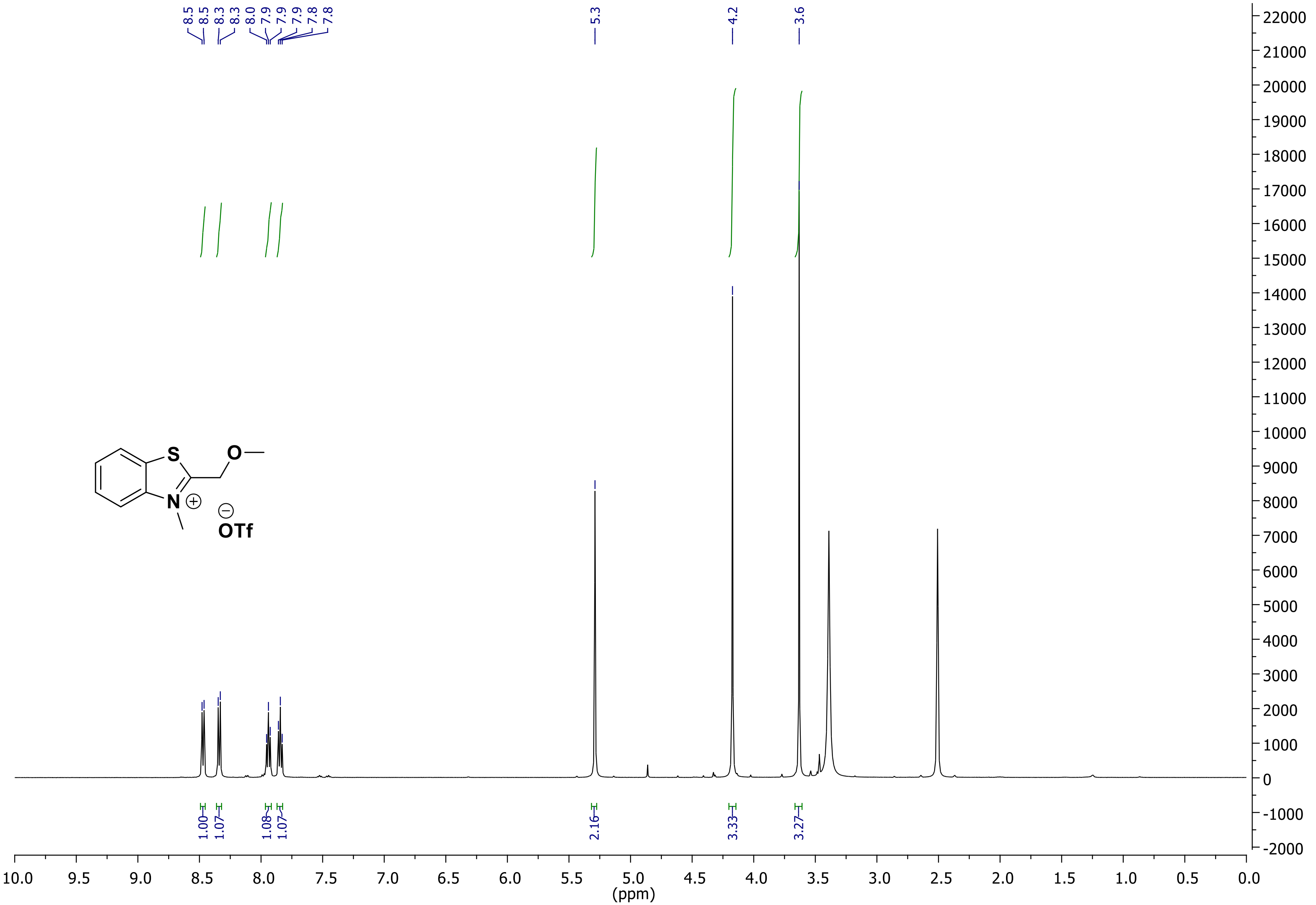

Figure S12. ^{1}H NMR (300 MHz, d_6-DMSO): **4a**.

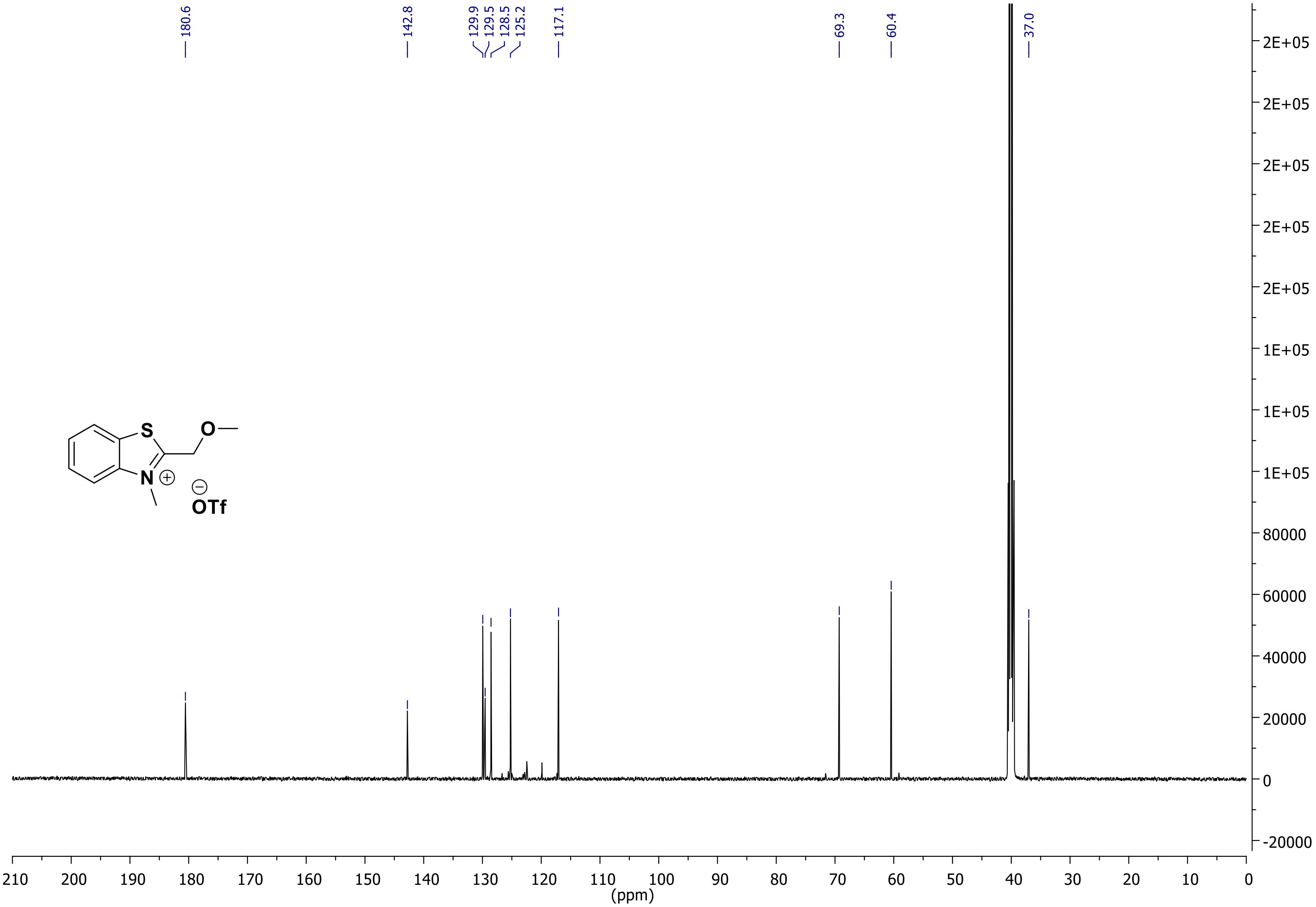

Figure 13. $^{13}C\{^1H\}$ NMR (126 MHz, d_6-DMSO): **4a**.

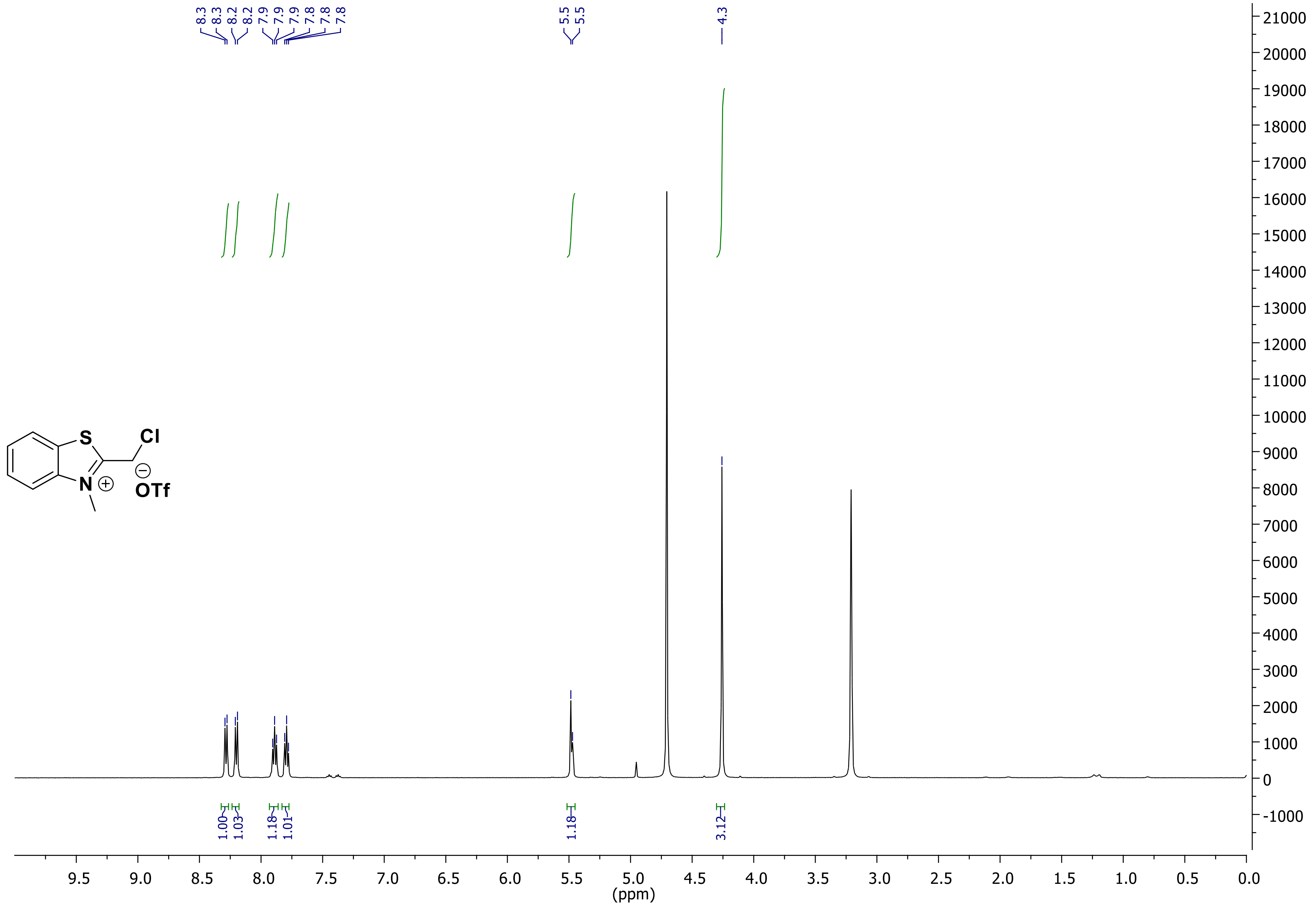

Figure S14. ^{1}H NMR (500 MHz, d_4–CD_3OD): **4b**.

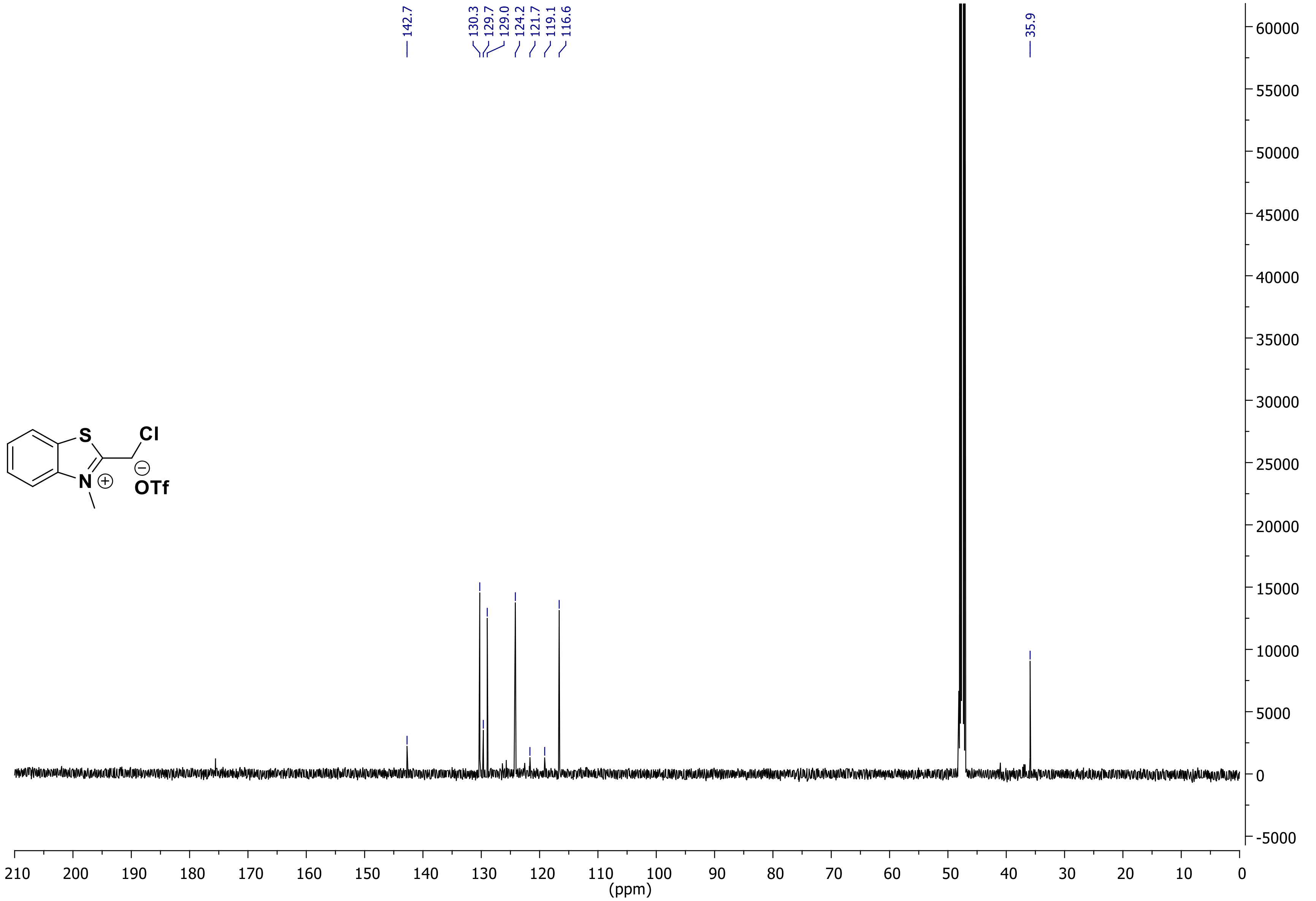

Figure S15. $^{13}C\{^{1}H\}$ NMR (126 MHz, d_4–CD_3OD): **4b**.

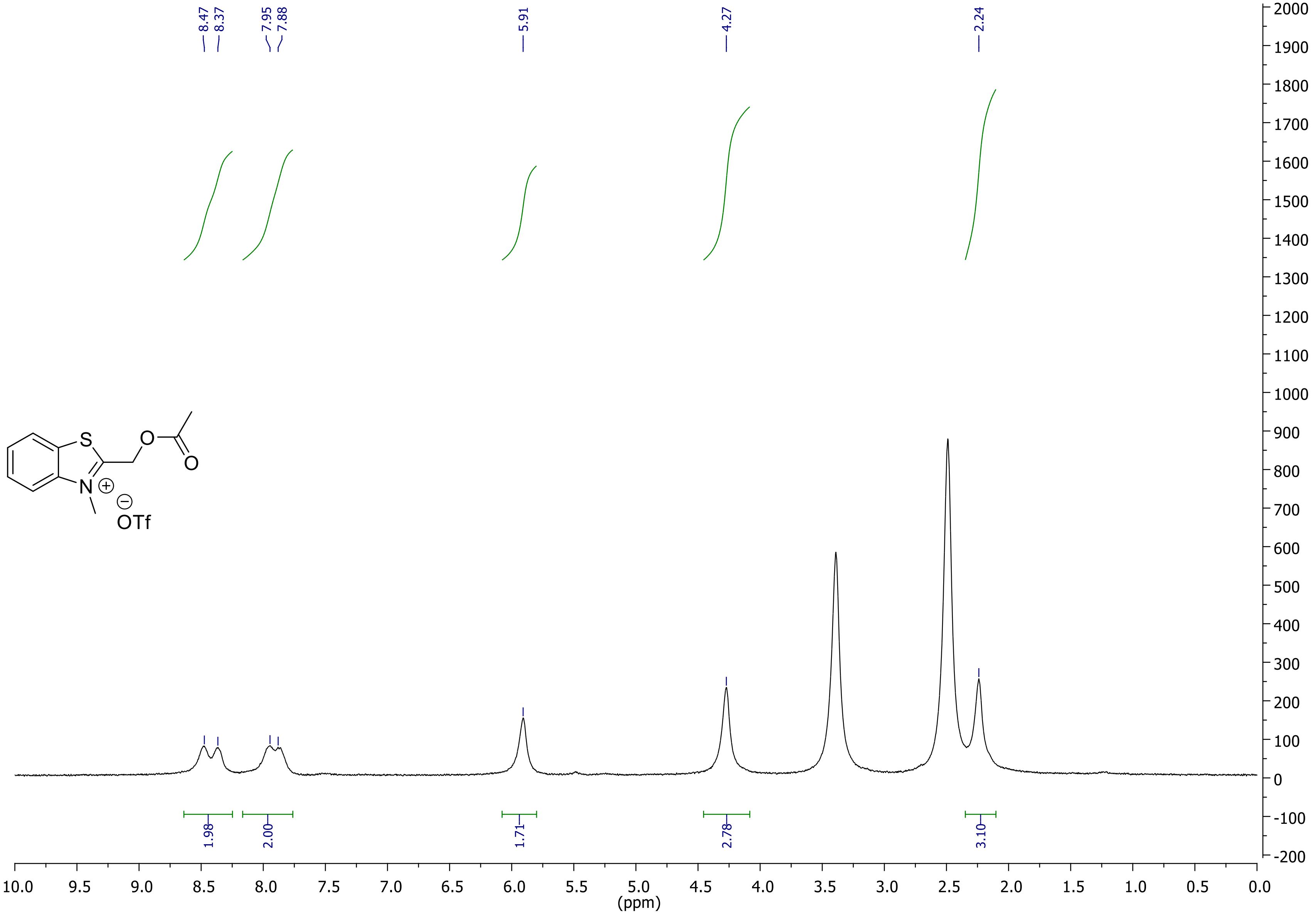

Figure S16. ^{1}H NMR (300 MHz, d_6-DMSO): **4e**.

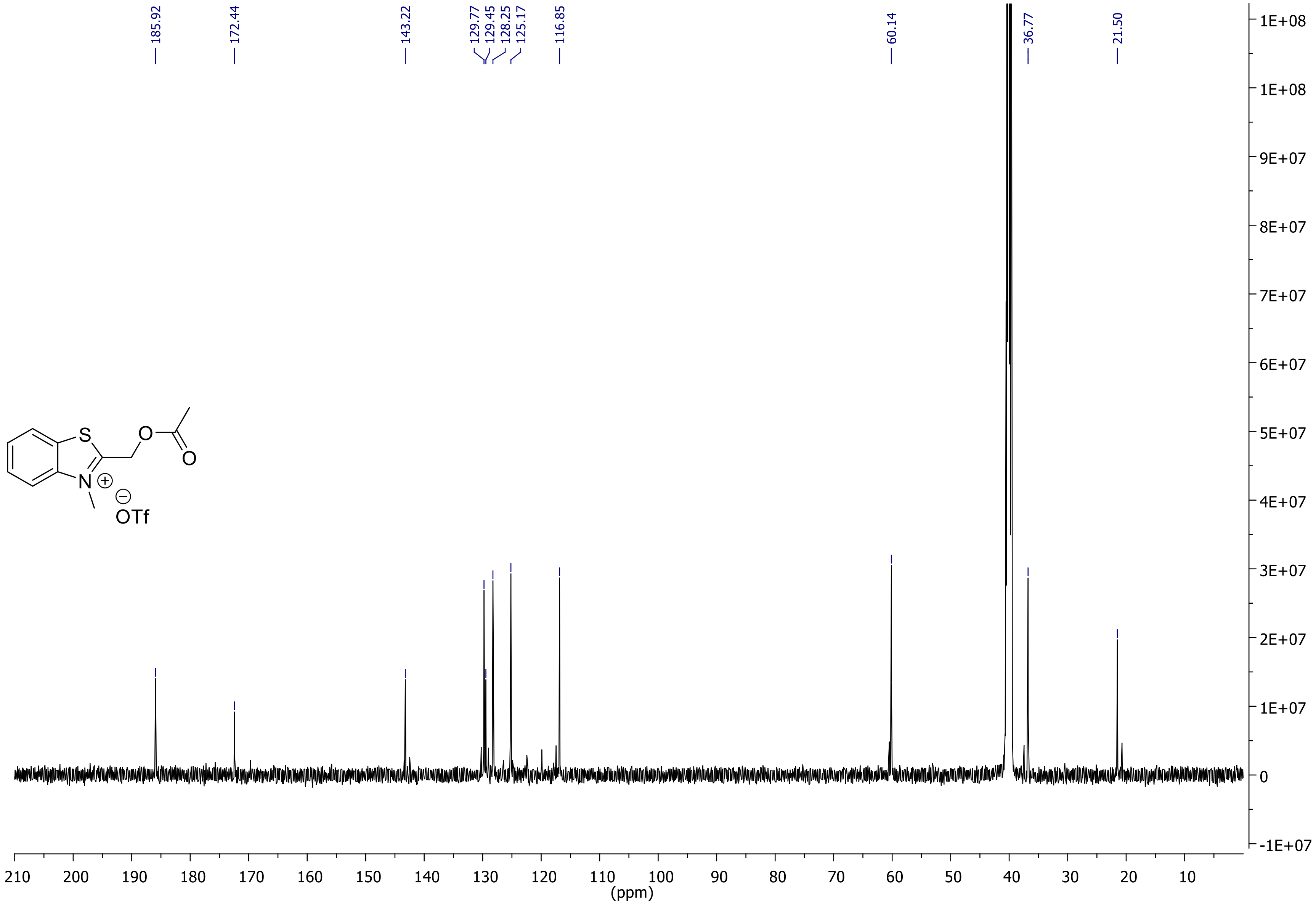

Figure S17. $^{13}C\{^{1}H\}$ NMR (300 MHz, d_6-DMSO): **4e**.

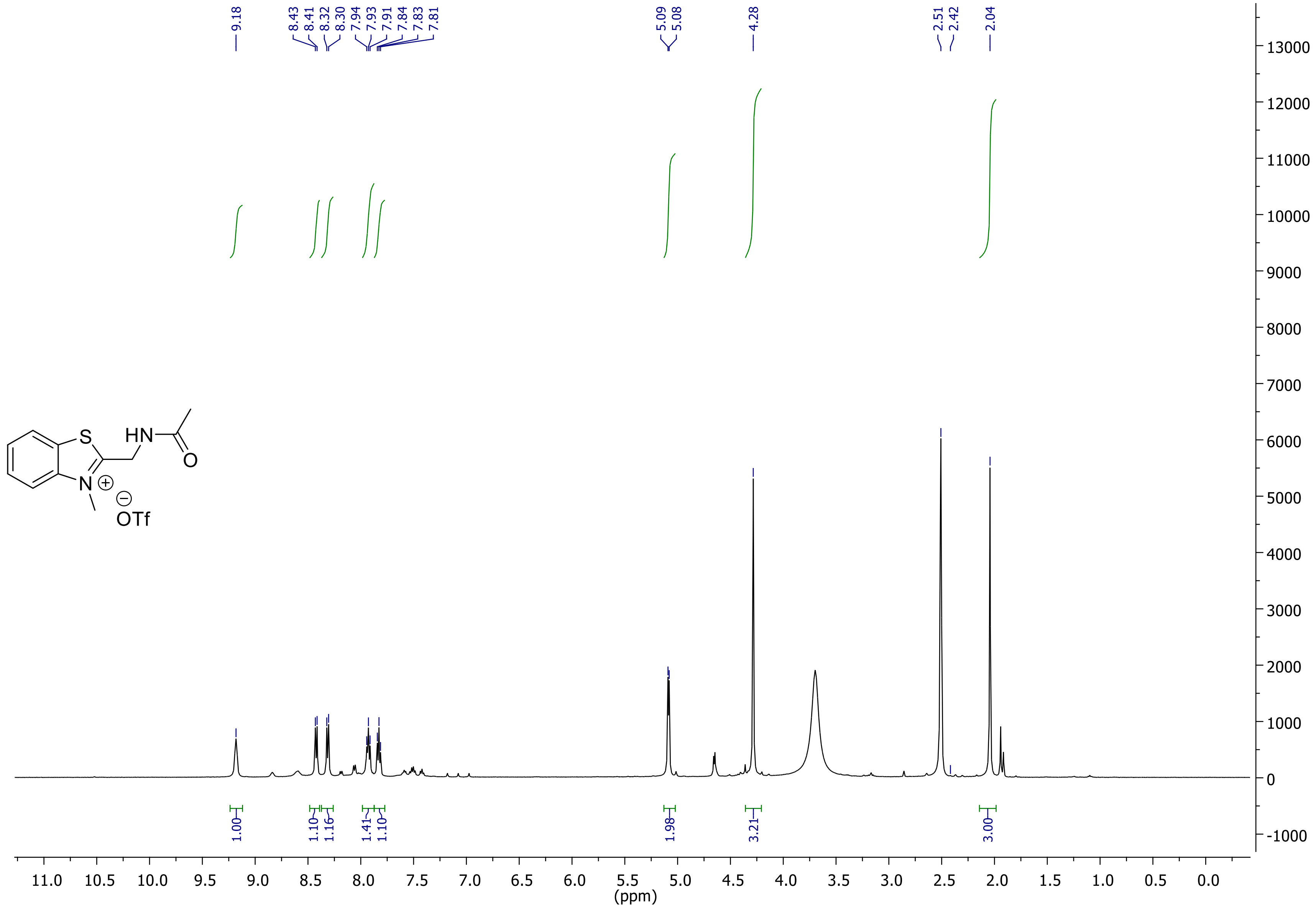

Figure S18. ^{1}H NMR (300 MHz, d_6-DMSO): **4g**.

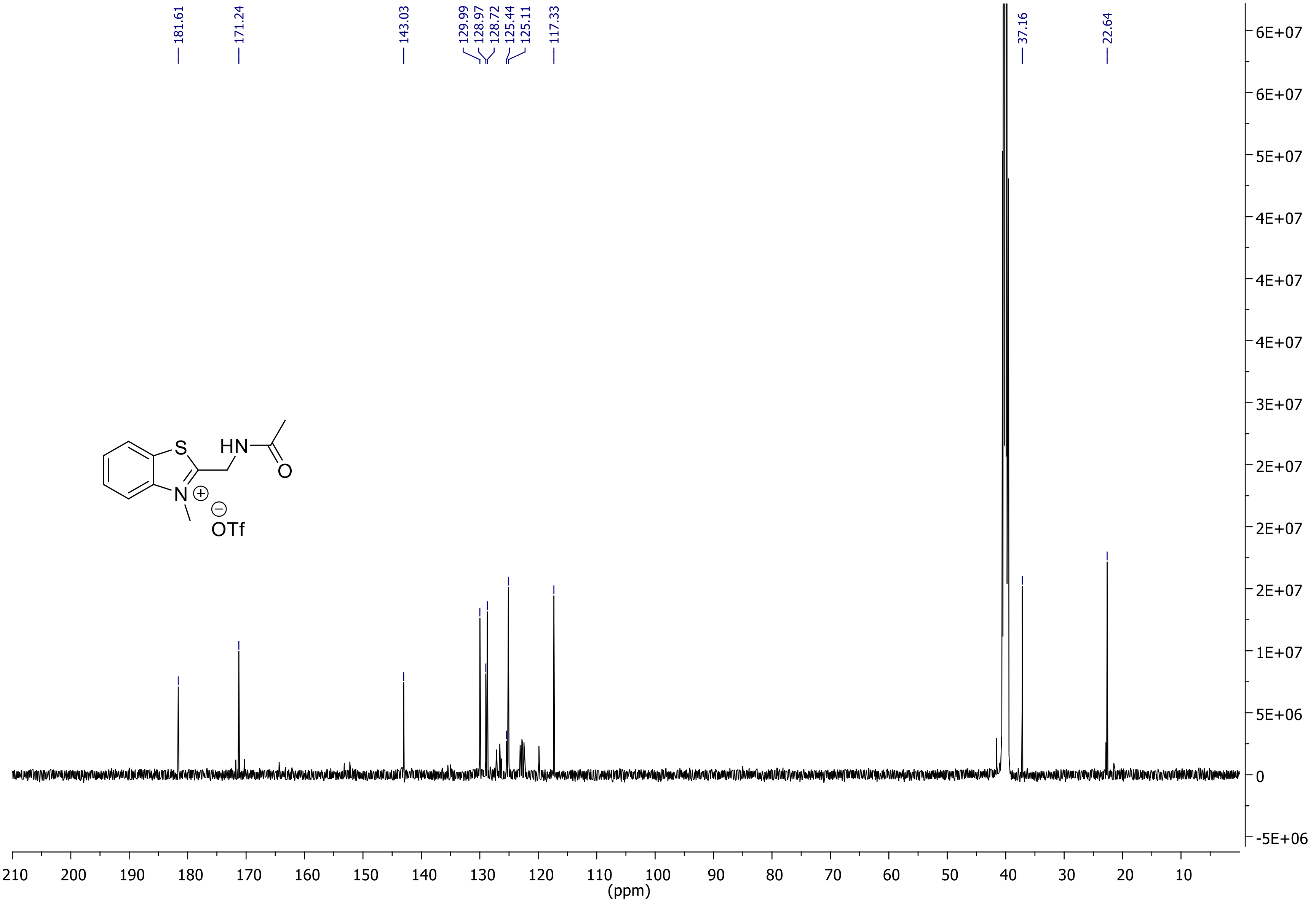

Figure 19. $^{13}C\{^{1}H\}$ NMR (126 MHz, d_6-DMSO): **4g**.

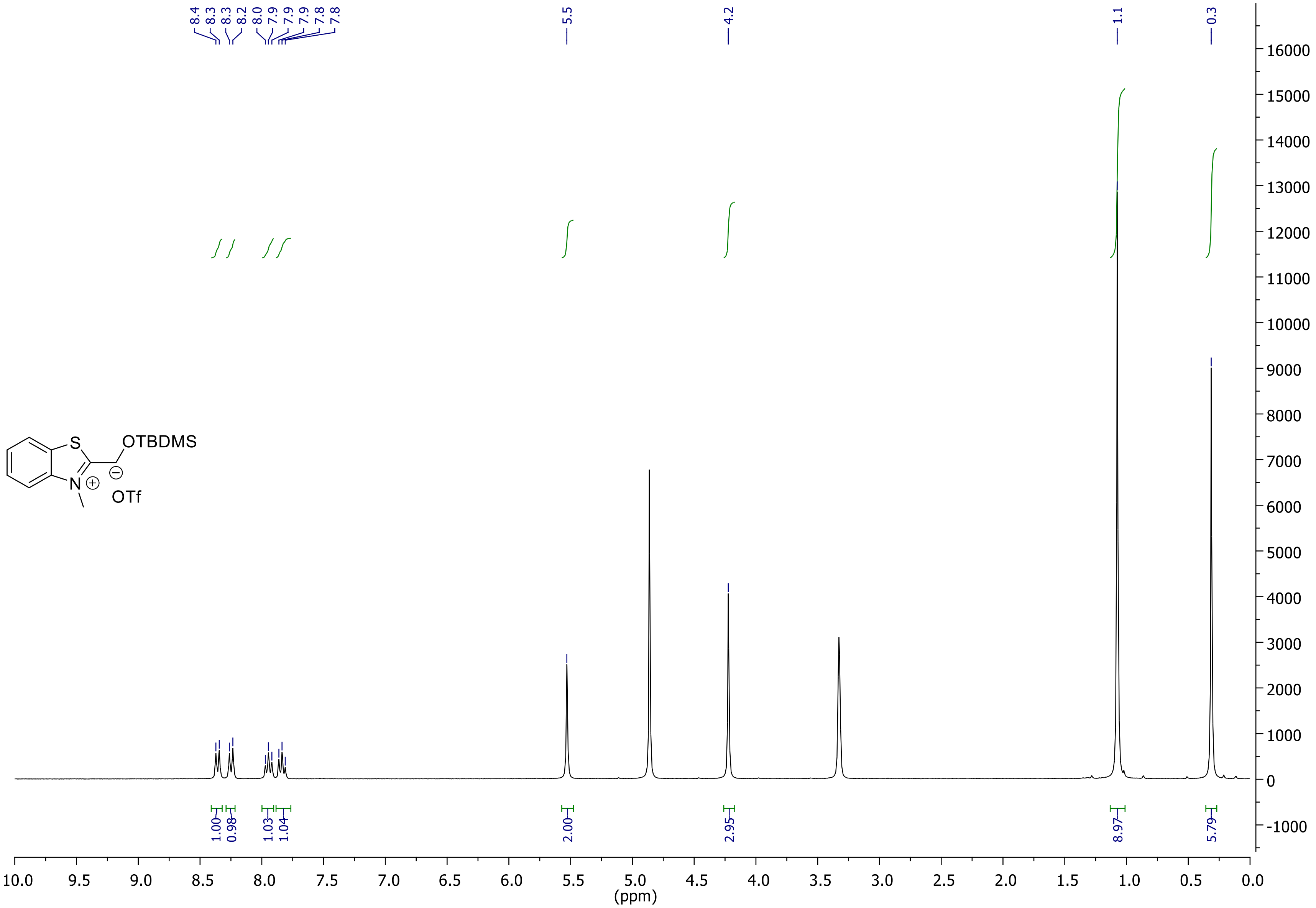

Figure S20. ^{1}H NMR (300 MHz, d_4–CD_3OD): **4h**.

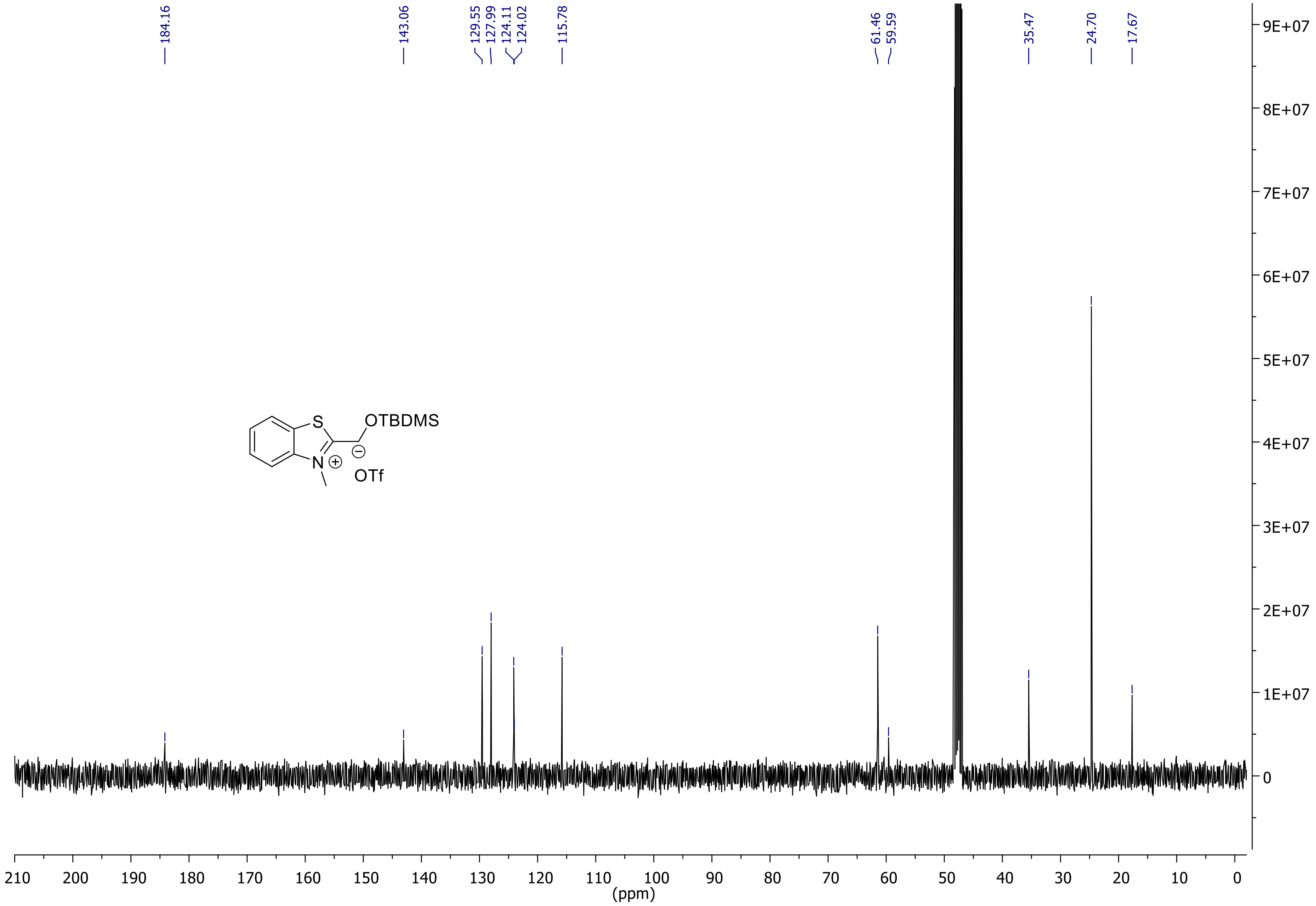

Figure S21. $^{13}C\{^{1}H\}$ NMR (126 MHz, d_4–CD_3OD): **4h**.

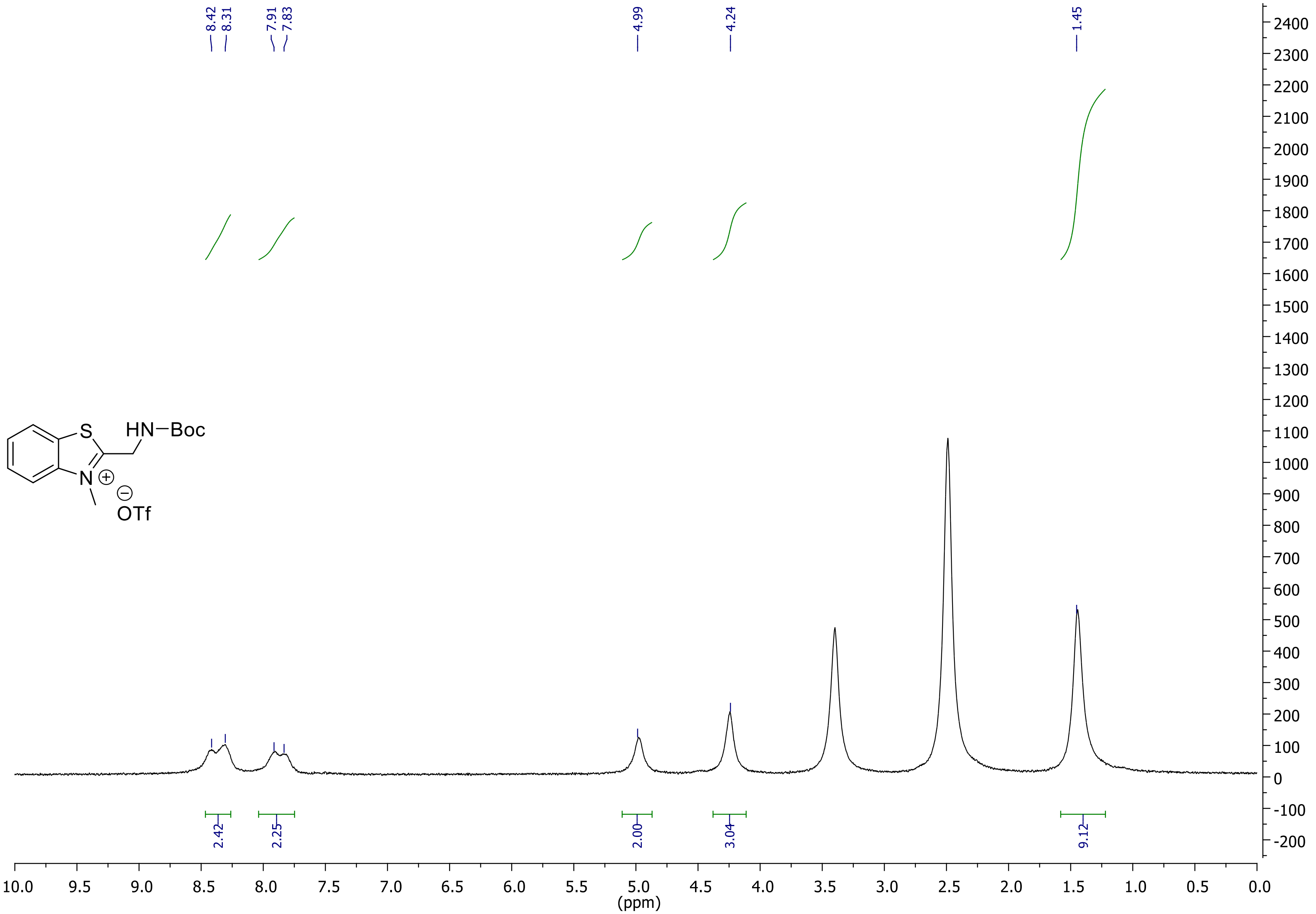

Figure S22. ^{1}H NMR (300 MHz, d_6-DMSO): **4i**.

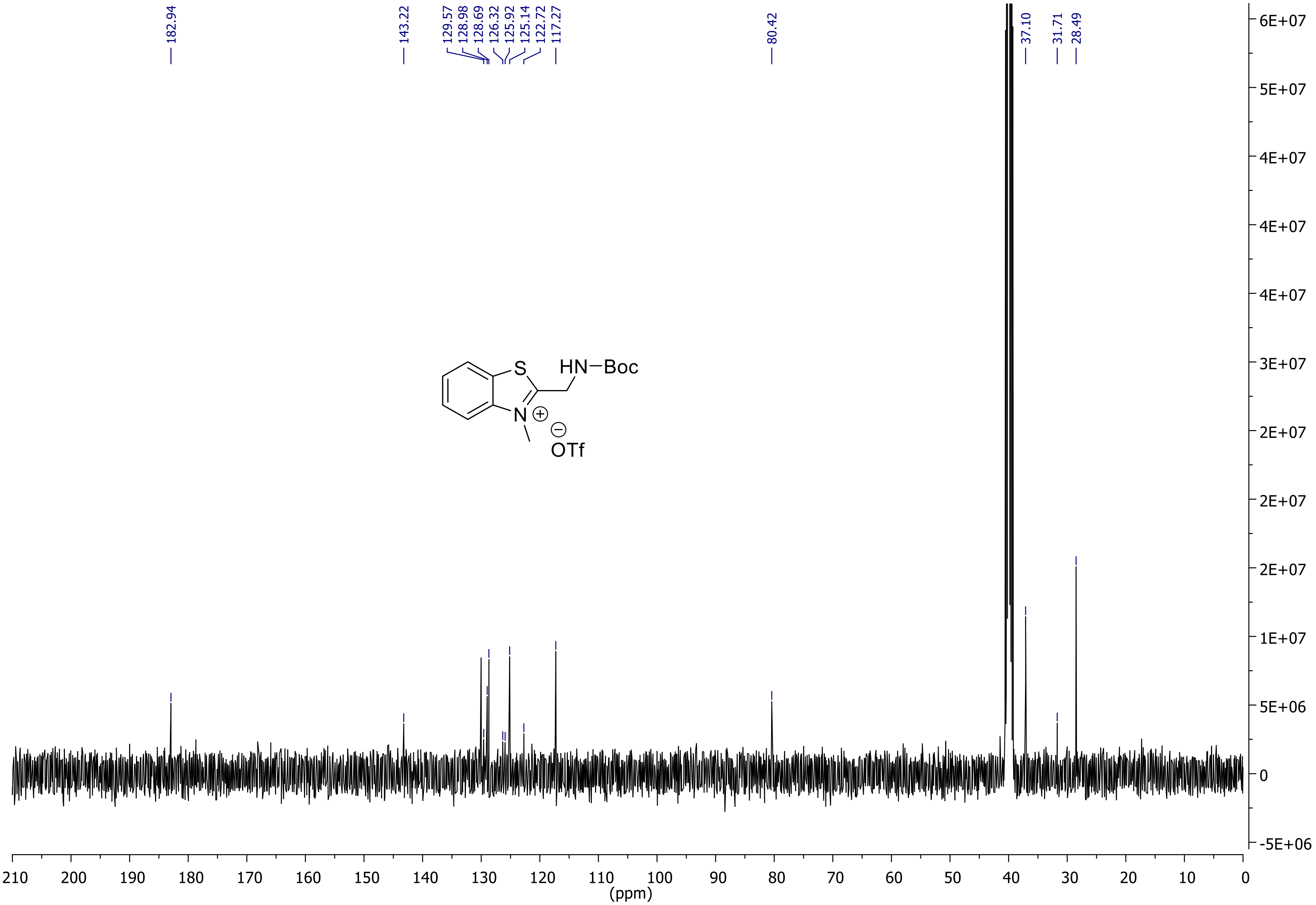

Figure S23. $^{13}C\{^{1}H\}$ NMR (126 MHz, d_6-DMSO): **4i**.

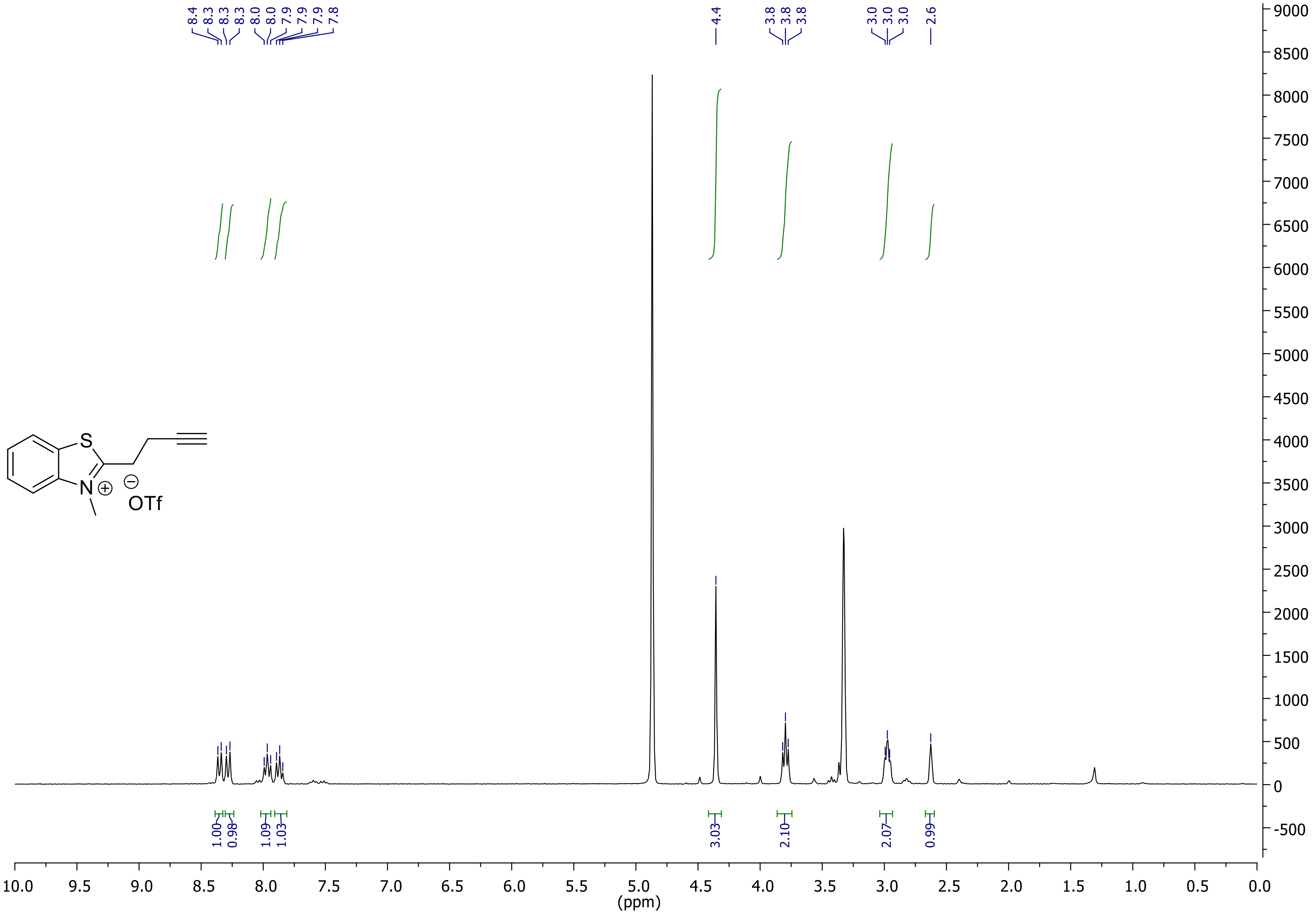

Figure S24. ^{1}H NMR (300 MHz, d_4–CD_3OD): **4j**.

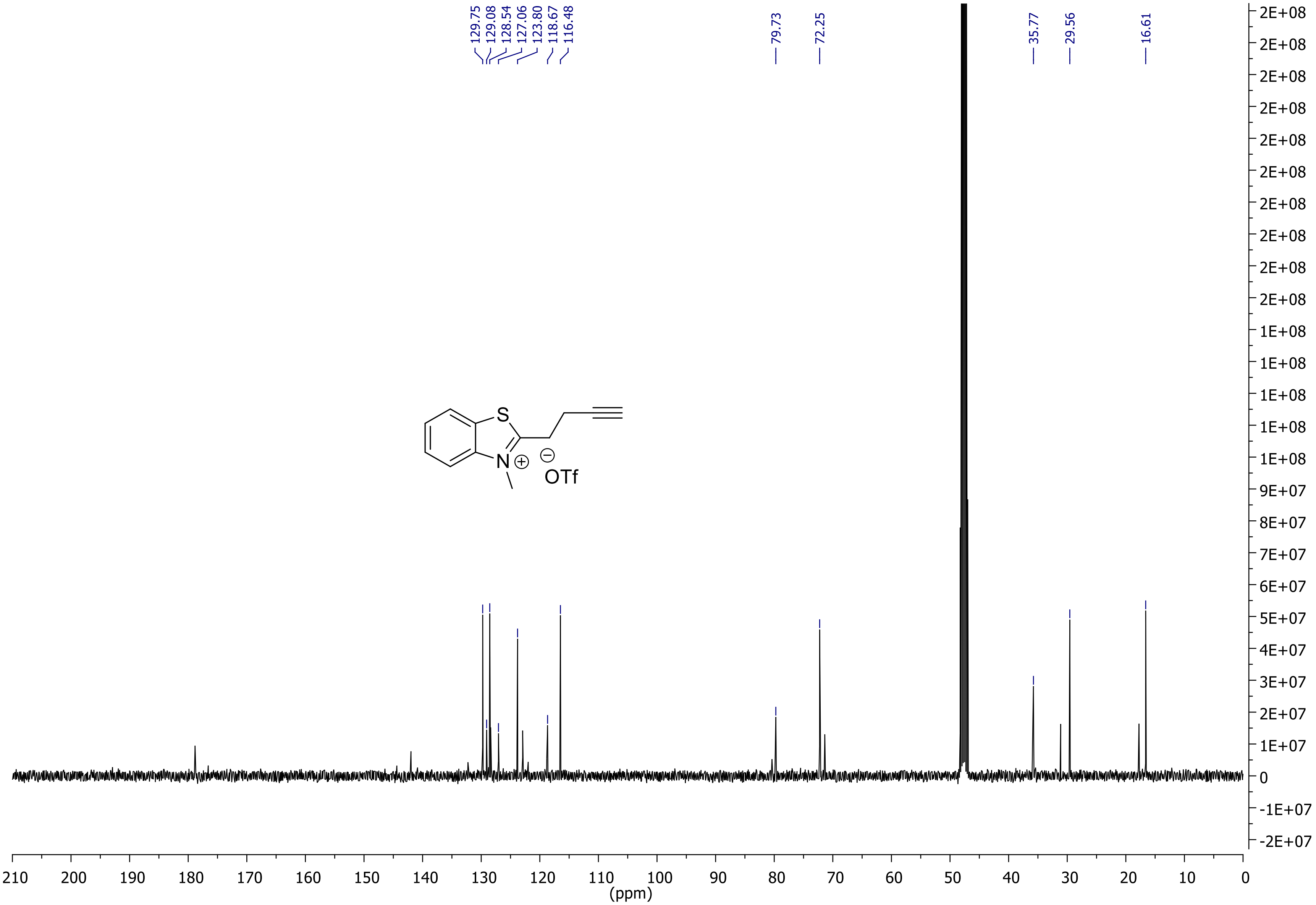

Figure S25. $^{13}C\{^{1}H\}$ NMR (126 MHz, d_4–CD_3OD): **4j**.

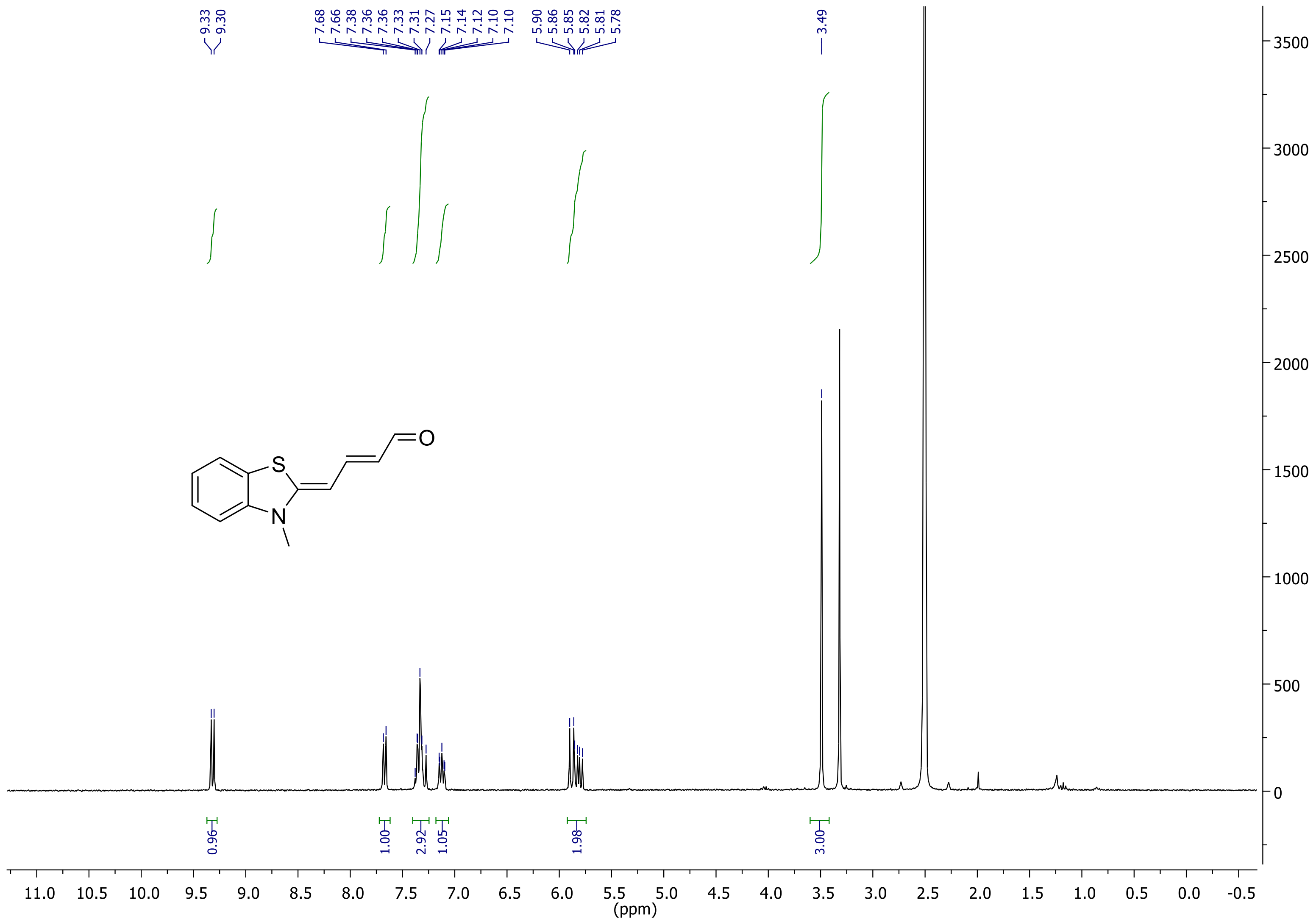

Figure S26. ^{1}H NMR (500 MHz, d_6-DMSO): **13**.

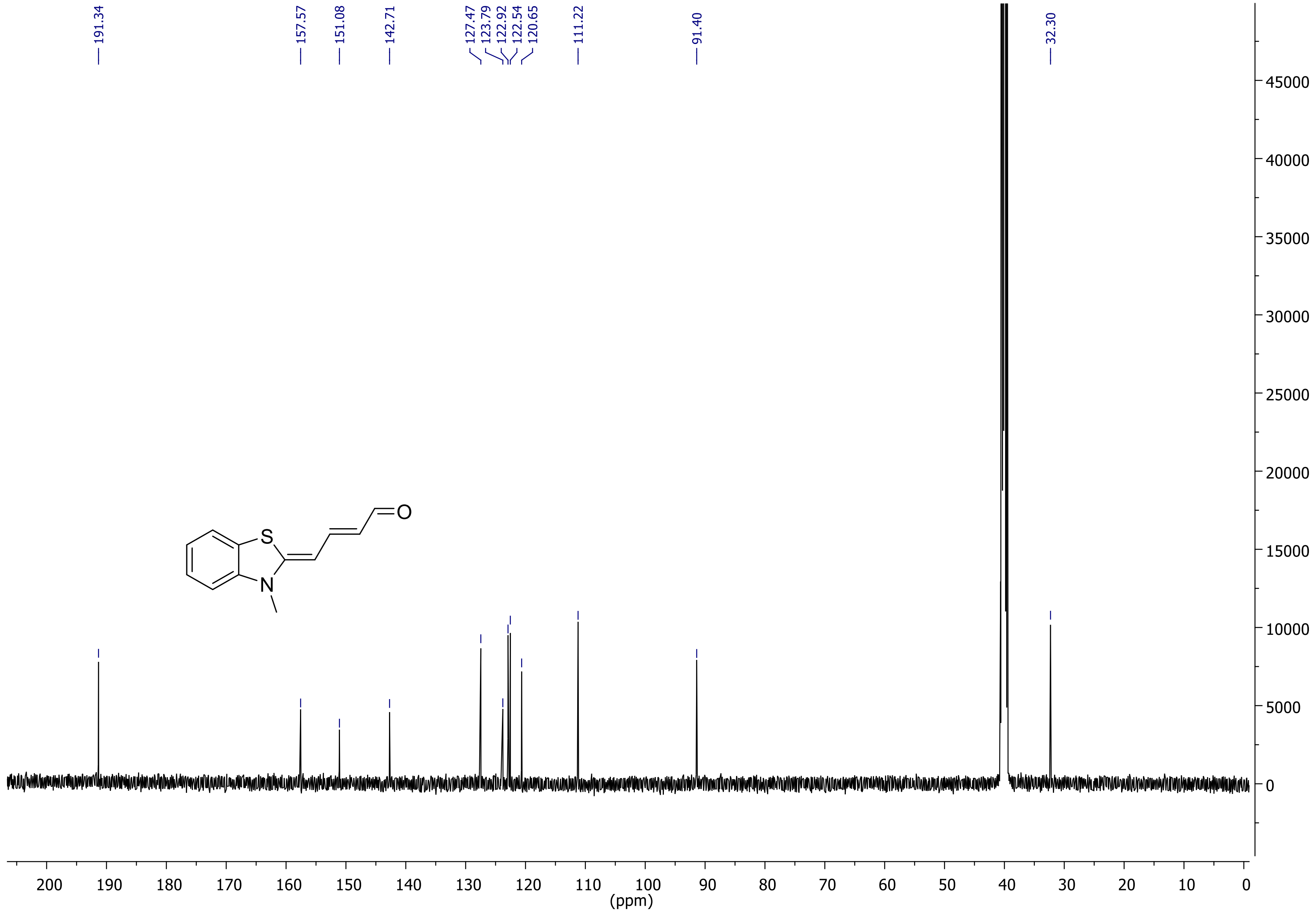

Figure S27. $^{13}C\{^1H\}$ NMR (126 MHz, d_6–DMSO): **13**.

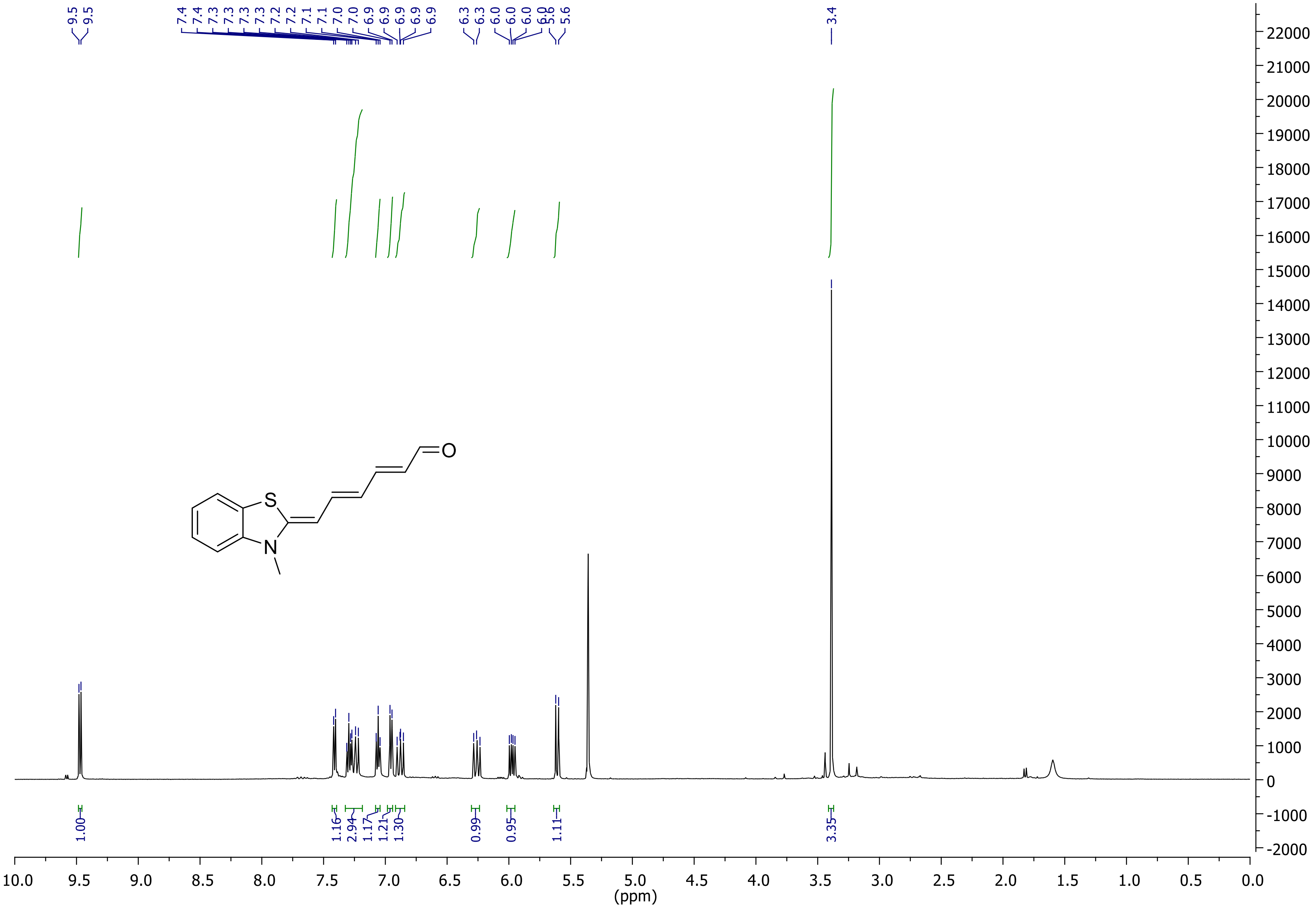

Figure S28. ^{1}H NMR (500 MHz, CD_2Cl_2): **15**.

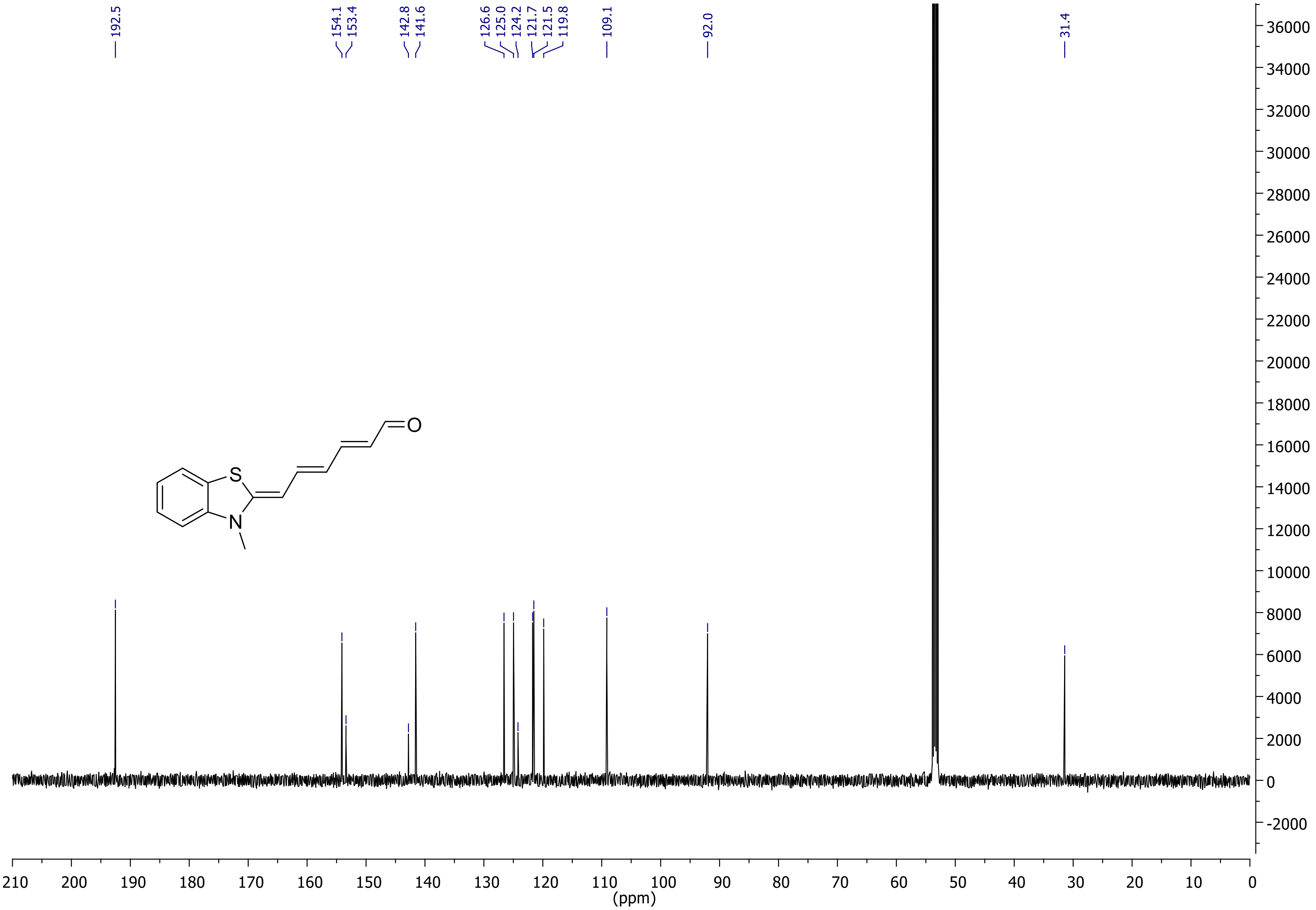

Figure S29. $^{13}C\{^{1}H\}$ NMR (126 MHz, CD_2Cl_2): **15**.

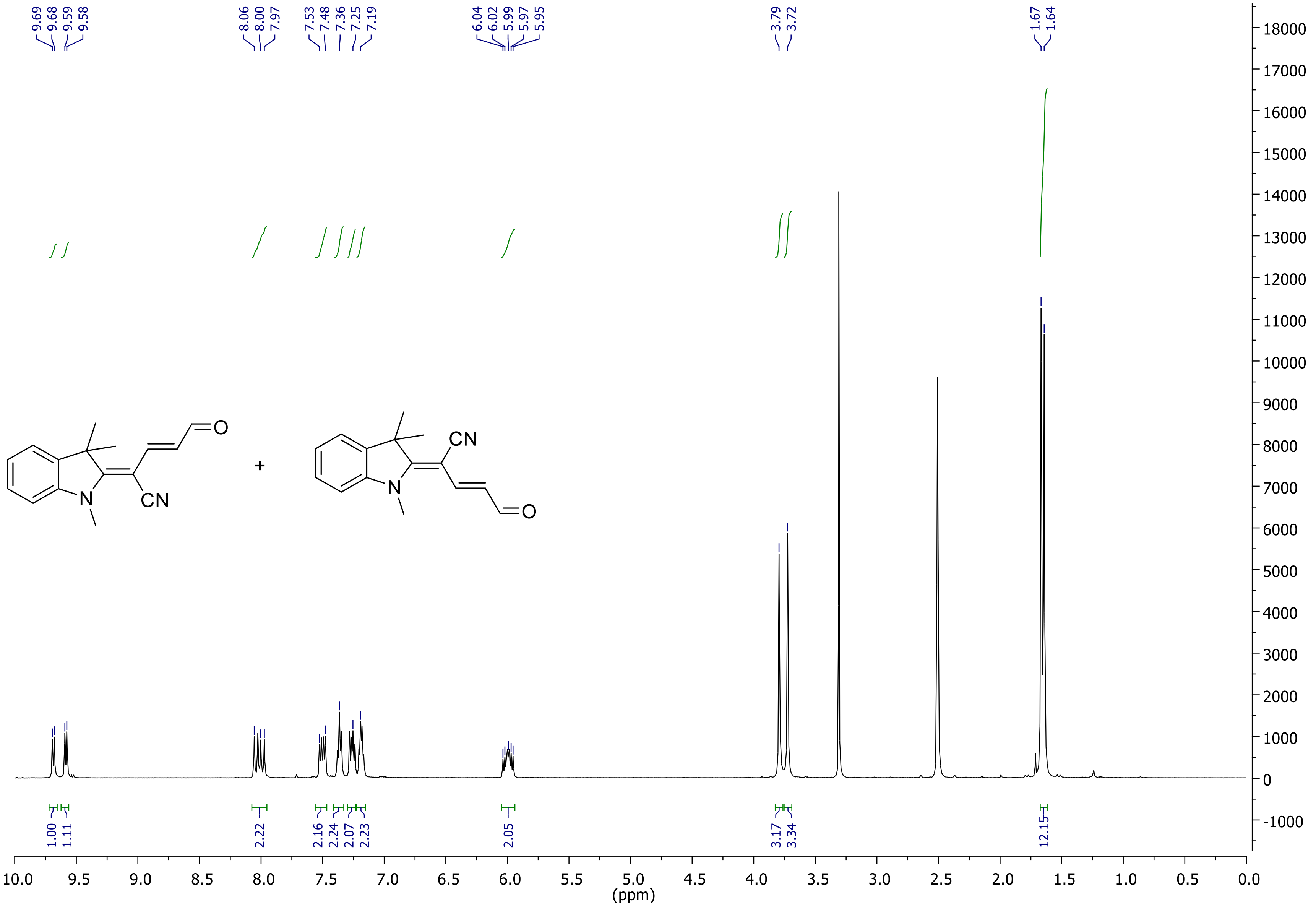

Figure S30. ^{1}H NMR (500 MHz, d_6-DMSO): **16**.

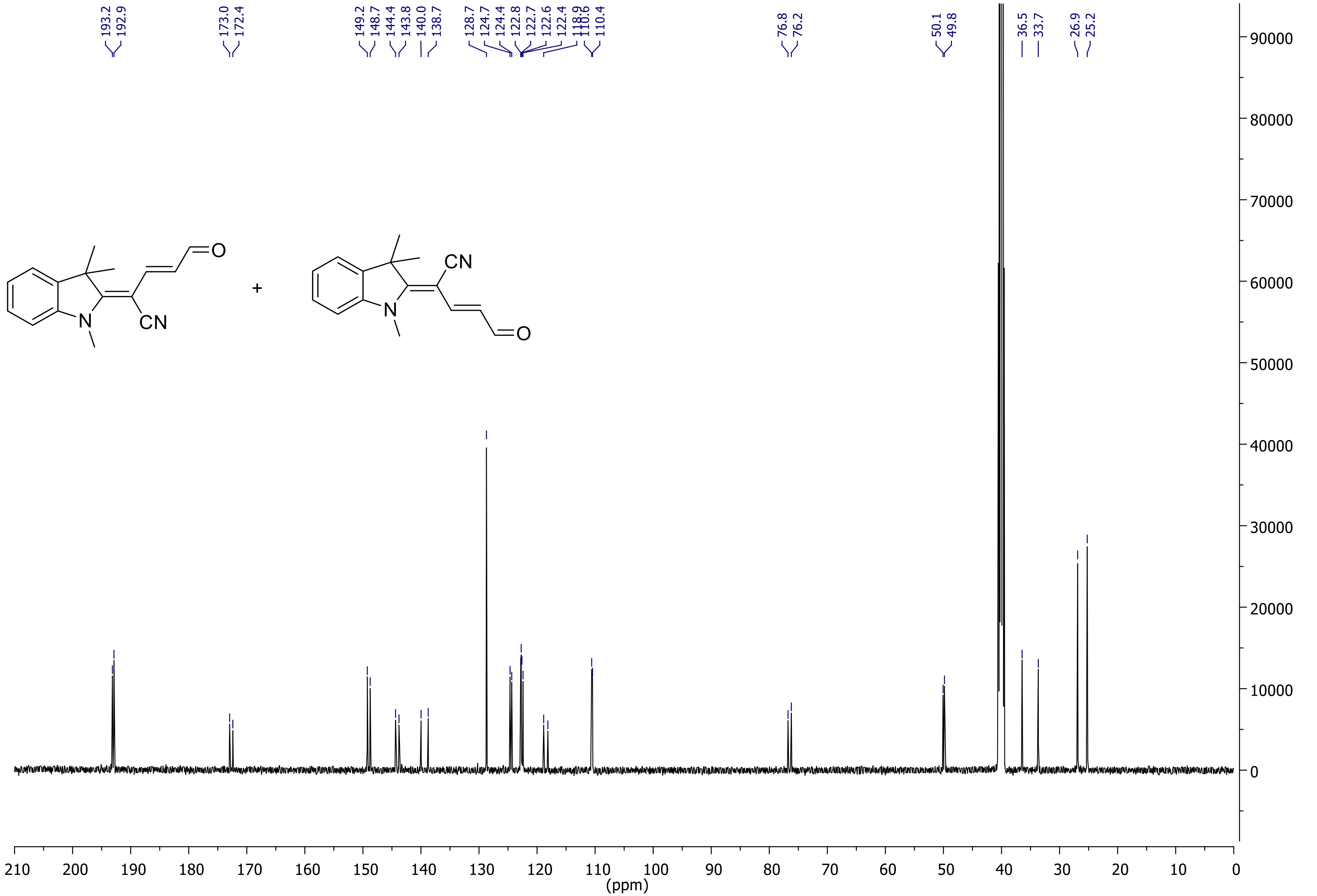

Figure S31. $^{13}C\{^{1}H\}$ NMR (126 MHz, d_6–DMSO): **16**.

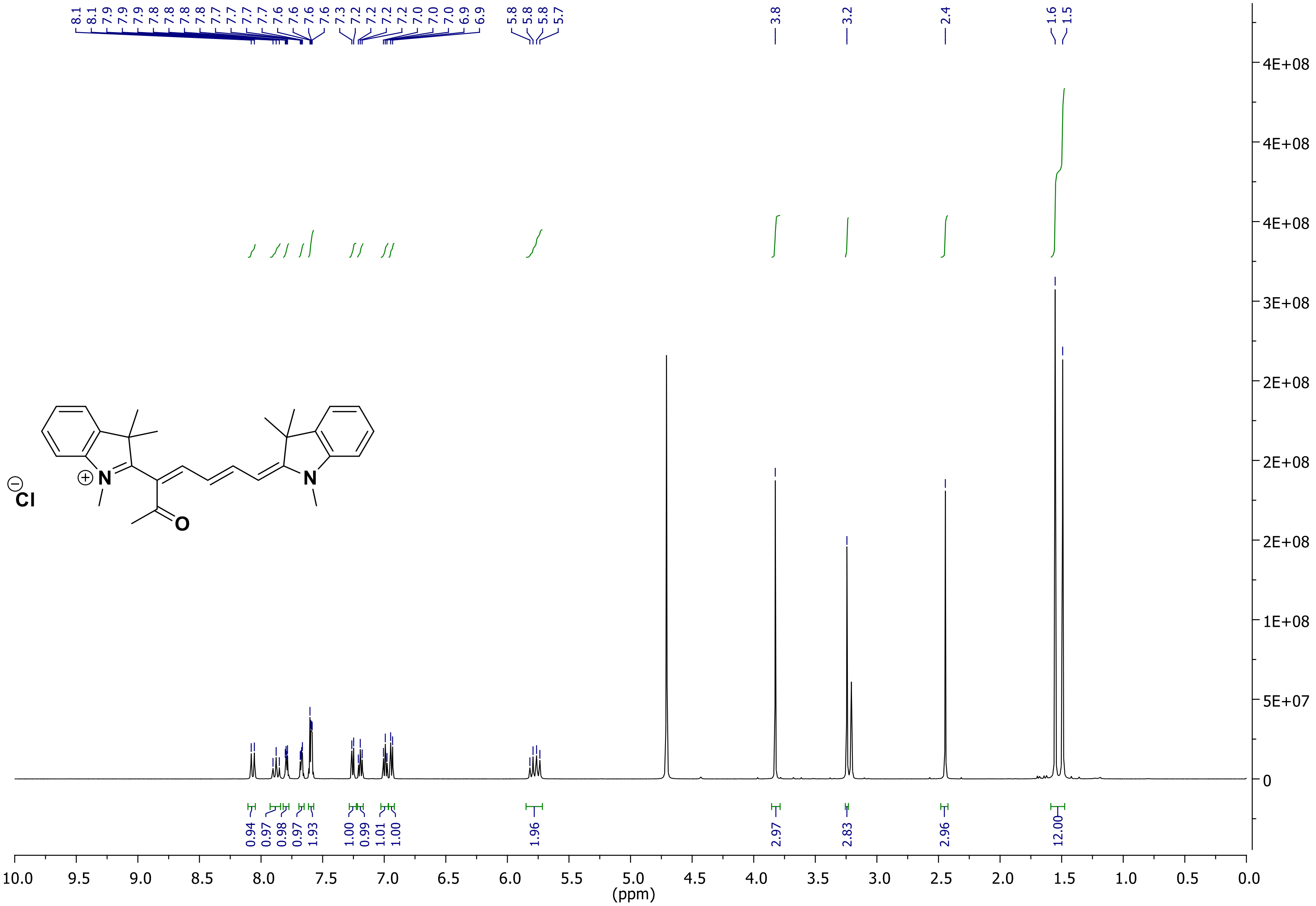

Figure S32. ^{1}H NMR (500 MHz, d_4–CD_3OD): **Cy5-3**.

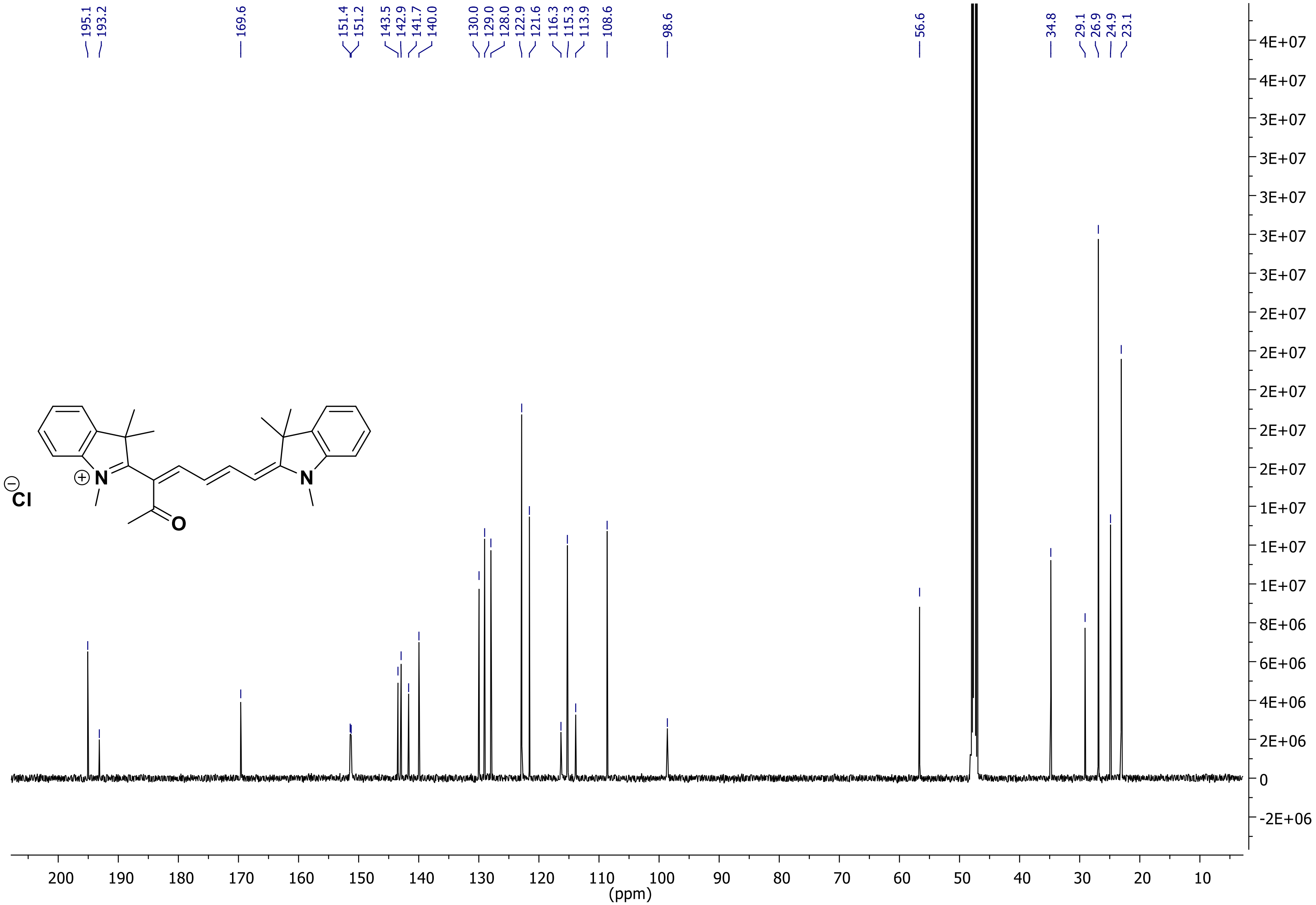

Figure S33. $^{13}C\{^1H\}$ NMR (126 MHz, d_4–CD_3OD): **Cy5-3**.

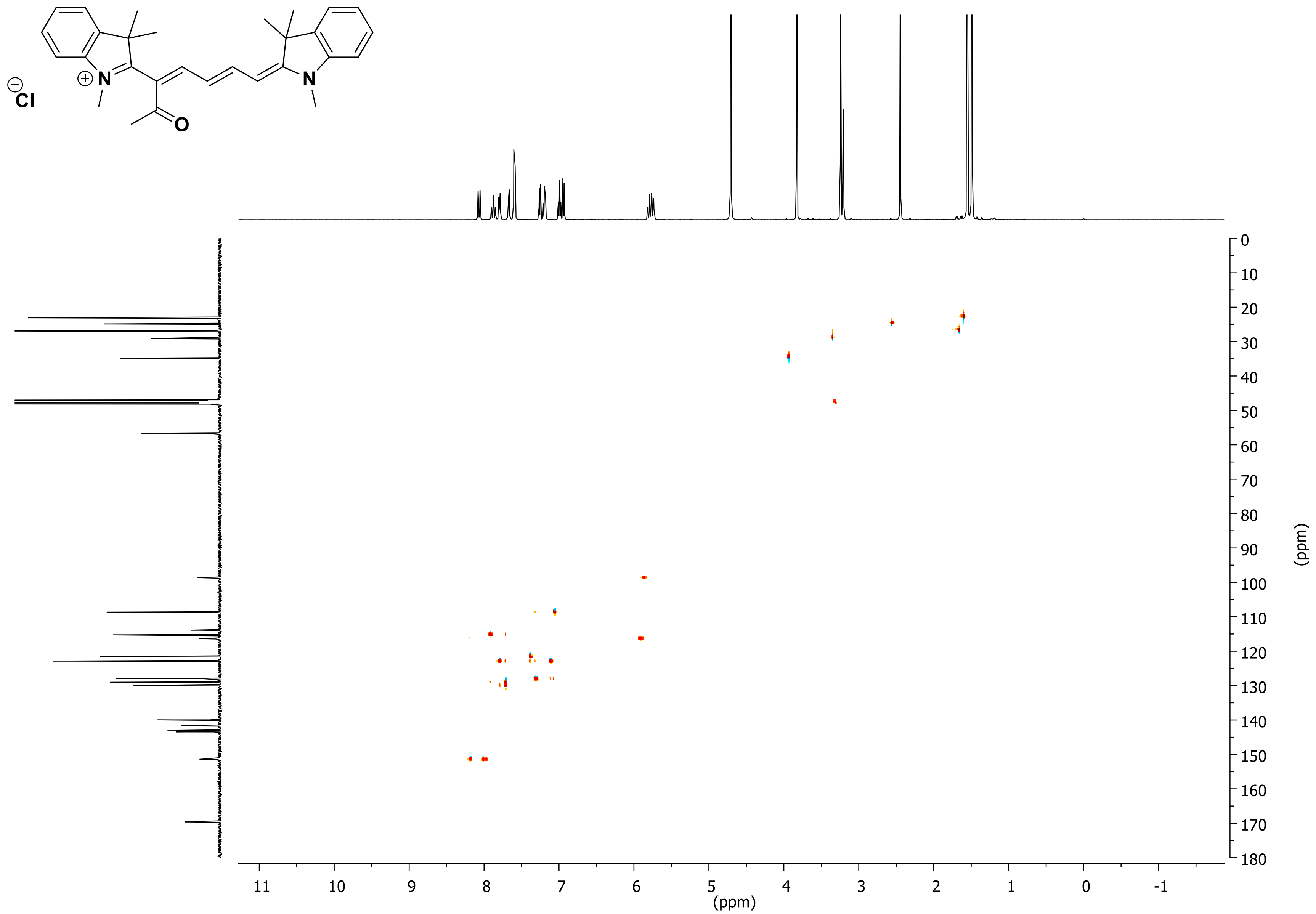

Figure S34. ^{1}H–$^{13}C\{^{1}H\}$ gHSQC (500 MHz, d_4–CD_3OD): **Cy5-3**.

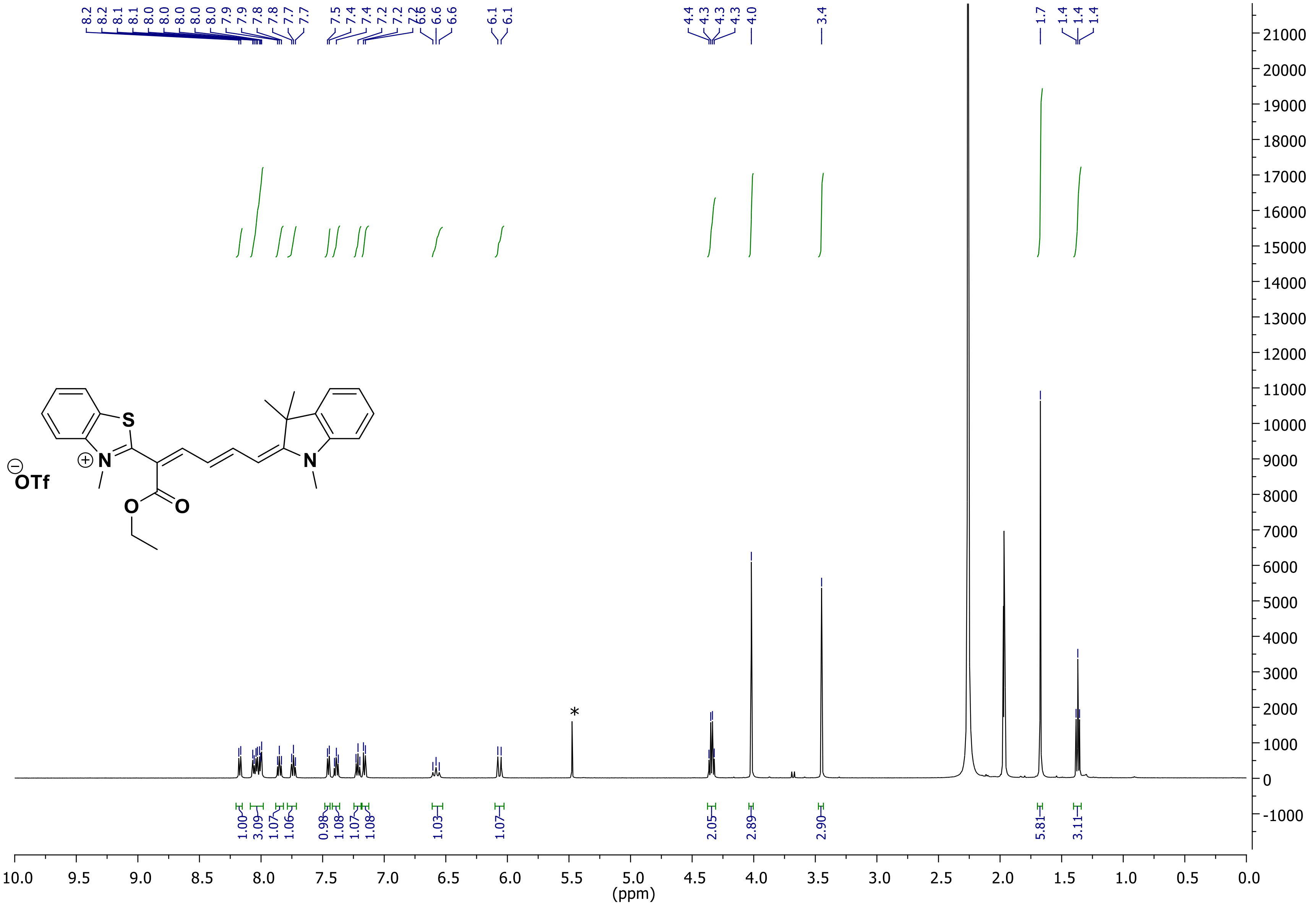

Figure S35. ^1H NMR (500 MHz, d_3-CD_3CN): **Cy5-5** (* dichloromethane).

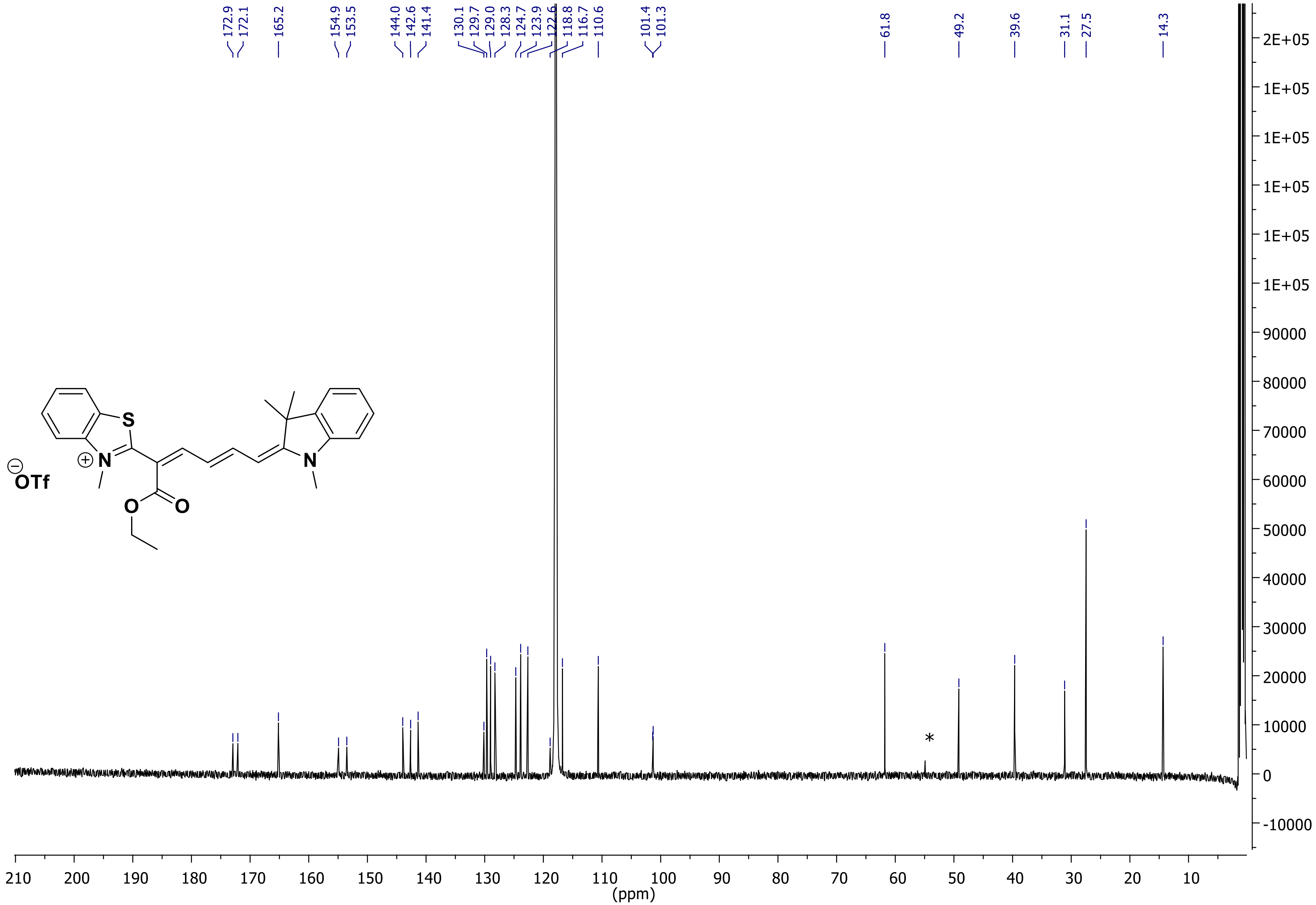

Figure S36. $^{13}C\{^1H\}$ NMR (126 MHz, d_3-CD_3CN): **Cy5-5** (* dichloromethane).

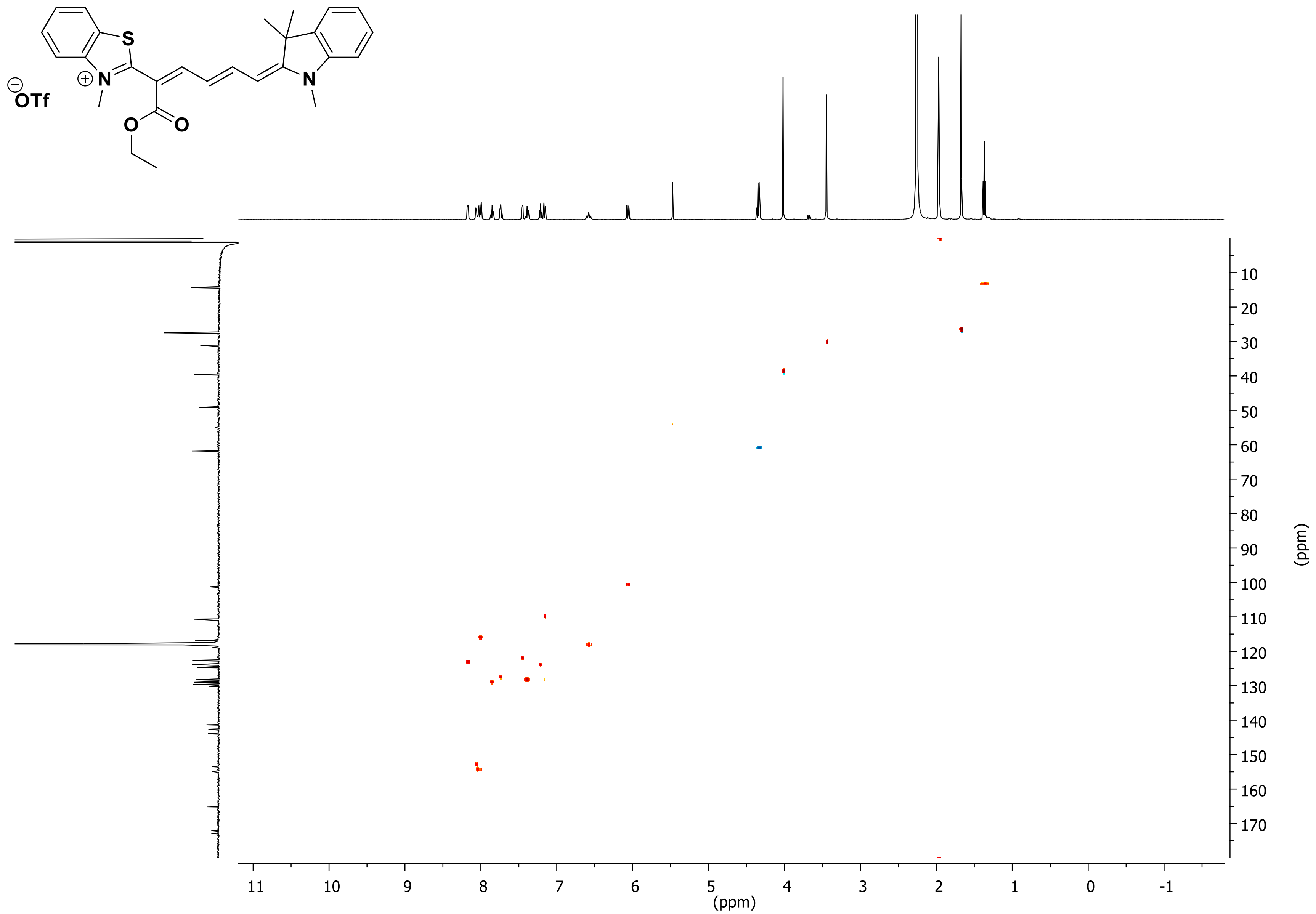

Figure S37. ^{1}H–$^{13}C\{^{1}H\}$ gHSQC (500 MHz, d_3-CD_3CN): **Cy5-5**.

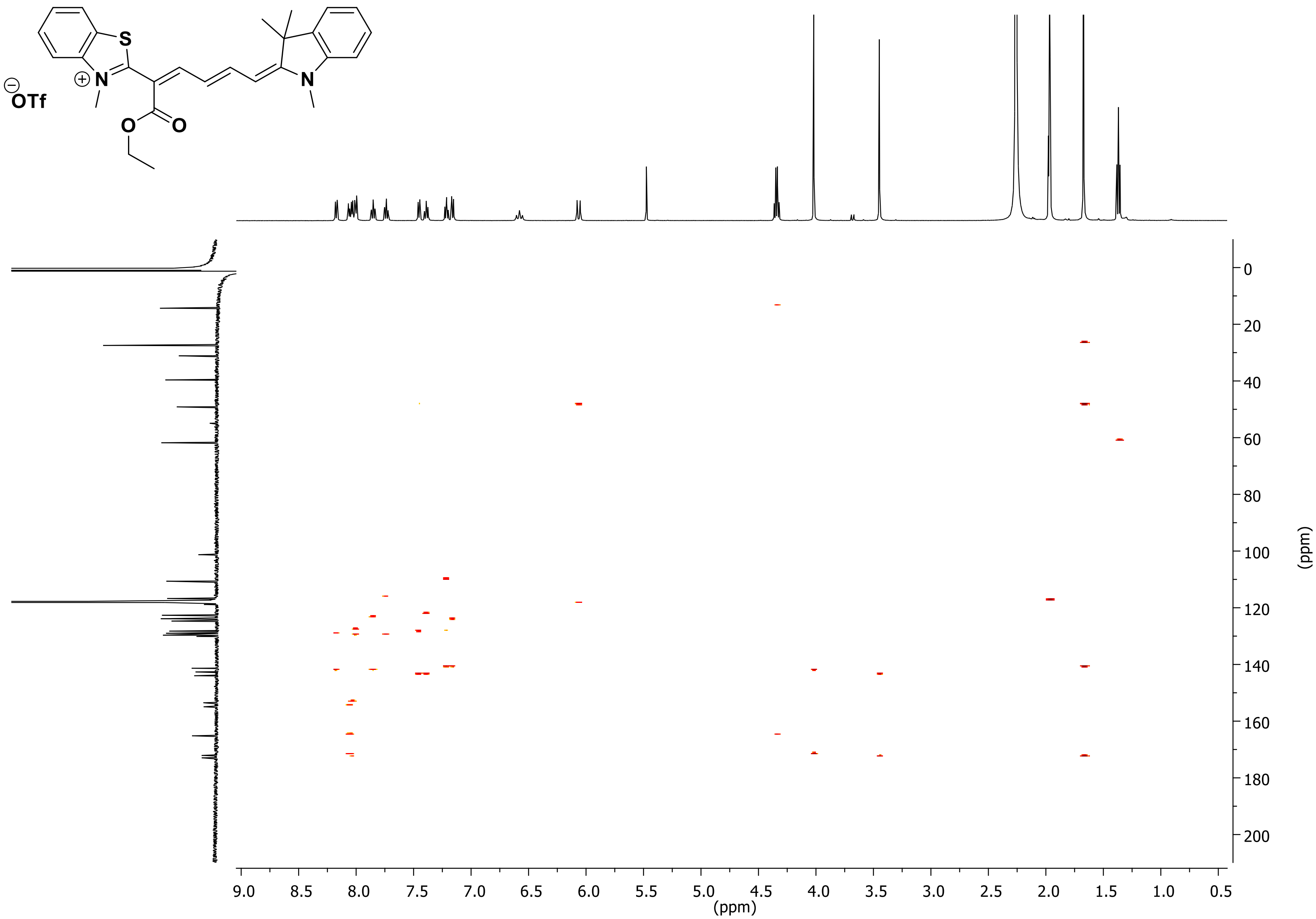

Figure S38. ^{1}H–$^{13}C\{^{1}H\}$ gHMBC (500 MHz, d_3-CD_3CN): **Cy5-5**.

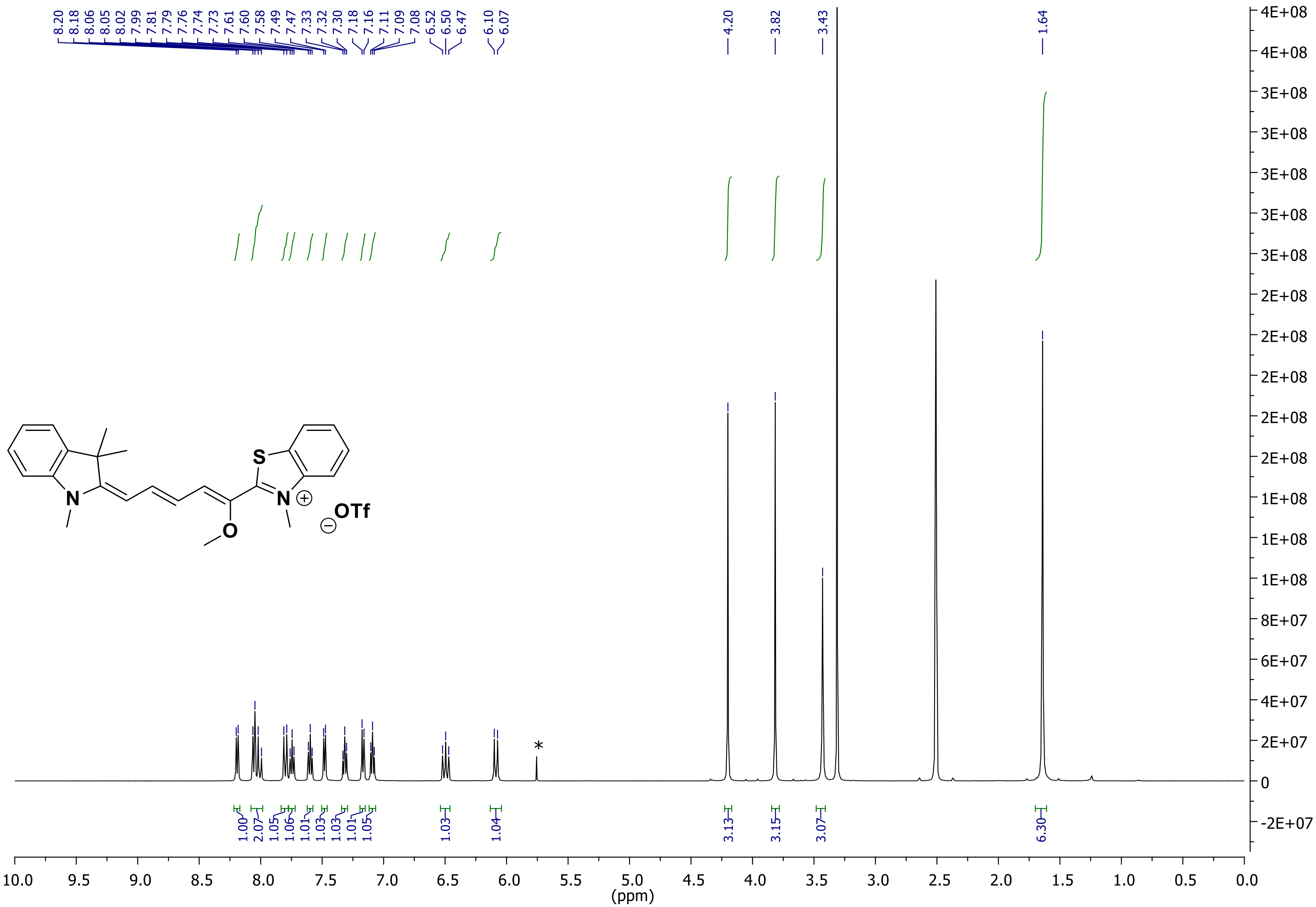

Figure S39. ^{1}H NMR (500 MHz, d_6–DMSO): **Cy5-7** (* dichloromethane).

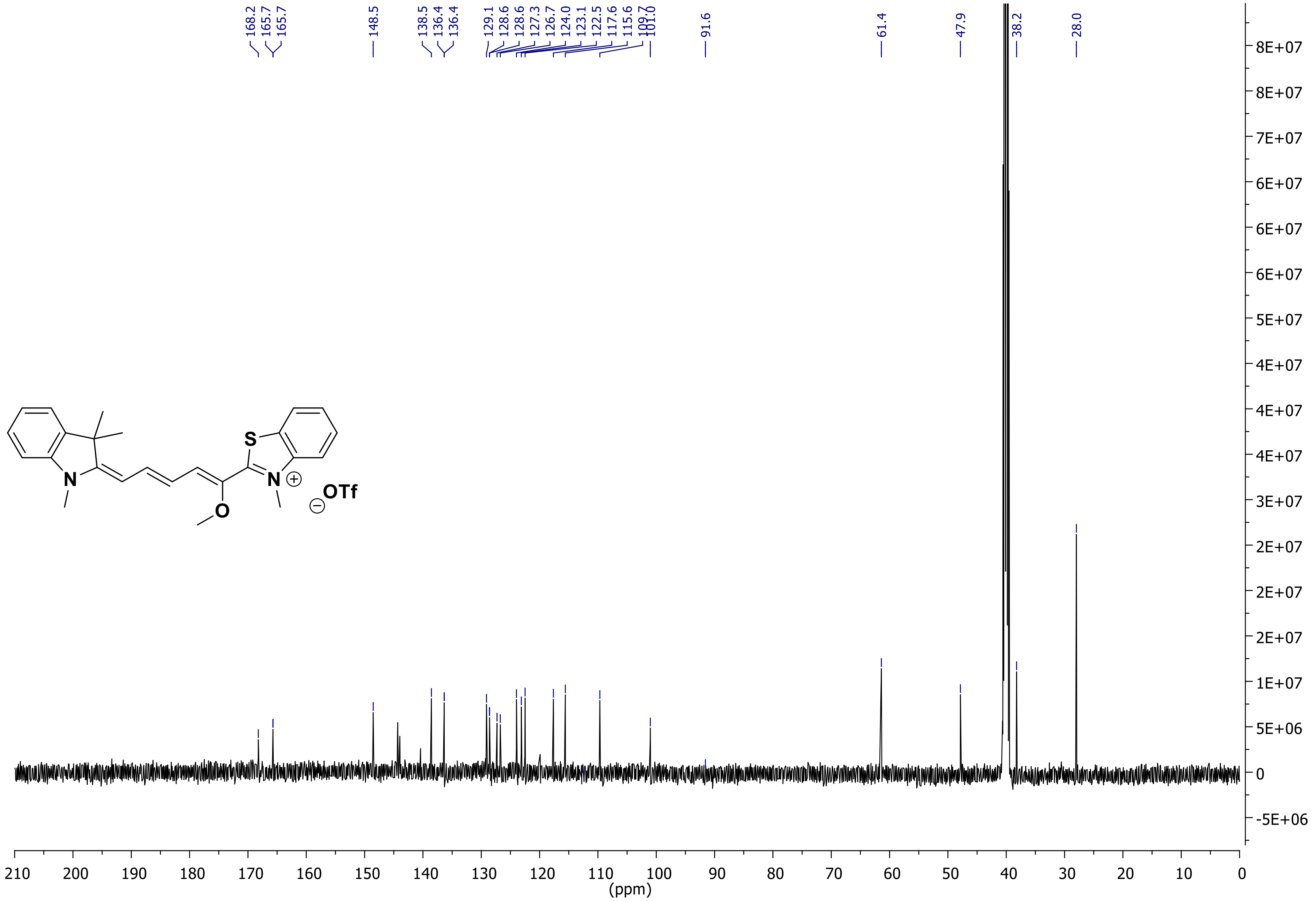

Figure S40. $^{13}C\{^1H\}$ NMR (126 MHz, d_6–DMSO): **Cy5-7**.

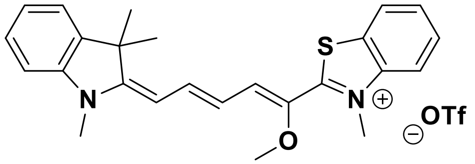

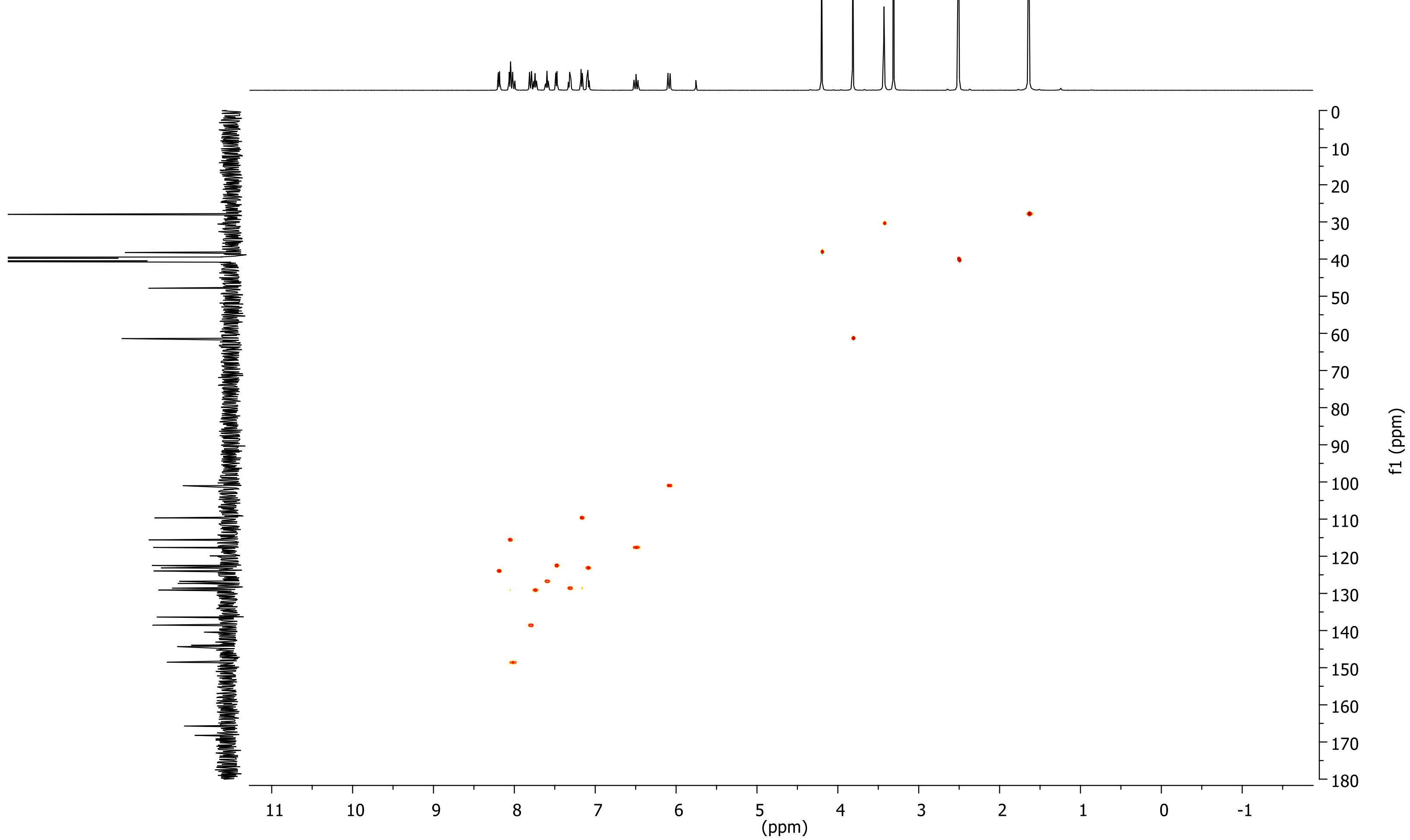

Figure S41. ^{1}H–$^{13}C\{^{1}H\}$ gHSQC (500 MHz, d_6–DMSO): **Cy5-7**.

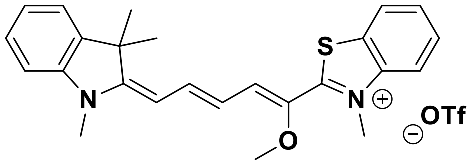

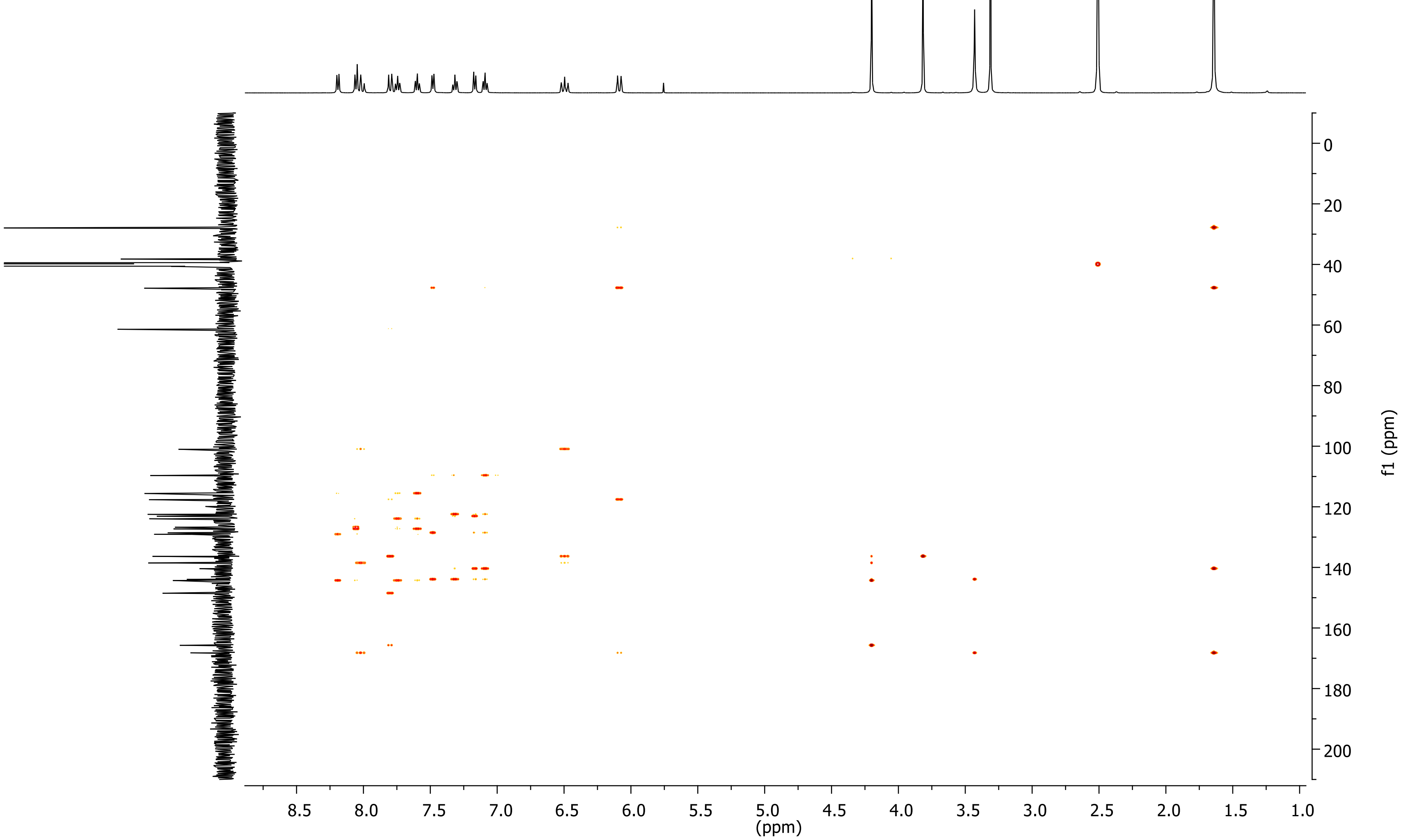

Figure S42. ^{1}H–$^{13}C\{^{1}H\}$ gHMBC (500 MHz, d_6–DMSO): **Cy5-7**.

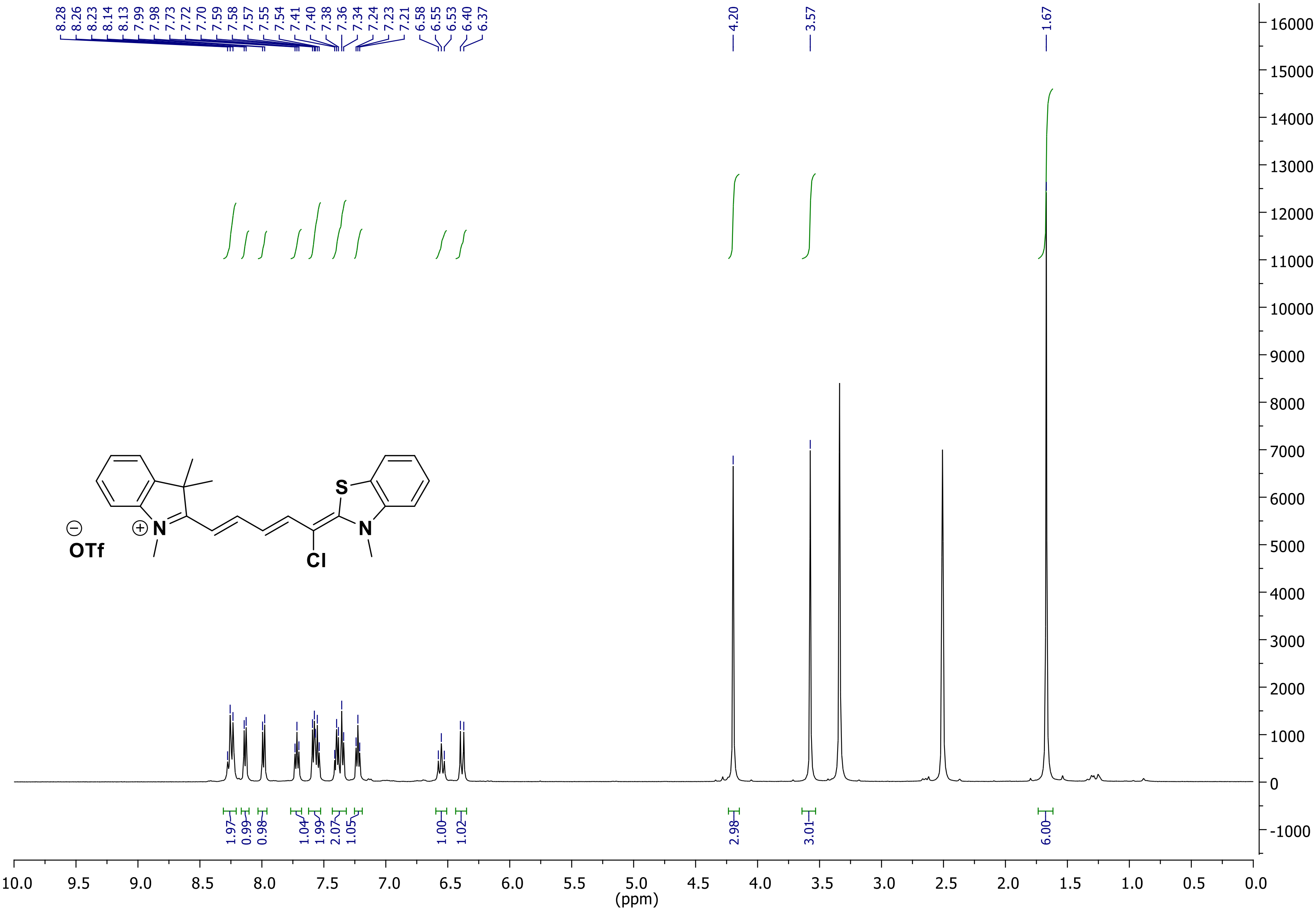

Figure S43. ^{1}H NMR (300 MHz, d_6–DMSO): **Cy5-8**.

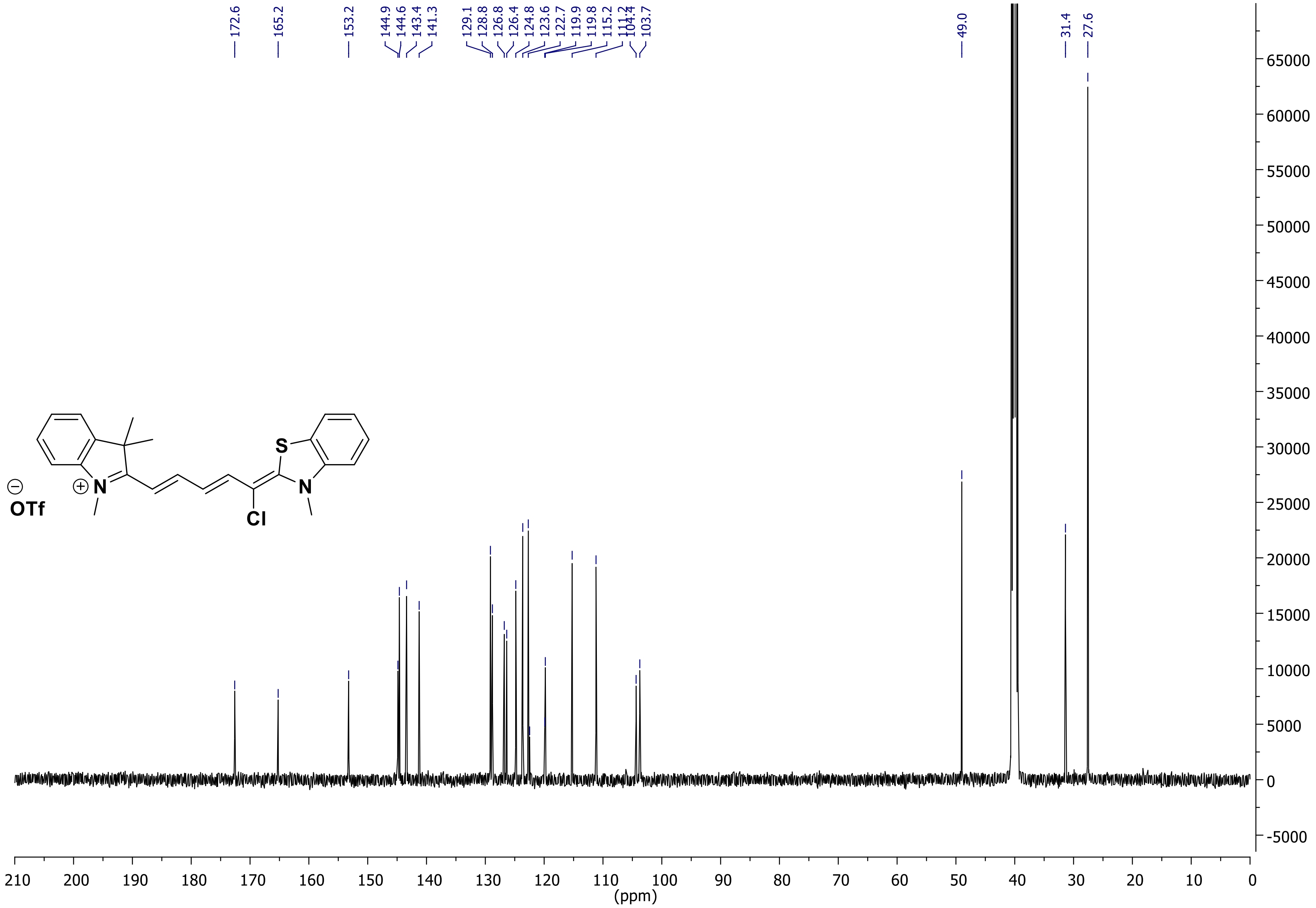

Figure S44. $^{13}C\{^{1}H\}$ NMR (126 MHz, d_6–DMSO): **Cy5-8**.

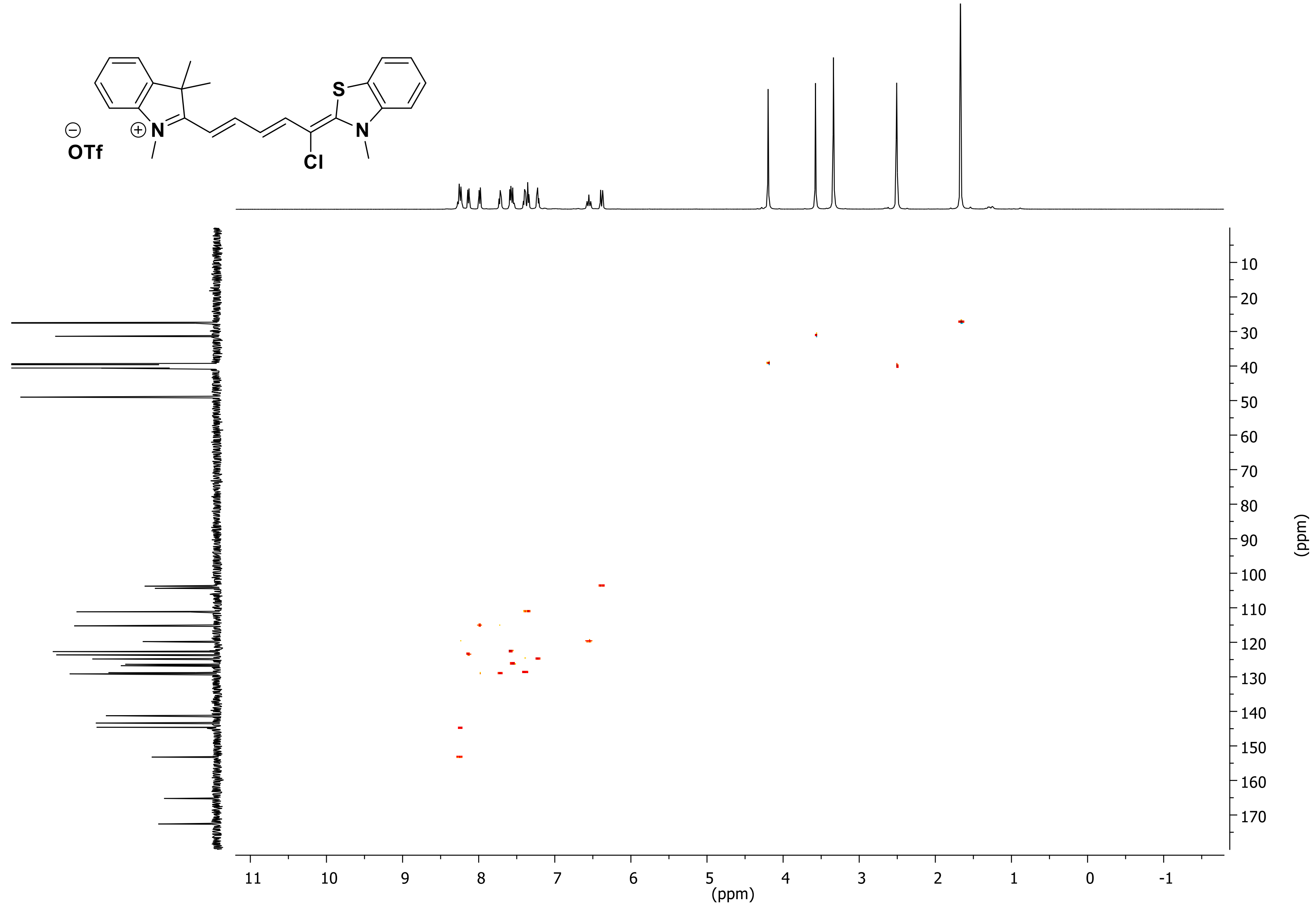

Figure S45. ^1H–$^{13}C\{^1H\}$ gHSQC (500 MHz, d_6–DMSO): **Cy5-8** .

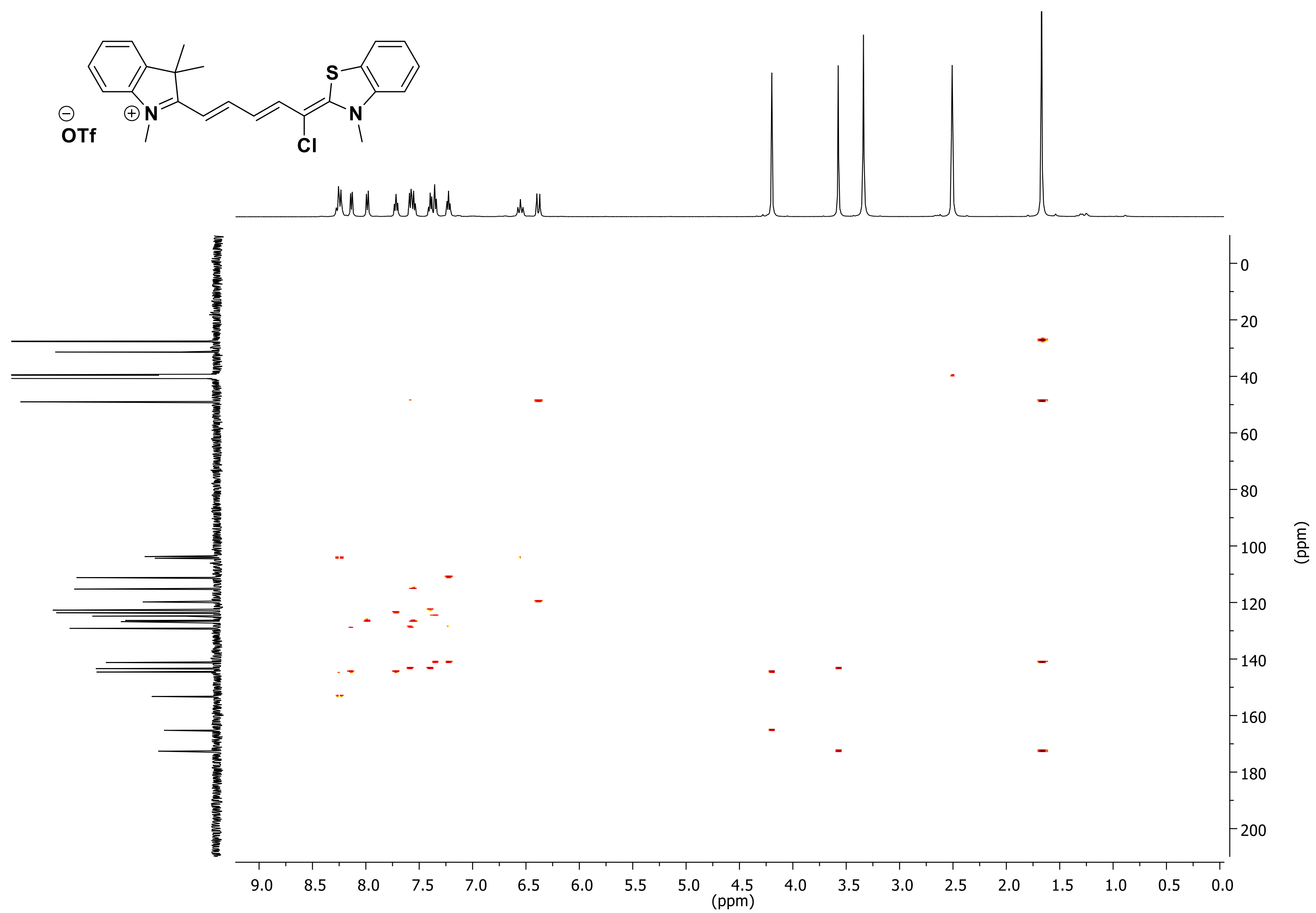

Figure S46. ^{1}H–$^{13}C\{^{1}H\}$ gHMBC (500 MHz, d_6–DMSO): **Cy5-8**.

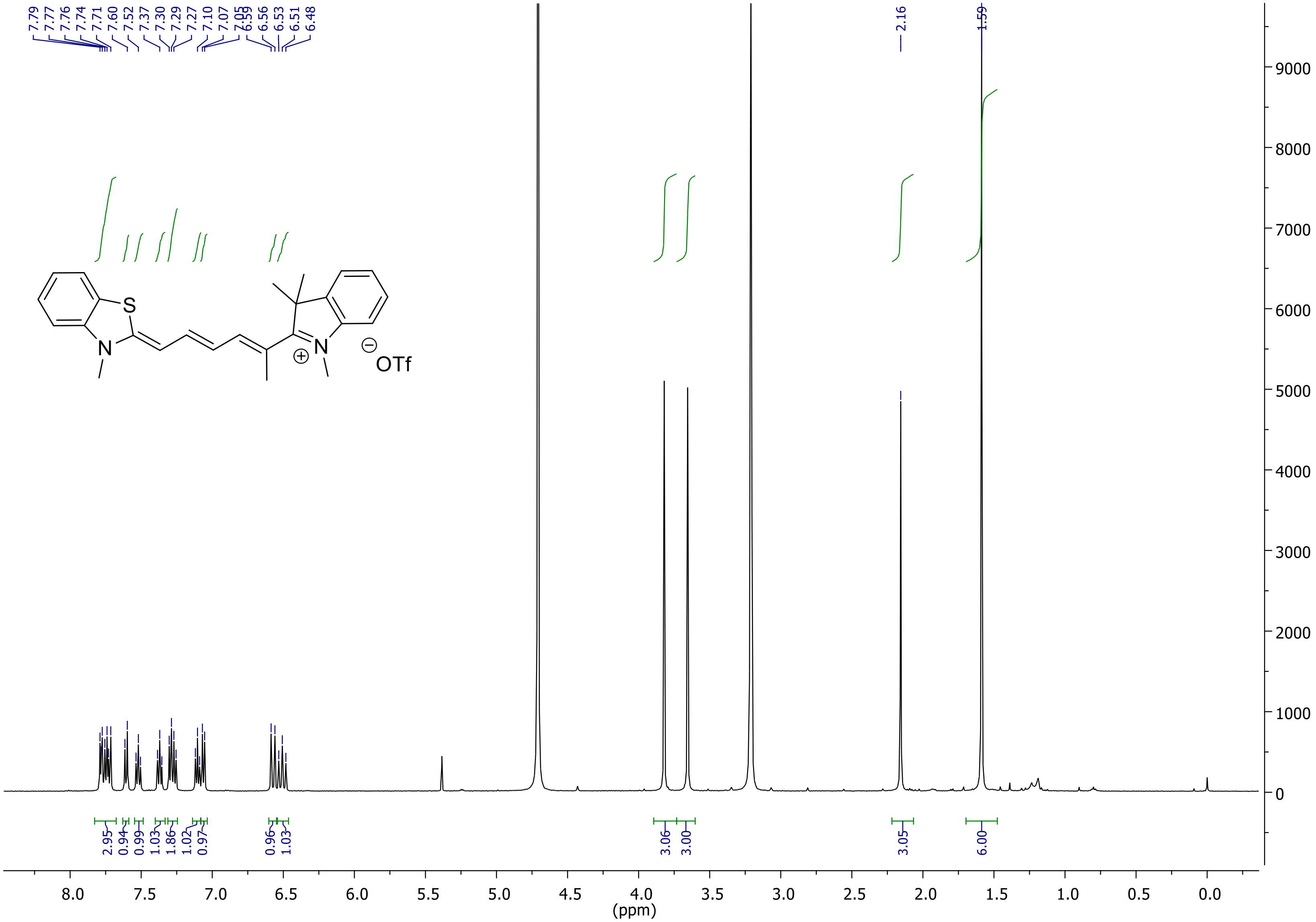

Figure S47. ^{1}H NMR (500 MHz, d_4-CD_3OD): **Cy5-10**.

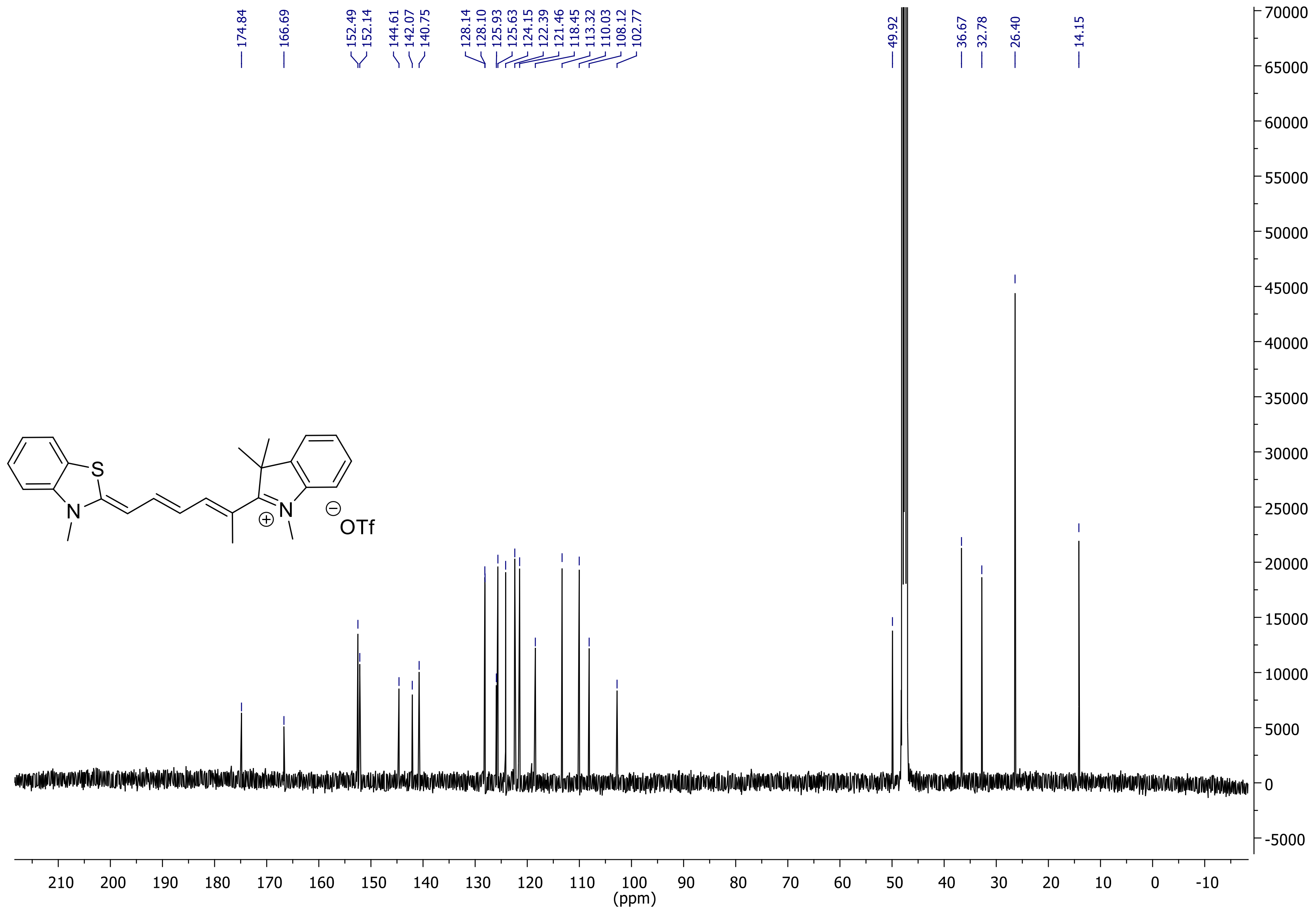

Figure S48. $^{13}C\{^{1}H\}$ NMR (126 MHz, d_4-CD_3OD): **Cy5-10**.

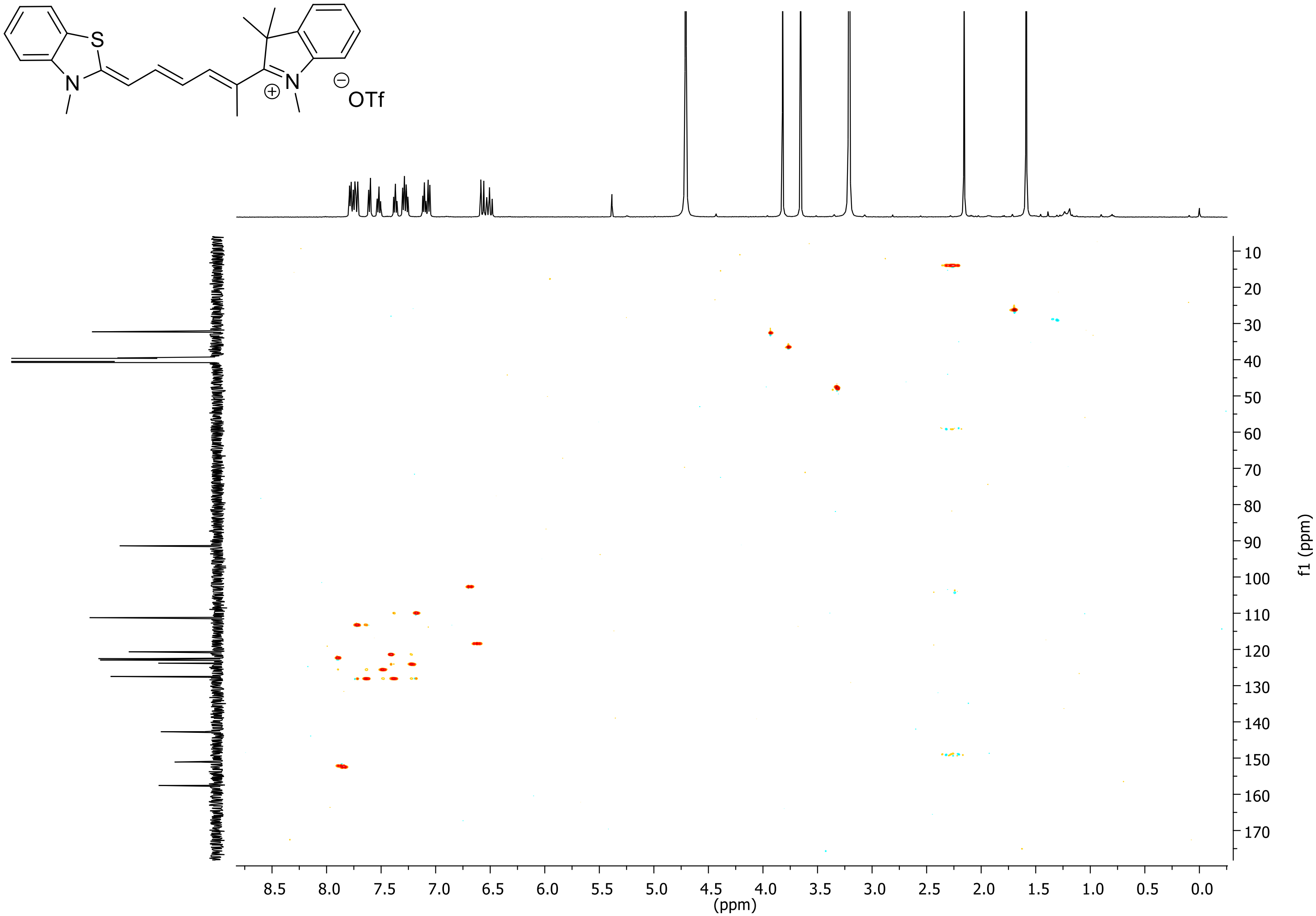

Figure S49. ^{1}H–$^{13}C\{^{1}H\}$ gHSQC (500 MHz, d_4-CD_3OD): **OB227 Cy5-10**.

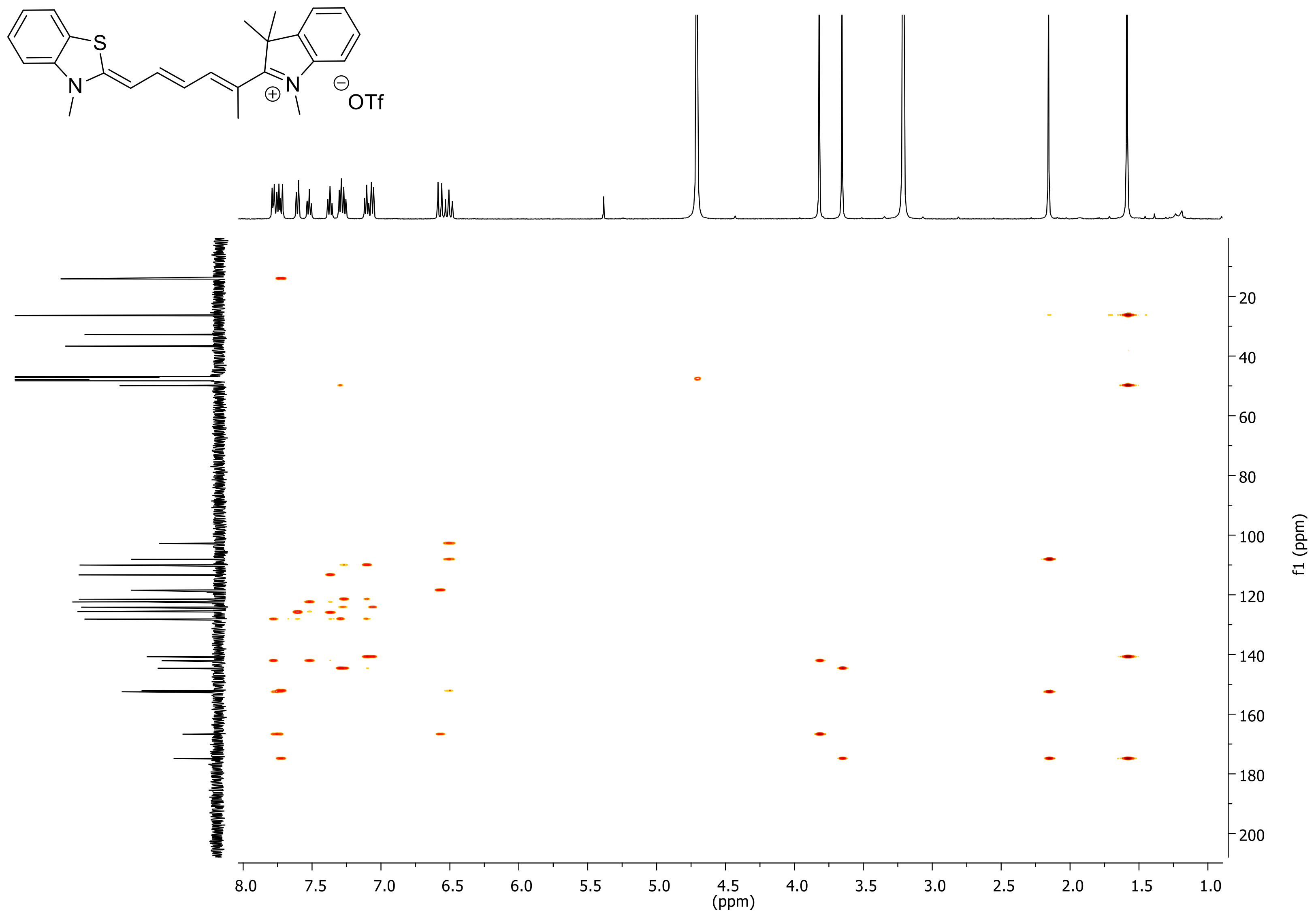

Figure S50. ${}^{1}H$–${}^{13}C\{{}^{1}H\}$ gHMBC (500 MHz, d_4-CD_3OD): **OB227 Cy5-10**.

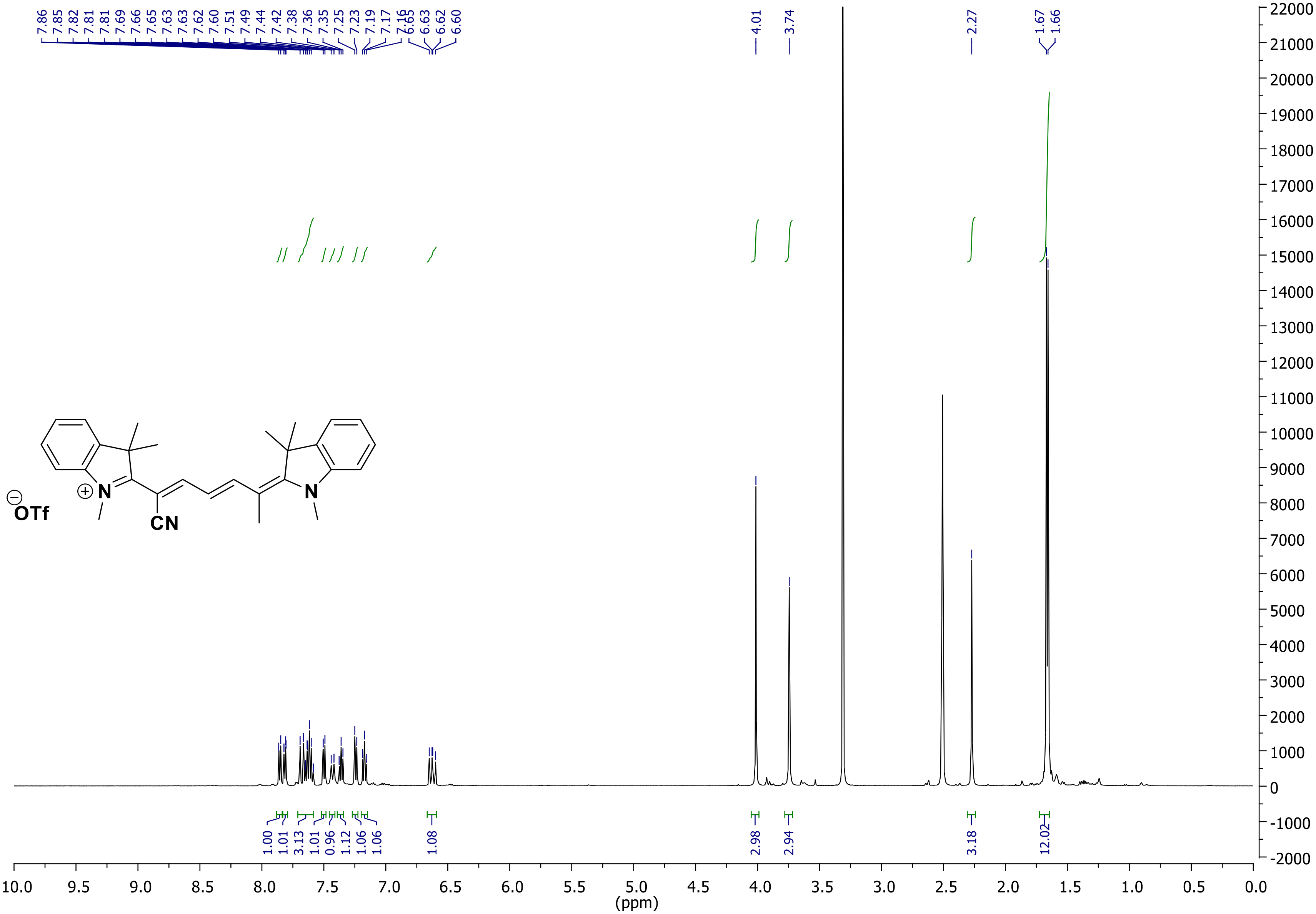

Figure S51. ^{1}H NMR (500 MHz, d_6–DMSO): **Cy5-11**.

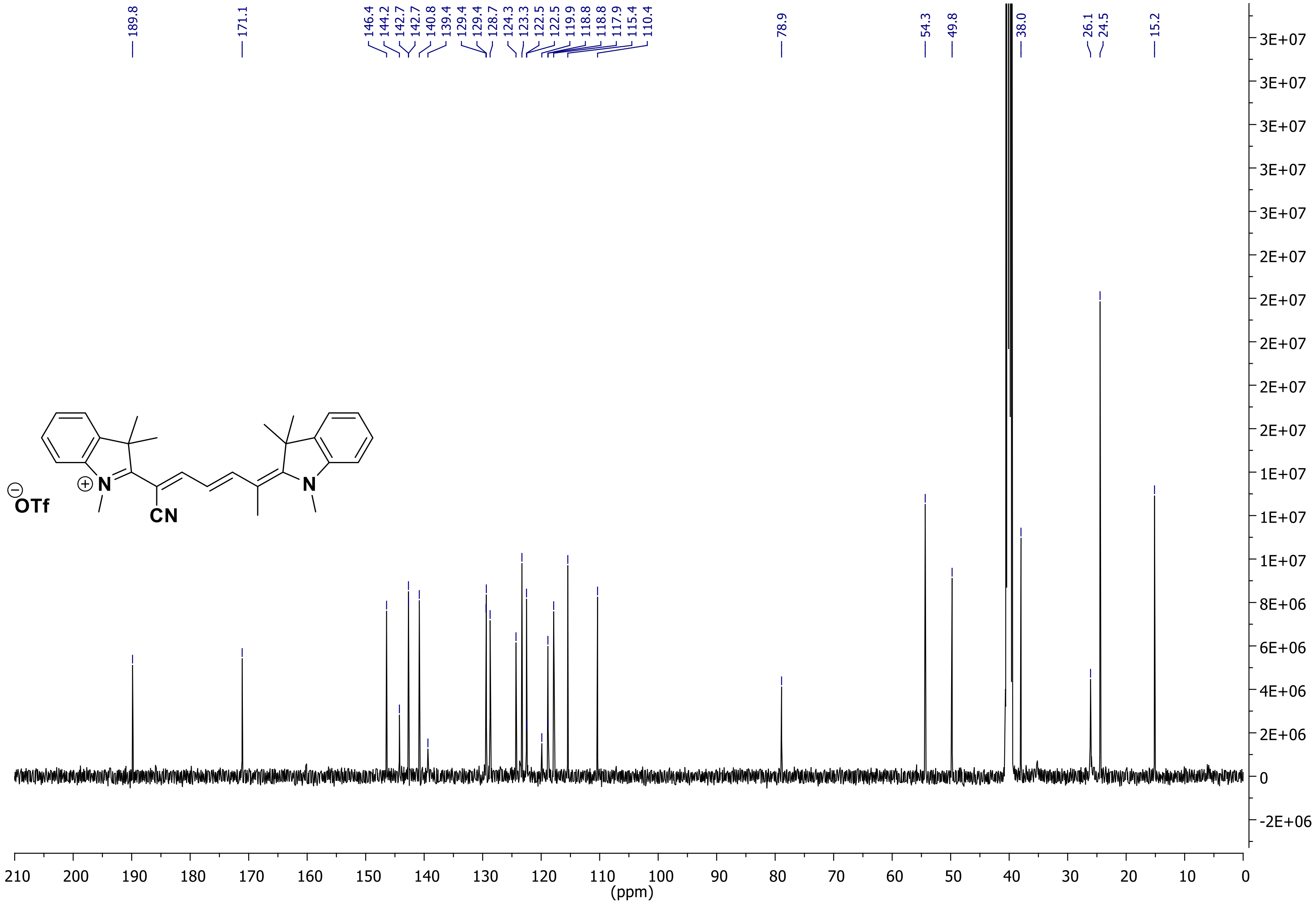

Figure S52. $^{13}C\{^{1}H\}$ NMR (126 MHz, d_6–DMSO): **Cy5-11**.

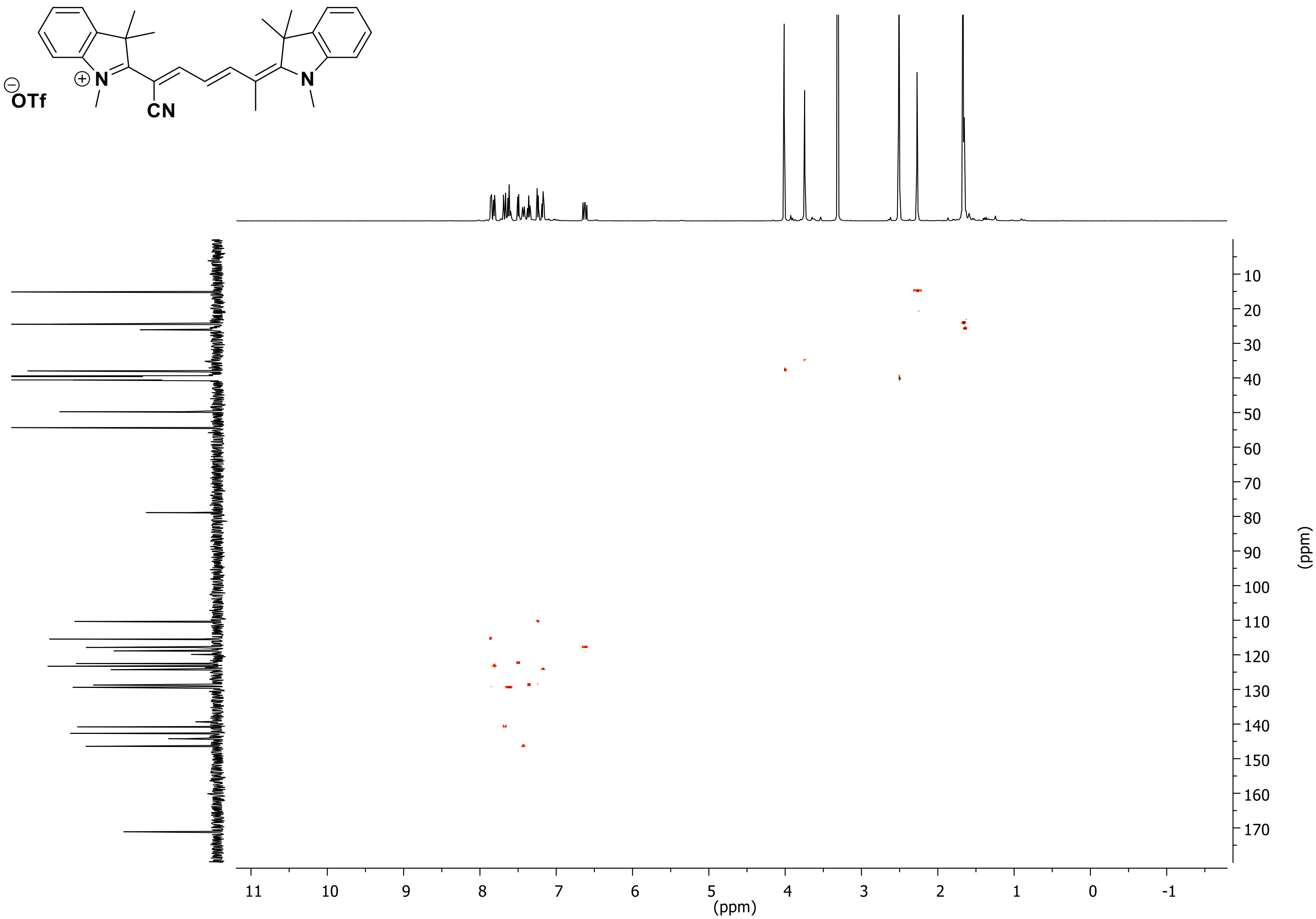

Figure S53. ^{1}H–^{13}C{^{1}H} gHSQC (500 MHz, d_6–DMSO): **Cy5-11**.

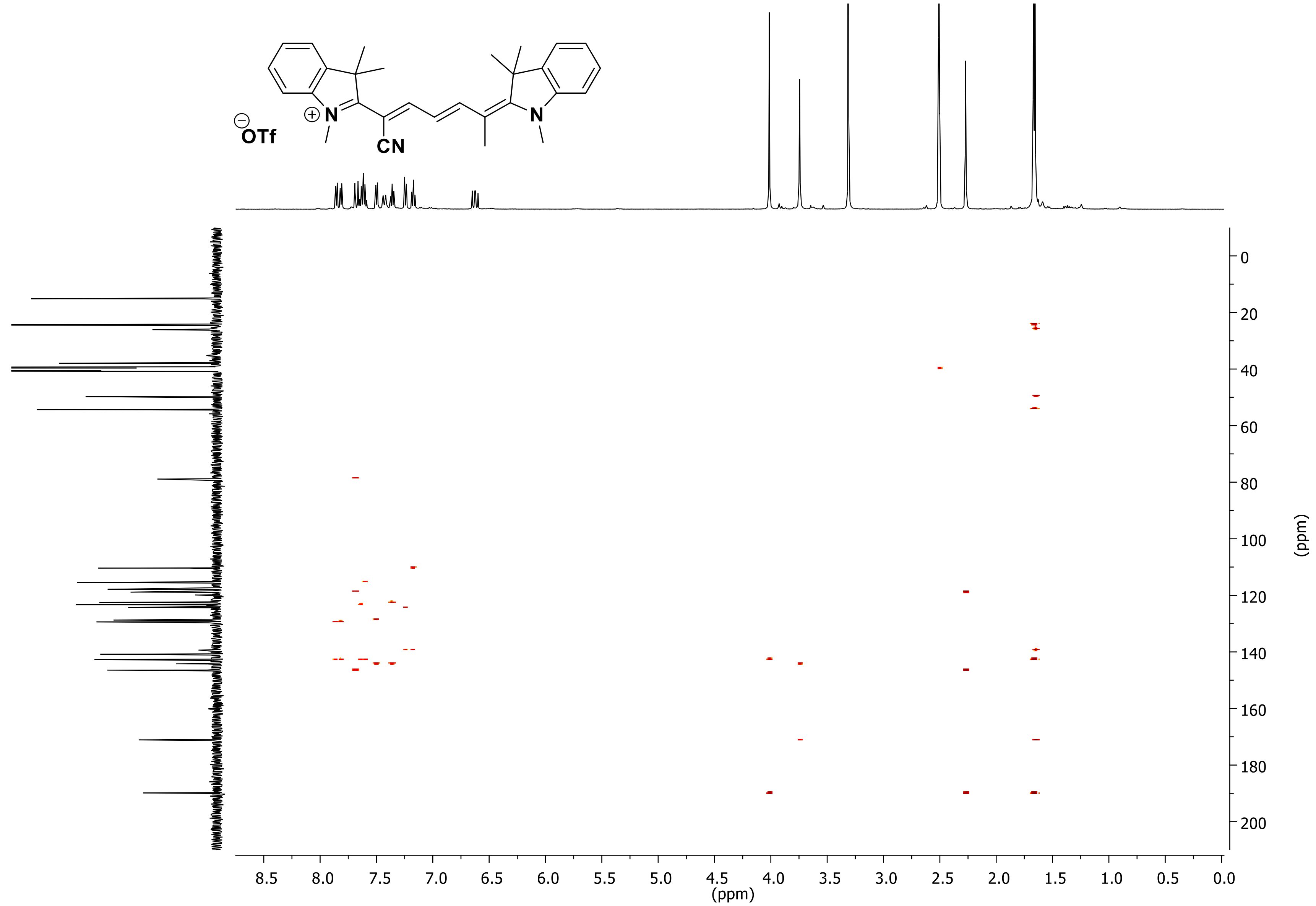

Figure S54. ^{1}H–$^{13}C\{^{1}H\}$ gHMBC (500 MHz, d_6–DMSO): **Cy5-11**.

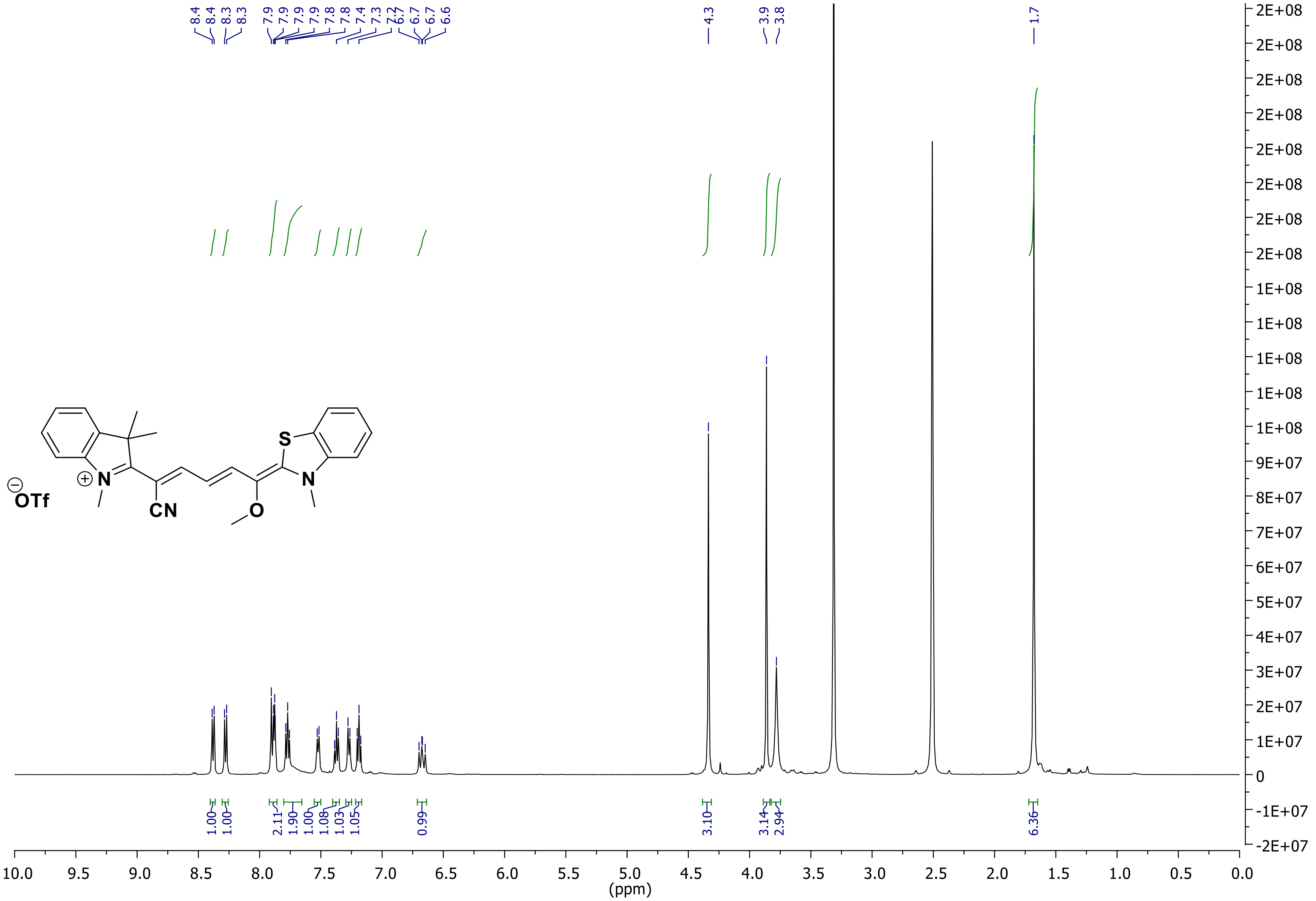

Figure S55. ^{1}H NMR (500 MHz, d_6–DMSO): **Cy5-12**.

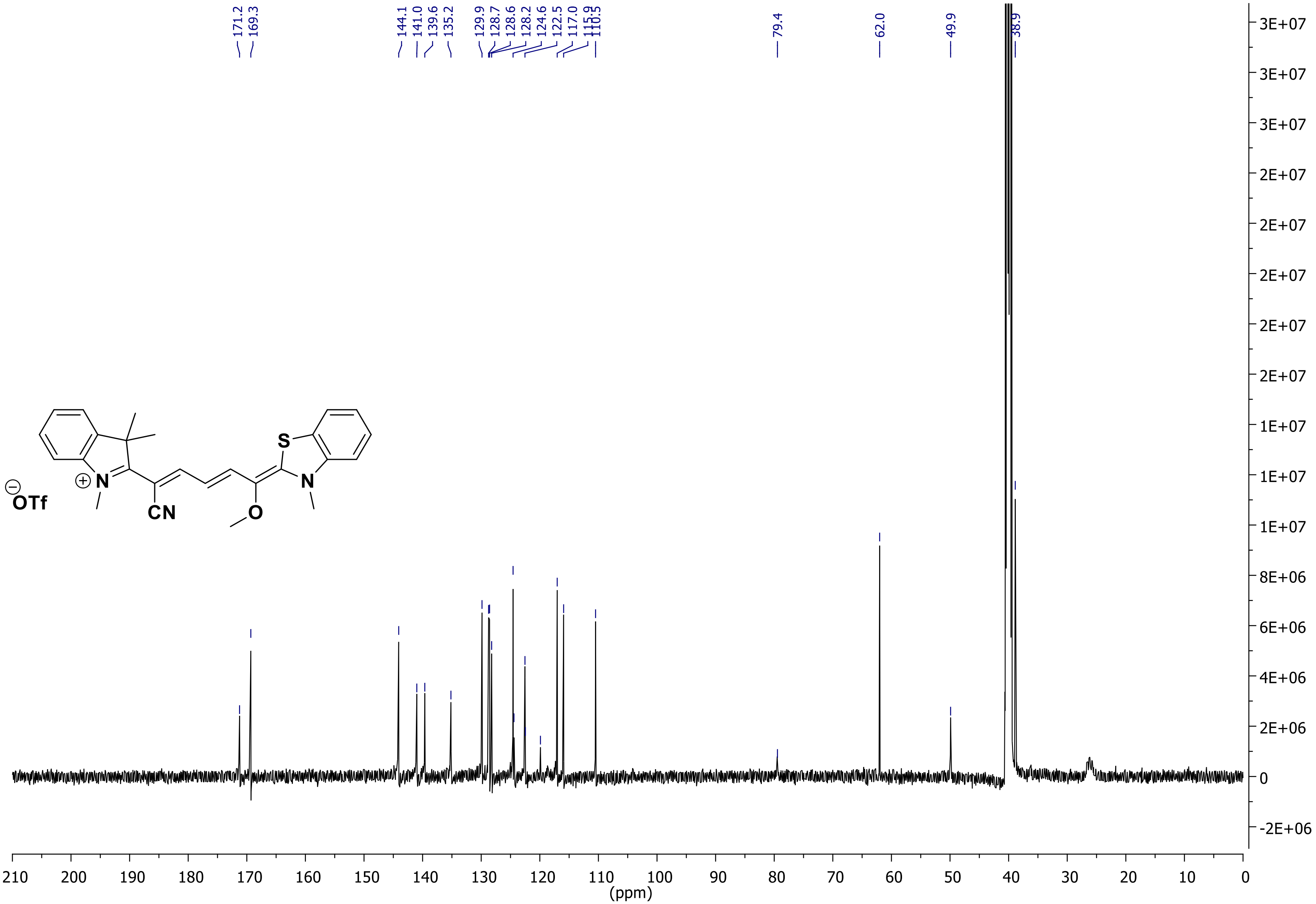

Figure S56. $^{13}C\{^{1}H\}$ NMR (126 MHz, d_6–DMSO): **Cy5-12**.

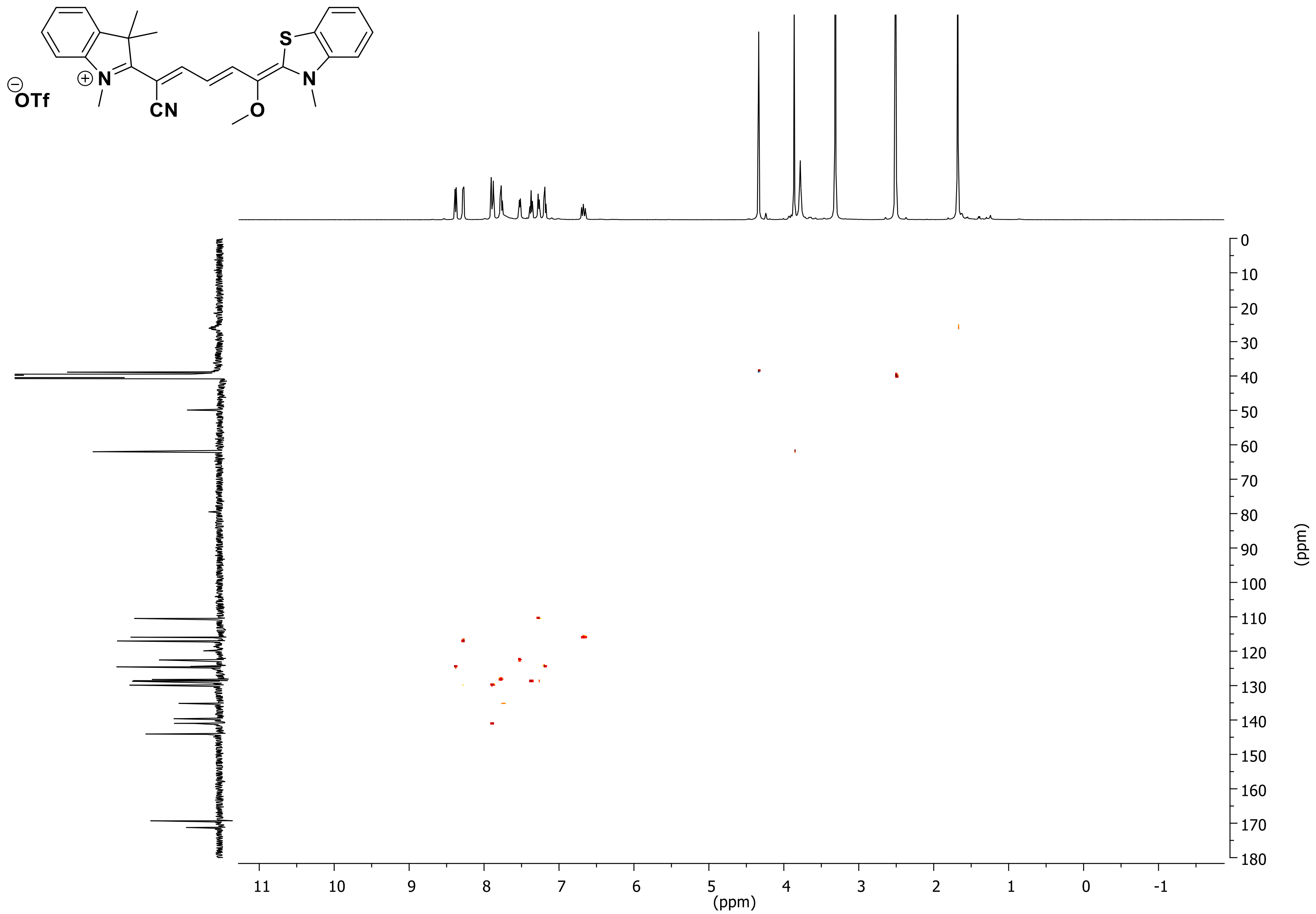

Figure S57. ^{1}H–$^{13}C\{^{1}H\}$ gHSQC (500 MHz, d_6–DMSO): **Cy5-12**.

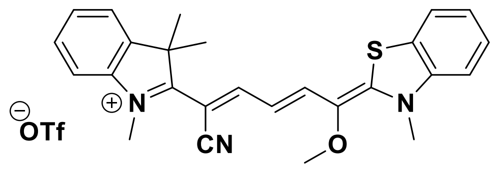

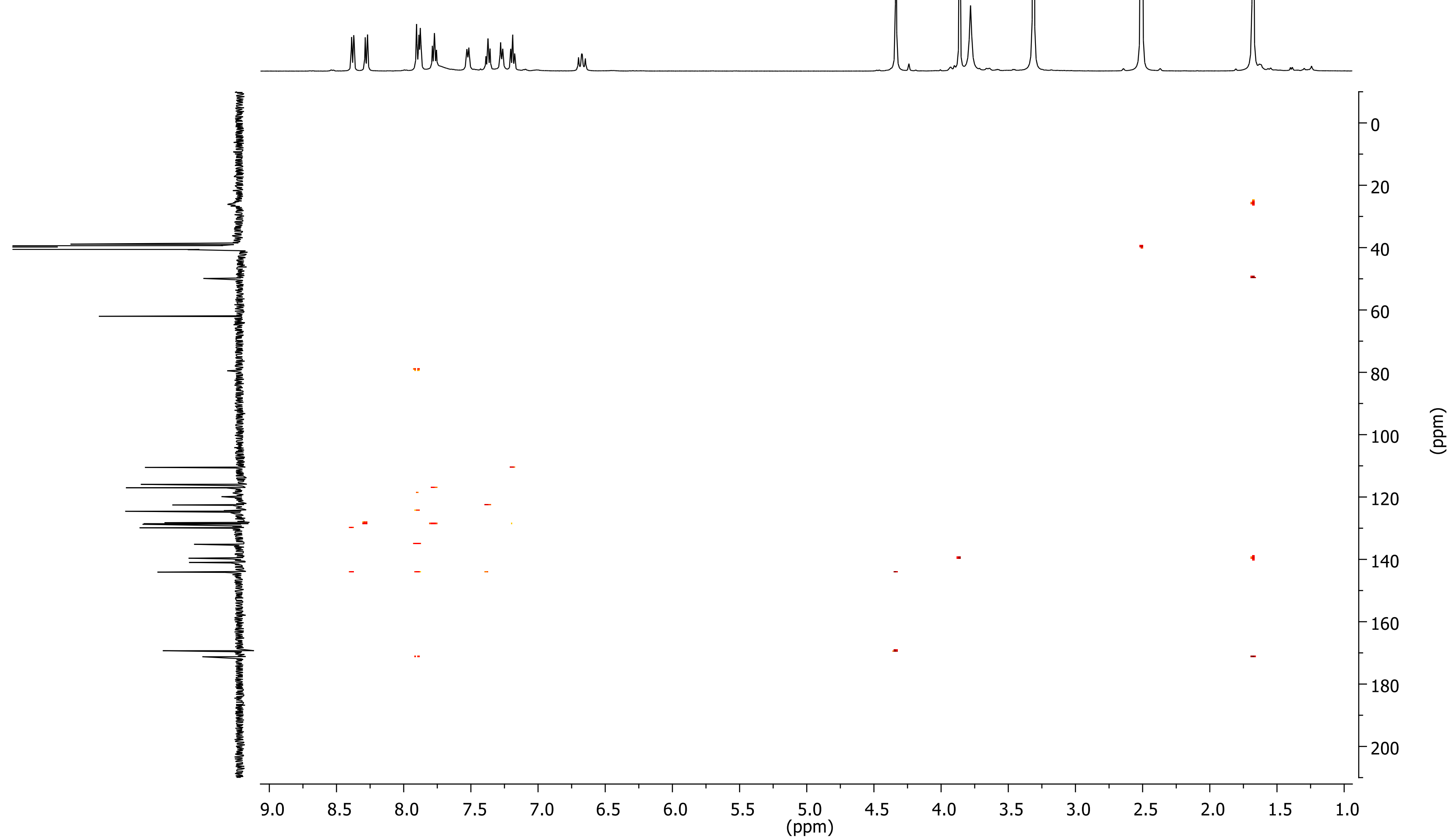

Figure S58. ^{1}H–$^{13}C\{^{1}H\}$ gHMBC (500 MHz, d_6–DMSO): **Cy5-12**.

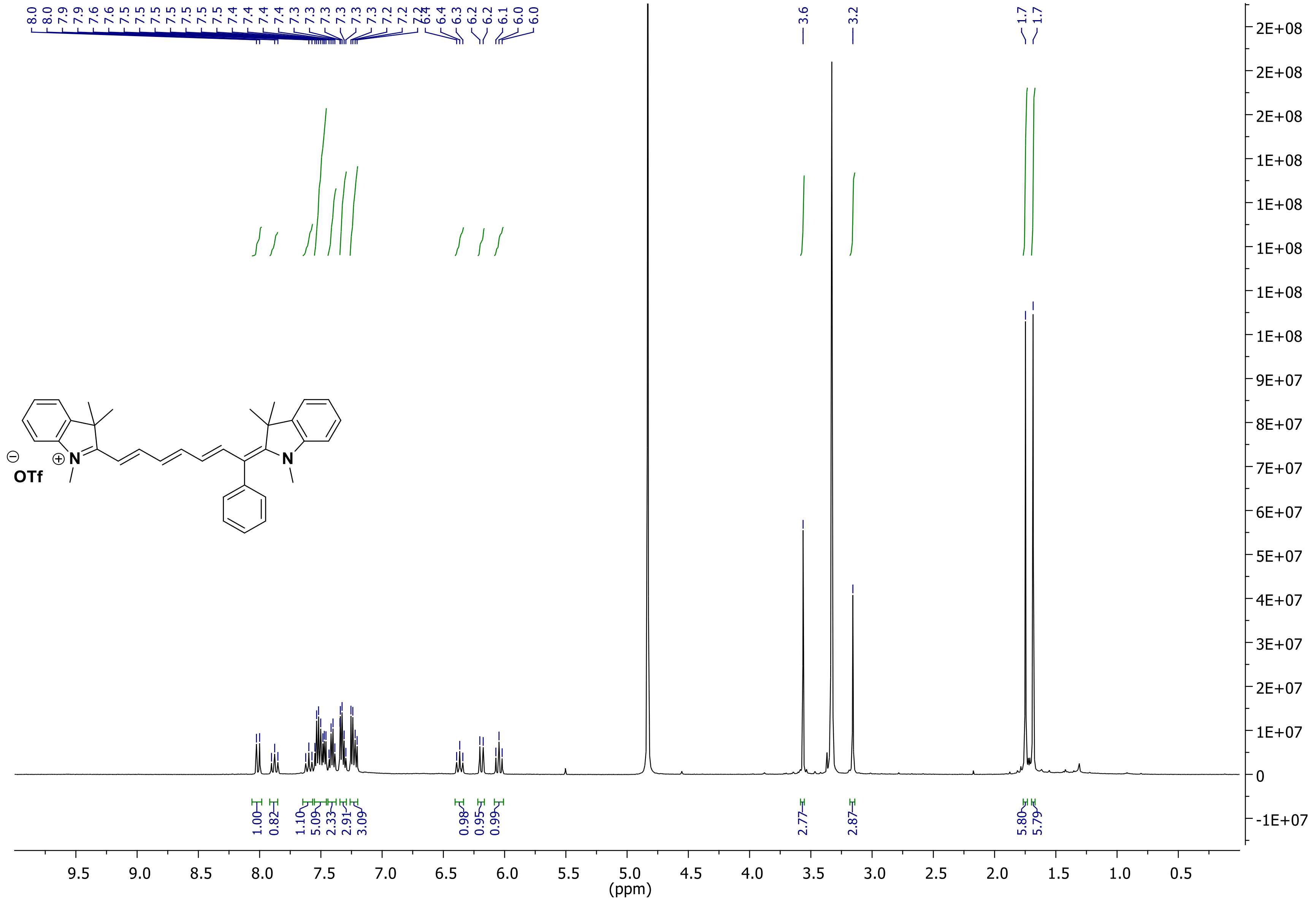

Figure S59. ^{1}H NMR (500 MHz, d_4–CD_3OD): **Cy7-2**.

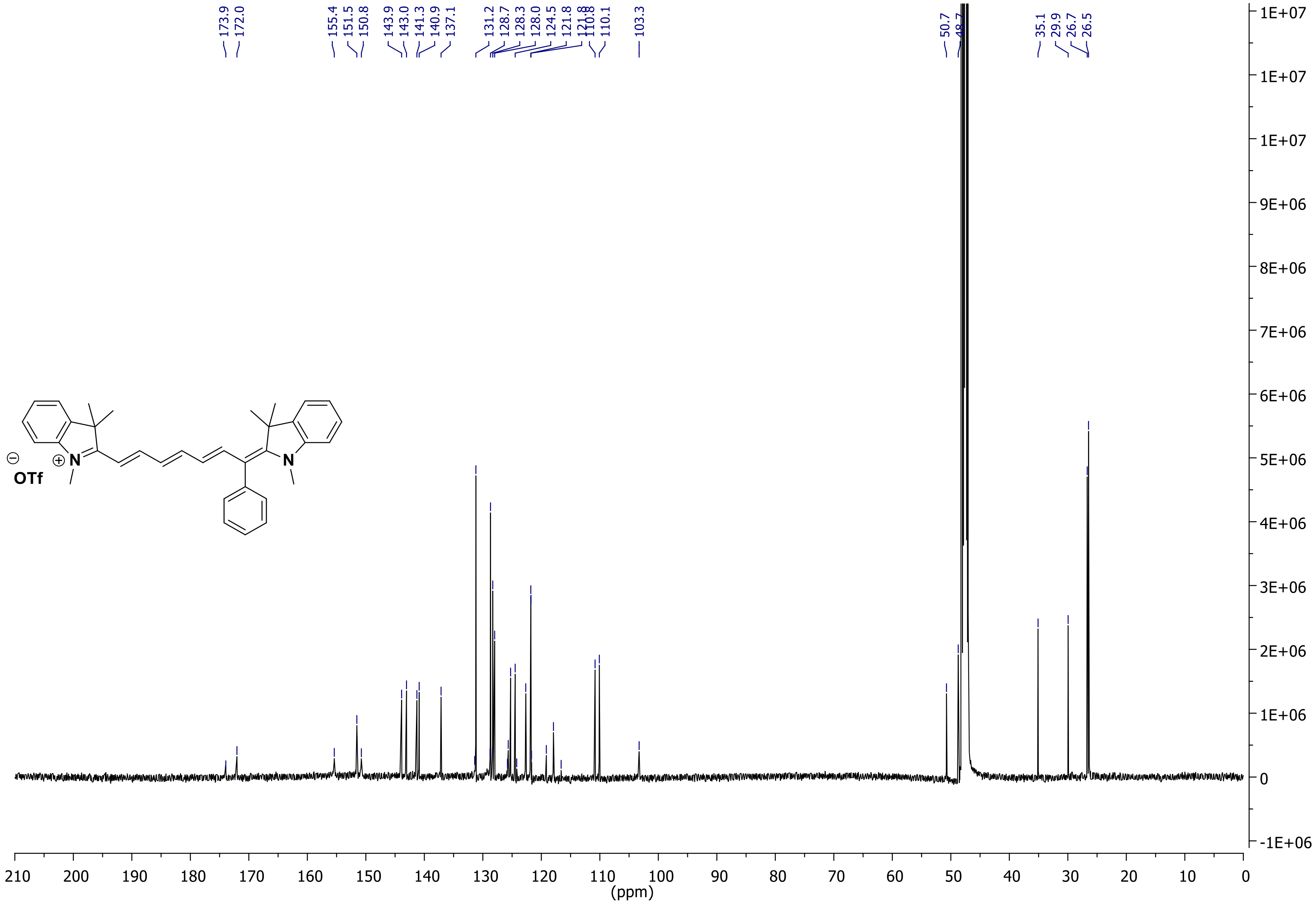

Figure S60. $^{13}C\{^{1}H\}$ NMR (126 MHz, *d*$_4$–CD_3OD): **Cy7-2**.

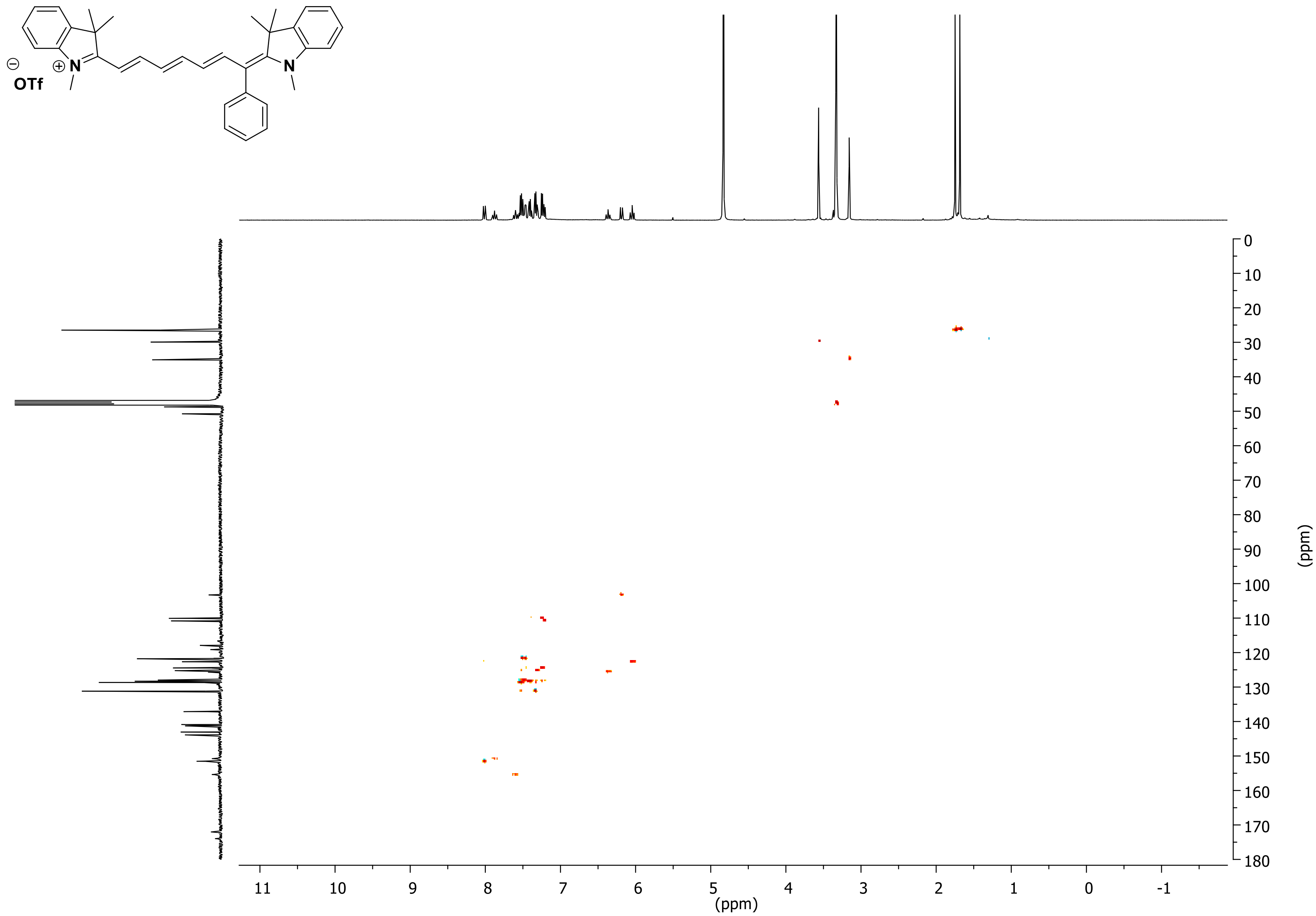

Figure S61. ^{1}H–$^{13}C\{^{1}H\}$ gHSQC (500 MHz, d_4–CD_3OD): **Cy7-2**.

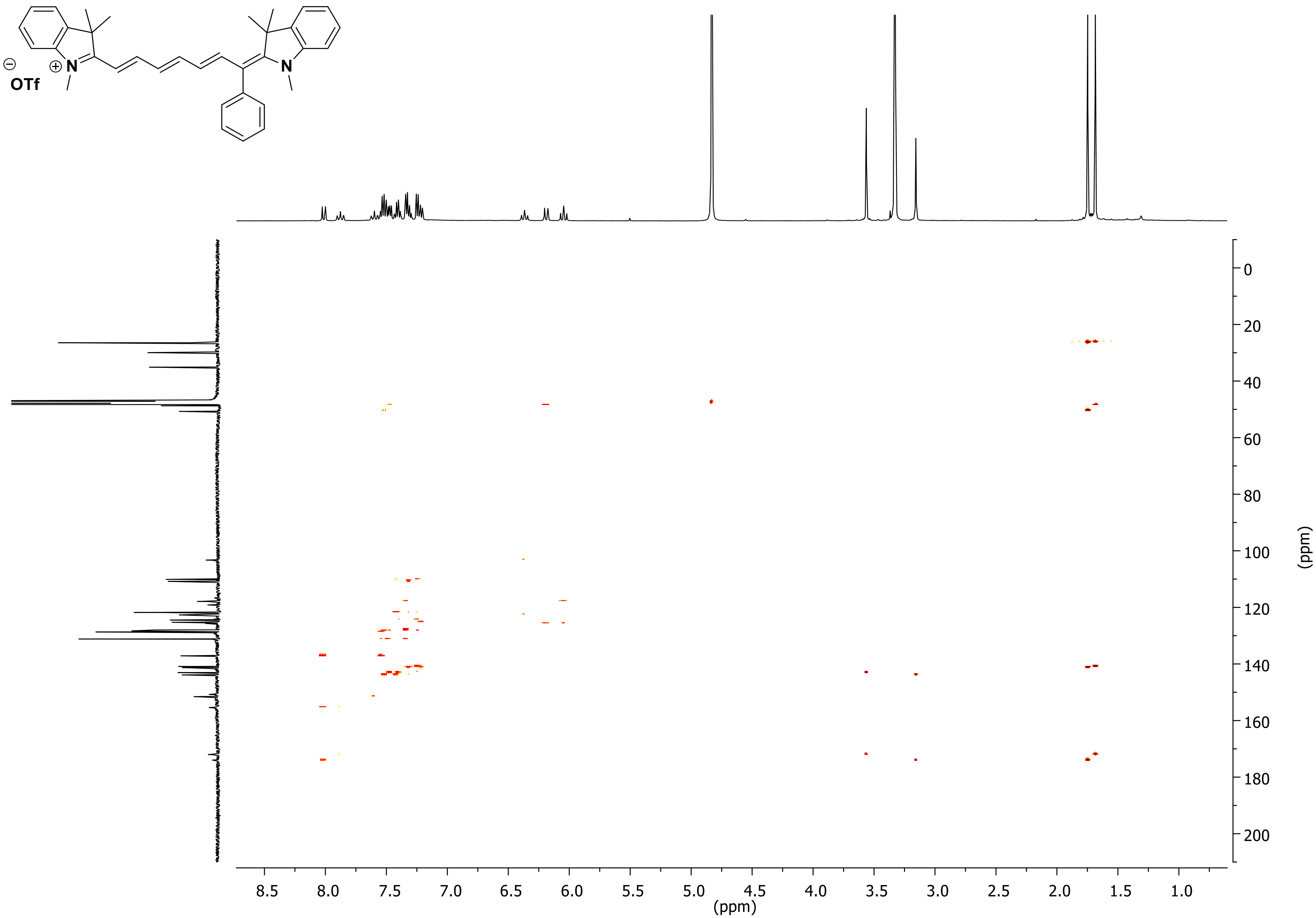

Figure S62. ^{1}H–$^{13}C\{^{1}H\}$ gHMBC (500 MHz, d_4–CD_3OD): **Cy7-2**.

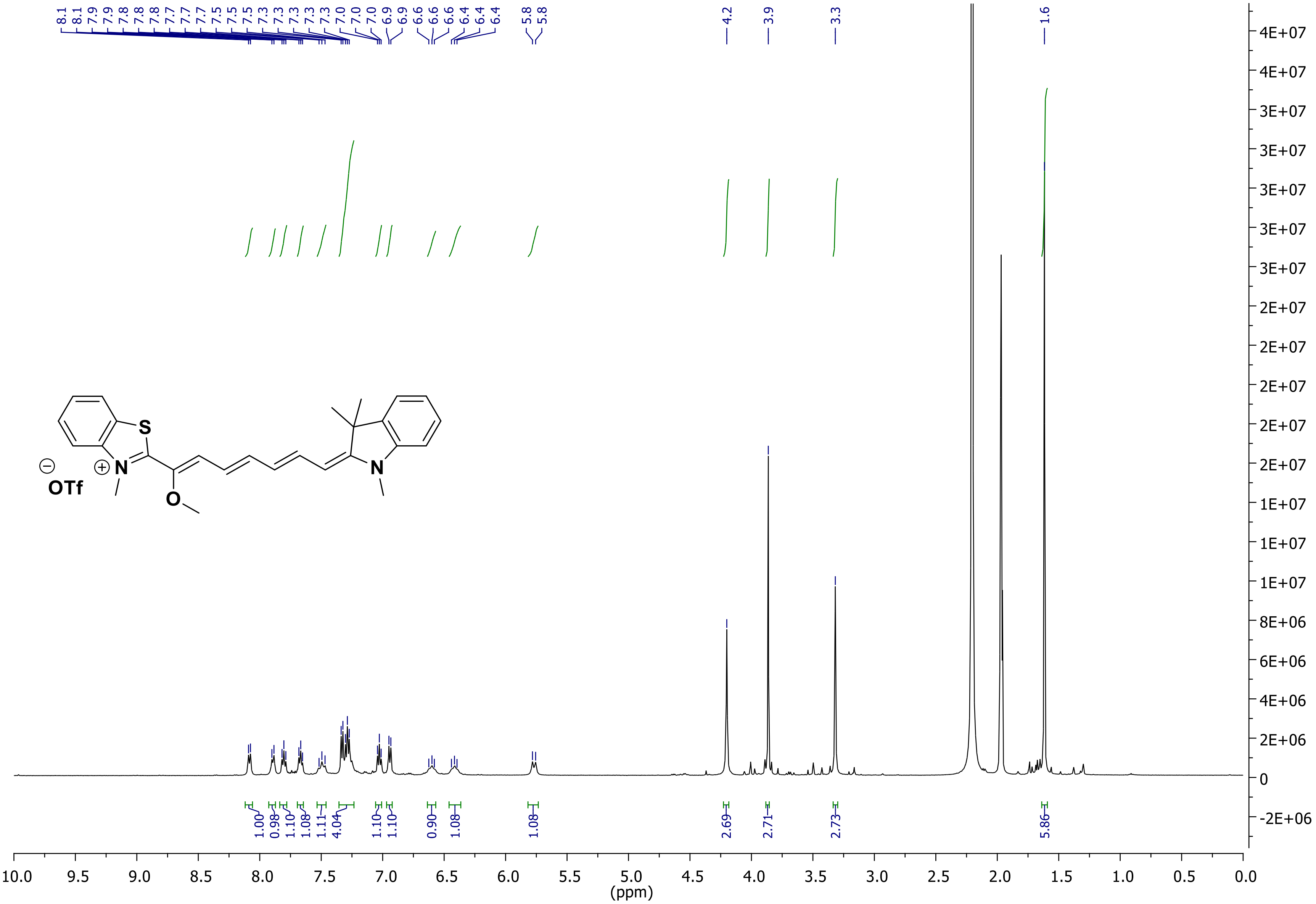

Figure S63. ^{1}H NMR (500 MHz, d_3-CD_3CN): **Cy7-3**.

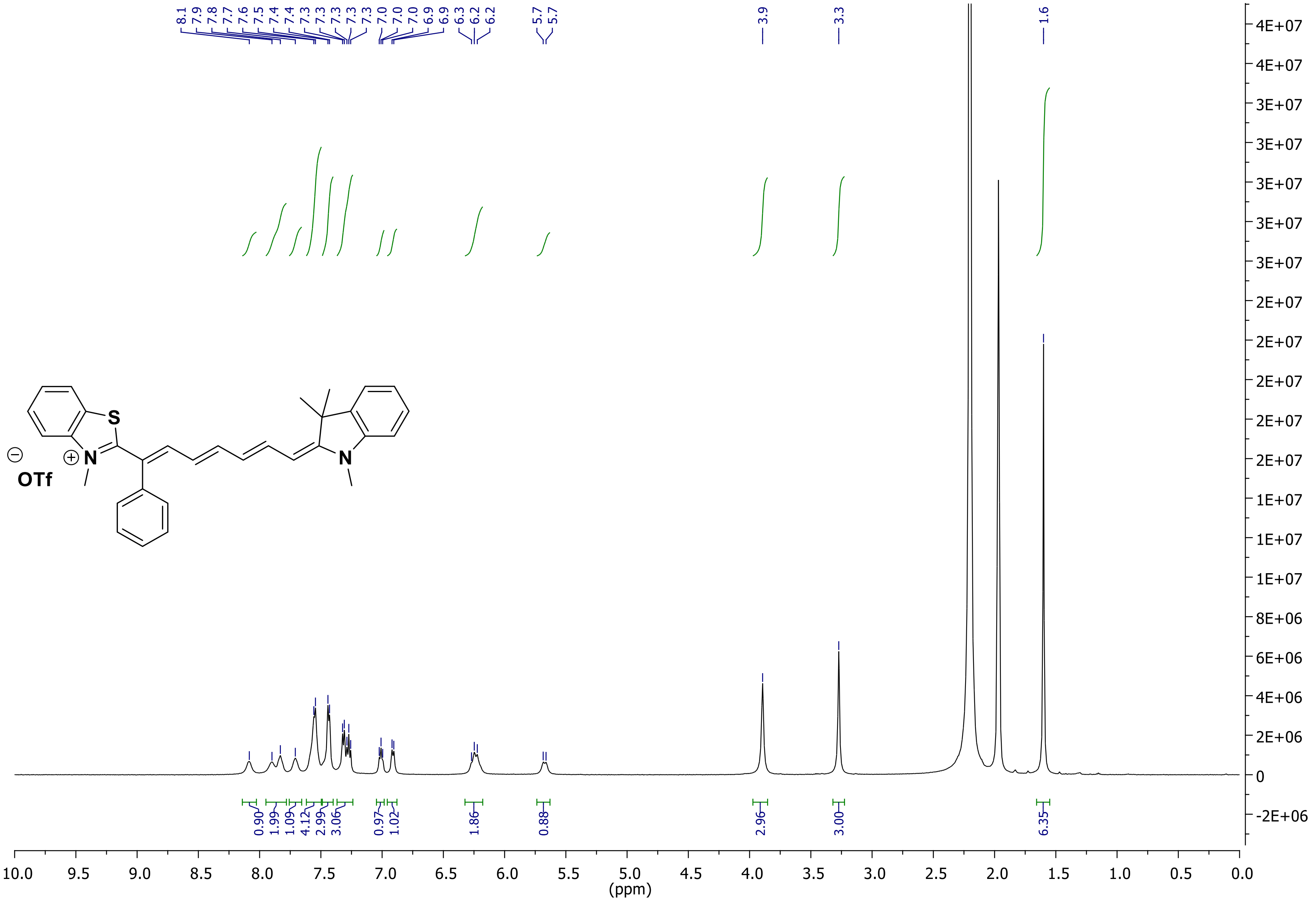

Figure S64. ^{1}H NMR (500 MHz, d_3-CD_3CN): **Cy7-5**.

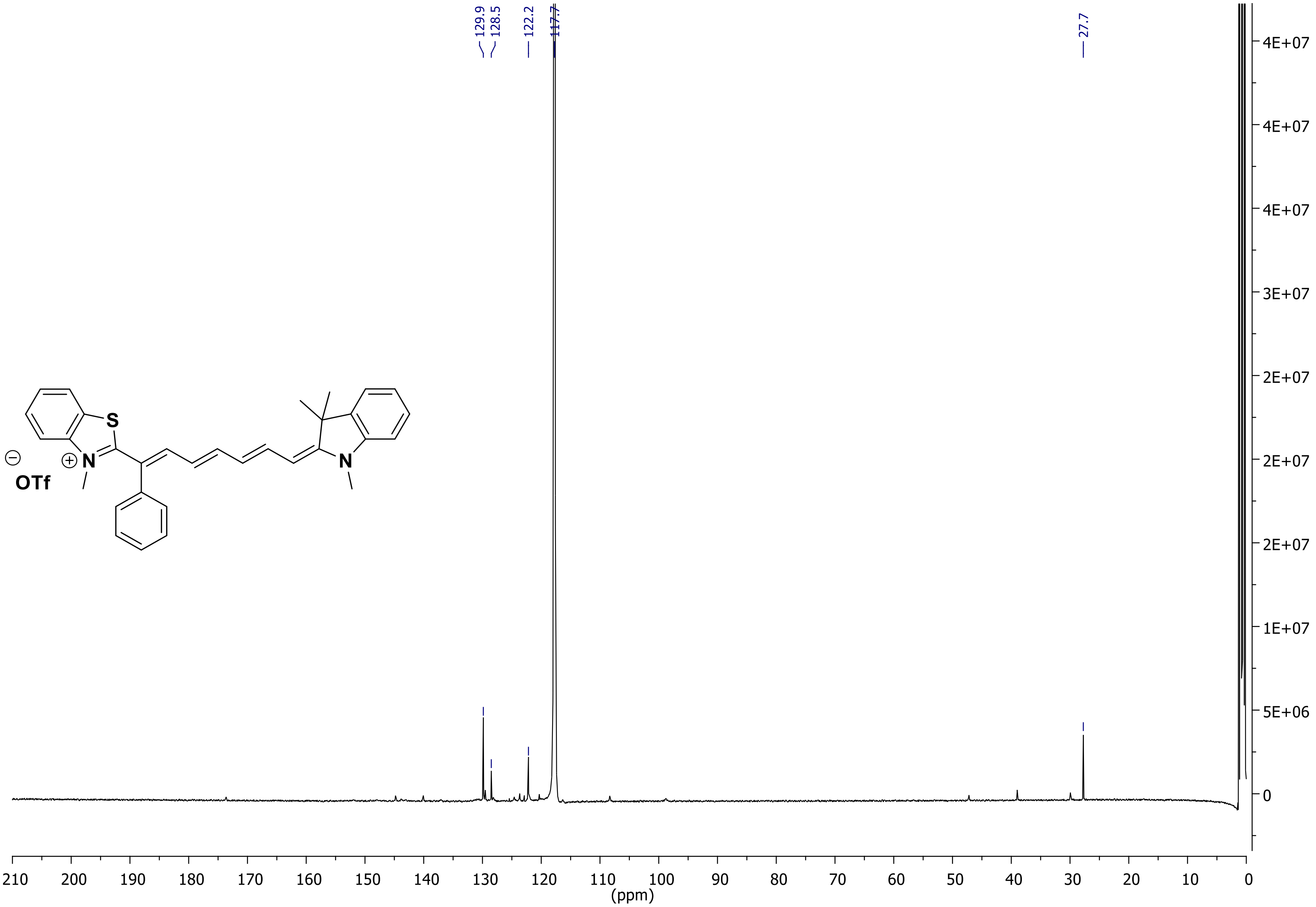

Figure S65. $^{13}C\{^{1}H\}$ NMR (126 MHz, d_3-CD_3CN): **Cy7-5**.

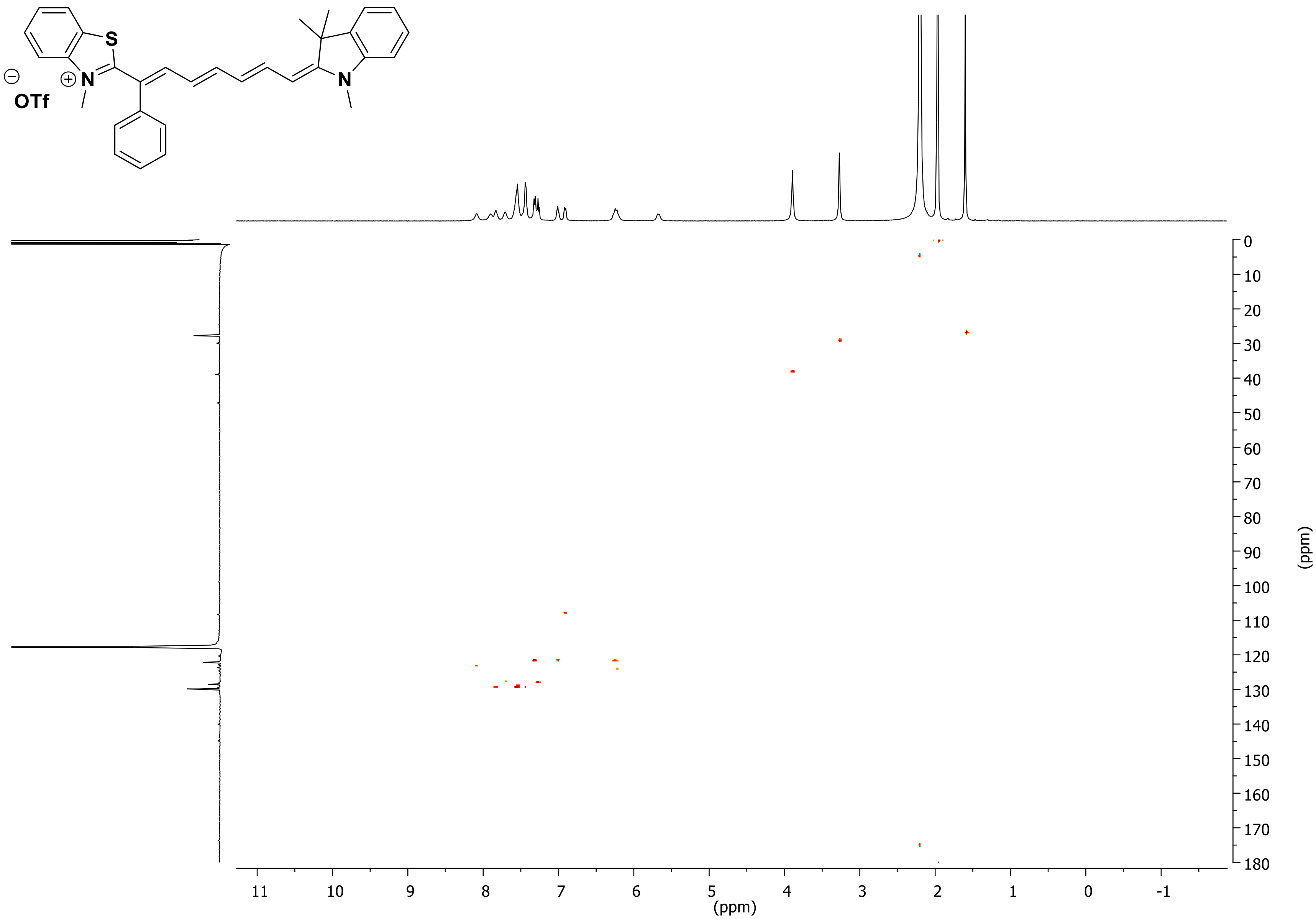

Figure S66. ^{1}H–^{13}C{^{1}H} gHSQC (500 MHz, d_3-CD_3CN): **Cy7-5**.

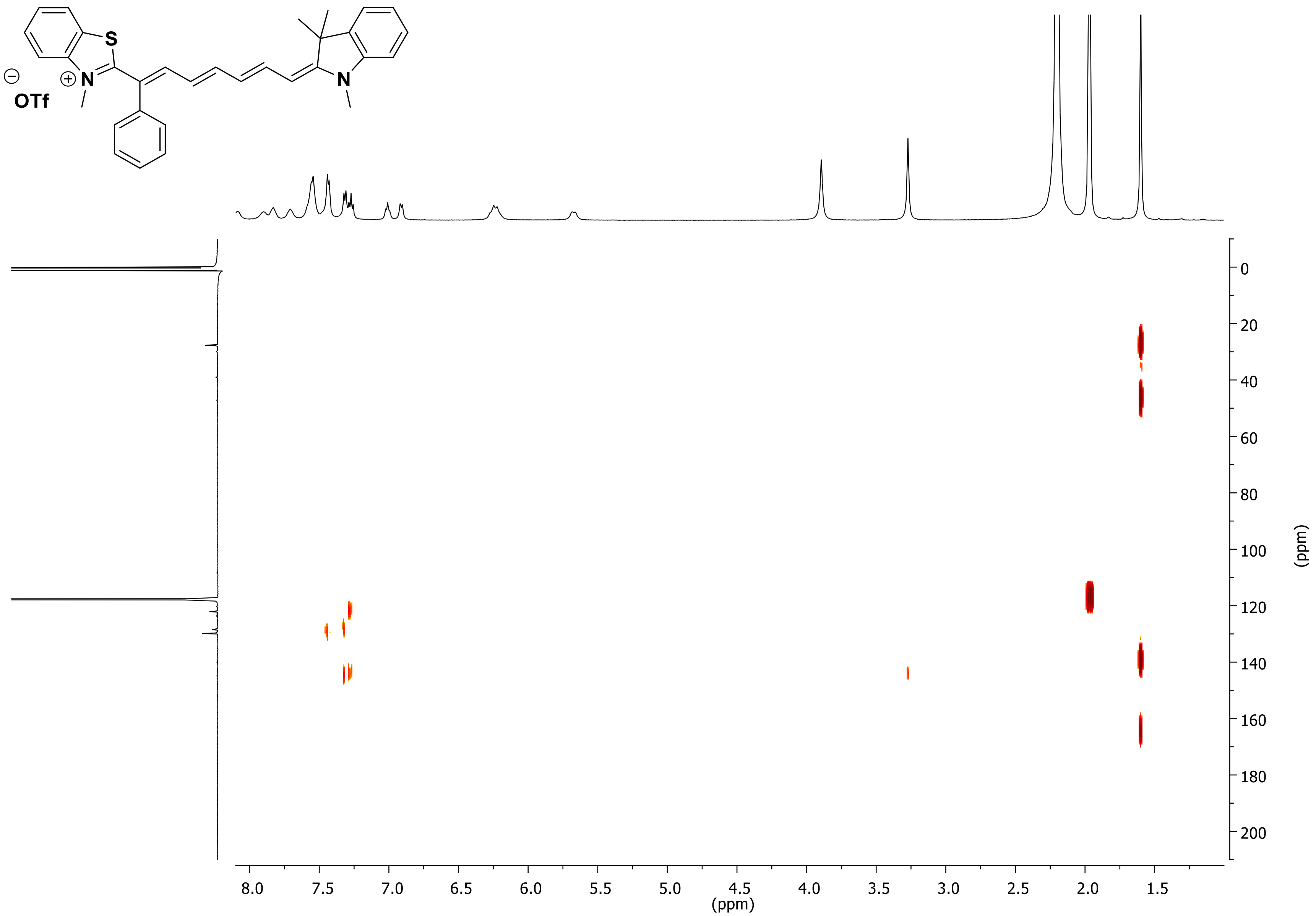

Figure S67. ^{1}H–$^{13}C\{^{1}H\}$ gHMBC (500 MHz, d_3-CD_3CN): **Cy7-5**.

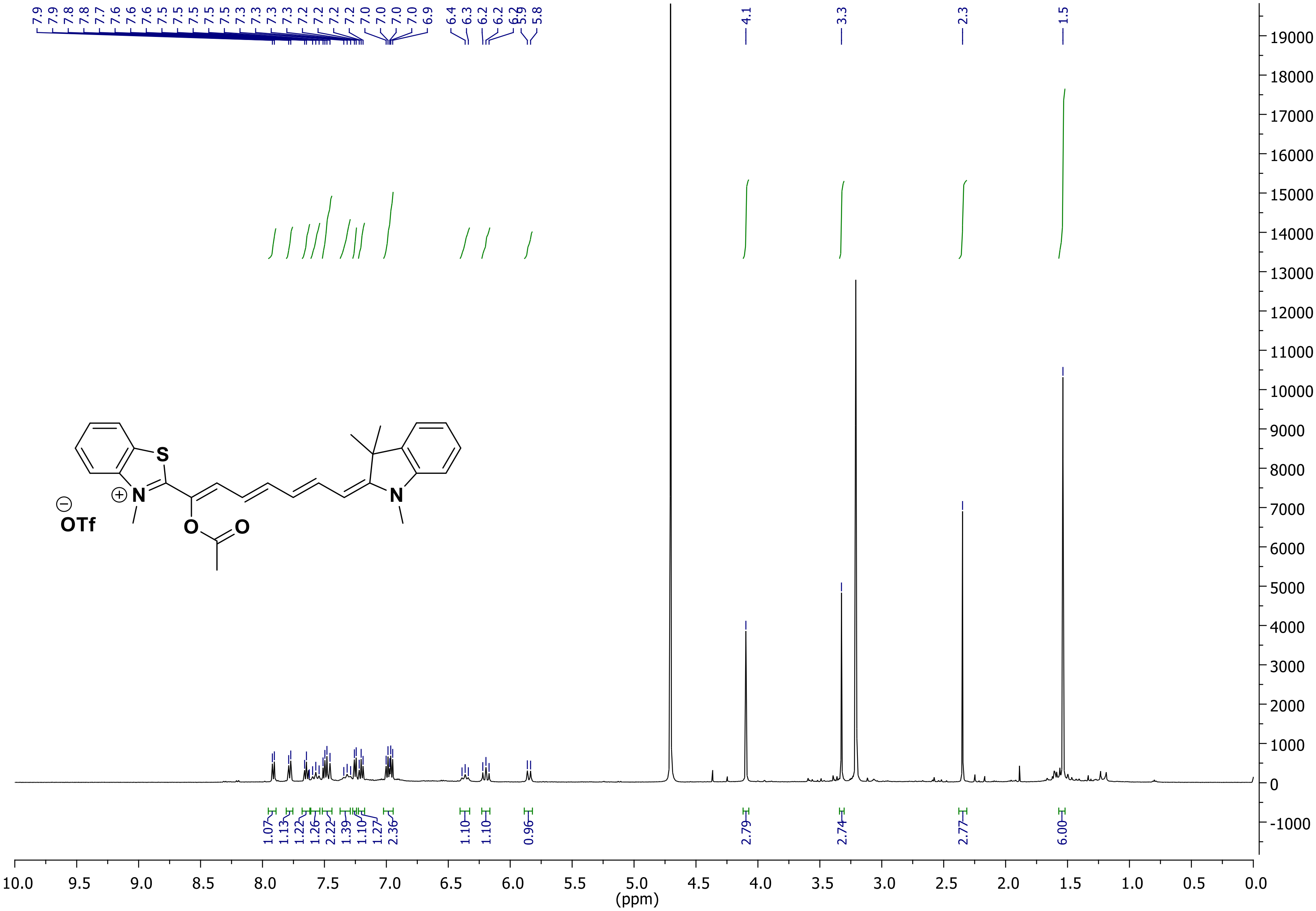

Figure S68. ^{1}H NMR (500 MHz, d_4–CD_3OD): **Cy7-6**.

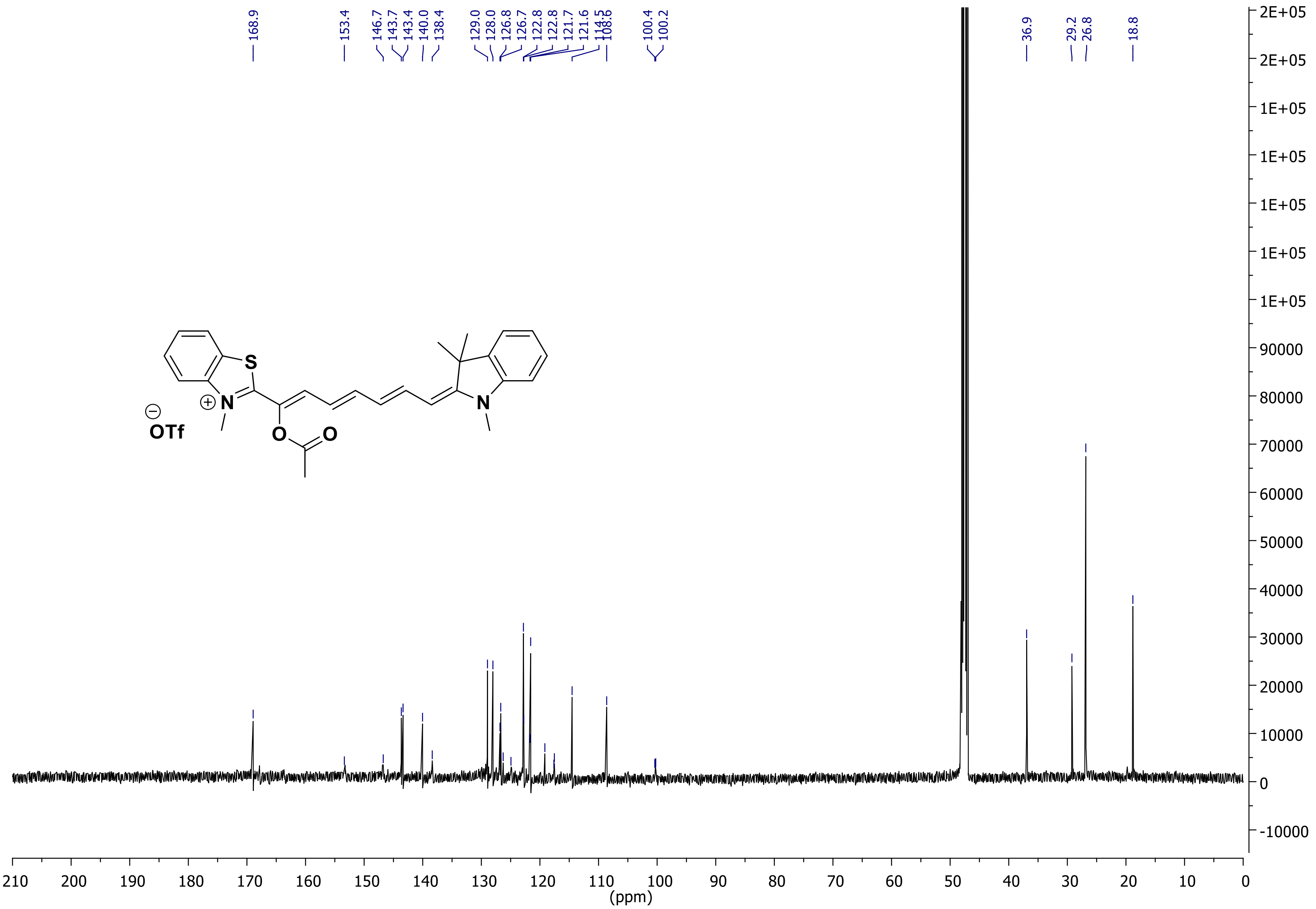

Figure S69. $^{13}C\{^{1}H\}$ NMR (126 MHz, d_4–CD_3OD): **Cy7-6**.

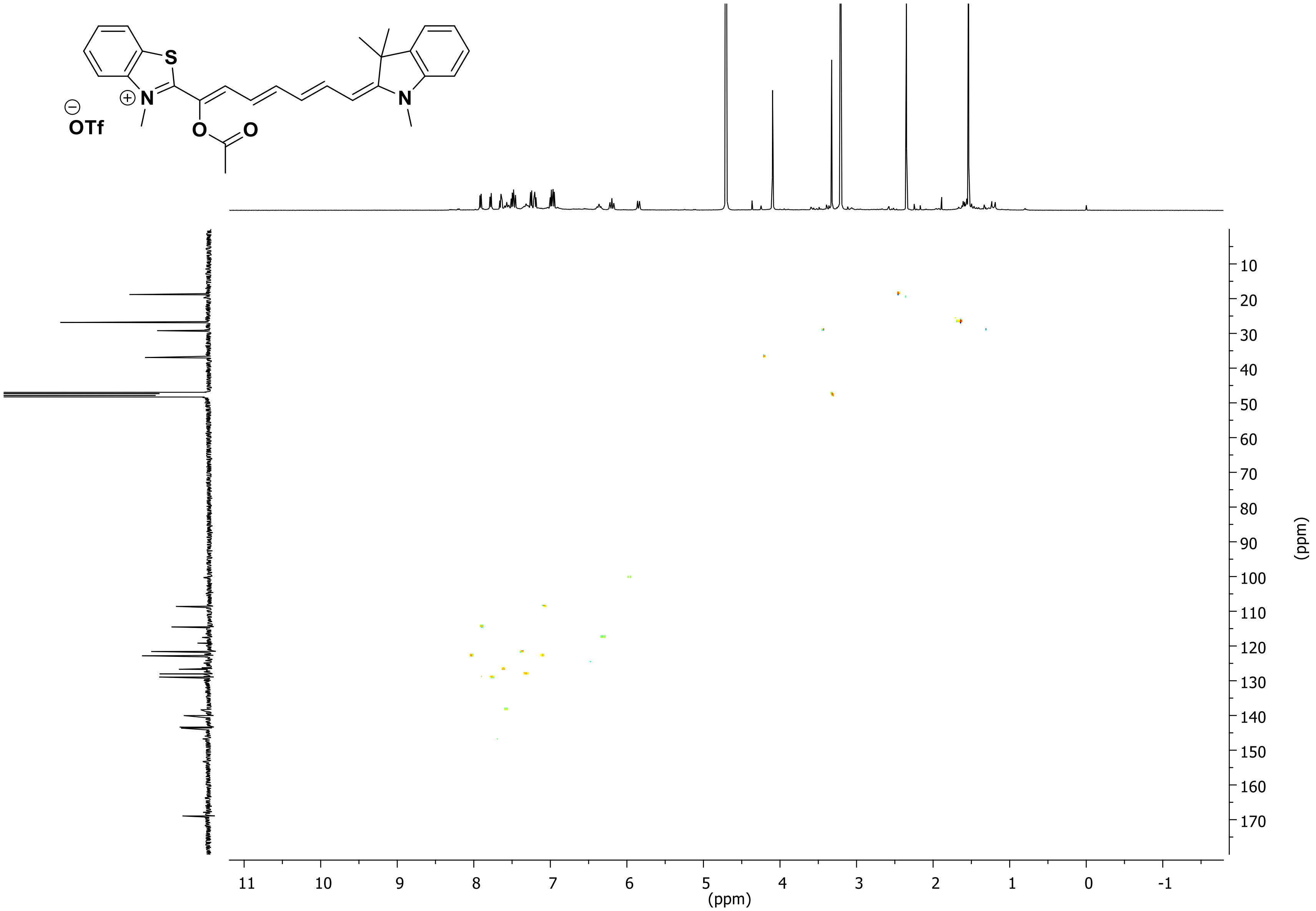

Figure S70. ^{1}H–$^{13}C\{^{1}H\}$ gHSQC (500 MHz, d_4–CD_3OD): **Cy7-6**.

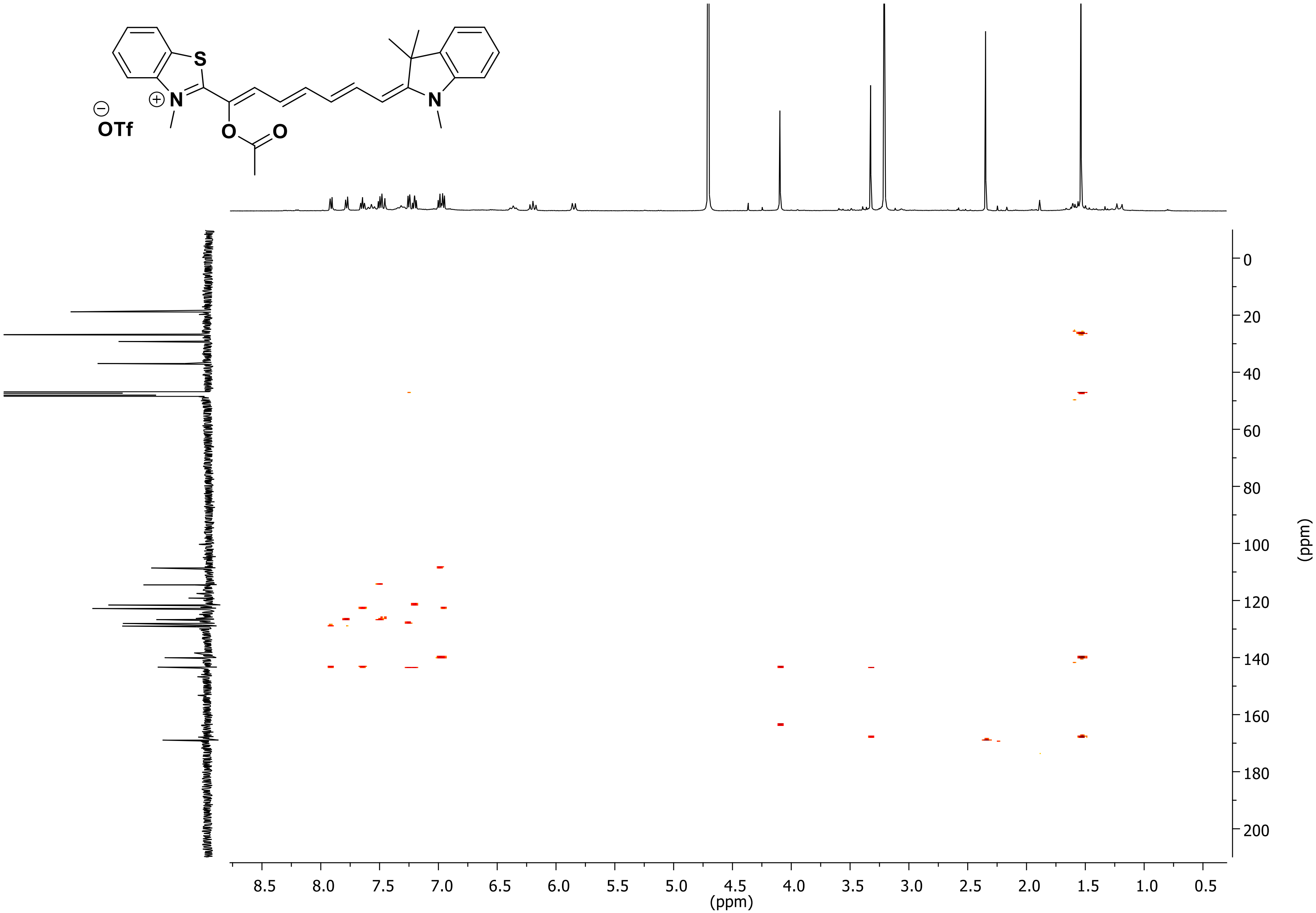

Figure S71. ^{1}H–$^{13}C\{^{1}H\}$ gHMBC (500 MHz, d_4–CD_3OD): **Cy7-6**.

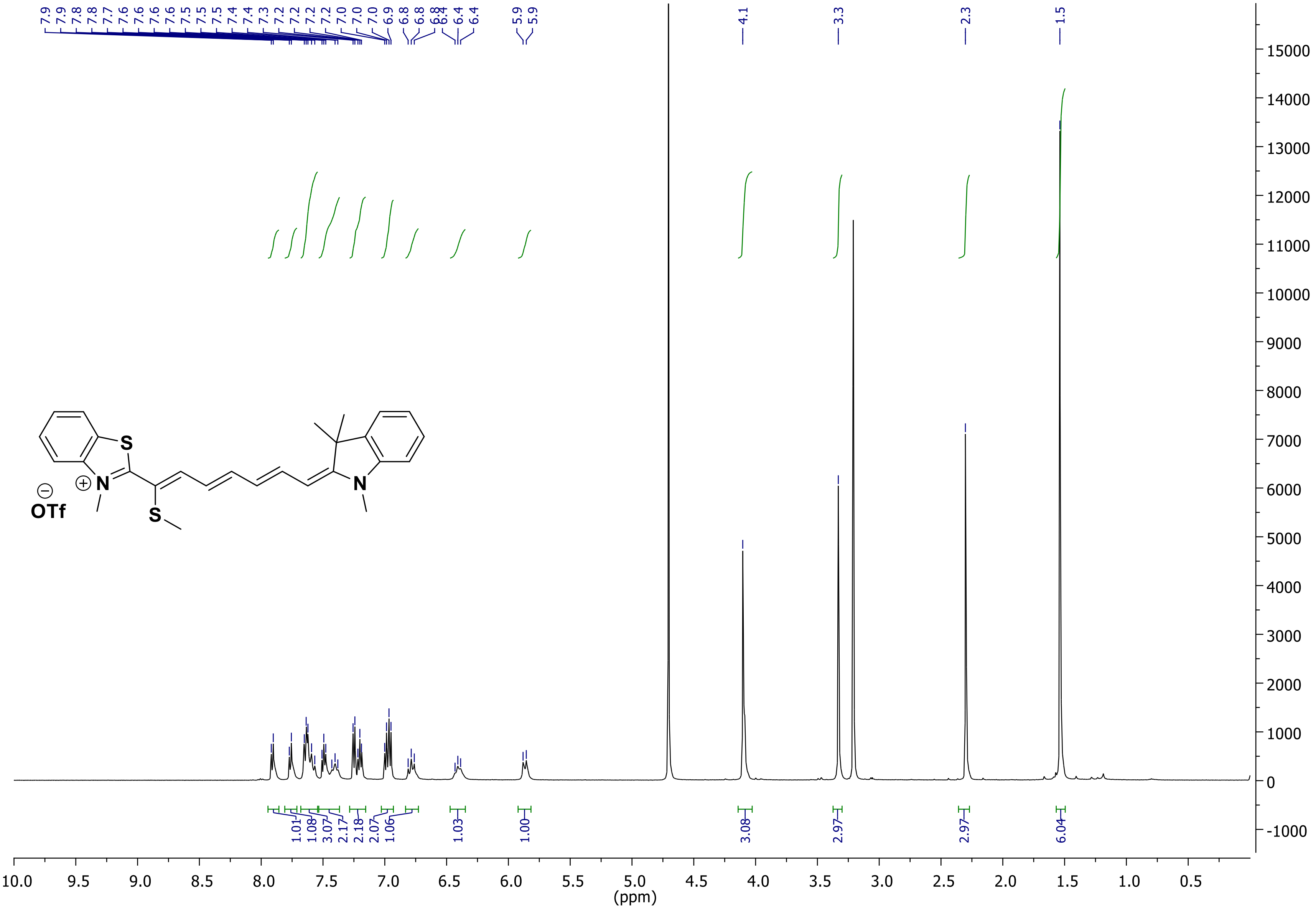

Figure S72. ^{1}H NMR (500 MHz, d_4–CD_3OD): **Cy7-7**.

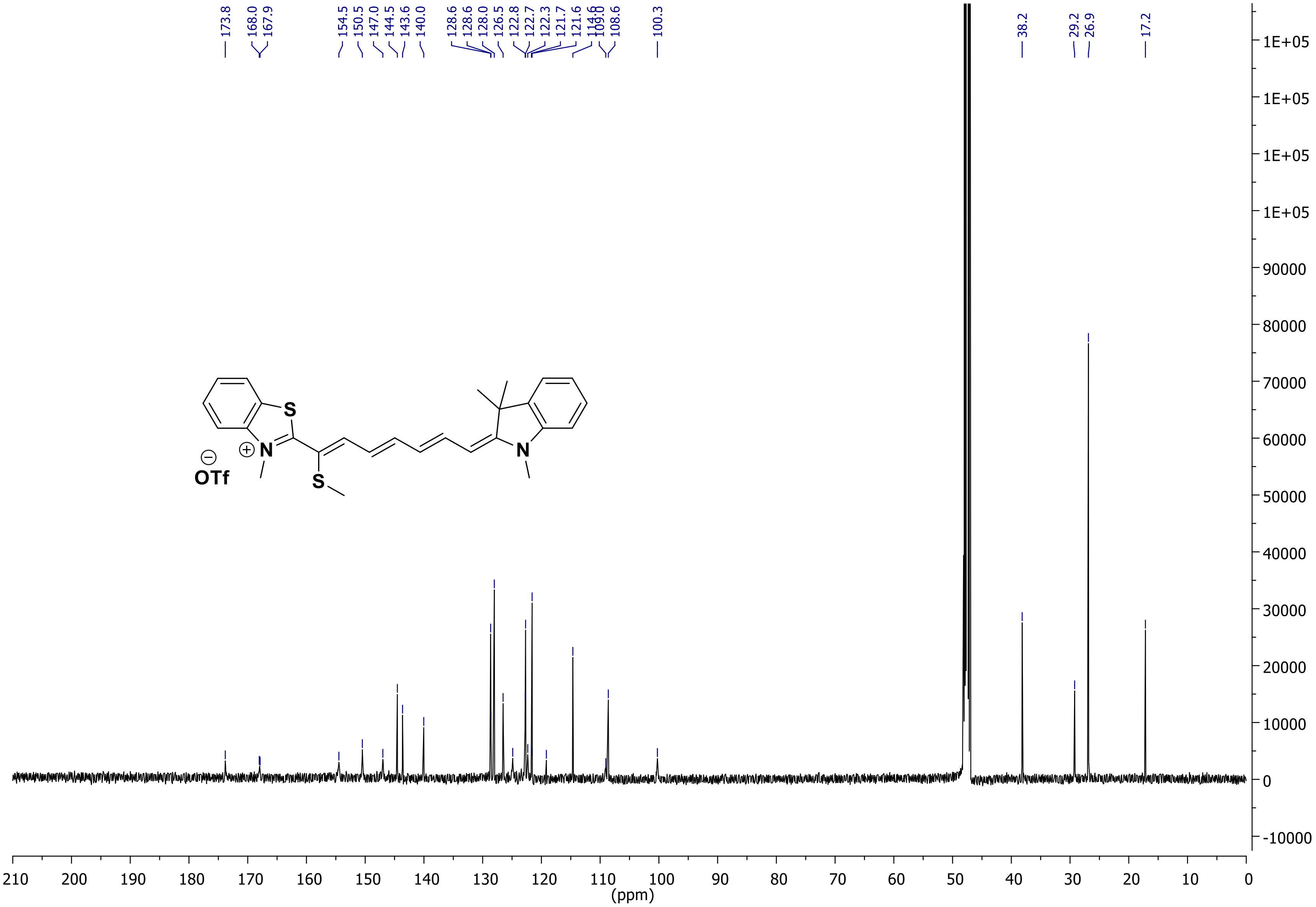

Figure S73. $^{13}C\{^1H\}$ NMR (126 MHz, d_4–CD_3OD): **Cy7-7**.

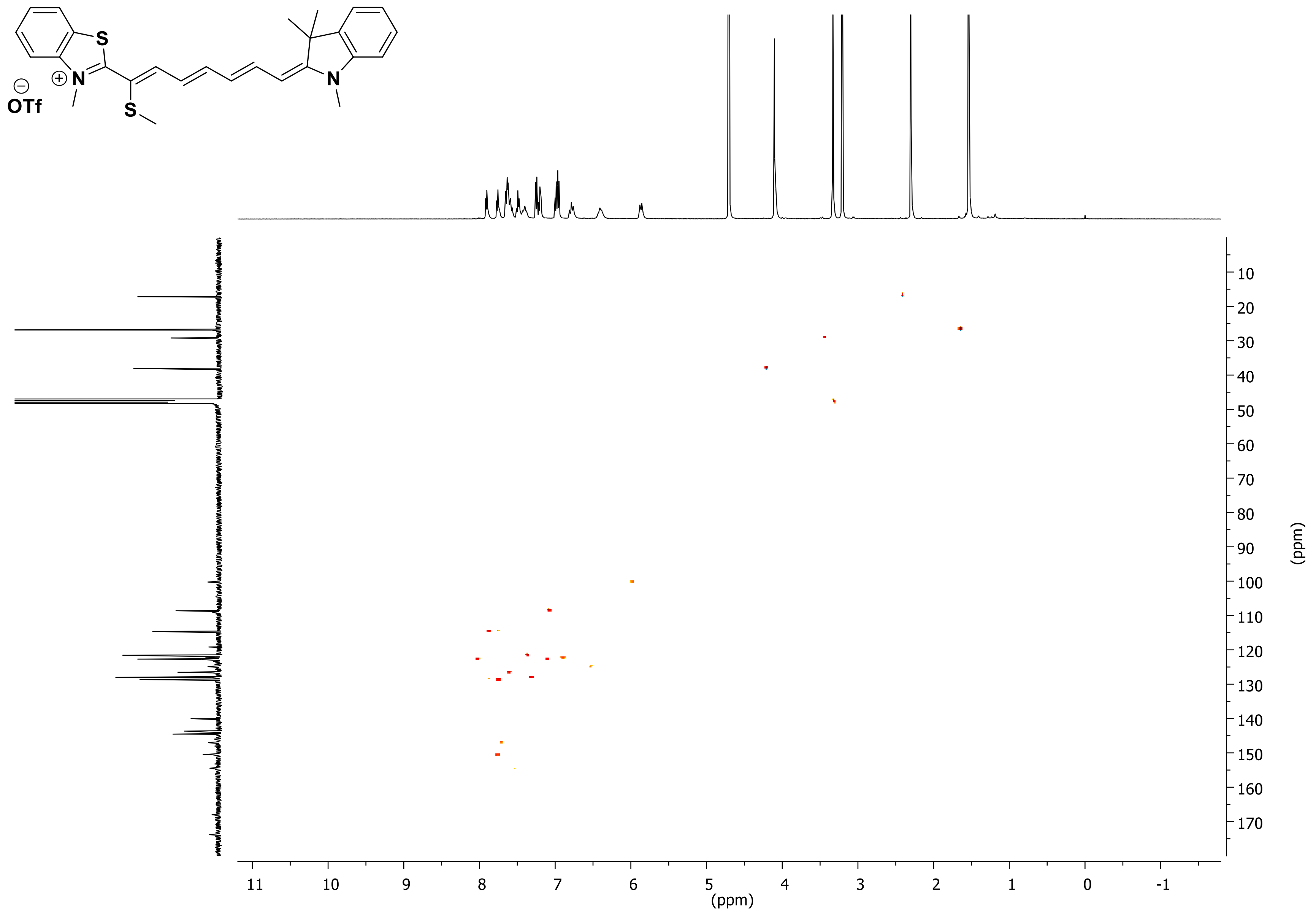

Figure S74. ^{1}H–$^{13}C\{^{1}H\}$ gHSQC (500 MHz, d_4–CD_3OD): **Cy7-7**.

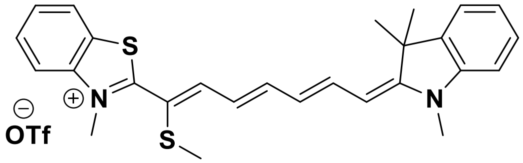

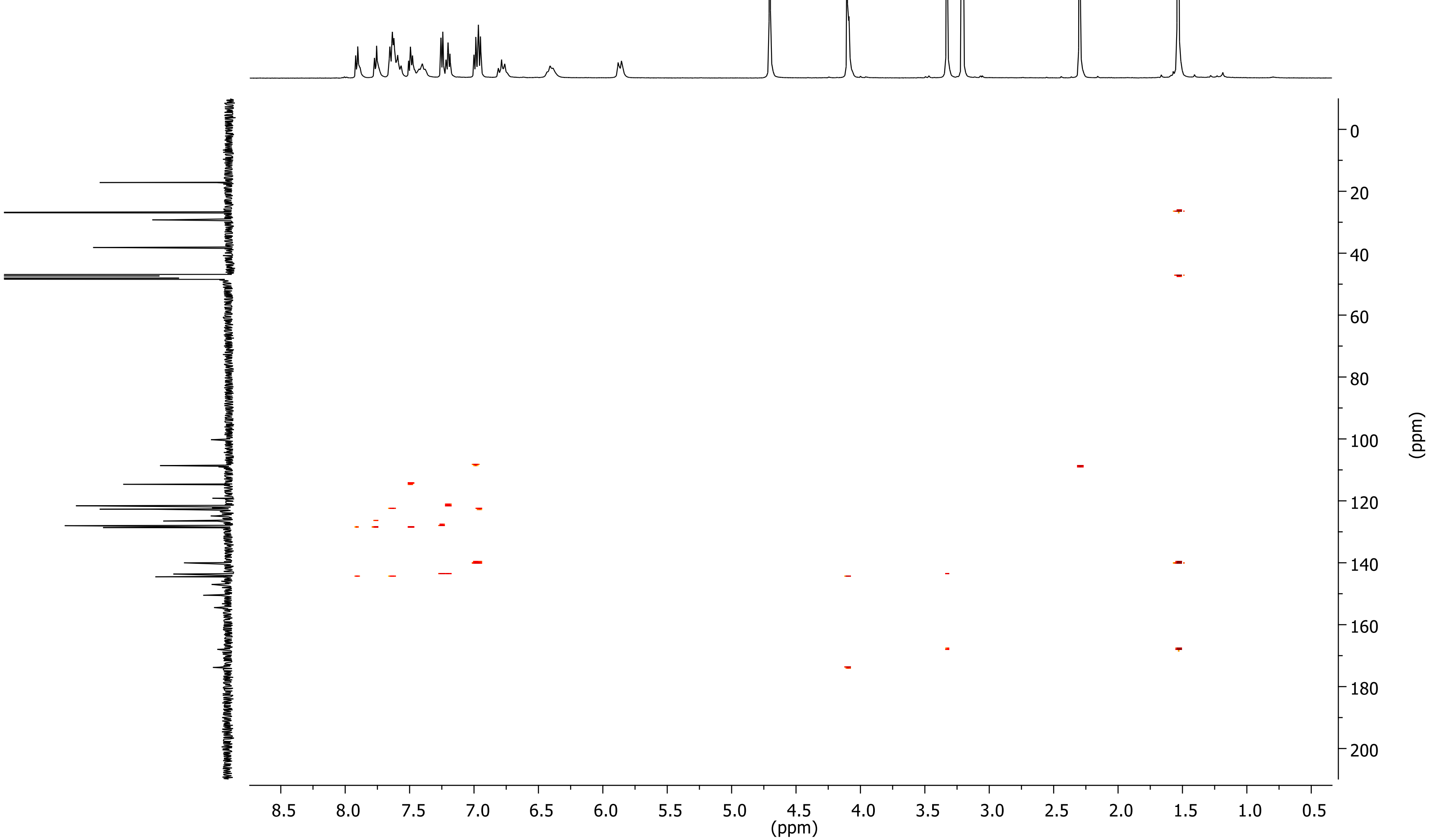

Figure S75. ^{1}H–$^{13}C\{^{1}H\}$ gHMBC (500 MHz, d_4–CD_3OD): **Cy7-7**.

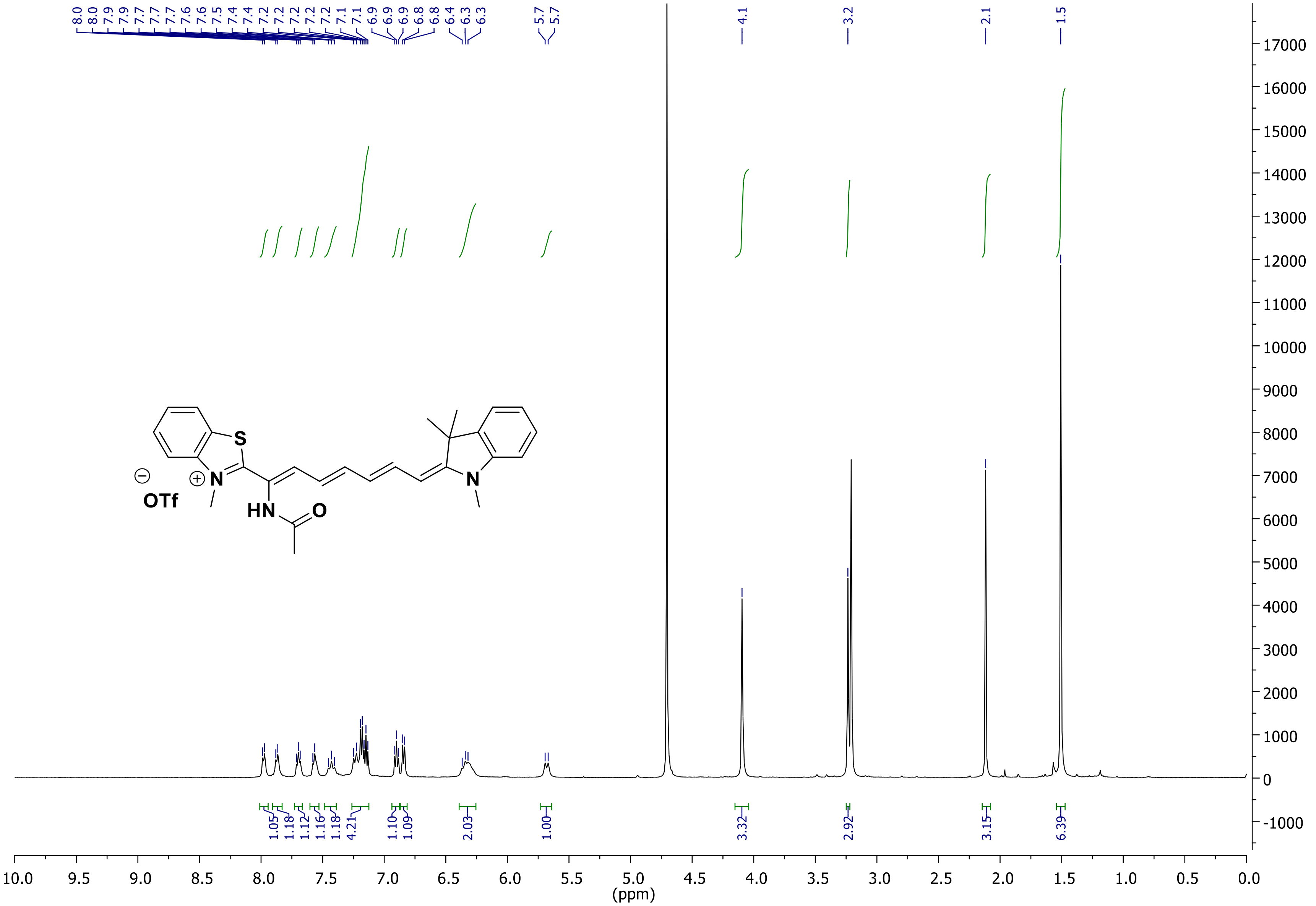

Figure S76. ^{1}H NMR (500 MHz, d_4–CD_3OD): **Cy7-8**.

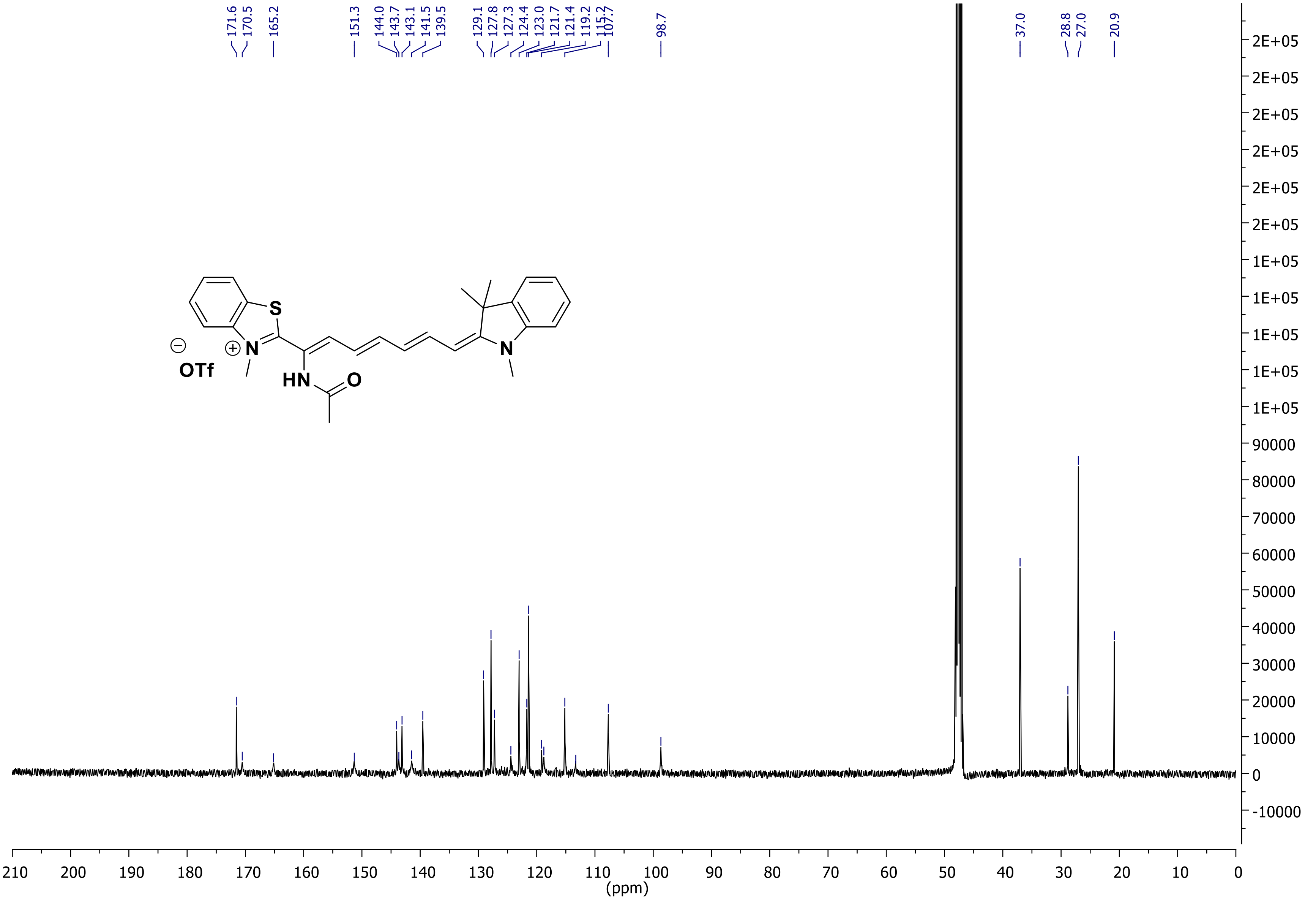

Figure S77. $^{13}C\{^{1}H\}$ NMR (126 MHz, d_4–CD_3OD): **Cy7-8**.

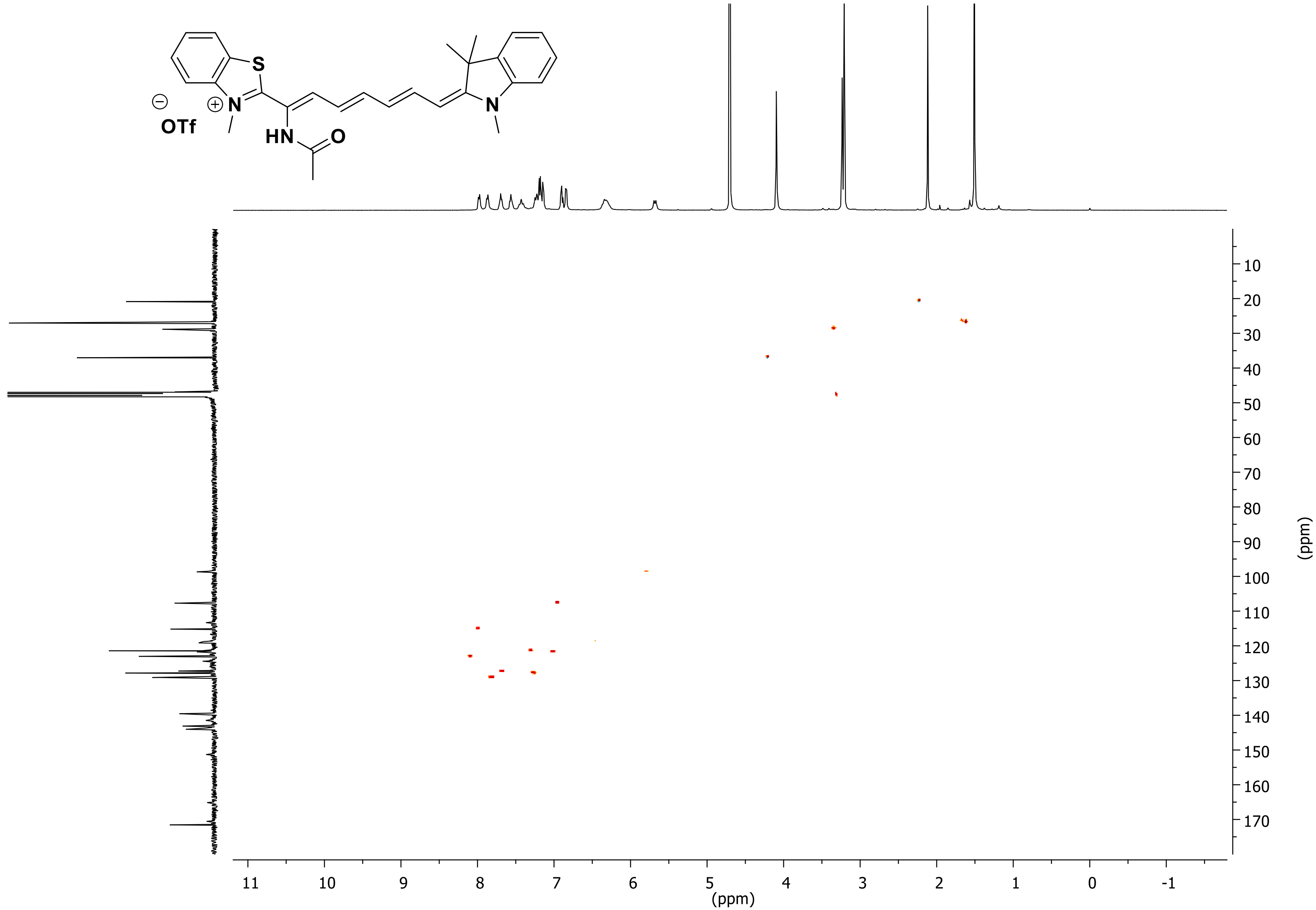

Figure S78. ^{1}H–$^{13}C\{^{1}H\}$ gHSQC (500 MHz, d_4–CD_3OD): **Cy7-8**.

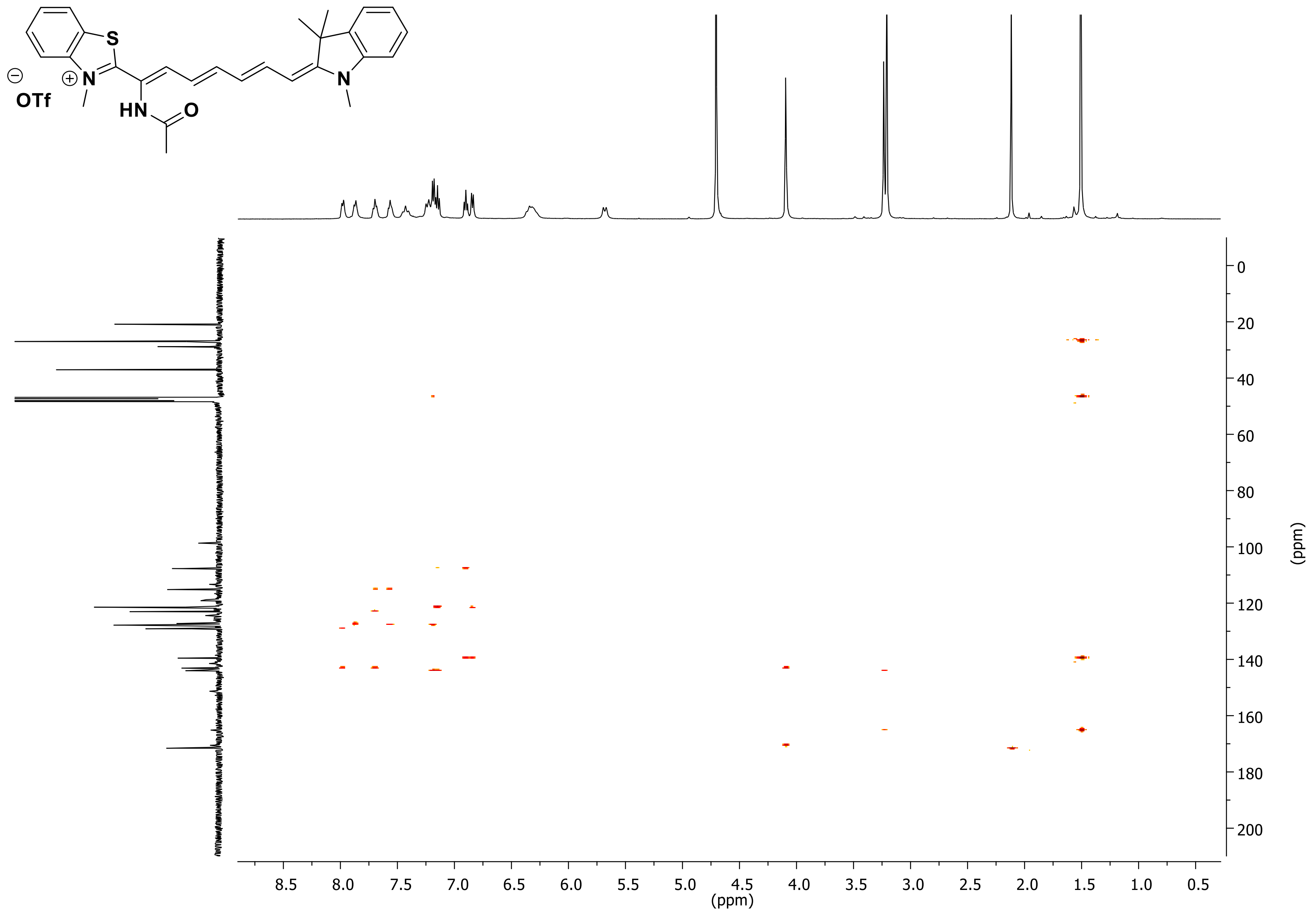

Figure S79. ^{1}H–$^{13}C\{^{1}H\}$ gHMBC (500 MHz, d_4–CD_3OD): **Cy7-8**.

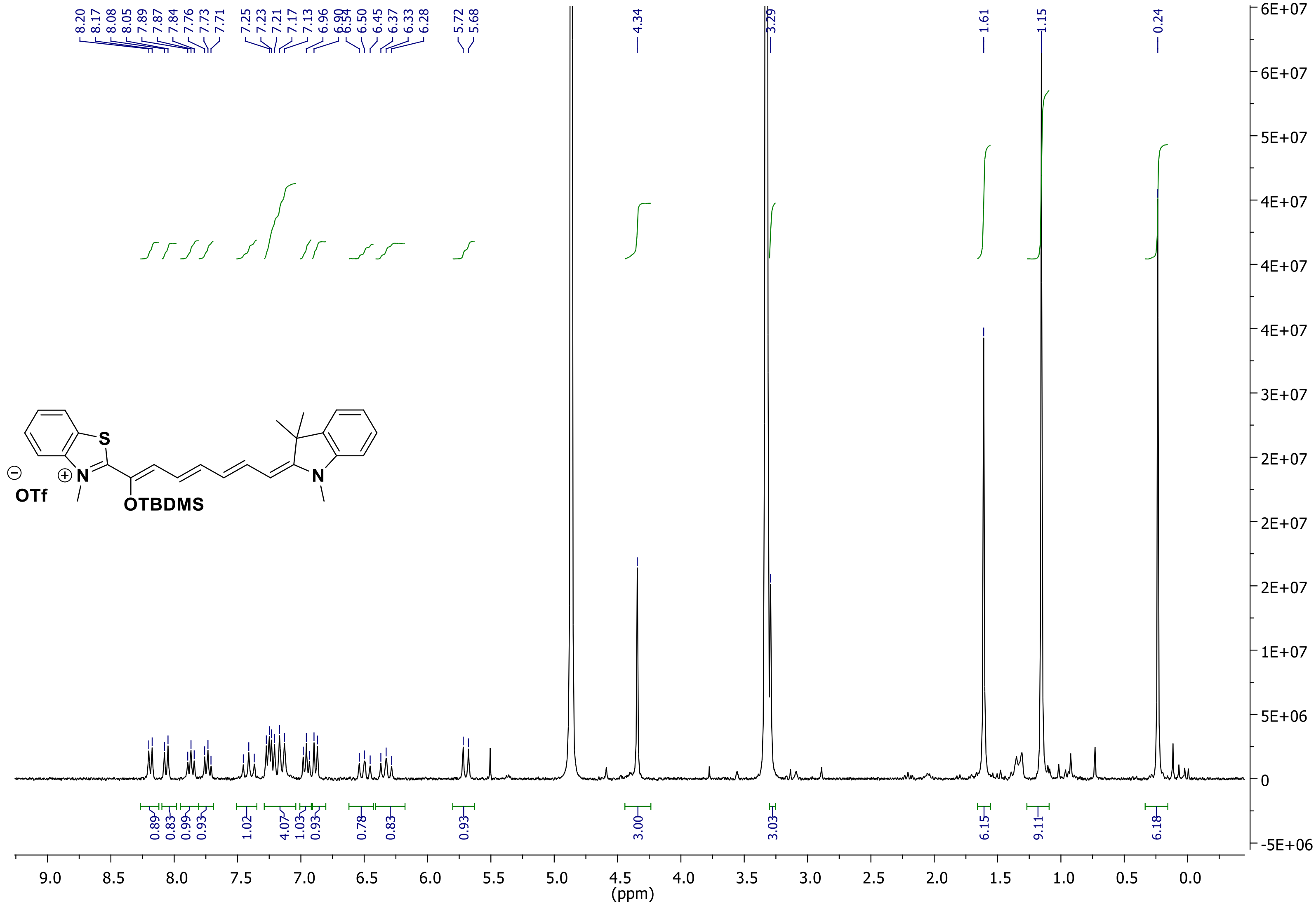

Figure S80. ^{1}H NMR (500 MHz, d_4–CD_3OD): **Cy7-9**.

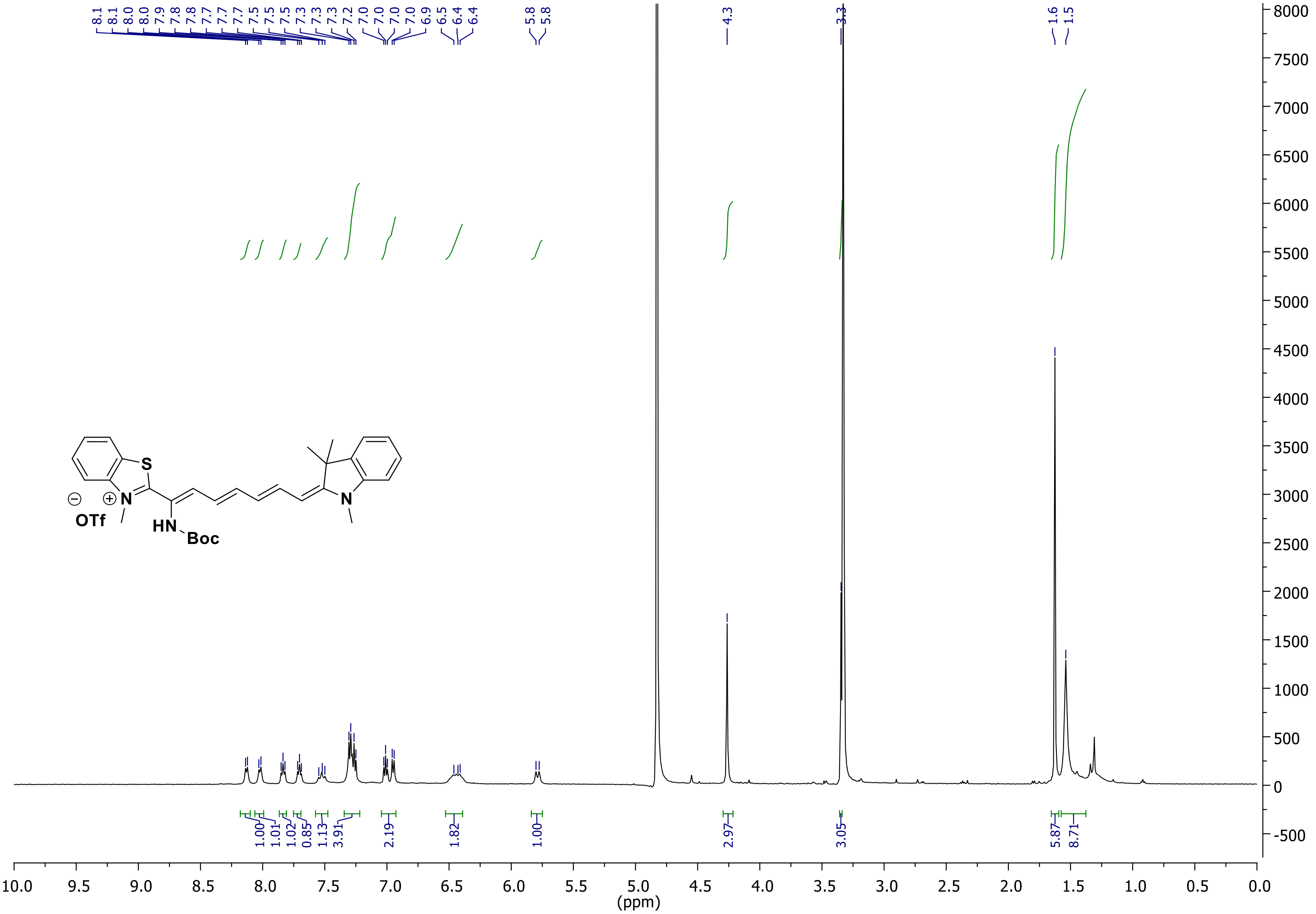

Figure S81. ^{1}H NMR (500 MHz, d_4–CD_3OD): **Cy7-10**.

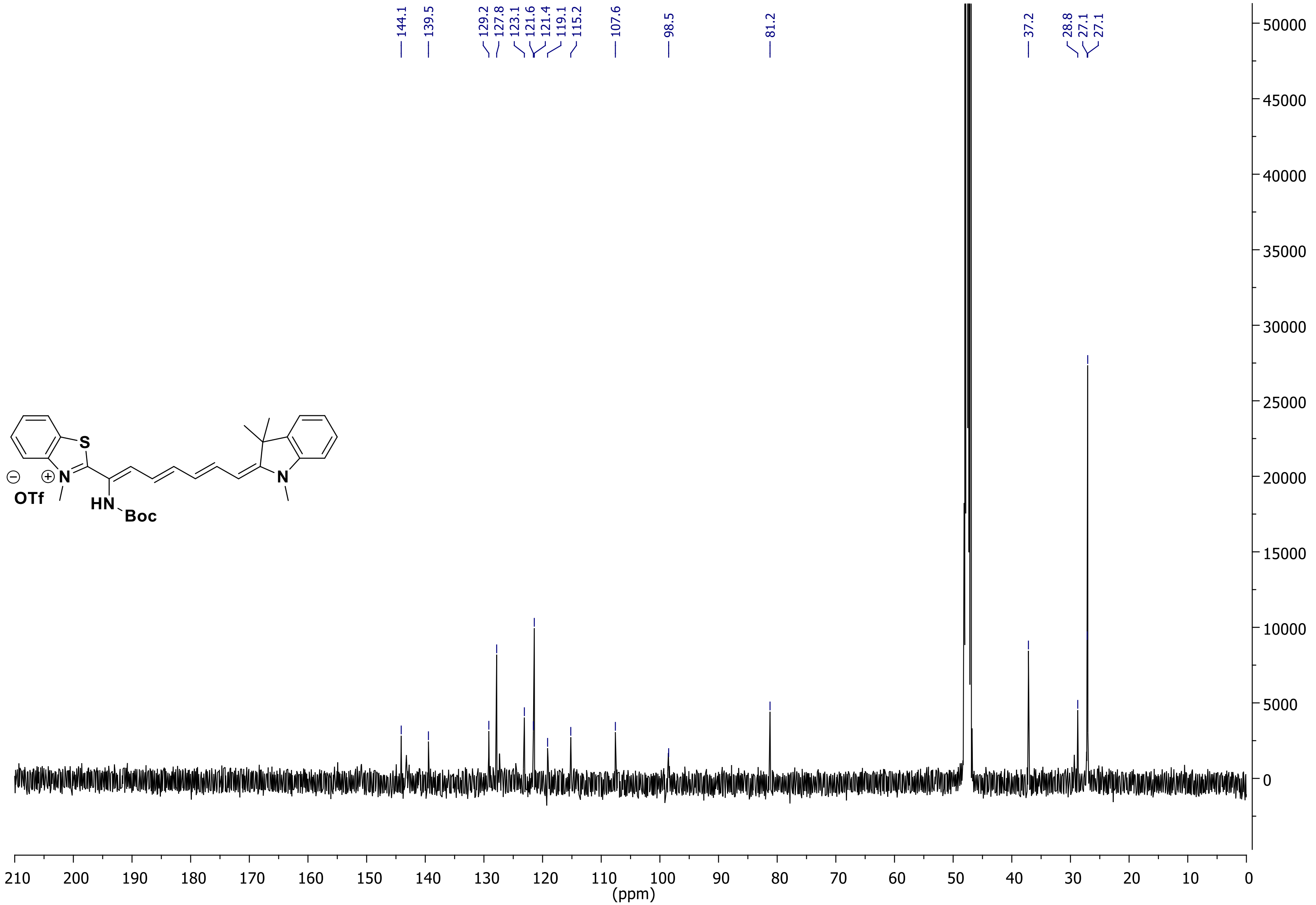

Figure S82. $^{13}C\{^{1}H\}$ NMR (126 MHz, d_4–CD_3OD): **Cy7-10**.

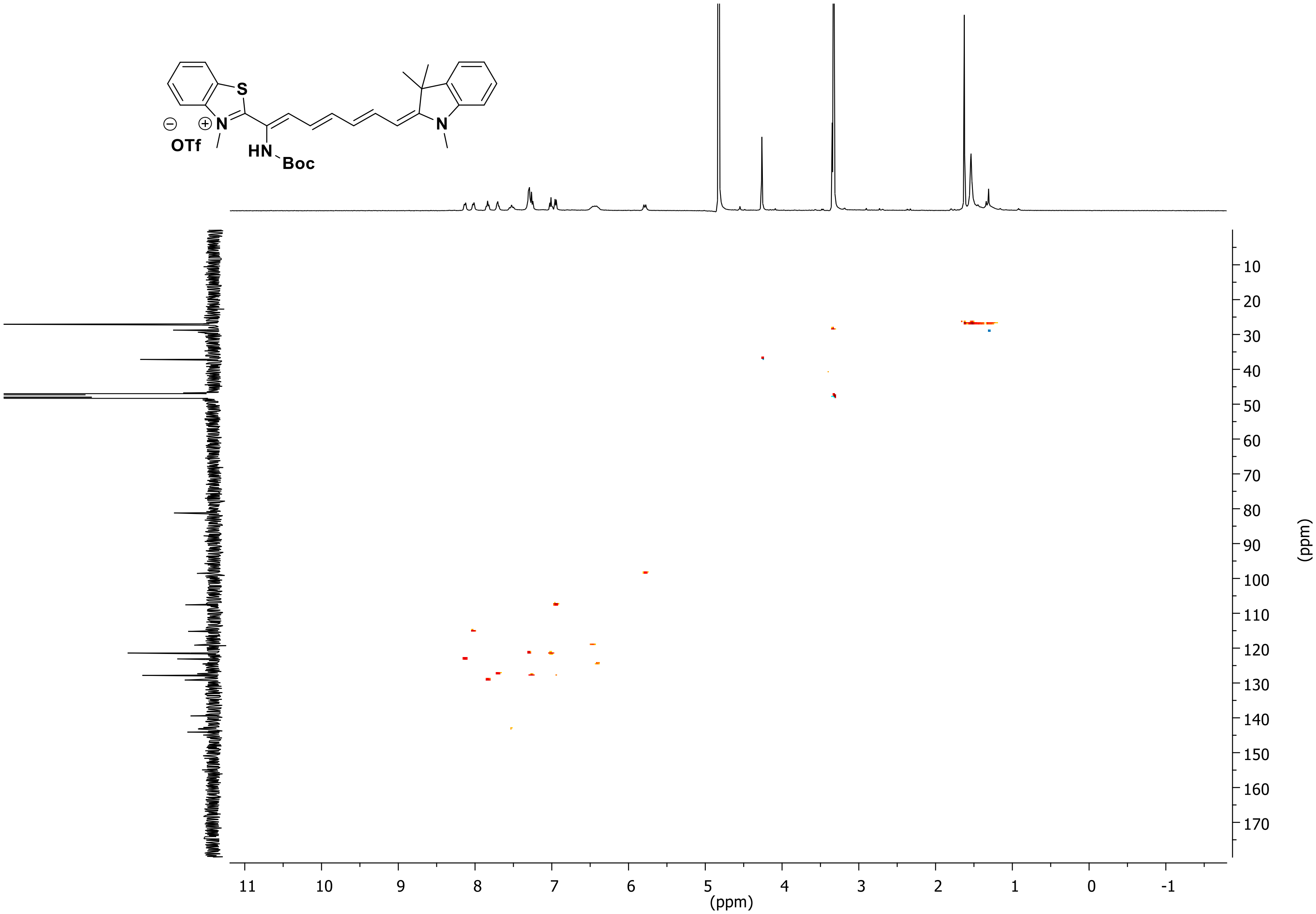

Figure S83. ^{1}H–$^{13}C\{^{1}H\}$ gHSQC (500 MHz, d_4–CD_3OD): **Cy7-10**.

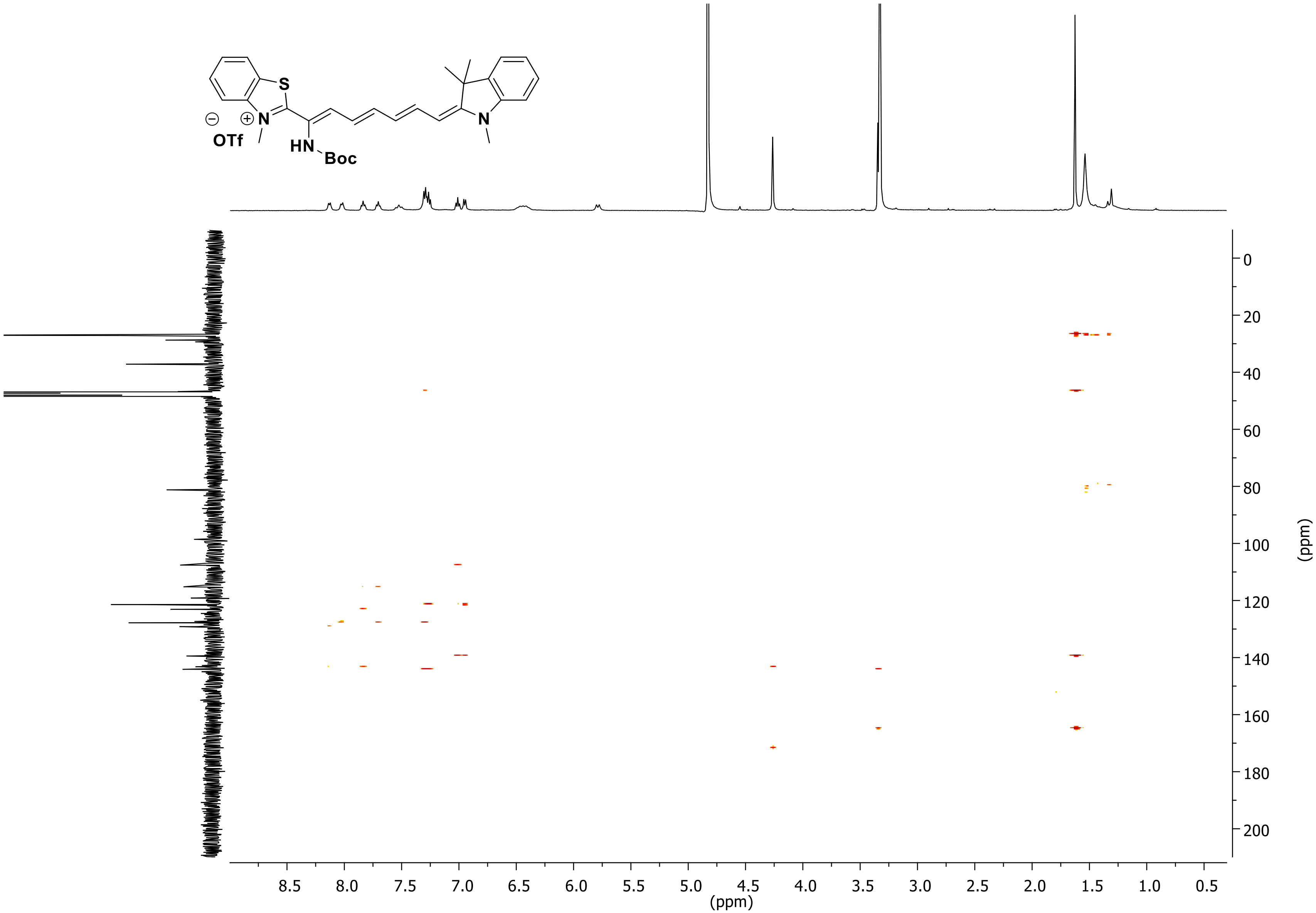

Figure S84. ^{1}H–$^{13}C\{^{1}H\}$ gHMBC (500 MHz, d_4–CD_3OD): **Cy7-10**.

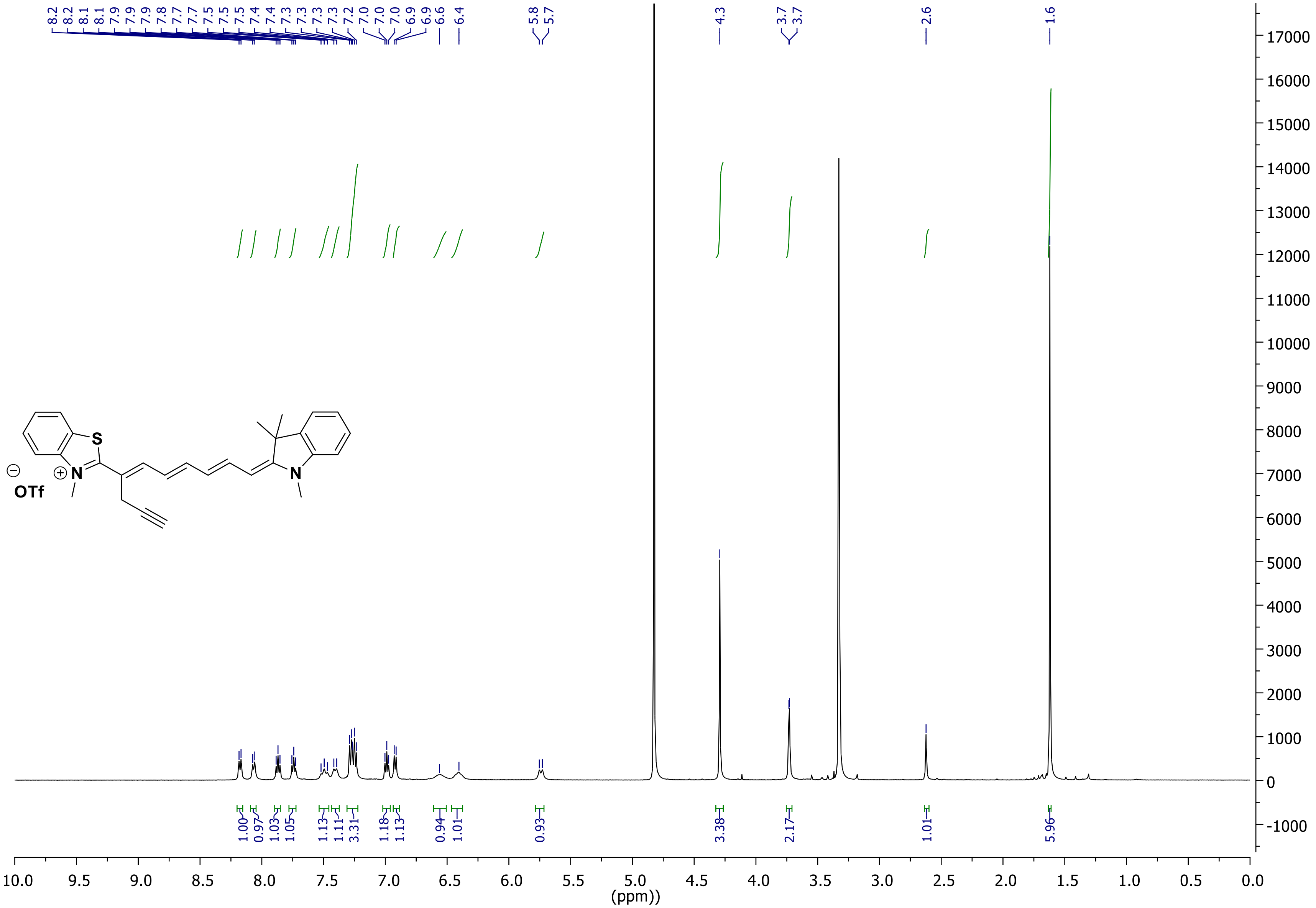

Figure S85. ^{1}H NMR (500 MHz, d_4–CD_3OD): **Cy7-11**.

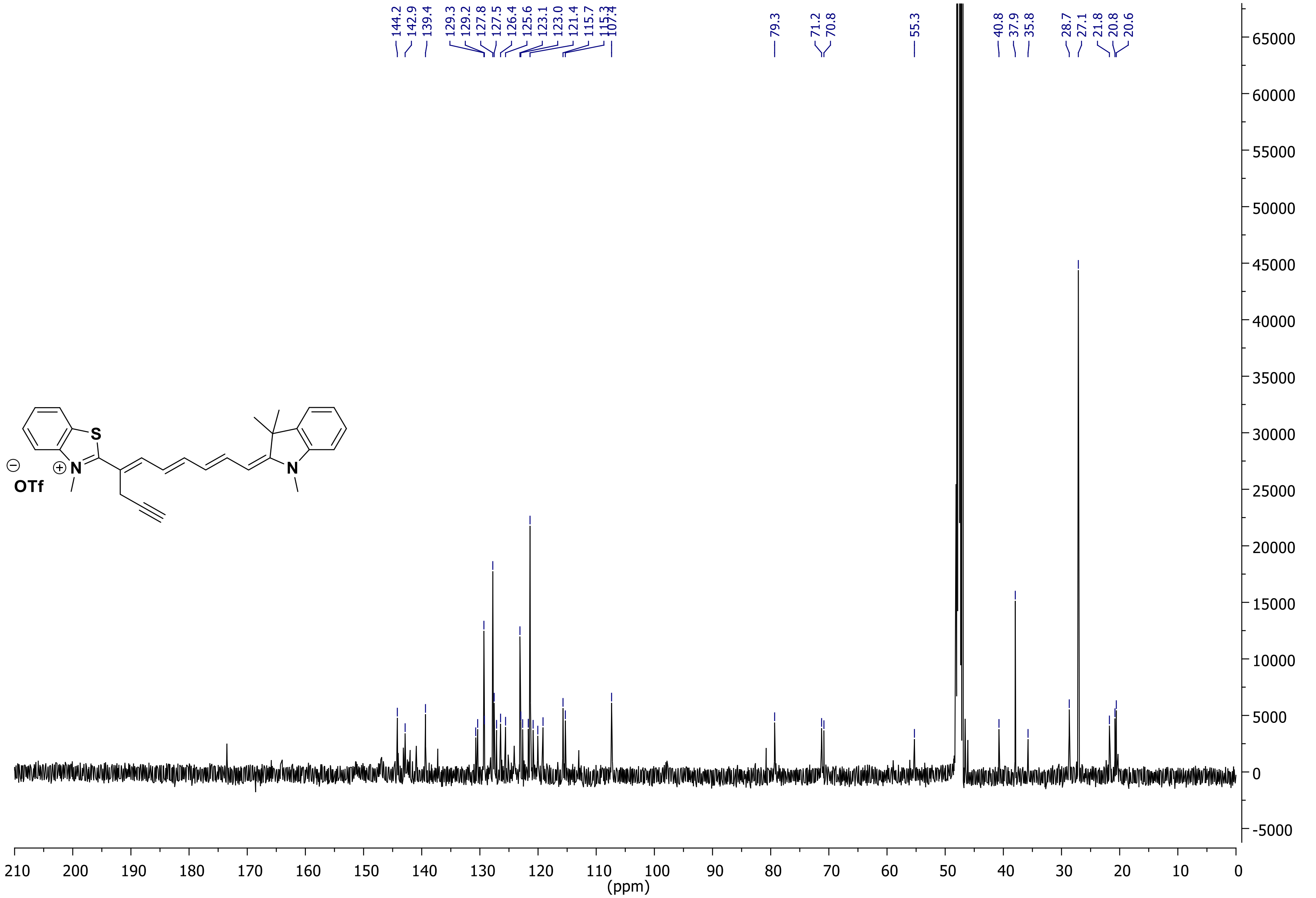

Figure S86. $^{13}C\{^{1}H\}$ NMR (126 MHz, d_4–CD_3OD): **Cy7-11**.

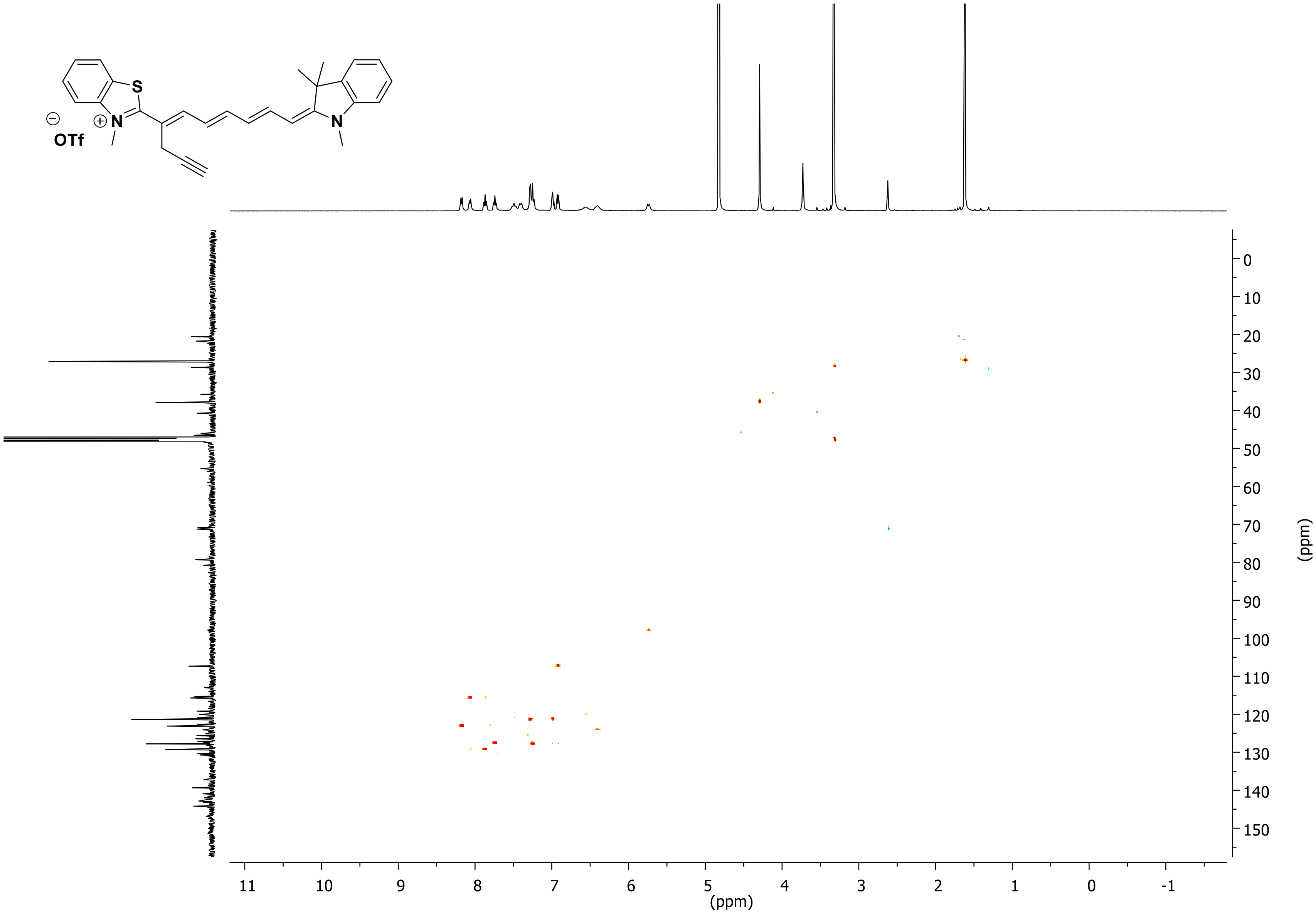

Figure S87. ^{1}H–$^{13}C\{^{1}H\}$ gHSQC (500 MHz, *d*$_4$–CD_3OD): **Cy7-11**.

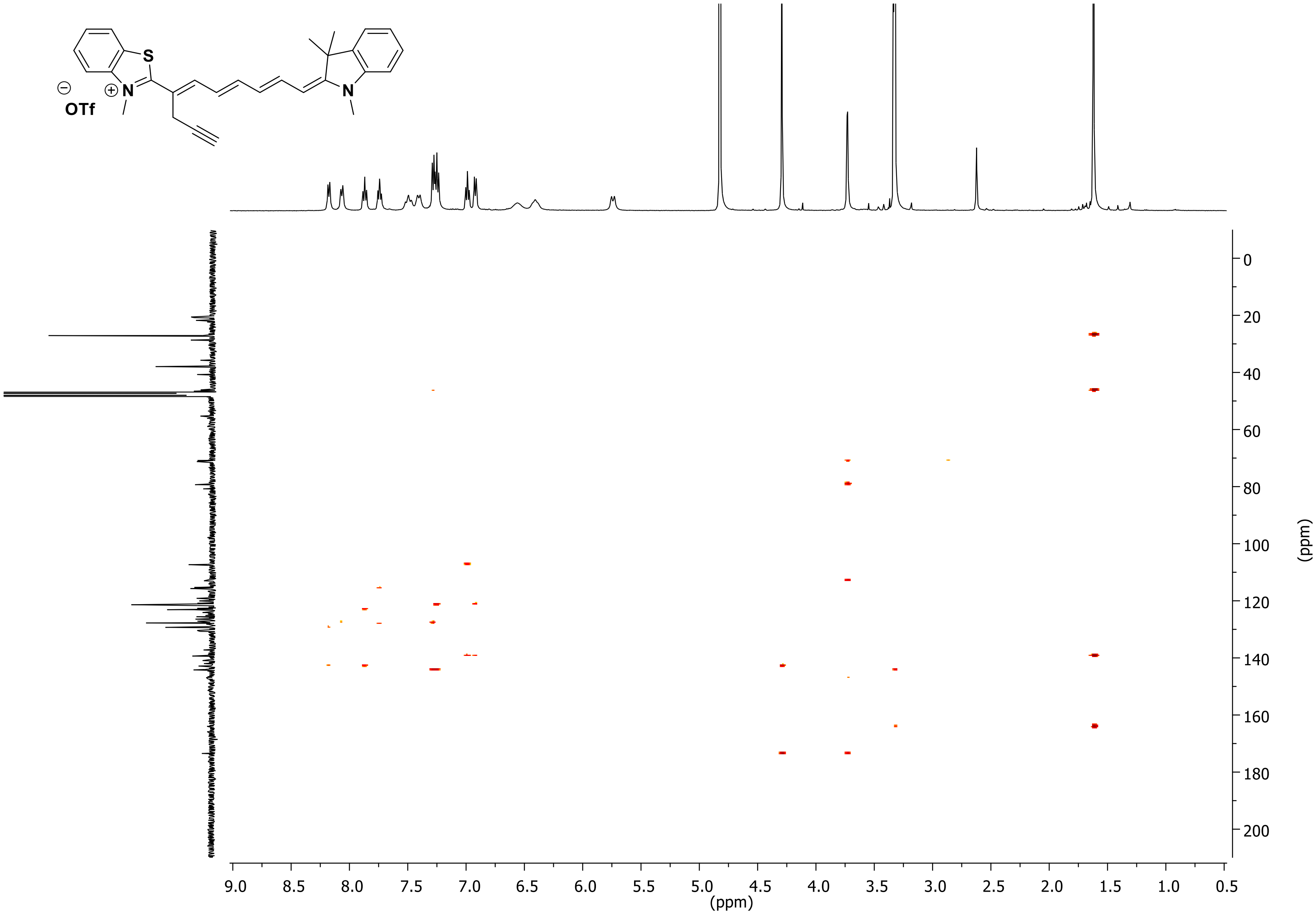

Figure S88. ^{1}H–$^{13}C\{^{1}H\}$ gHMBC (500 MHz, d_4–CD_3OD): **Cy7-11**.

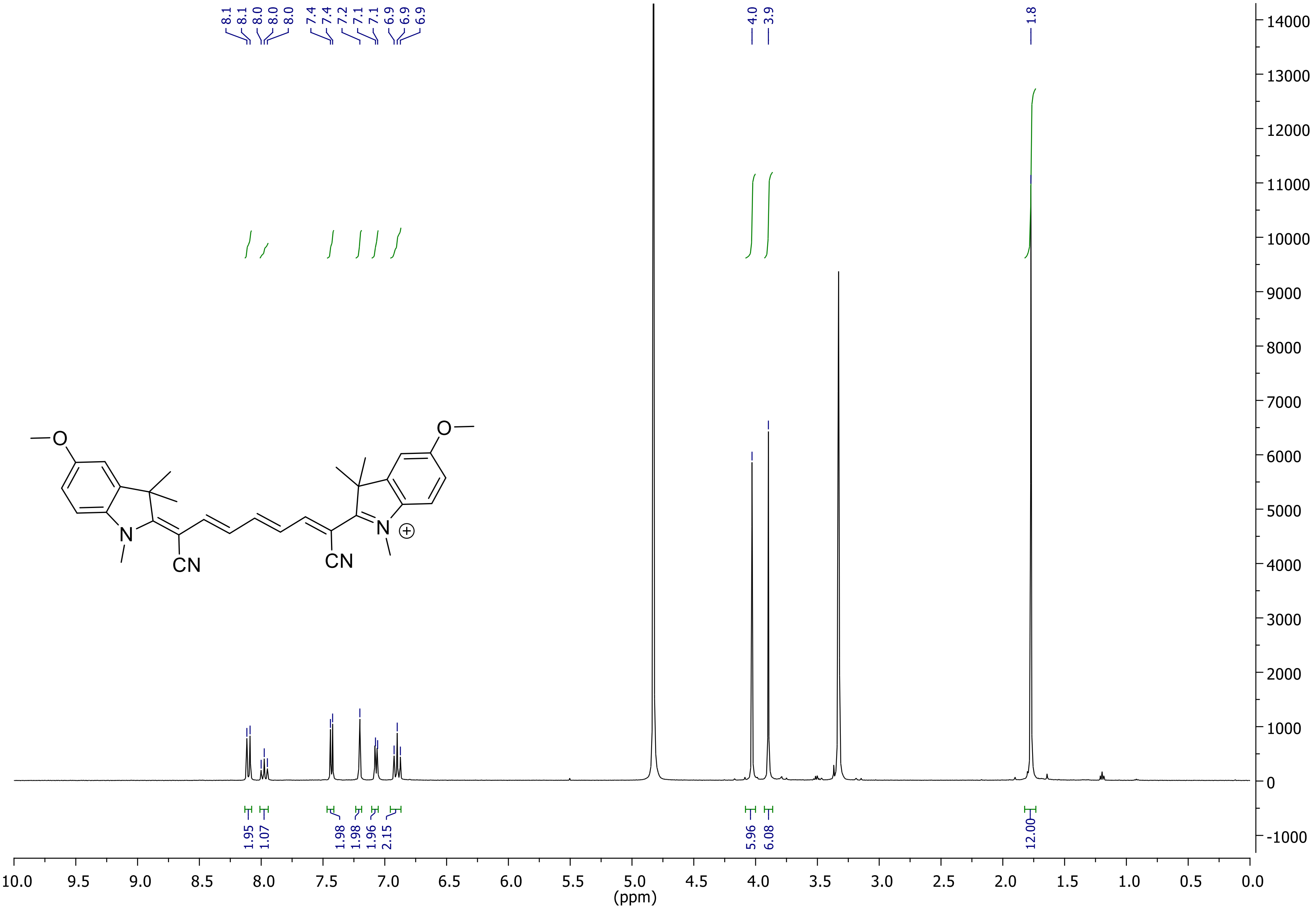

Figure S89. ^{1}H NMR (500 MHz, d_4–CD_3OD): **Cy7-13**.

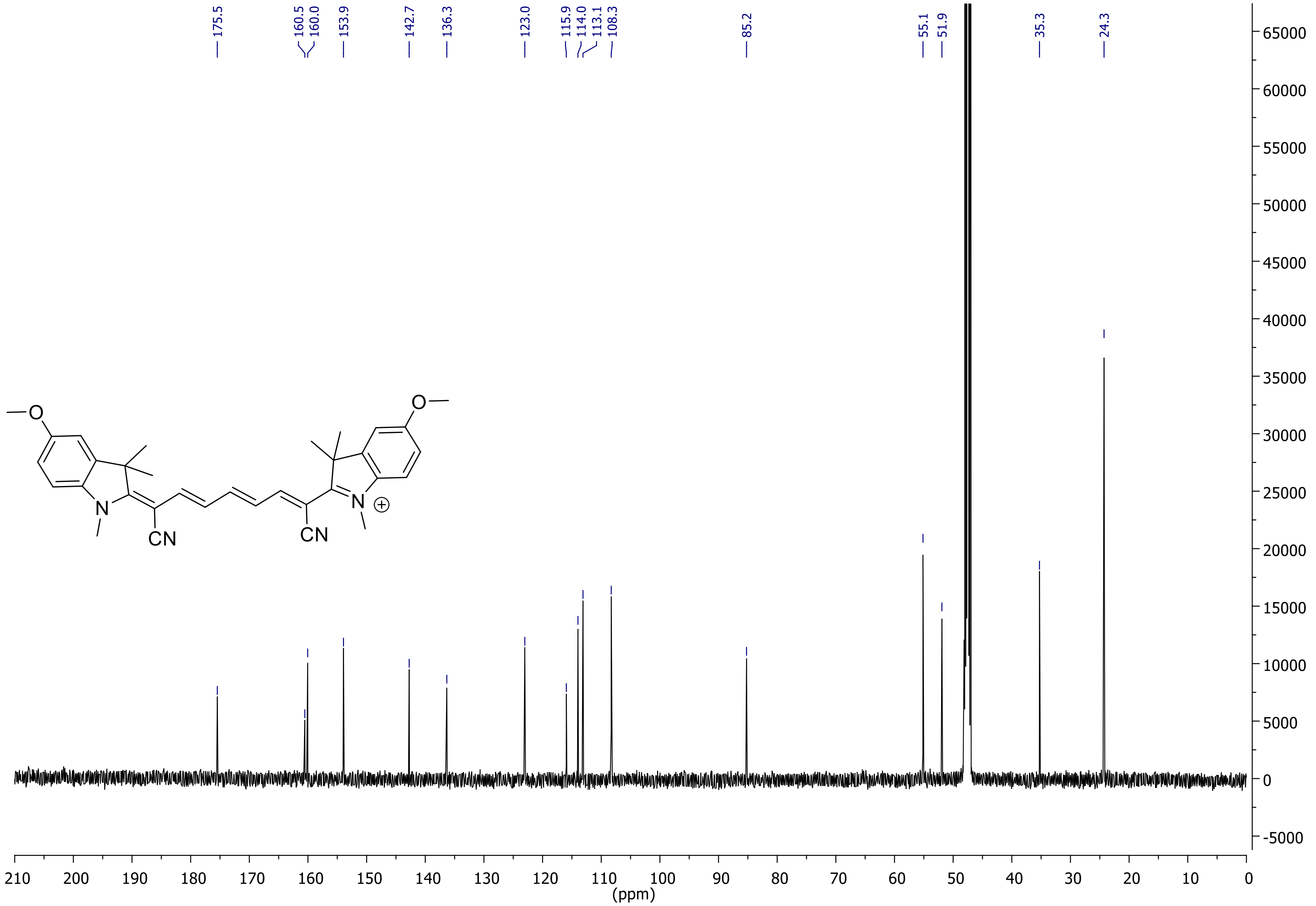

Figure S90. $^{13}C\{^{1}H\}$ NMR (126 MHz, d_4–CD_3OD): **Cy7-13**.

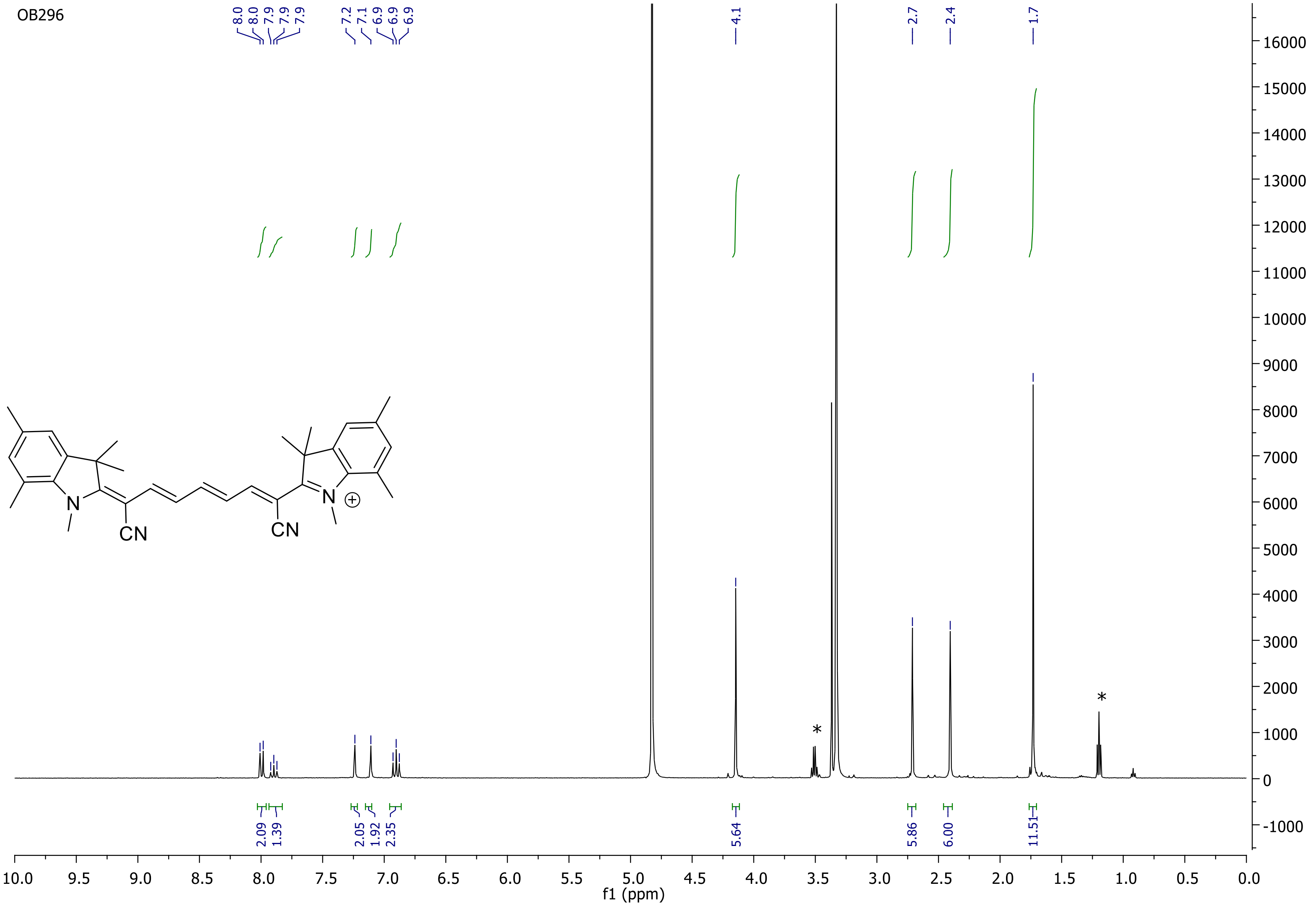

Figure S91. ^{1}H NMR (500 MHz, d_4–CD_3OD): **Cy7-14** (* diethyl ether).

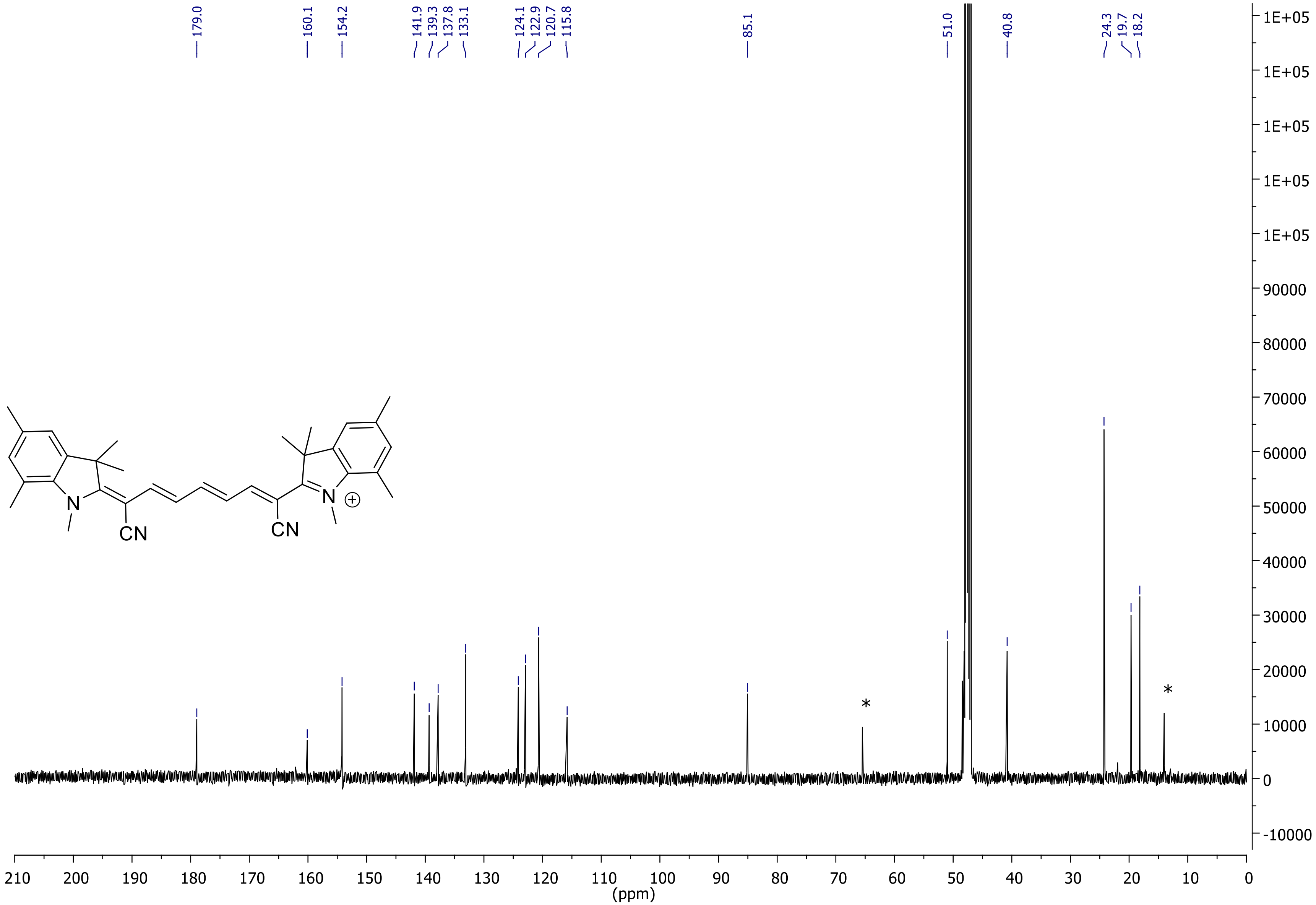

Figure S92. $^{13}C\{^{1}H\}$ NMR (126 MHz, d_4–CD_3OD): **Cy7-14** (* diethyl ether).

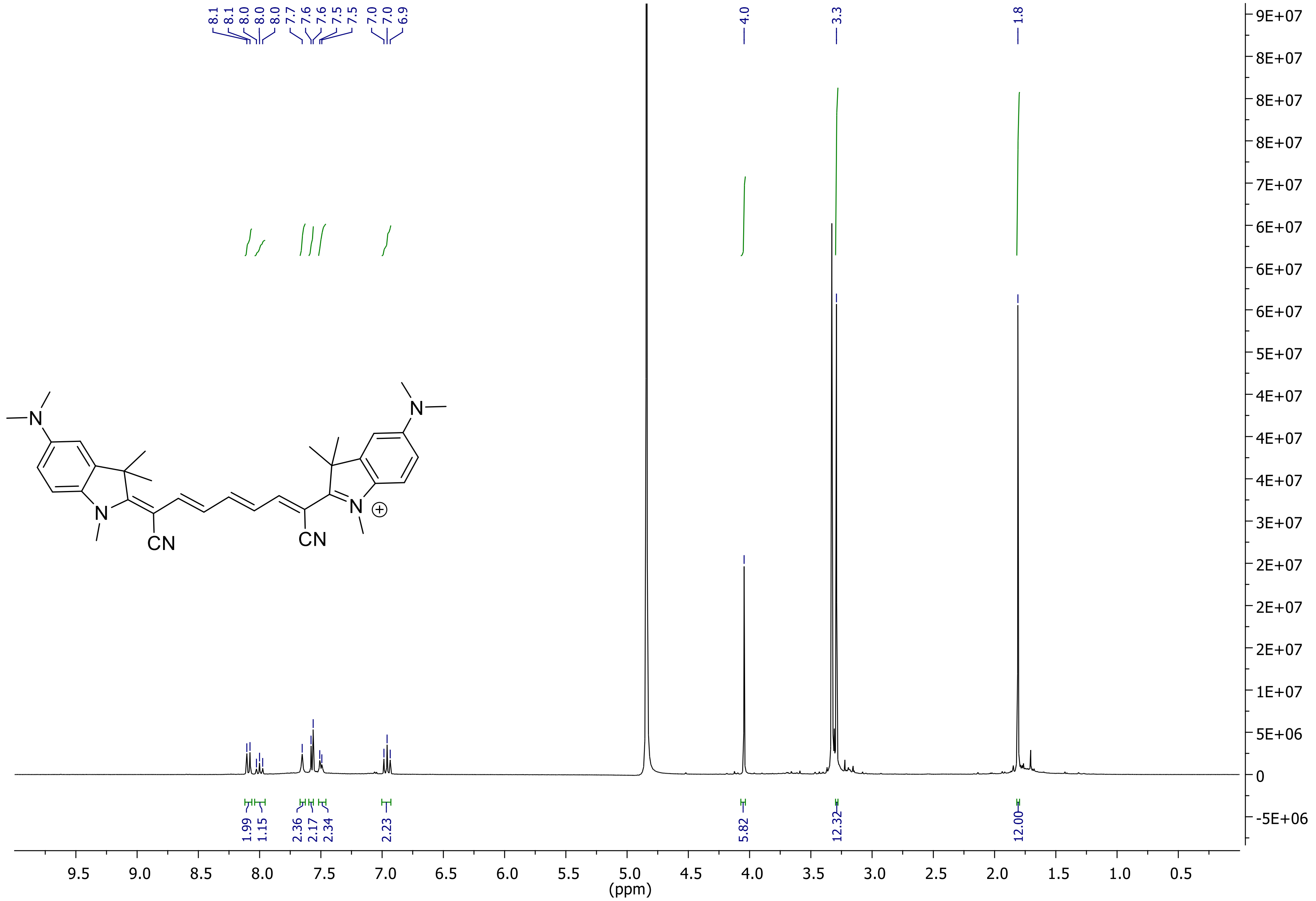

Figure S93. ^{1}H NMR (500 MHz, d_4–CD_3OD): **Cy7-15**.

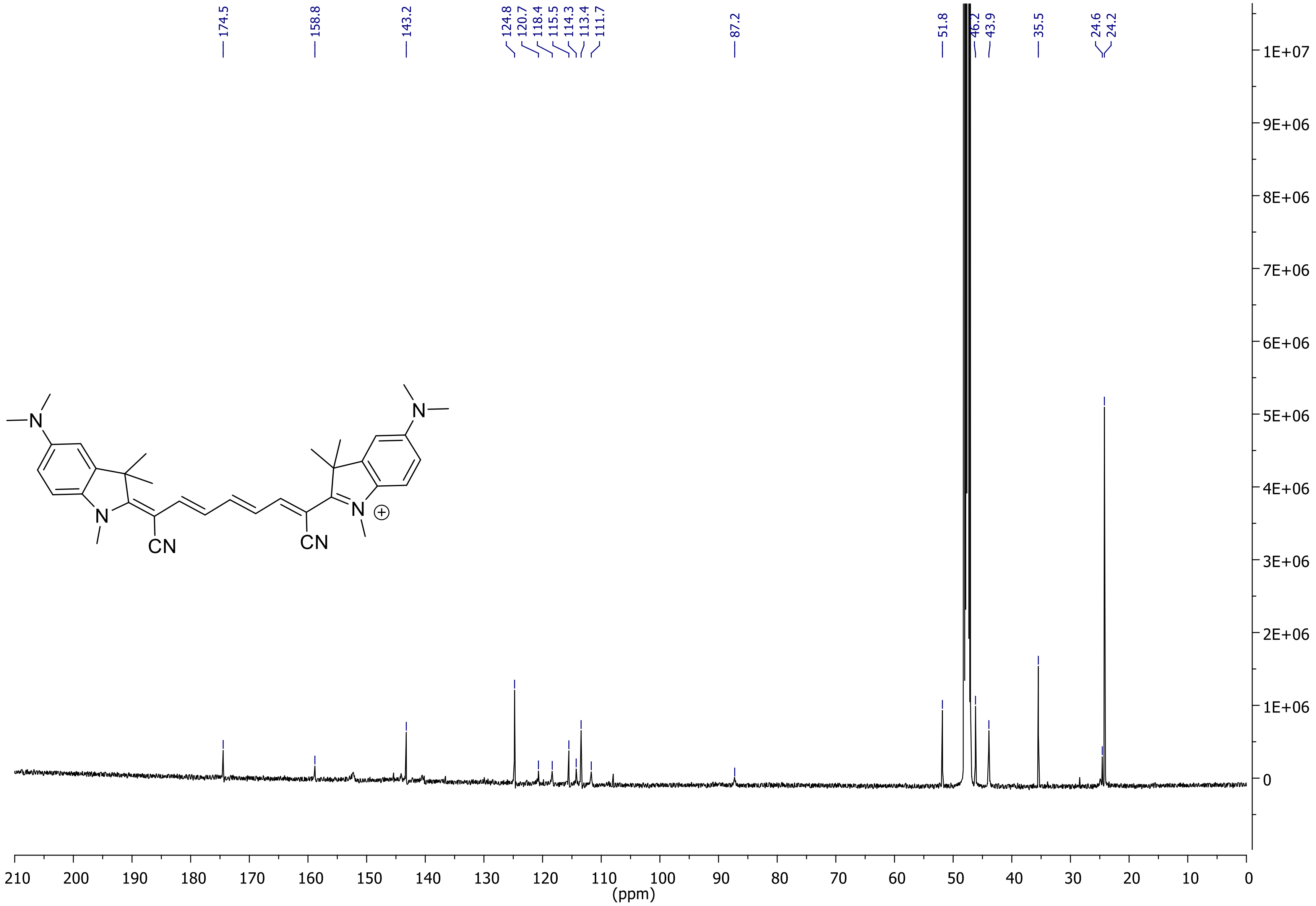

Figure S94. $^{13}C\{^1H\}$ NMR (126 MHz, d_4–CD_3OD): **Cy7-15**.

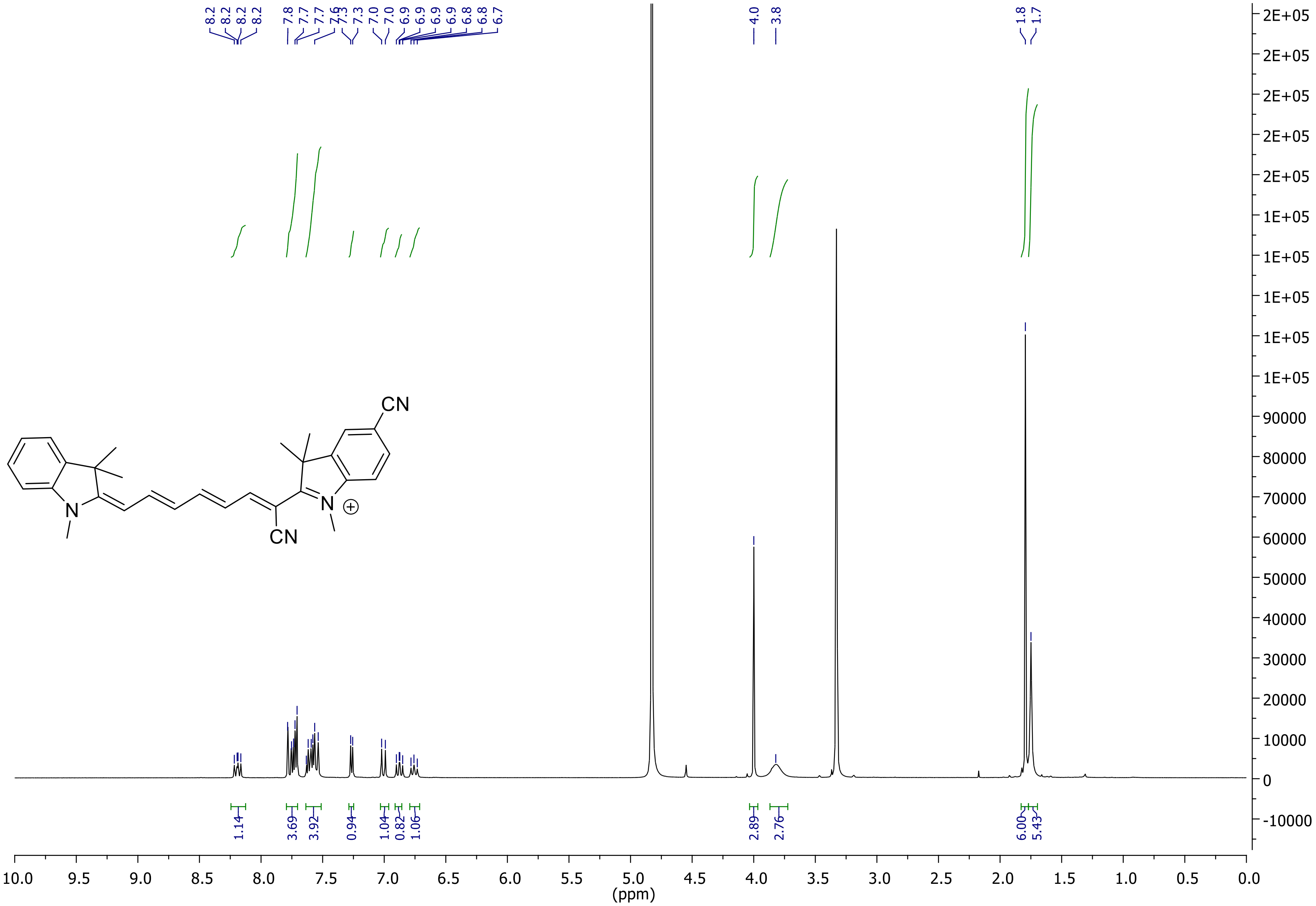

Figure S95. ^{1}H NMR (500 MHz, d_4–CD_3OD): **Cy7-17**.

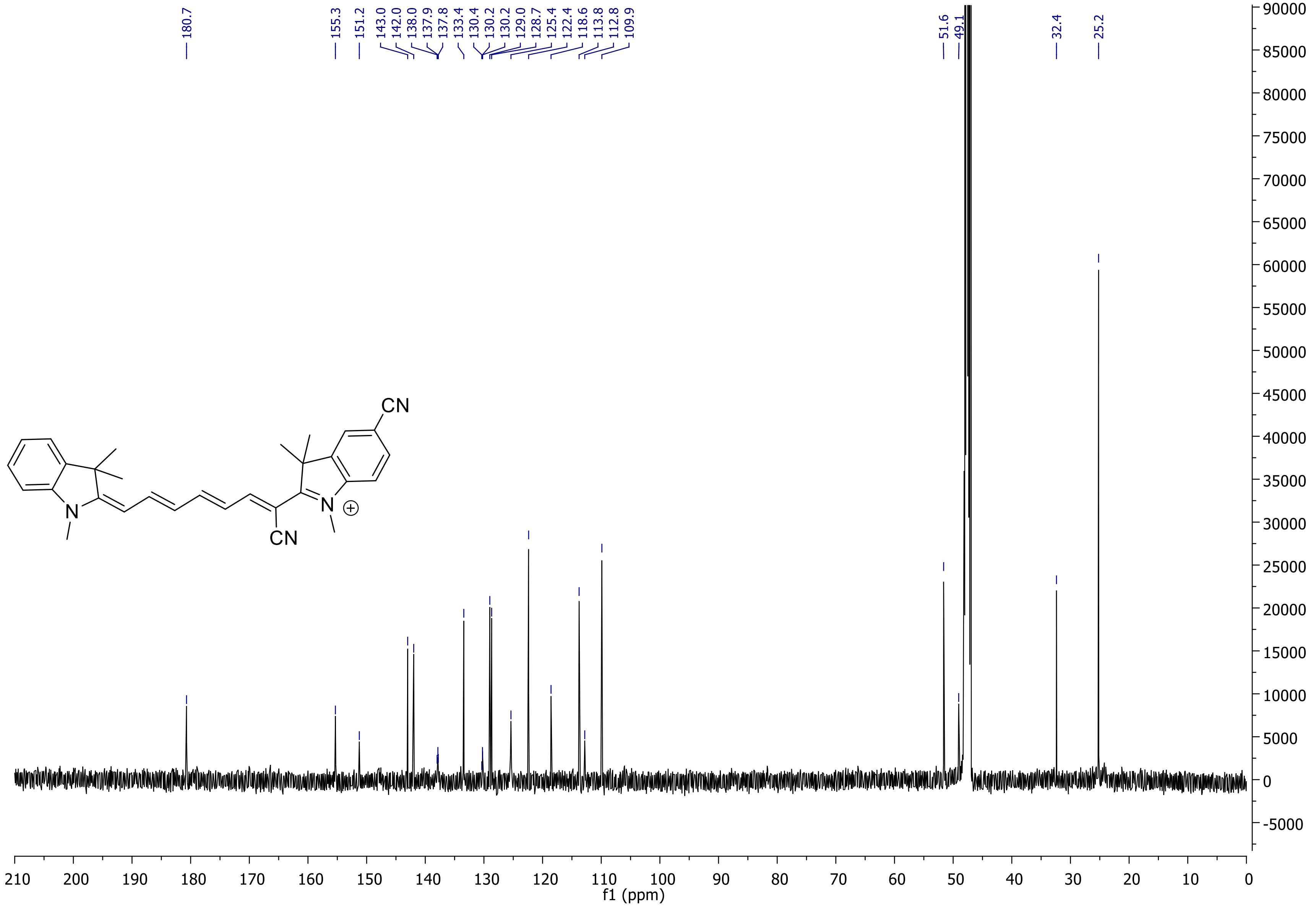

Figure S96. $^{13}C\{^{1}H\}$ NMR (126 MHz, d_4–CD_3OD): **Cy7-17**.

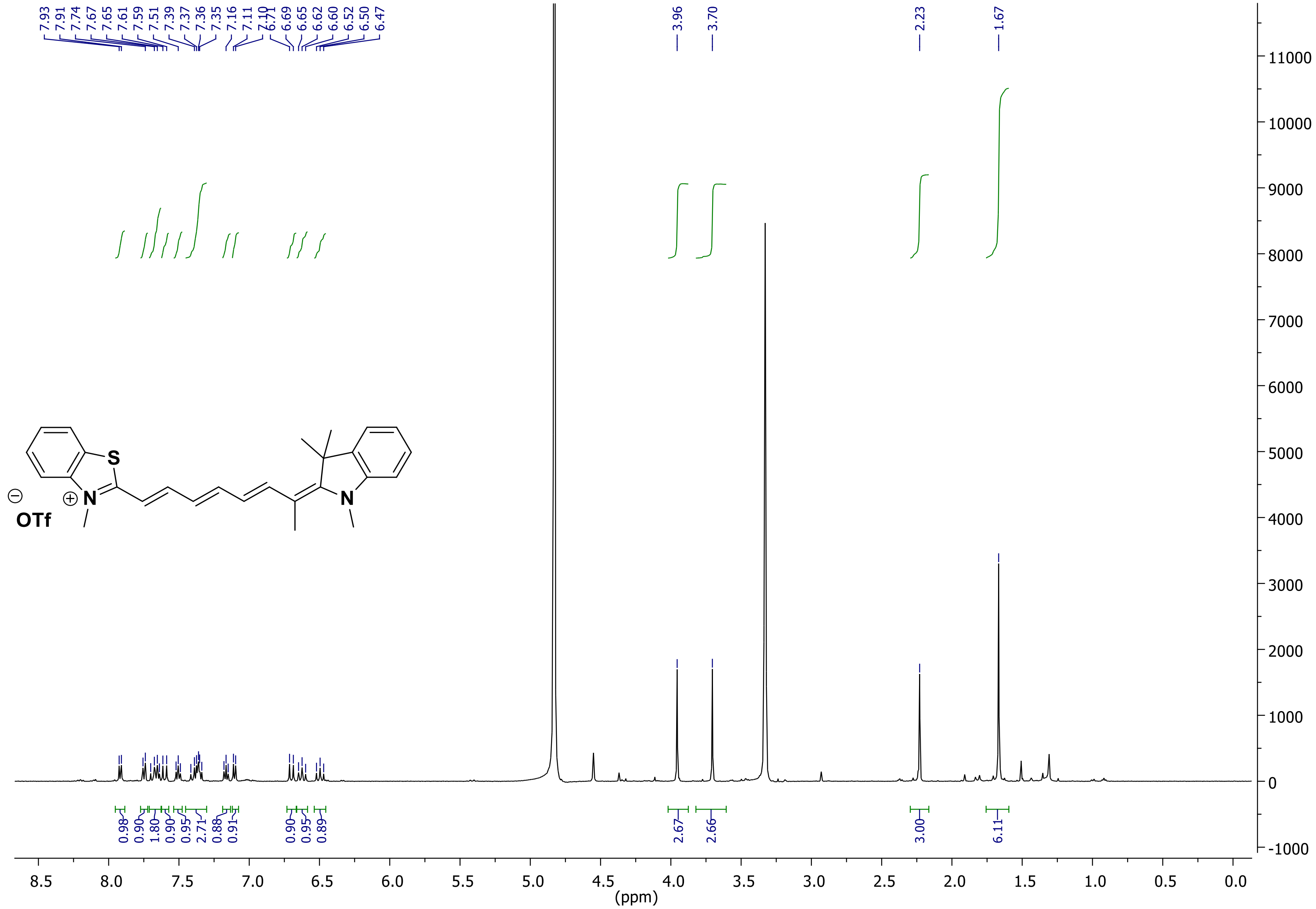

Figure S97. ^{1}H NMR (500 MHz, d_4–CD_3OD): **Cy7-18**.

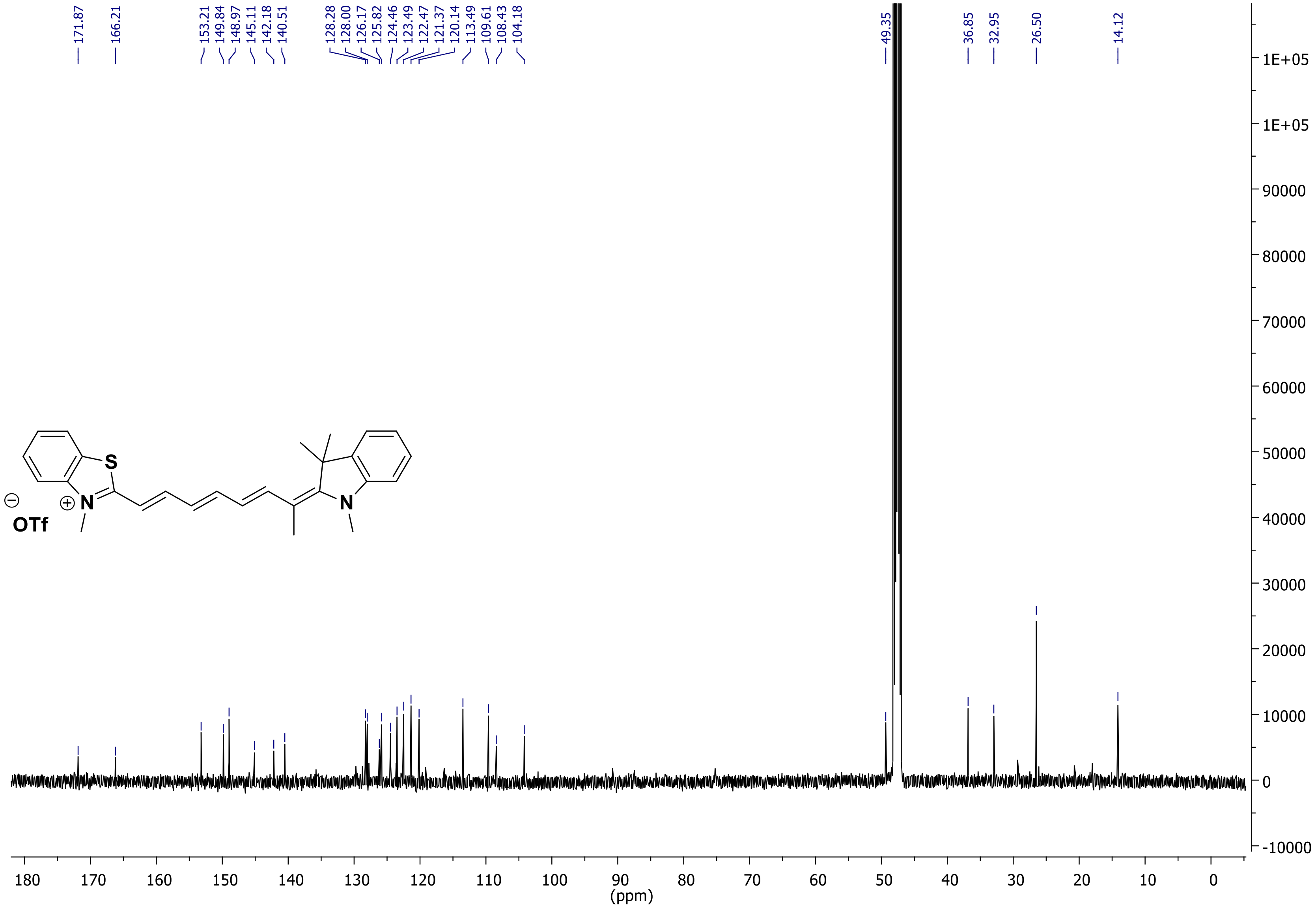

Figure S98. $^{13}C\{^{1}H\}$ NMR (126 MHz, d_4–CD_3OD): **Cy7-18**.

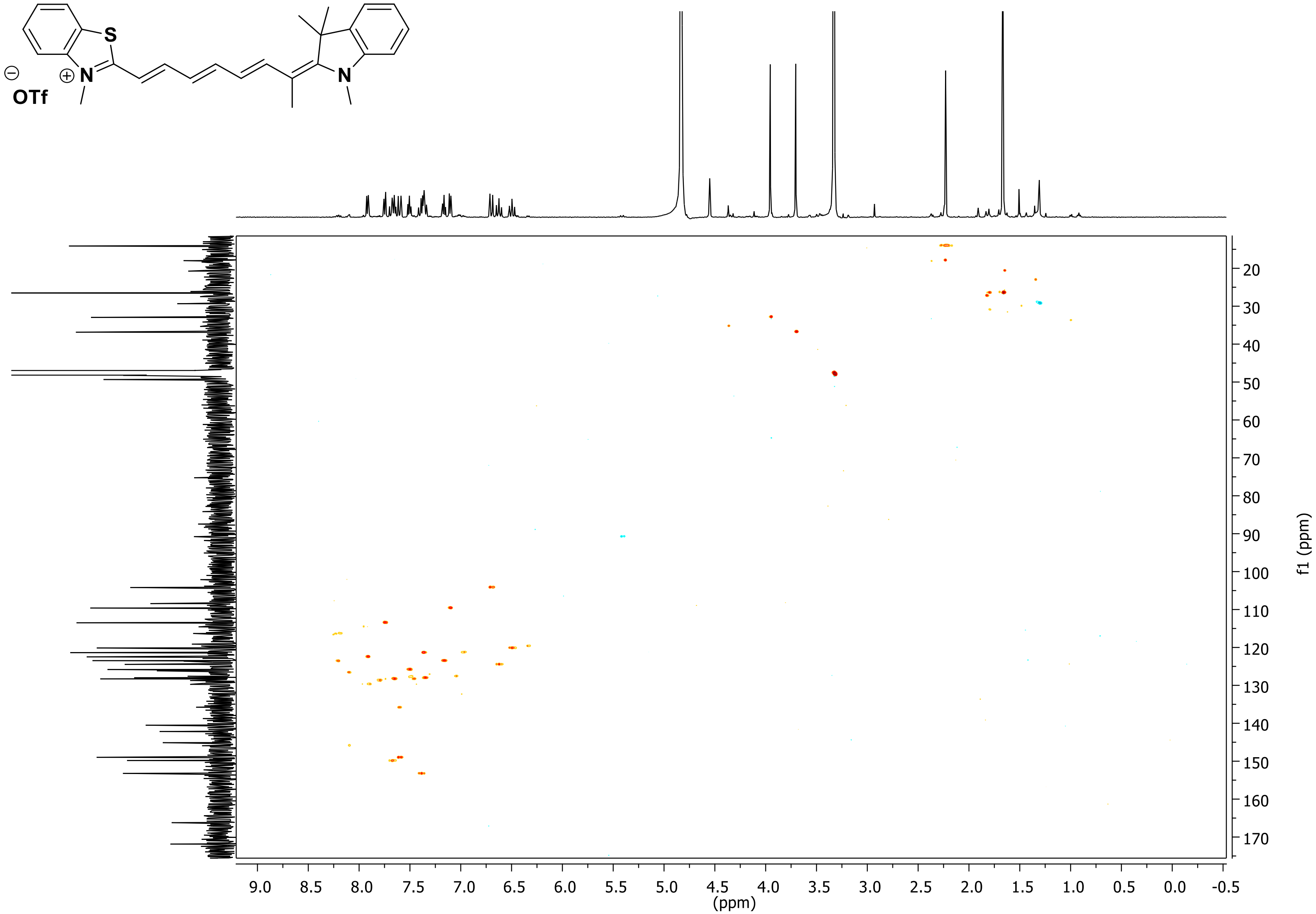

Figure S99. ^{1}H–$^{13}C\{^{1}H\}$ gHSQC (500 MHz, d_4–CD_3OD): **Cy7-18**.

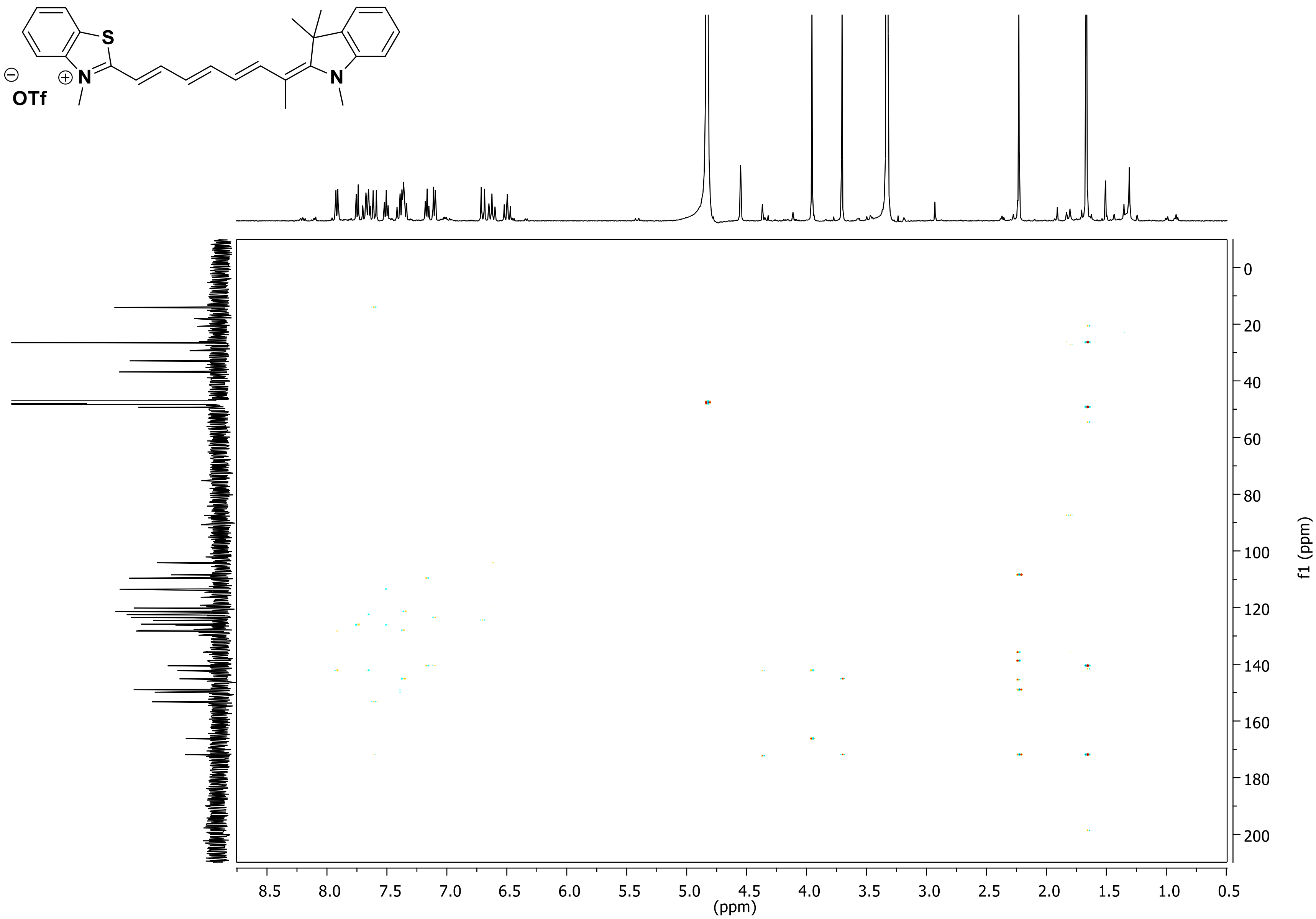

Figure S100. ^{1}H–$^{13}C\{^{1}H\}$ gHMBC (500 MHz, d_4–CD_3OD): **Cy7-18**.

MS Spectra

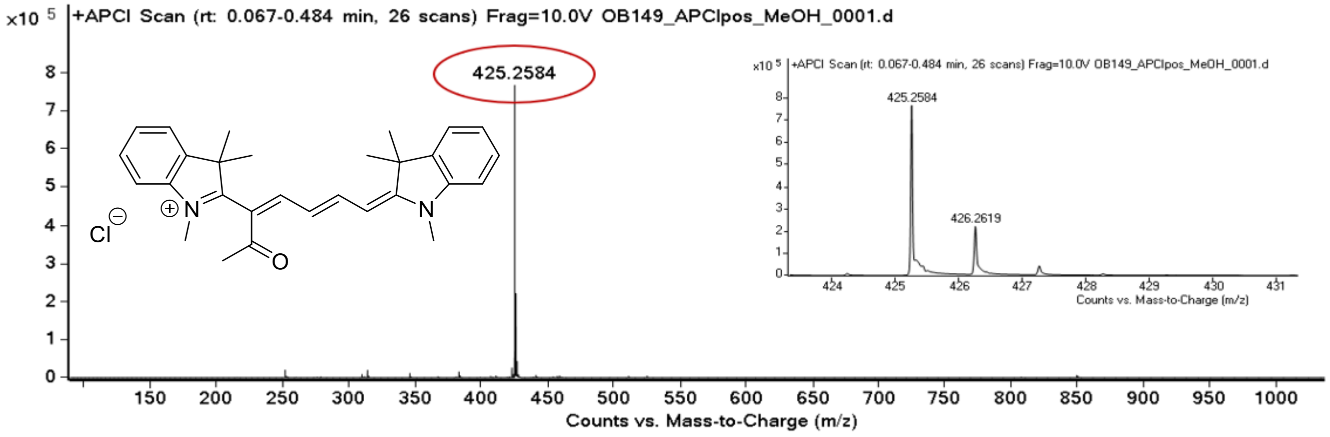

Figure S101. HRMS (ESI$^+$): **Cy5-3** (*m/z:* calcd for $C_{29}H_{33}N_2O^+$ [M – Cl $^-$]$^+$ 425.2587, found 425.2584).

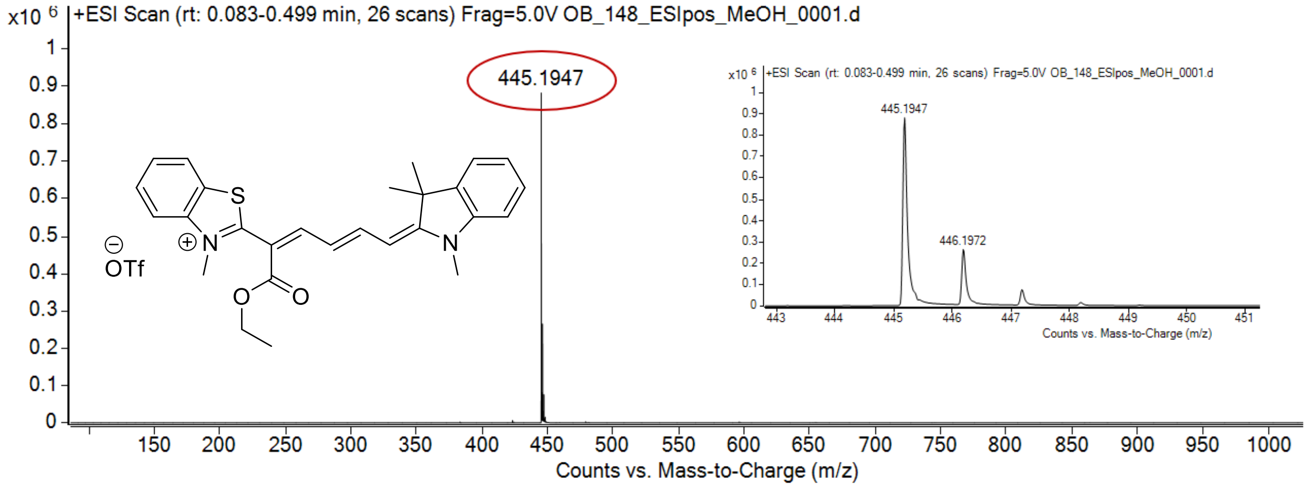

Figure S102. HRMS (ESI$^+$): **Cy5-5** (*m/z:* calcd for $C_{27}H_{29}N_2O_2S^+$ [M – OTf $^-$]$^+$ 445.1944, found 445.1947).

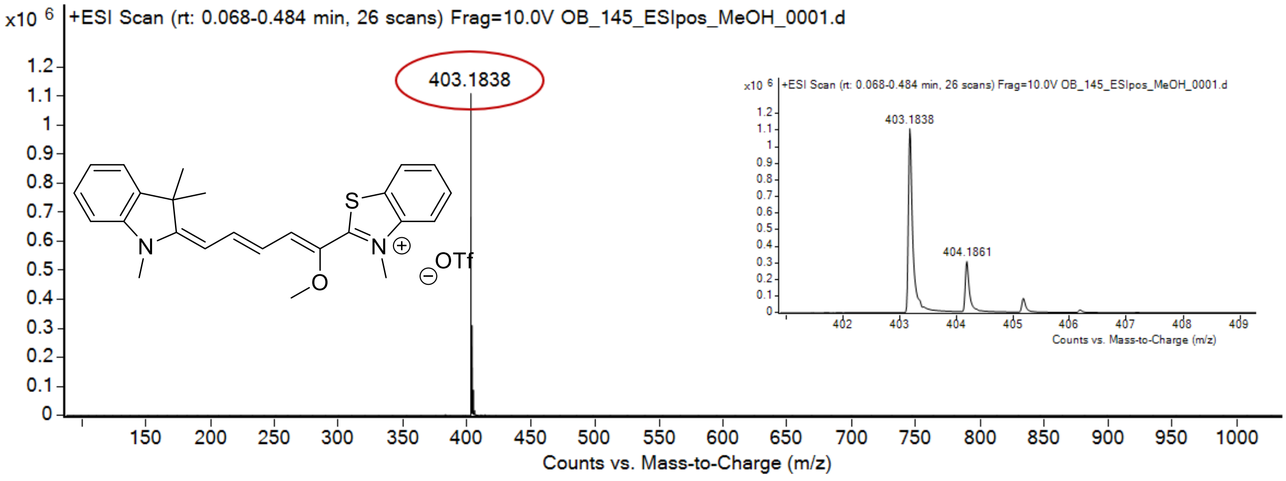

Figure S103. HRMS (ESI$^+$): **Cy5-7** (*m/z:* calcd for $C_{23}H_{27}N_2OS^+$ [M – OTf $^-$]$^+$ 403.1839, found 403.1838).

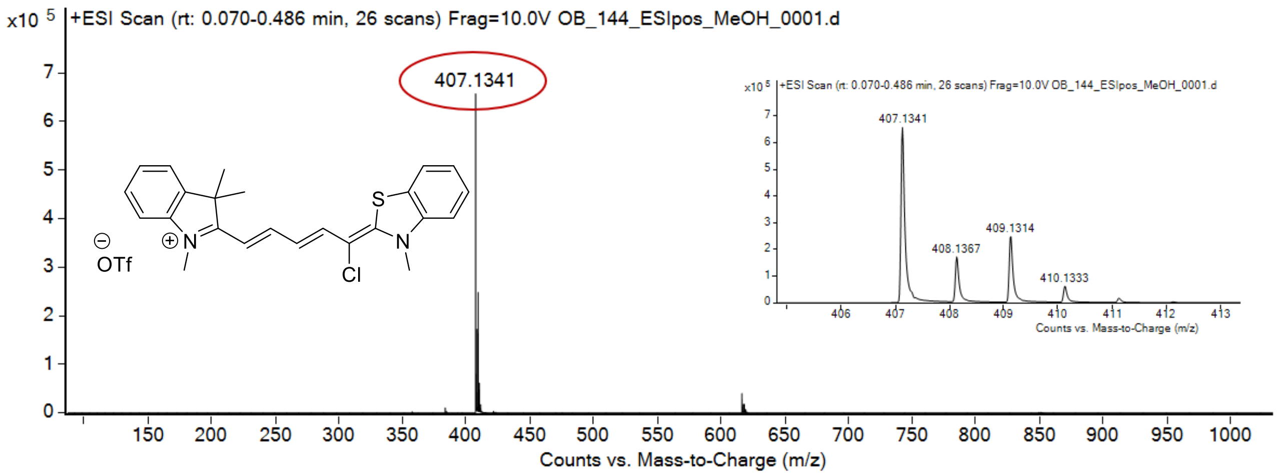

Figure S104. HRMS (ESI^+): **Cy5-8** (*m/z:* calcd for $C_{24}H_{24}ClN_2S^+$ $[M - OTf^-]^+$ 407.1343, found 407.1341).

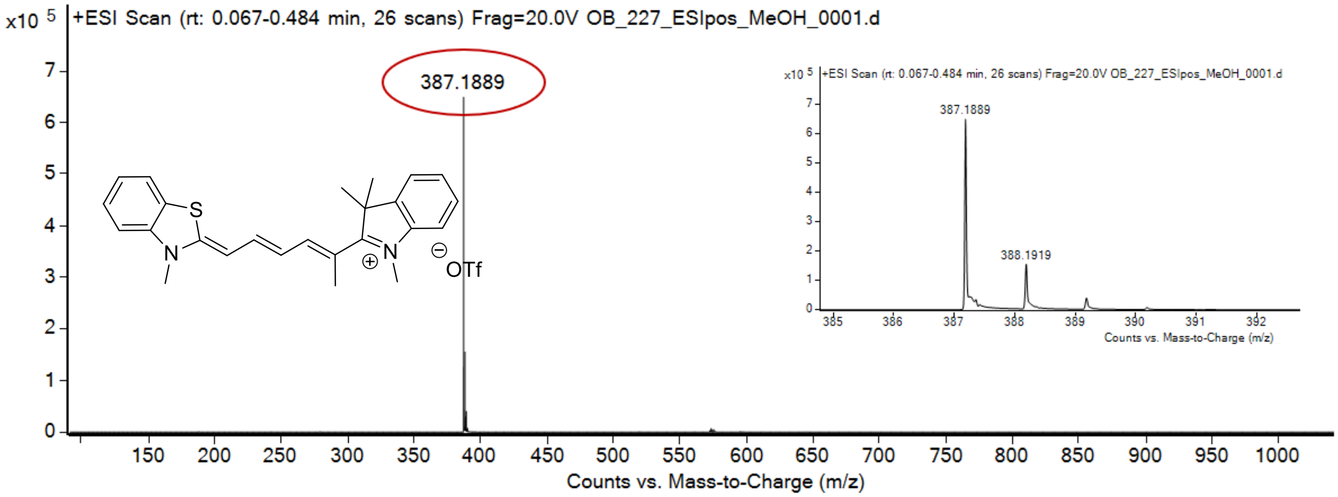

Figure S105. HRMS (ESI^+): **Cy5-10** (*m/z:* calcd for $C_{25}H_{27}N_2S^+$ $[M - OTf^-]^+$ 387.1889, found 387.1889).

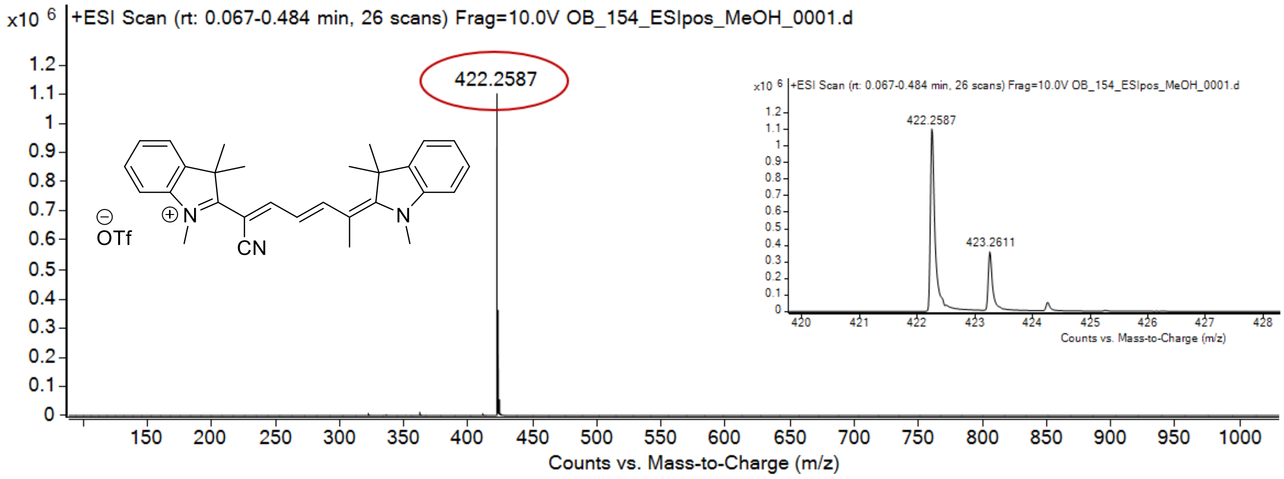

Figure S106. HRMS (ESI^+): **Cy5-11** (*m/z:* calcd for $C_{29}H_{32}N_3^+$ $[M - OTf^-]^+$ 422.2591, found 422.2587).

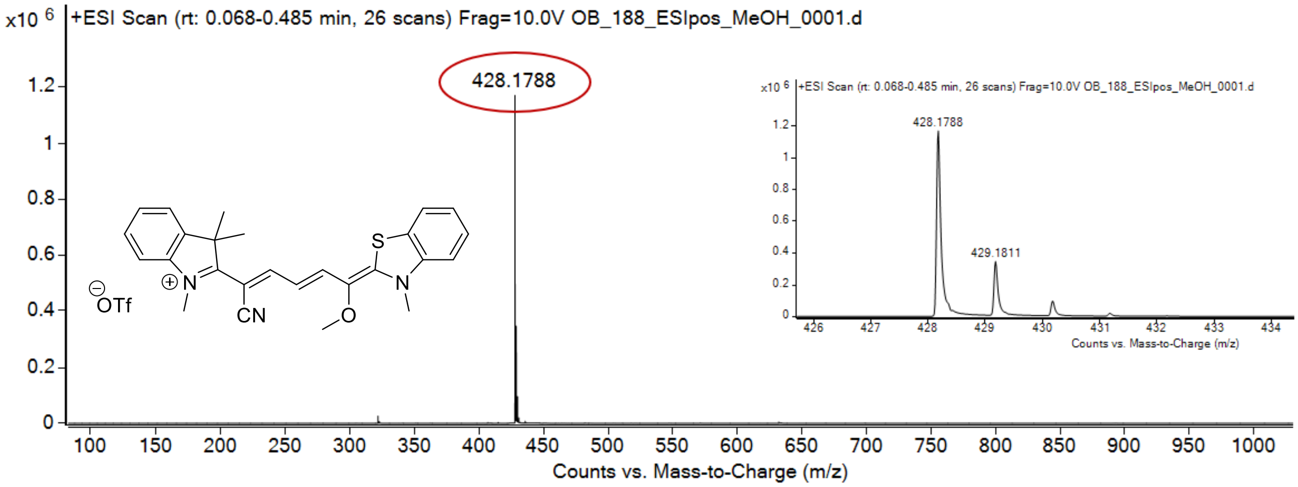

Figure S107. HRMS (ESI$^+$): **Cy5-12** (*m/z:* calcd for $C_{26}H_{26}N_3OS^+$ [M – OTf $^-$]$^+$ 428.1791, found 428.1788).

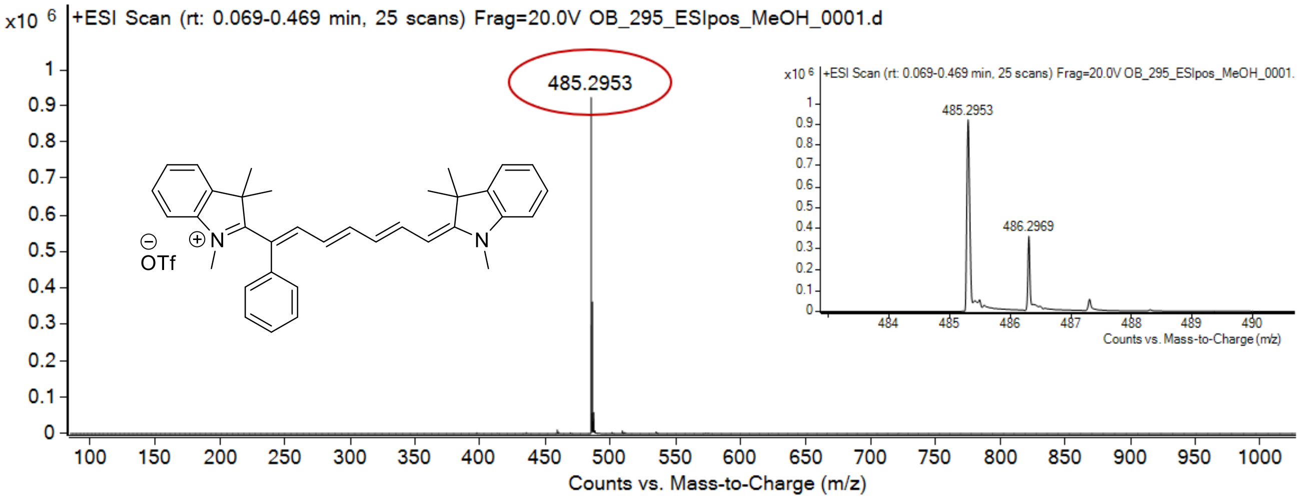

Figure S108. HRMS (ESI$^+$): **Cy7-2** (*m/z:* calcd for $C_{35}H_{37}N_2^+$ [M – OTf $^-$]$^+$ 485.2951, found 485.2953).

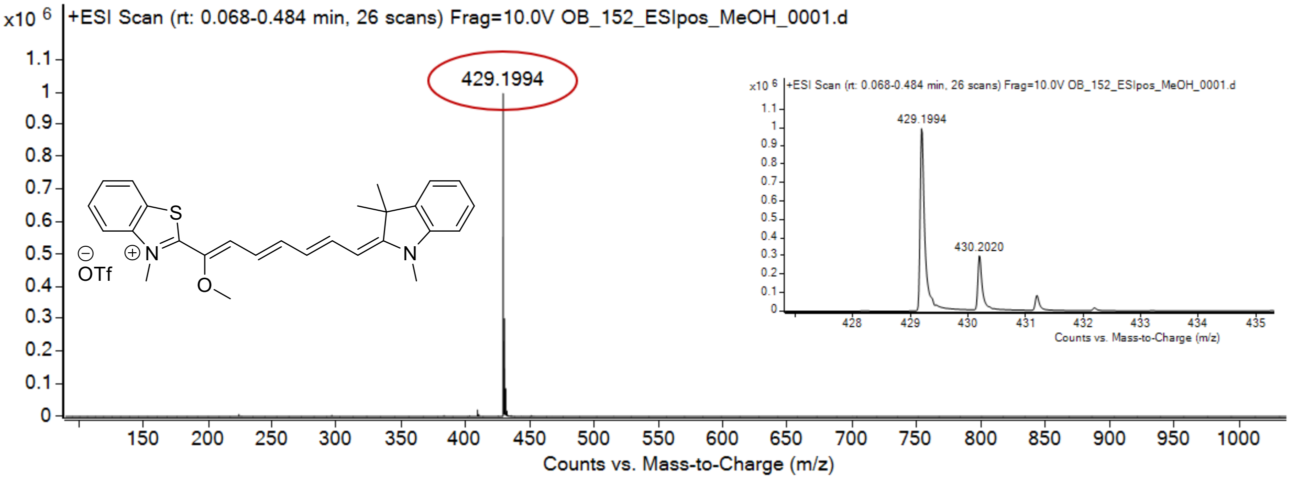

Figure S109. HRMS (ESI$^+$): **Cy7-3** (*m/z:* calcd for $C_{27}H_{29}N_2OS^+$ [M – OTf $^-$]$^+$ 429.1995, found 429.1994).

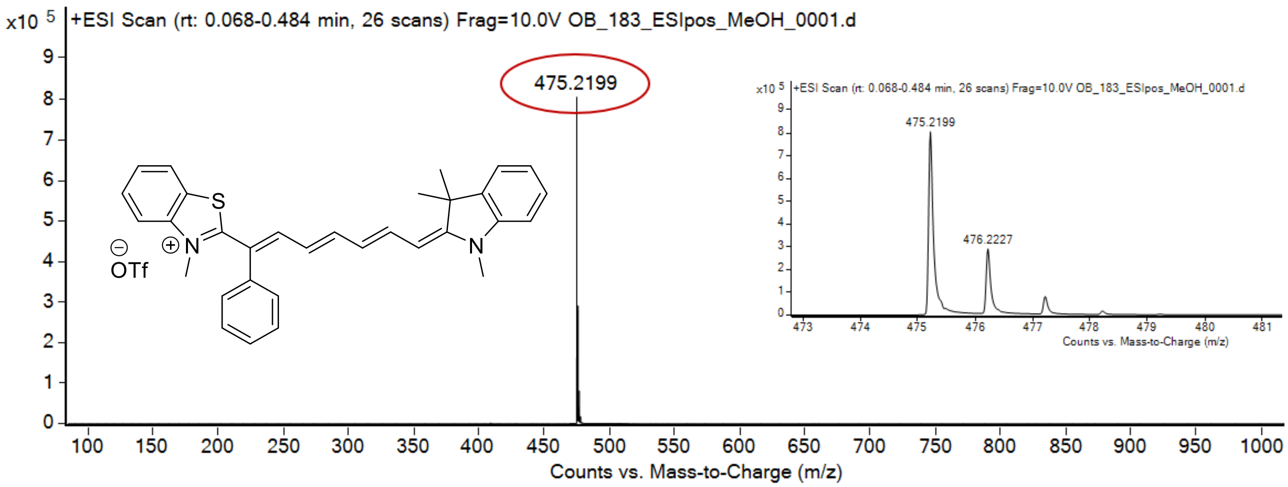

Figure S110. HRMS (ESI$^+$): **Cy7-5** (*m/z:* calcd for $C_{32}H_{31}N_2S^+$ [M – OTf $^-$]$^+$ 475.2202, found 475.2199).

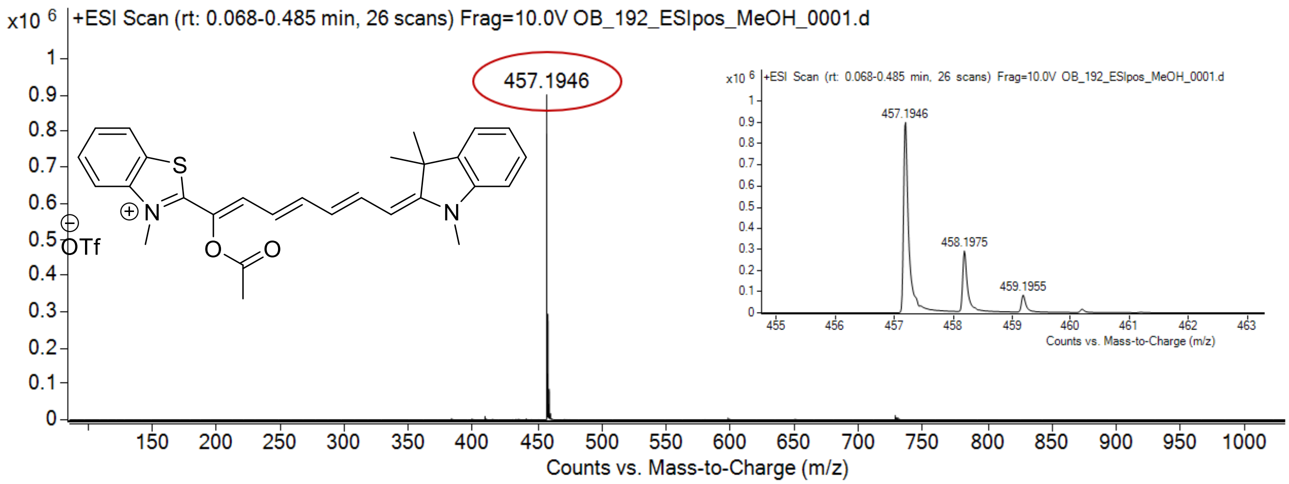

Figure S111. HRMS (ESI$^+$): **Cy7-6** (*m/z:* calcd for $C_{28}H_{29}N_2O_2S^+$ [M – OTf $^-$]$^+$ 457.1944, found 457.1946).

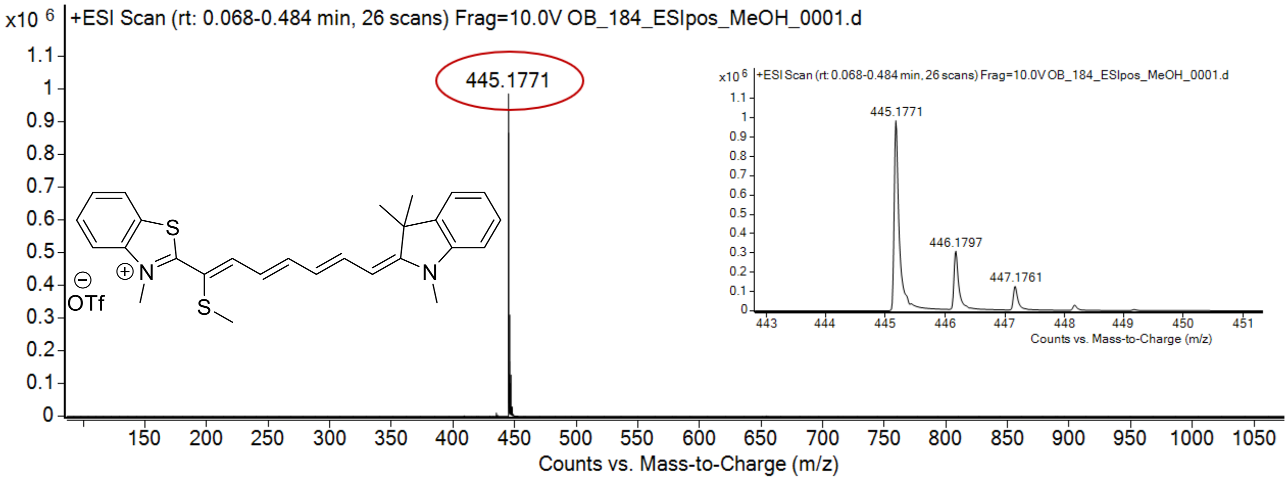

Figure S112. HRMS (ESI$^+$): **Cy7-7** (*m/z:* calcd for $C_{27}H_{29}N_2S_2^+$ [M – OTf $^-$]$^+$ 445.1767, found 445.1771).

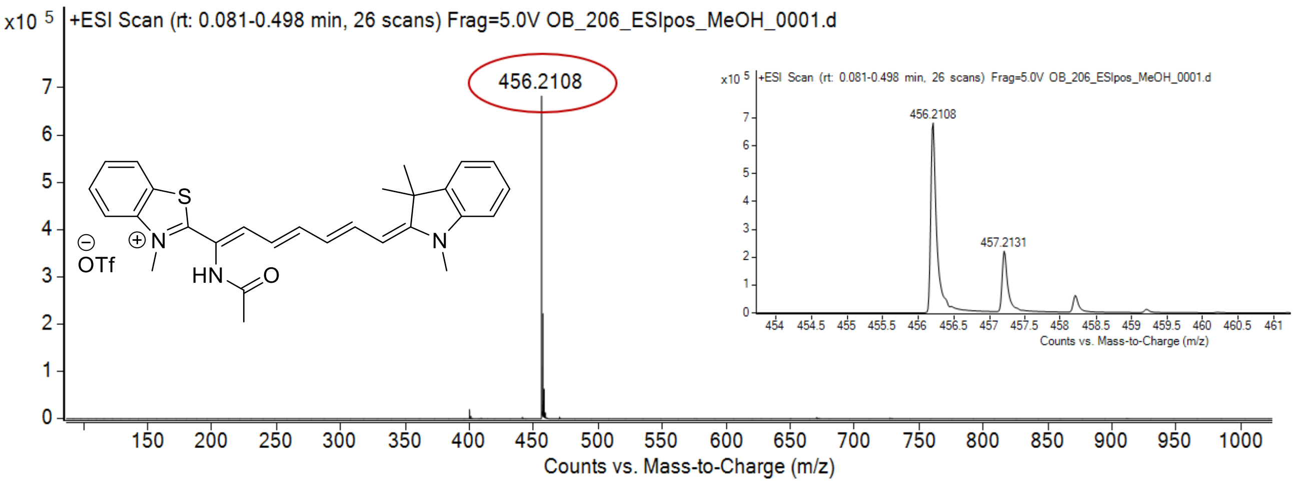

Figure S113. HRMS (ESI$^+$): **Cy7-8** (*m/z:* calcd for $C_{28}H_{30}N_3OS^+$ [M – OTf $^-$]$^+$ 456.2104, found 456.2108).

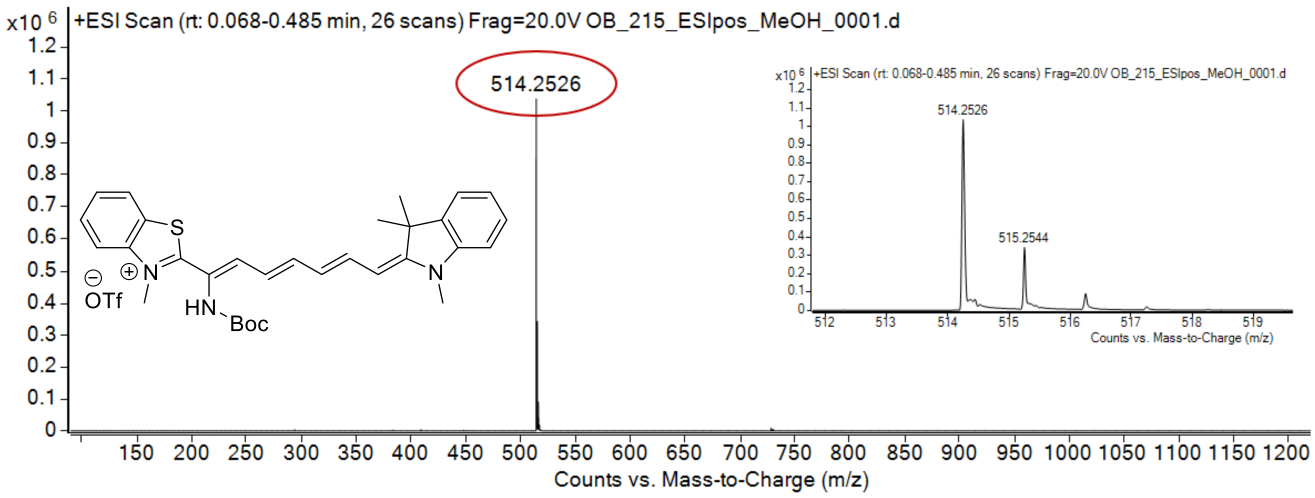

Figure S114. HRMS (ESI$^+$): **Cy7-10** (*m/z:* calcd for $C_{31}H_{36}N_3O_2S^+$ [M – OTf $^-$]$^+$ 514.2523, found 514.2526).

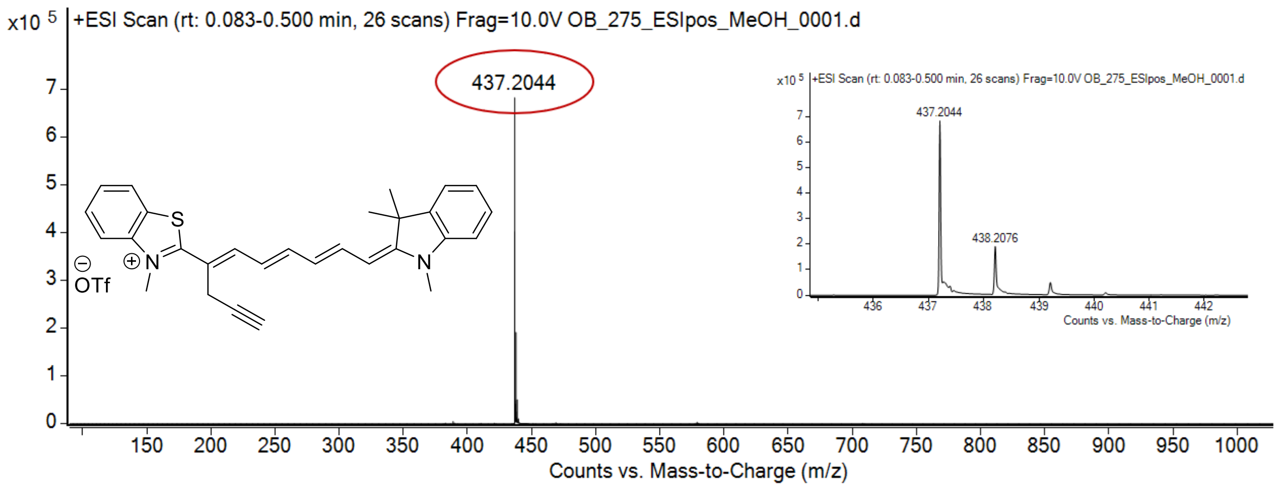

Figure S115. HRMS (ESI$^+$): **Cy7-11** (*m/z:* calcd for $C_{29}H_{29}N_2S^+$ [M – OTf $^-$]$^+$ 437.2046, found 437.2044).

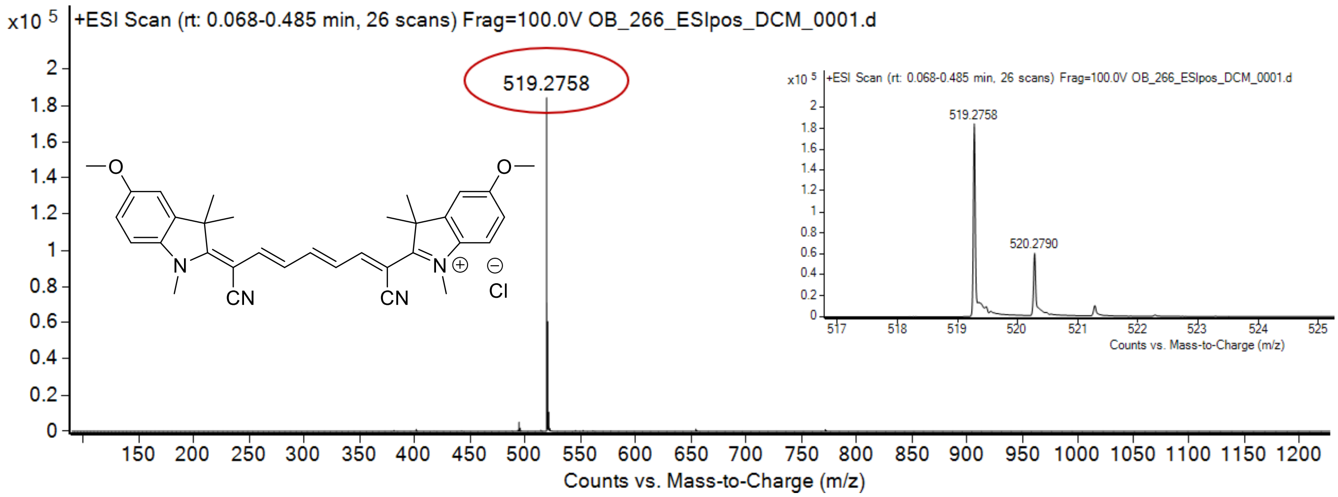

Figure S116. HRMS (ESI$^+$): **Cy7-13** (*m/z:* calcd for $C_{33}H_{35}N_4O_2^+$ $[M - Cl^-]^+$ 519.2755, found 519.2758).

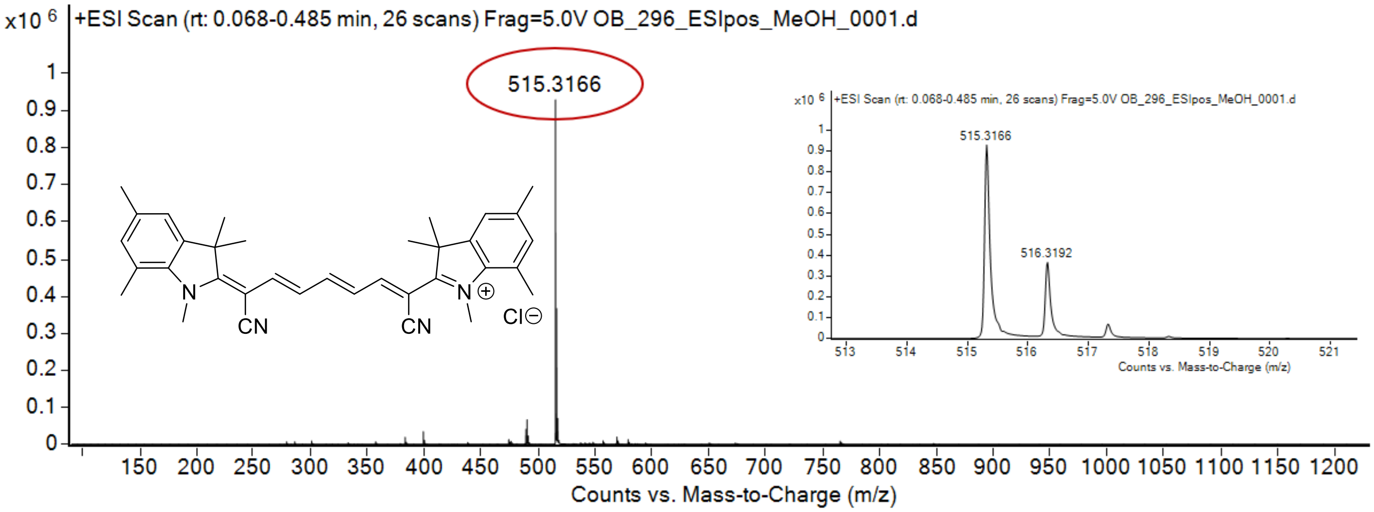

Figure S117. HRMS (ESI$^+$): **Cy7-14** (*m/z:* calcd for $C_{35}H_{39}N_4^+$ $[M - Cl^-]^+$ 515.3169, found 515.3166).

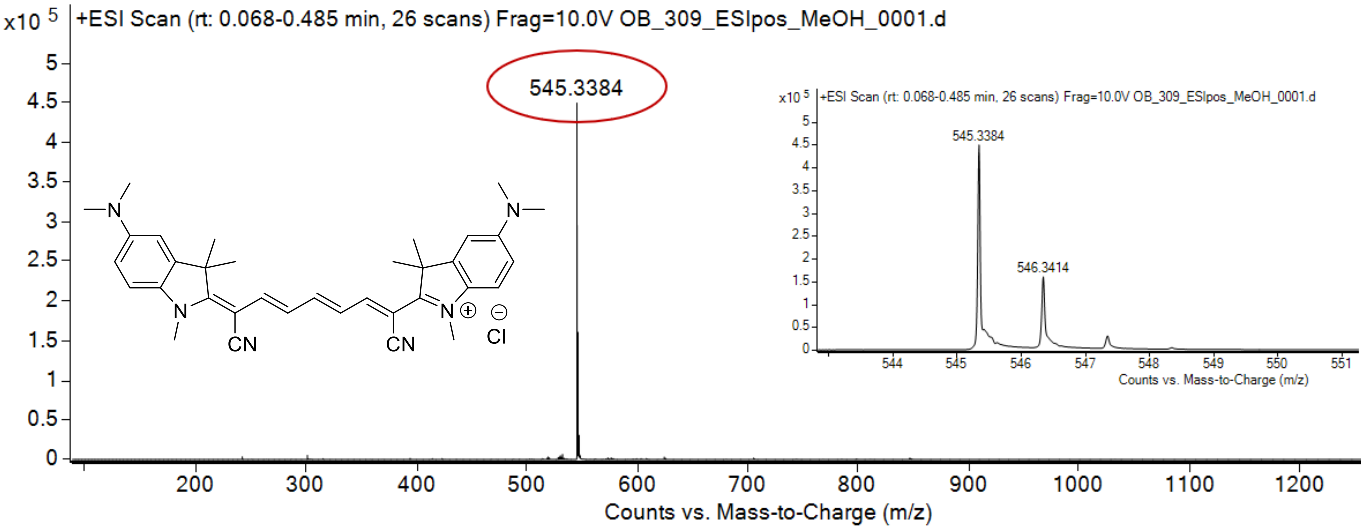

Figure S118. HRMS (ESI$^+$): **Cy7-15** (*m/z:* calcd for $C_{35}H_{41}N_6^+$ $[M - Cl^-]^+$ 545.3387, found 545.3384).

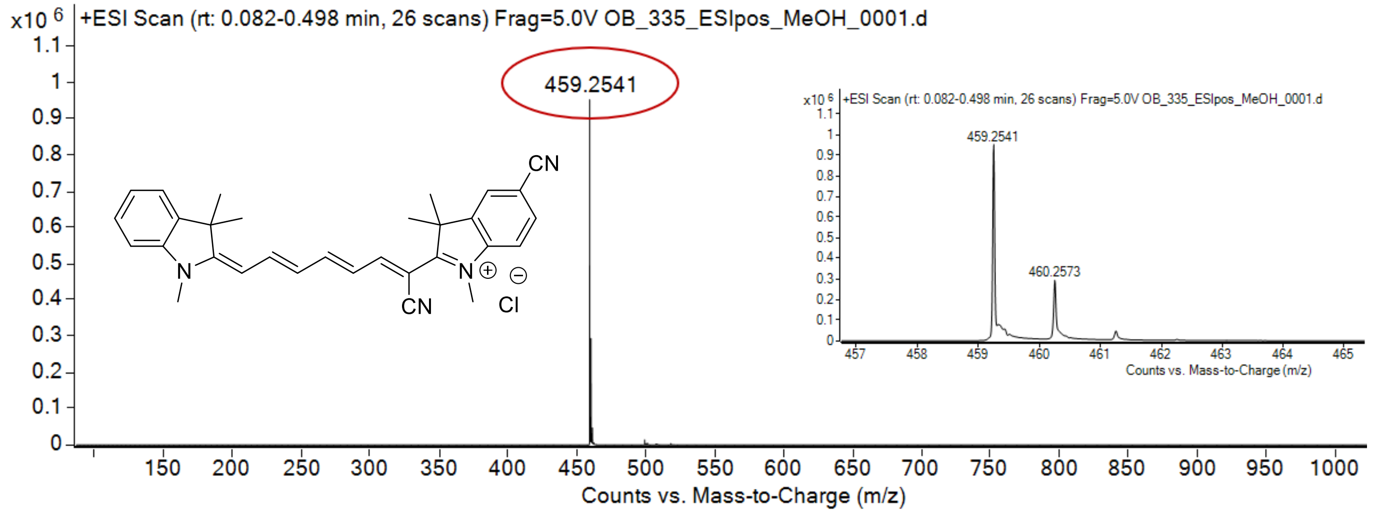

Figure S119. HRMS (ESI$^+$): **Cy7-17** (*m/z:* calcd for $C_{31}H_{31}N_4^+$ [M – Cl $^-$]$^+$ 459.2543, found 459.2541).

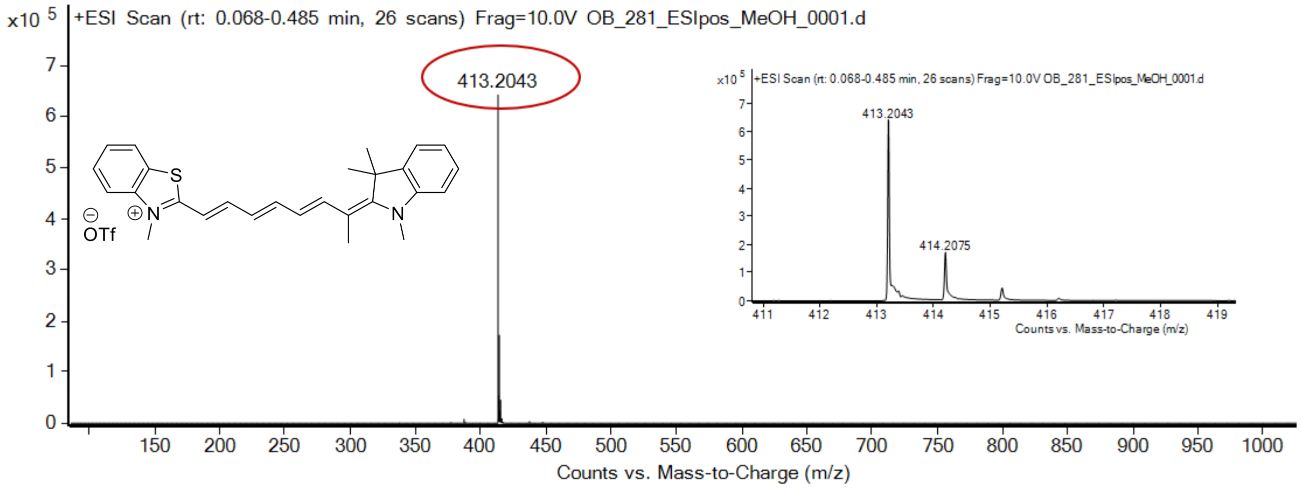

Figure S120. HRMS (ESI$^+$): **Cy-7 18** (*m/z:* calcd for $C_{27}H_{29}N_2S^+$ [M – OTf $^-$]$^+$ 413.2046, found 413.2043).

Absorption and Emission Spectra

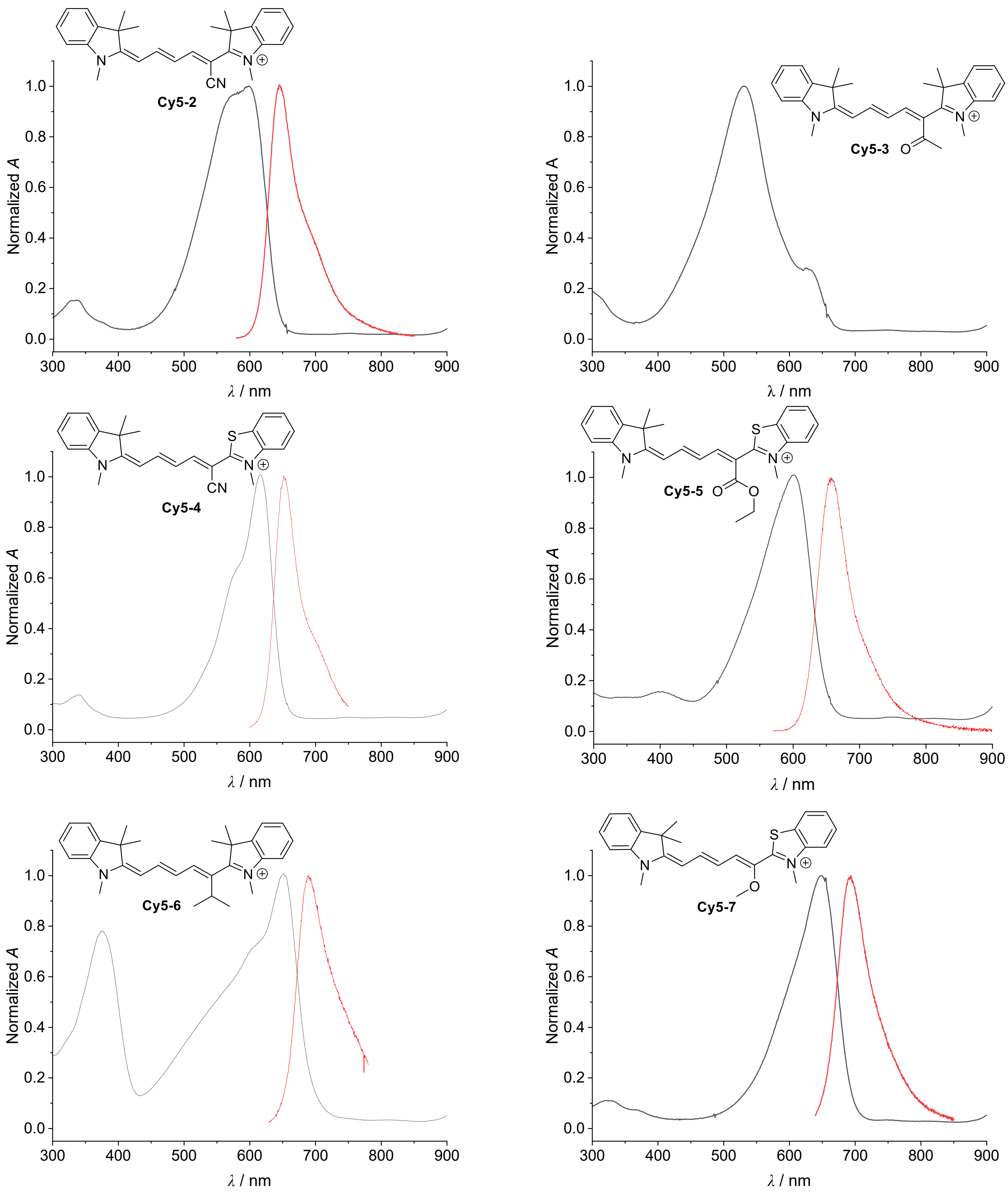

Figure S121. Absorption (black) and emission (red) spectra of **Cy5-2**, **Cy5-3**, **Cy5-4**, **Cy5-5**, **Cy5-6**, **Cy5-7** obtained in methanol.

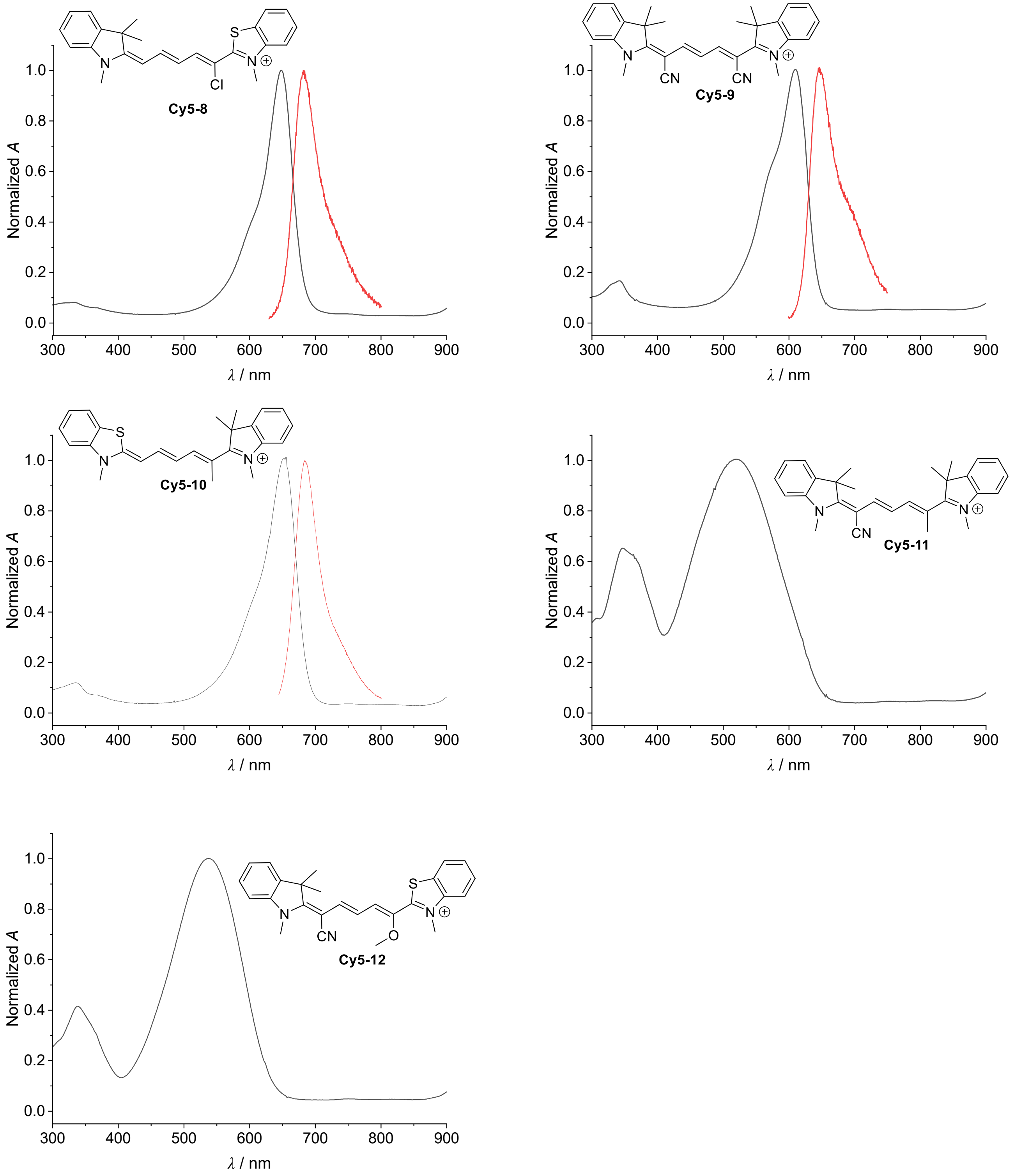

Figure S122. Absorption (black) and emission (red) spectra of **Cy5-8**, **Cy5-9**, **Cy5-10**, **Cy5-11**, **Cy5-12** obtained in methanol.

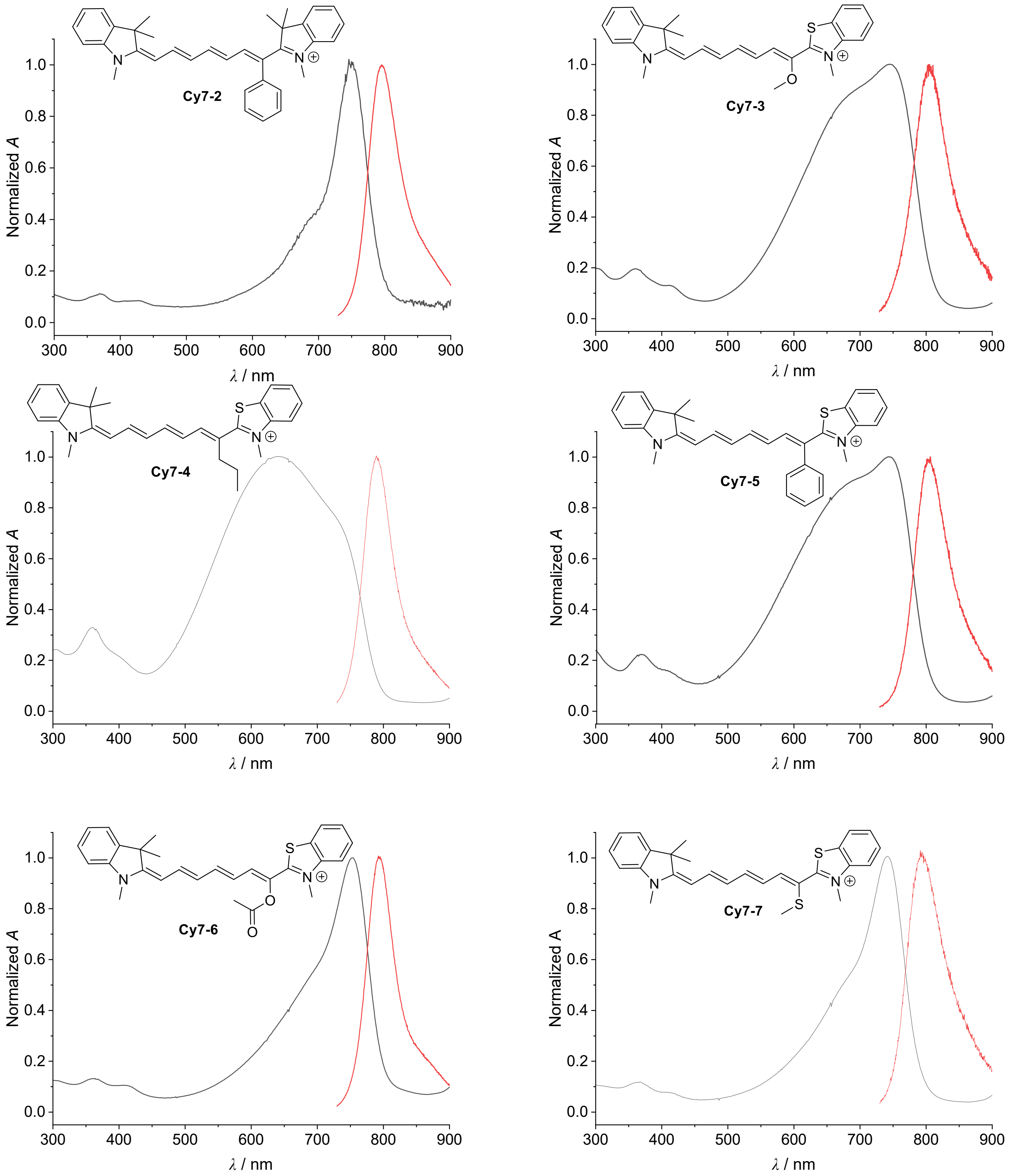

Figure S123. Absorption (black) and emission (red) spectra of **Cy7-2**, **Cy7-3**, **Cy7-4**, **Cy7-5**, **Cy7-6, Cy7-7** obtained in methanol.

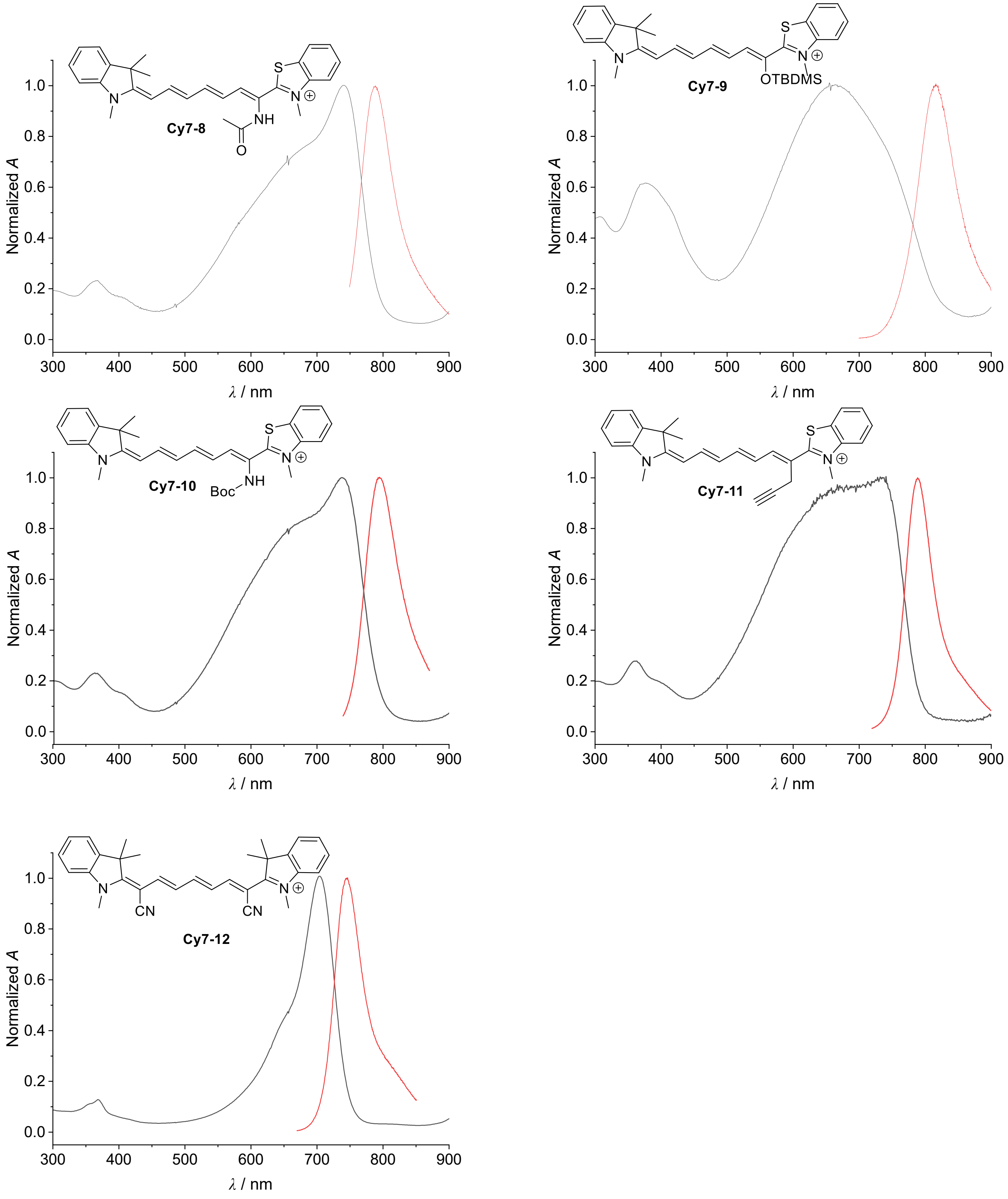

Figure S124. Absorption (black) and emission (red) spectra of **Cy7-8, Cy7-9**, **Cy7-10**, **Cy7-11**, **Cy7-12**, obtained in methanol.

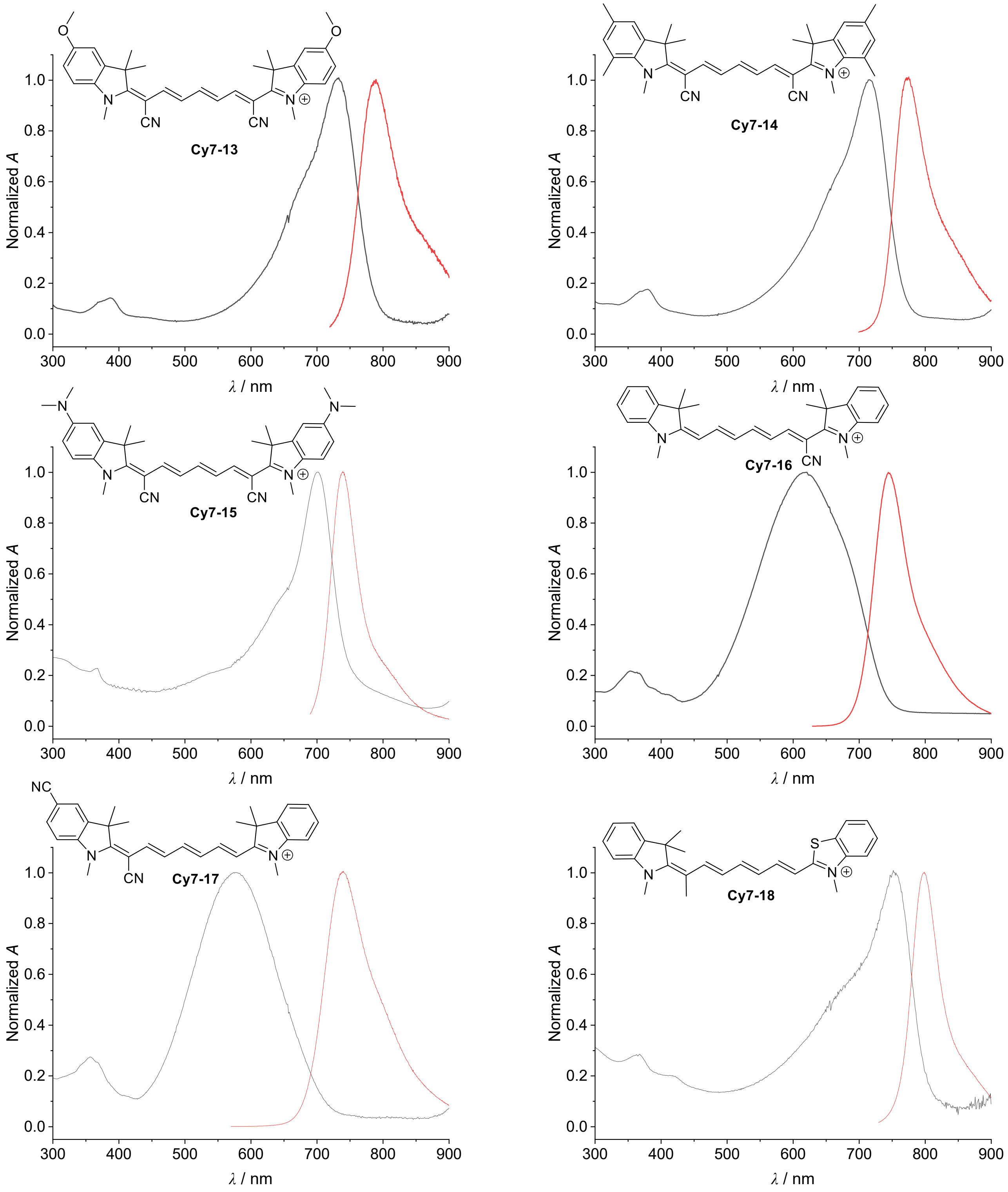

Figure S125. Absorption (black) and emission (red) spectra of **Cy7-13**, **Cy7-14**, **Cy7-15**, **Cy7-16**, **Cy7-17**, **Cy7-18** obtained in methanol.

Spectroscopic Determination of p*K*a

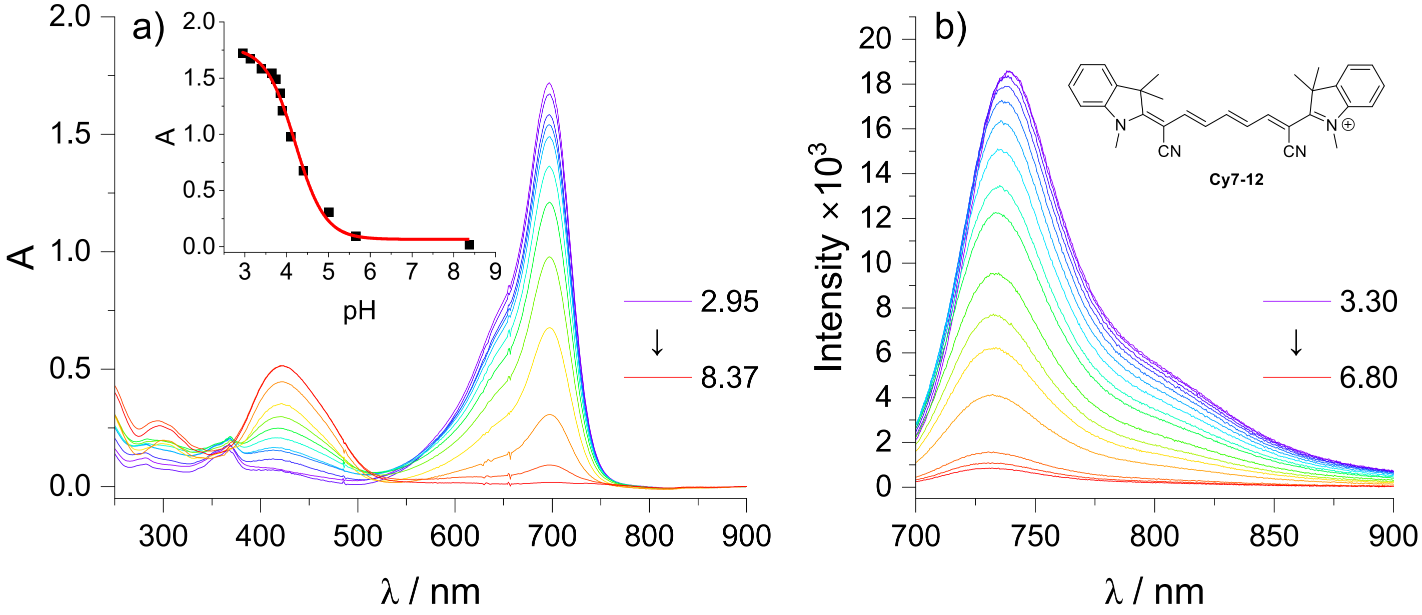

Figure S126. a) UV-vis spectra of **Cy-12** at different pH. b) Fluorescence emission (λ_{ex} = 690 nm) of **Cy7-12** at different pH. The numbers in the legend correspond to pH values (a single step corresponds to the ~0.5 pH unit). Measured in a water/methanol (95 : 5, *v/v*) solution of **Cy7-12** (c ~2.0 × 10^{-5} mol L^{-1}). The solution was acidified by the addition of small aliquots (3 μL) of aq. HCl (c ~5.0 × 10^{-3} mol L^{-1}).

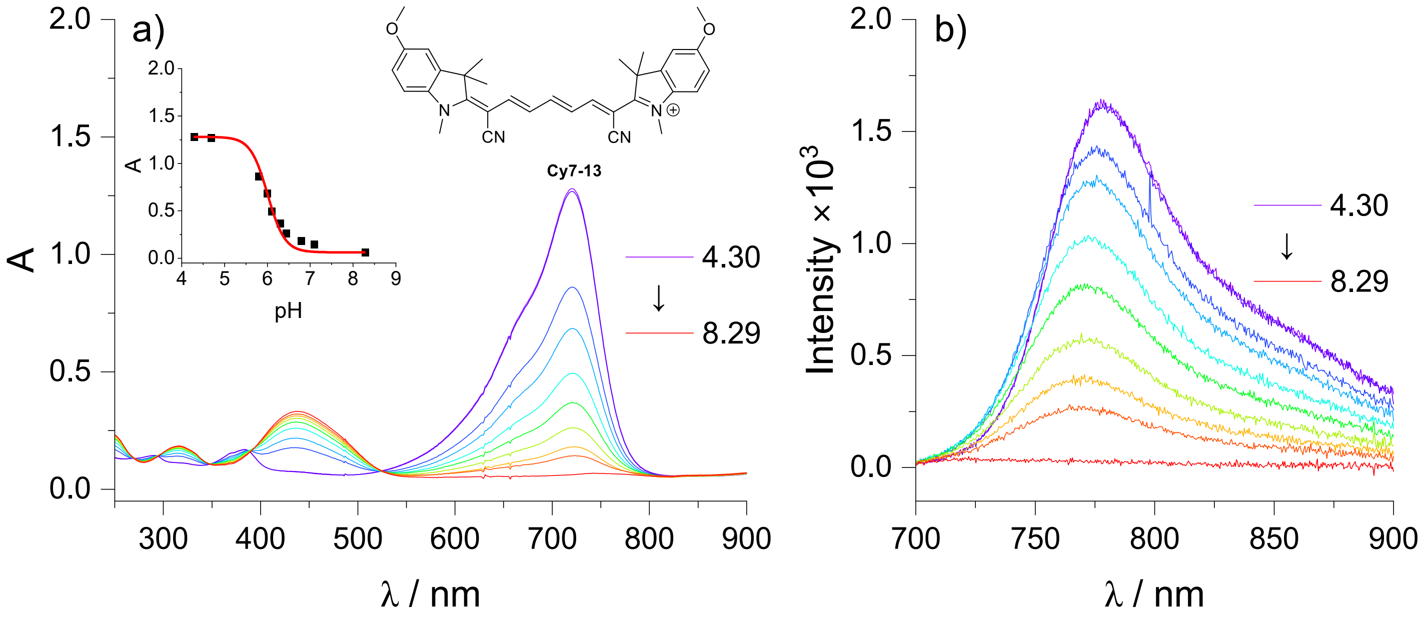

Figure S127. a) UV-vis spectra of **Cy-13** at different pH. b) Fluorescence emission (λ_{ex} = 690 nm) of **Cy7-13** at different pH. The numbers in the legend correspond to pH values (a single step corresponds to the ~0.5 pH unit). Measured in a water/methanol (95 : 5, *v/v*) solution of **Cy7-13** (c ~2.4 × 10^{-5} mol L^{-1}). The solution was acidified by the addition of small aliquots (3 μL) of aq. HCl (c ~5.0 × 10^{-3} mol L^{-1}).

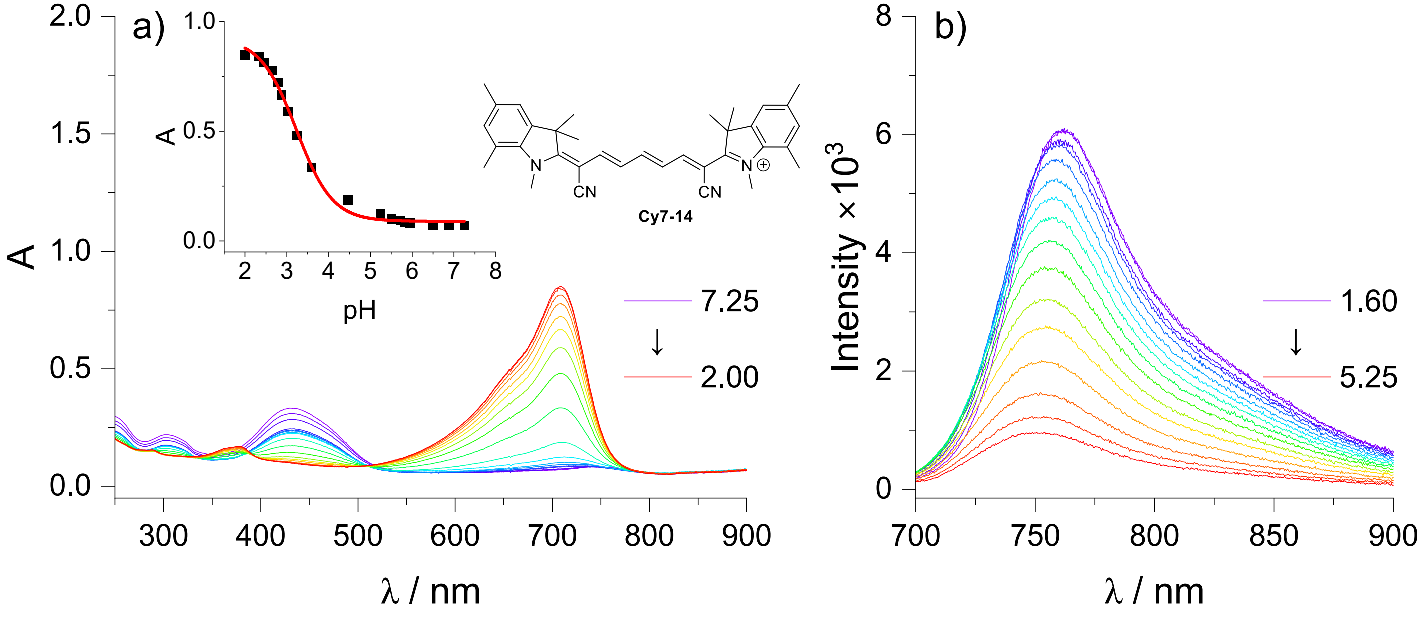

Figure S128. a) UV-vis spectra of **Cy-14** at different pH. b) Fluorescence emission (λ_{ex} = 690 nm) of **Cy7-14** at different pH. The numbers in the legend correspond to pH values (a single step corresponds to the ~0.5 pH unit). Measured in a water/methanol (95 : 5, *v/v*) solution of **Cy7-14** (c ~2.3×10^{-5} mol L^{-1}). The solution was acidified by the addition of small aliquots (3 μL) of aq. HCl (c ~5.0×10^{-3} mol L^{-1}).

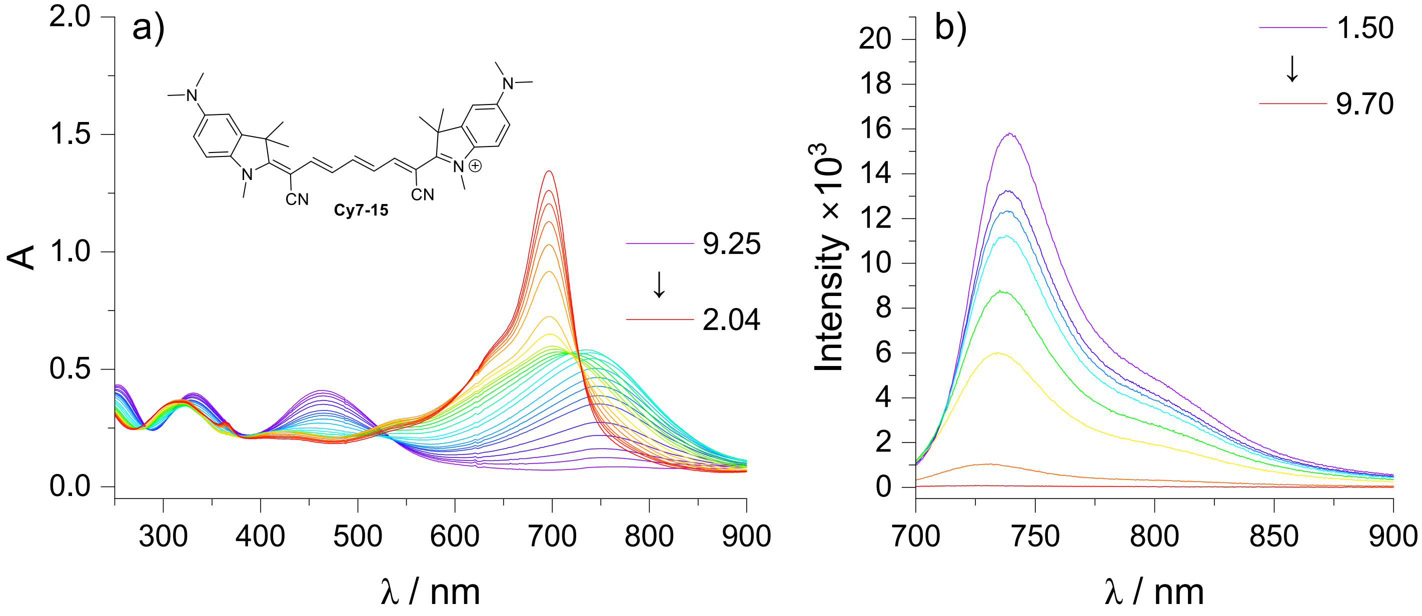

Figure S129. a) UV-vis spectra of **Cy-15** at different pH. b) Fluorescence emission (λ_{ex} = 690 nm) of **Cy7-15** at different pH. The numbers in the legend correspond to pH values (a single step corresponds to the ~0.5 pH unit). Measured in a water/methanol (95 : 5, *v/v*) solution of **Cy7-15** (c ~2.7×10^{-5} mol L^{-1}). The solution was acidified by the addition of small aliquots (3 μL) of aq. HCl (c ~5.0×10^{-3} mol L^{-1}).

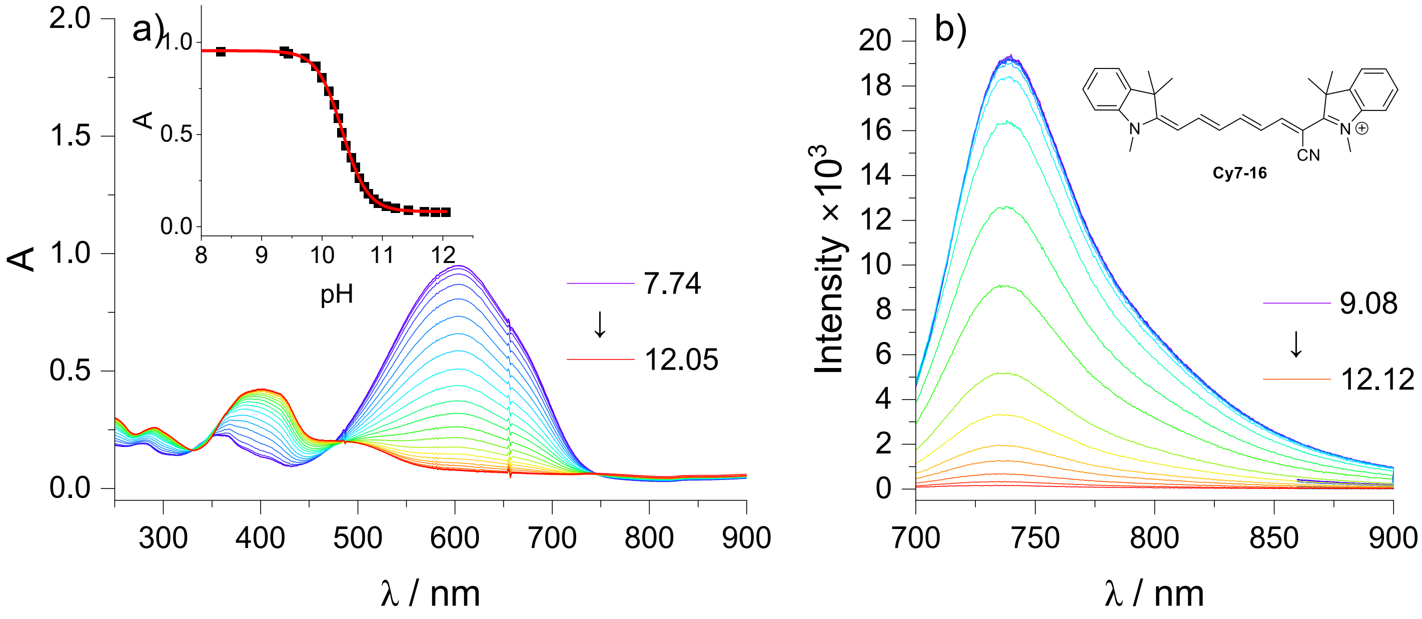

Figure S130. a) UV-vis spectra of **Cy-16** at different pH. b) Fluorescence emission (λ_{ex} = 620 nm) of **Cy-16** at different pH. The numbers in the legend correspond to pH values (a single step corresponds to the ~0.5 pH unit). Measured in a water/methanol (95 : 5, *v/v*) solution of **Cy7-16** (c ~3.1 × 10^{-5} mol L^{-1}). The solution was basified by the addition of small aliquots (3 μL) of aq. HCl (c ~8.0 × 10^{-3} mol L^{-1}).

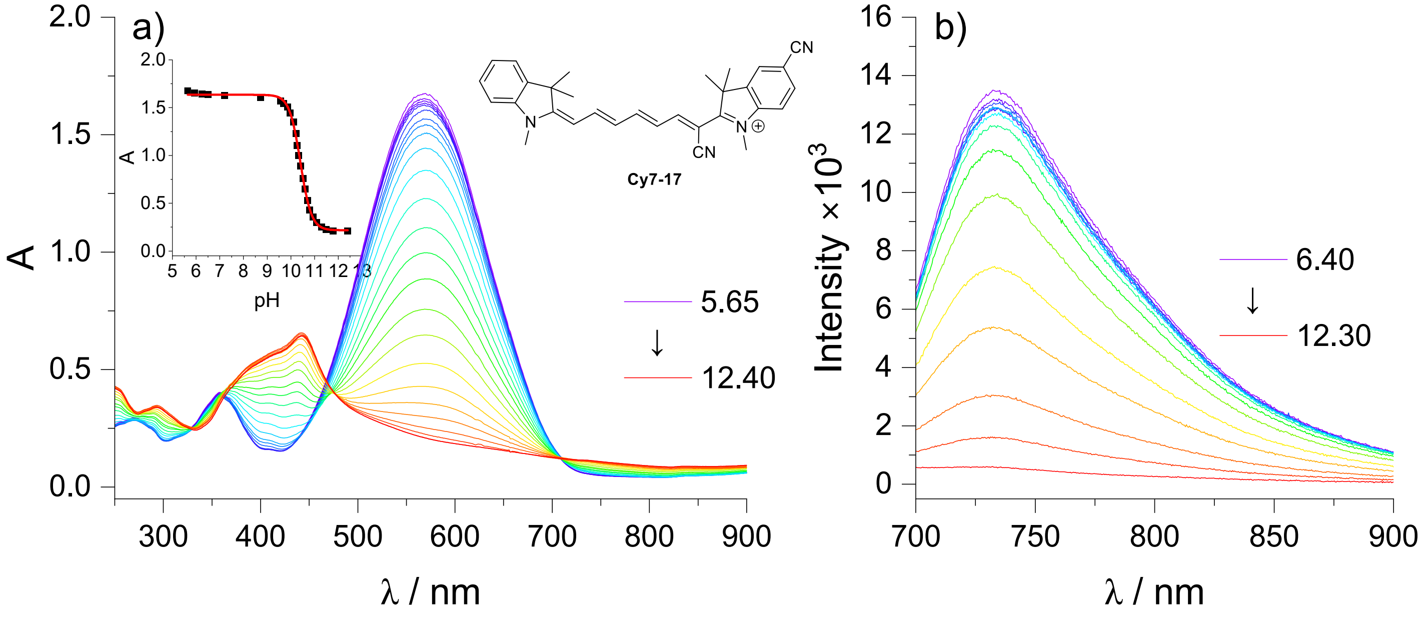

Figure S131. a) UV-vis spectra of **Cy-17** at different pH. b) Fluorescence emission (λ_{ex} = 590 nm) of **Cy-17** at different pH. Numbers in the legenda correspond to pH values (line step ~ 0.3 pH unit). Measured in a water/methanol (95 : 5, *v/v*) solution of **Cy7-17** (c~ 3.5 × 10^{-5} mol L^{-1}). The solution was basified by the addition of small aliquots (3 μL) of aq. HCl (c ~8.0 × 10^{-3} mol L^{-1}).

Nucleophile Sensing

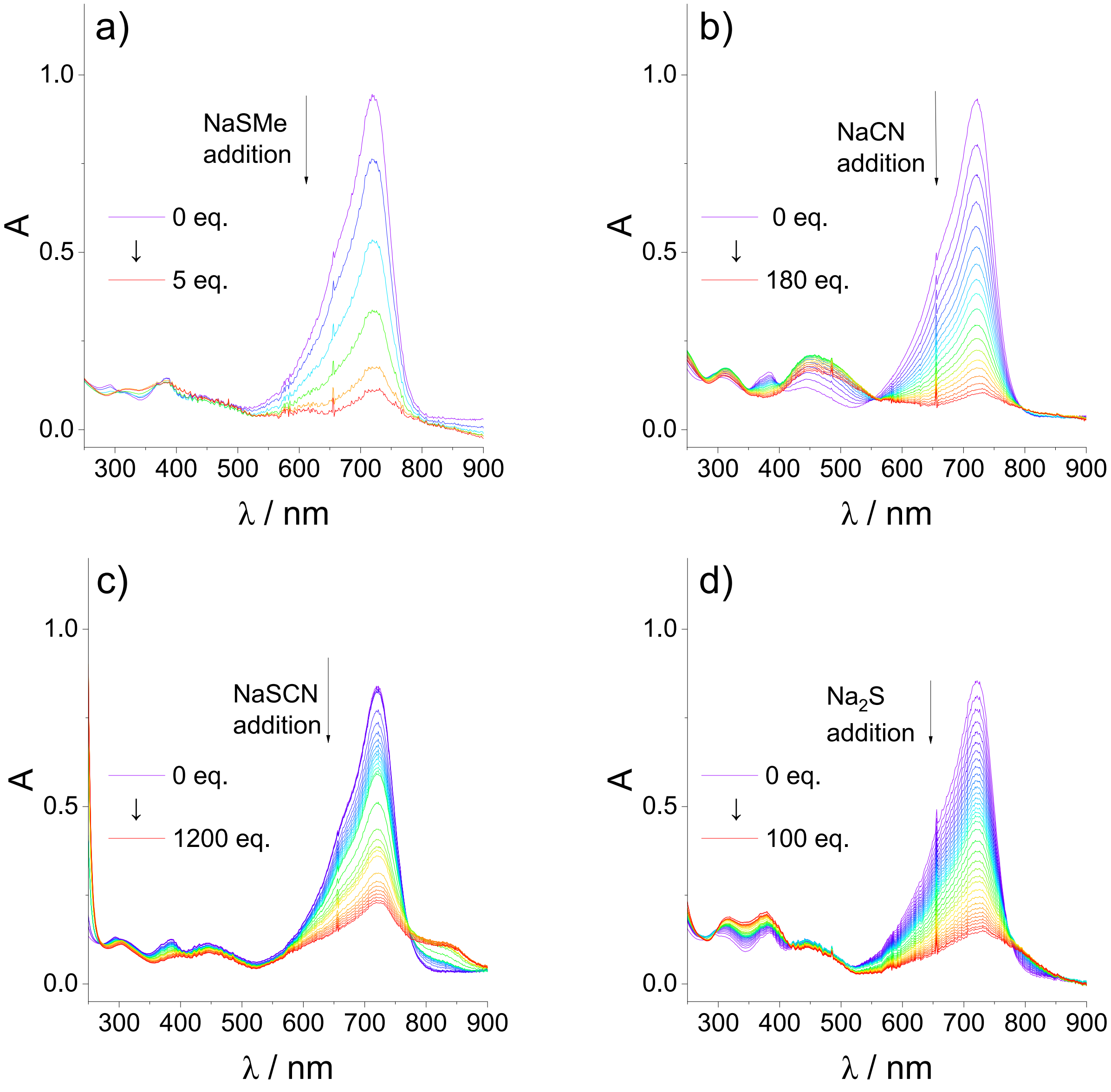

Figure S132. Absorption spectra of **Cy7-13** upon the addition of a methanolic solution (c ~5.0 × 10^{-2} mol L^{-1}) of: a) NaSMe, b) NaCN, c) NaSCN, d) Na_2S. Measured in a Britton-Robinson buffer (pH = 5.1)/methanol (95 : 5, *v/v*) solution of **Cy7-13** (c ~2.0 × 10^{-5} mol L^{-1}).

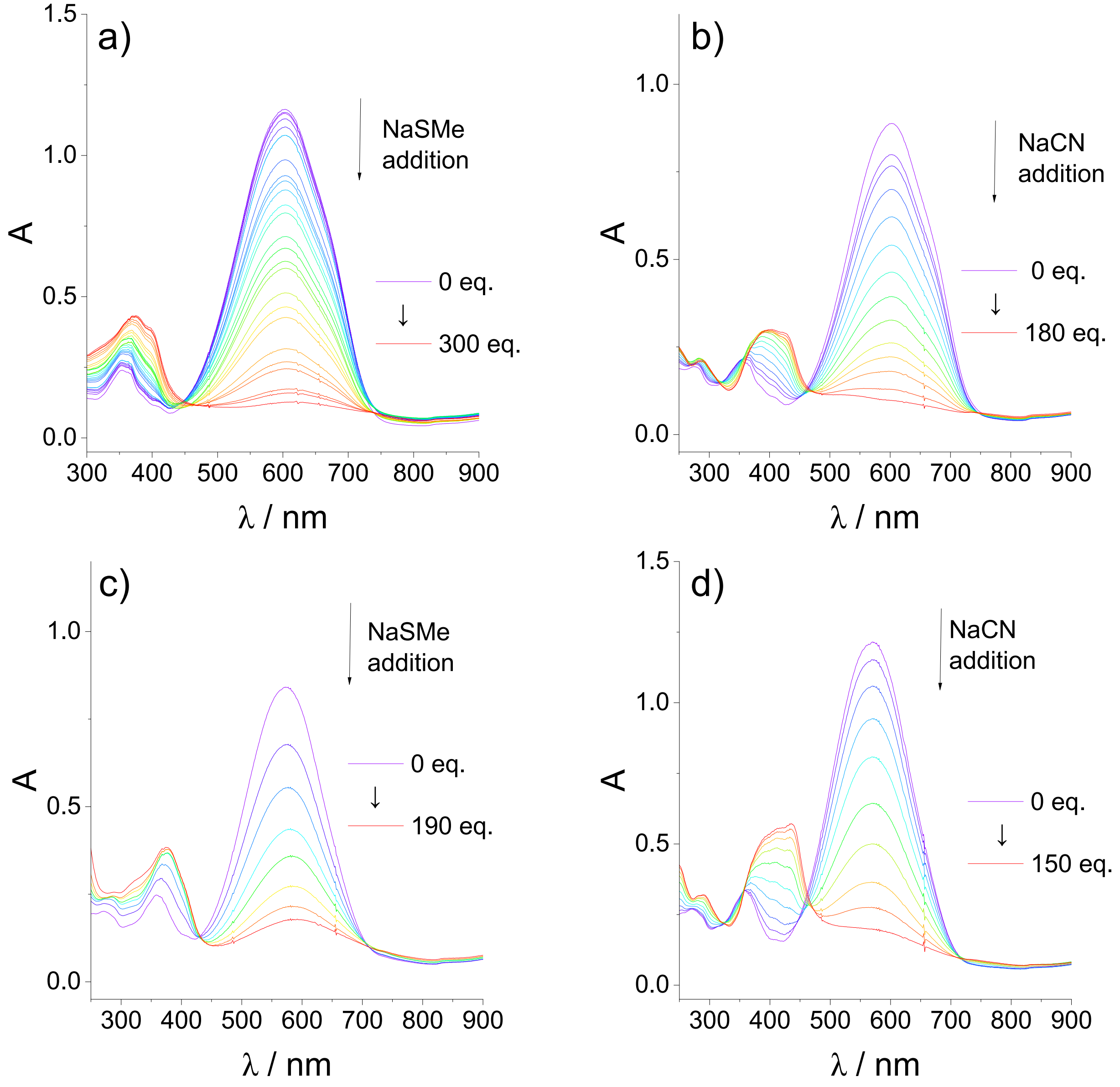

Figure S133. Absorption spectra of **Cy7-16** (a, b) and **Cy7-17** (c, d) upon the addition of addition of a methanolic solution (c ~5.0 × 10^{-2} mol L^{-1}) of: a) NaSMe, b) NaCN, c) NaSMe, d) NaCN. Measured in (a, b) a PBS buffer (pH = 7.4)/methanol (95 : 5, *v/v*) solution of **Cy7-16** (c ~3.0 × 10^{-5} mol L^{-1}) and (c, d) a Britton-Robinson buffer (pH = 7.4)/methanol (95 : 5, *v/v*) solution of **Cy7-17** (c ~3.2 × 10^{-5} mol L^{-1}).

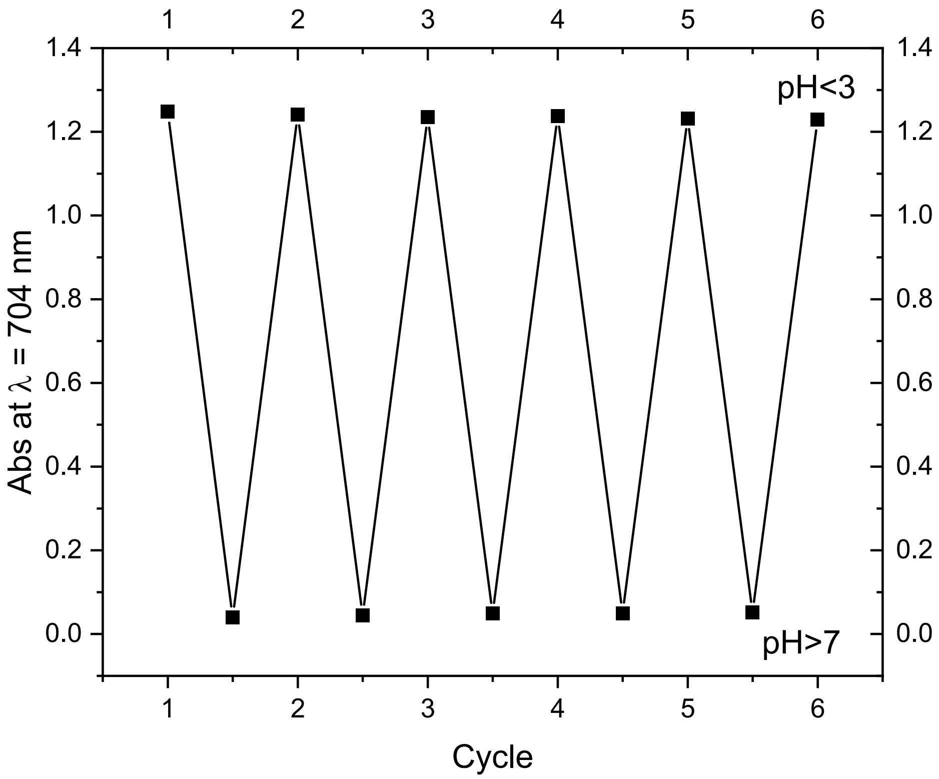

Figure S134. pH-reversibility study for **C7-12** (c ~2.7 × 10^{-5} mol L^{-1}) in a 95:5 (v/v) water/methanol mixture in the range of pH = 3–7. The absorption was measured at λ = 704 nm.

HPLC Measurements

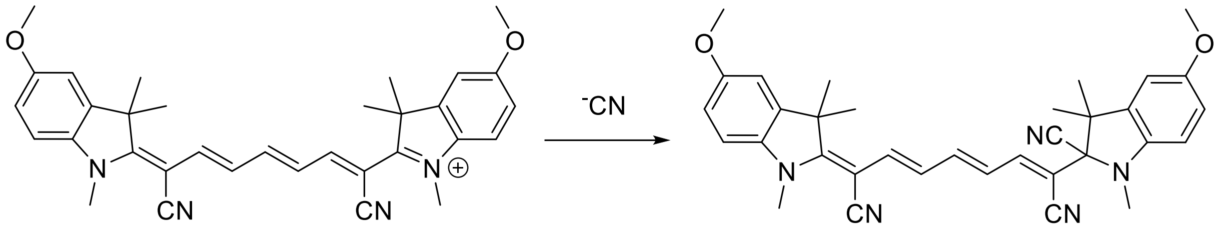

Cy7-13

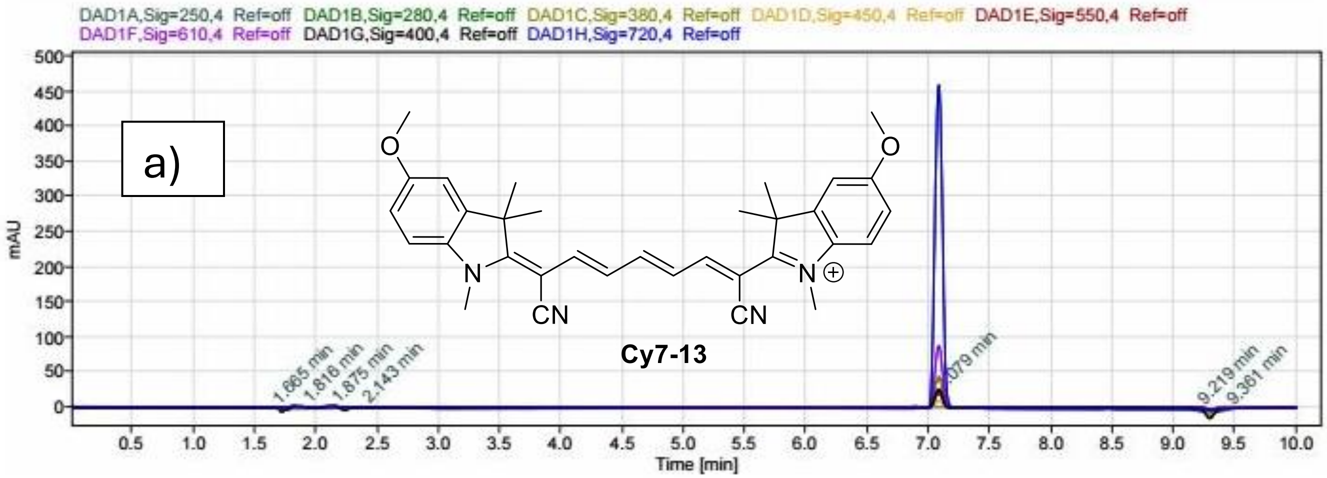

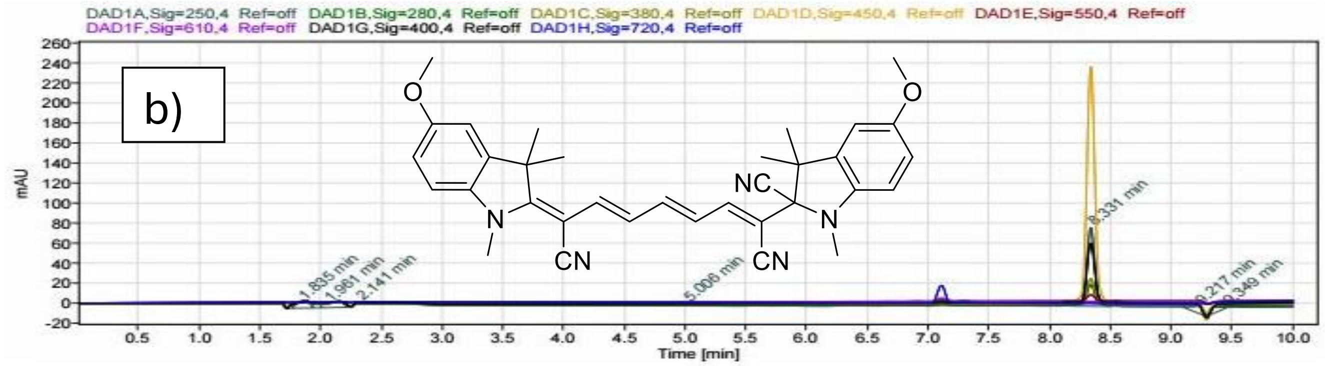

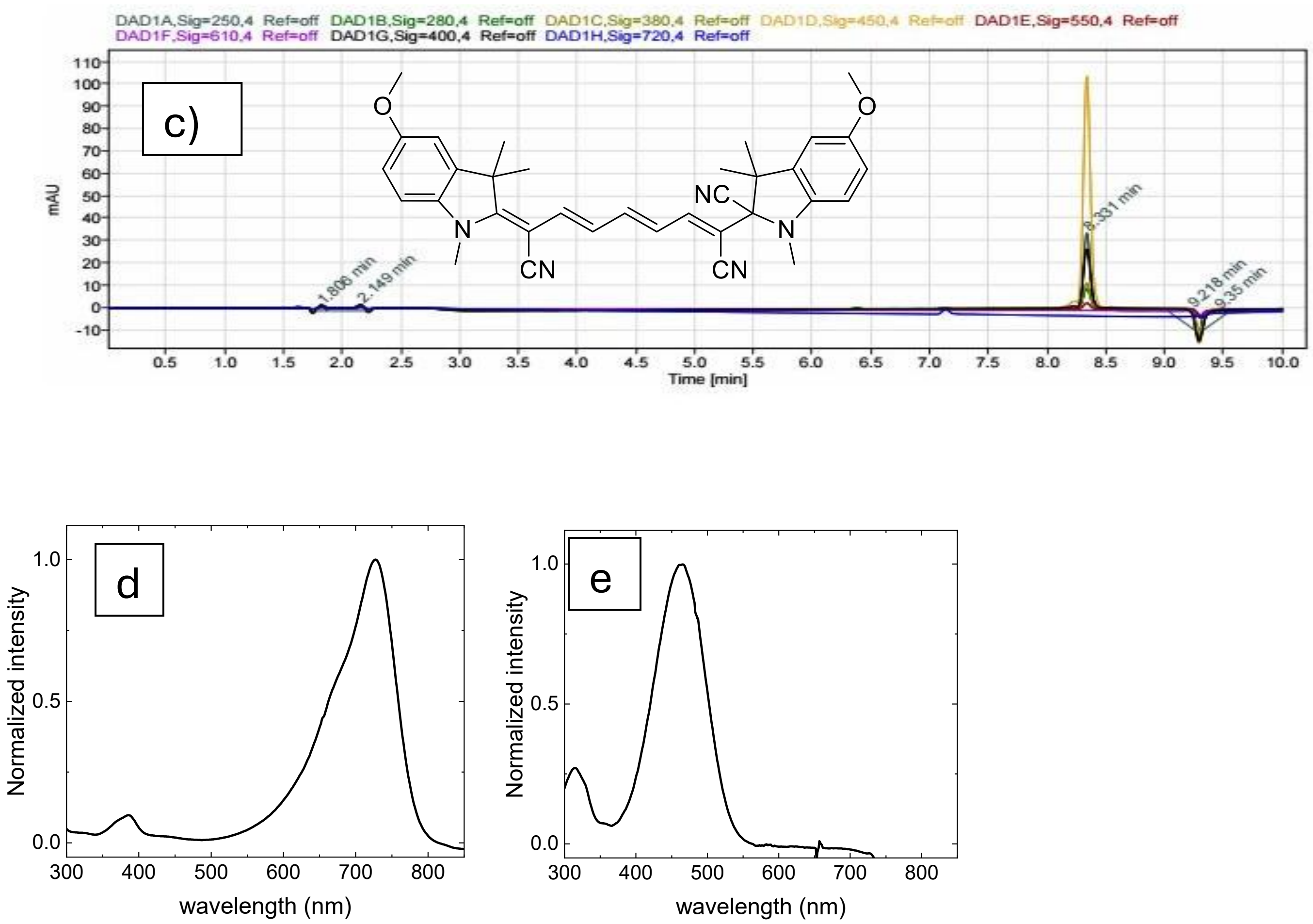

Figure S135. a) HPLC chromatogram for **Cy7-13** (c ~2.0 × 10^{-5} mol L^{-1}). b) HPLC chromatogram after the addition of aq. NaCN (180 equiv, aq. solution, c ~2.0 × 10^{-1} mol L^{-1}) to **Cy7-13** in a Britton-Robinson buffer/methanol (95 : 5, *v/v*) solution at pH = 5.1. c) HPLC chromatogram after addition of NaCN (3 equiv) to **Cy7-13** in d_4–CD_3OD in the presence of DCl (2 equiv). d) Absorption spectra extracted from an HPLC peak at 7.1 min, corresponding to **Cy7-13**. e) Absorption spectra extracted from an HPLC peak at 8.33 min, corresponding to **Cy7-13** (c ~2.0 × 10^{-5} mol L^{-1}) after the addition of NaCN (3 equiv, c ~2.0 × 10^{-1} mol L^{-1} in D_2O) to **Cy7-13** in d_4–CD_3OD in the presence of DCl (2 equiv).

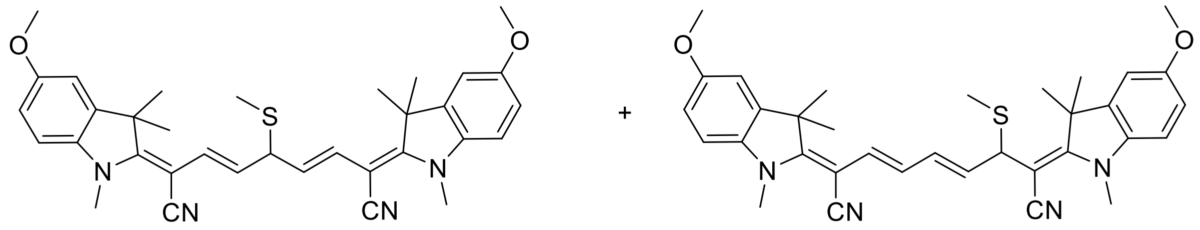

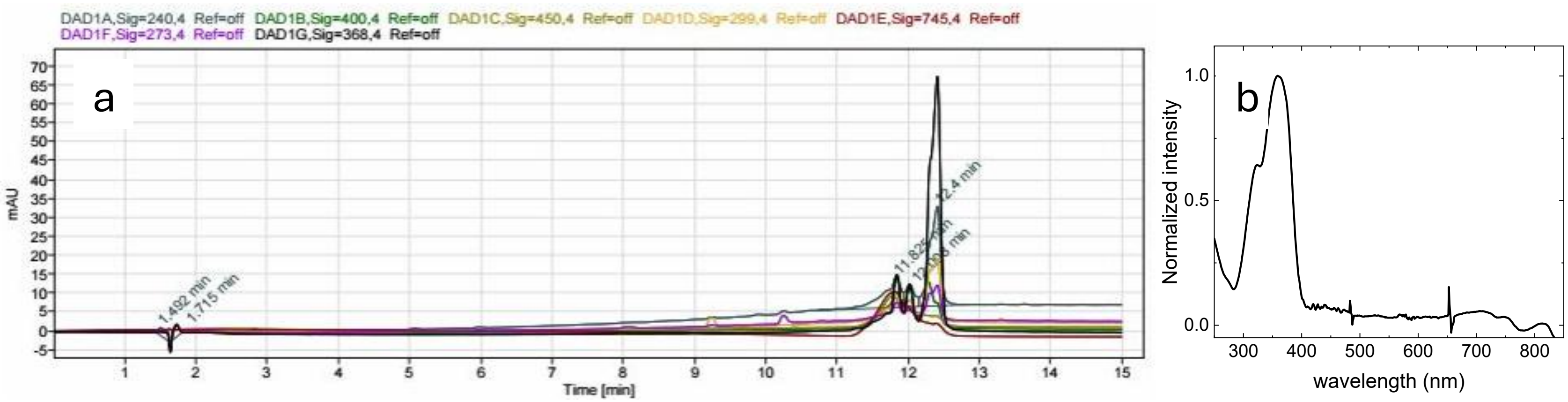

Figure S136. a) HPLC analysis for **Cy7-13** (c ~2.0 × 10^{-5} mol L^{-1}) after the addition of NaSMe (5 equiv, c ~2.0 × 10^{-1} mol L^{-1} in d_4–CD_3OD) in d_4–CD_3OD) in the presence of DCl (2 equiv, c ~4.0 × 10^{-1} mol L^{-1} in D_2O). b) Absorption spectra extracted from an HPLC peak at 12.40 min, corresponding to **Cy7-13** (c ~2.0 × 10^{-5} mol L^{-1}) after the addition of NaSMe (5 equiv, c ~2.0 × 10^{-1} mol L^{-1} in d_4–CD_3OD).

NMR experiments of nucleophiles sensing

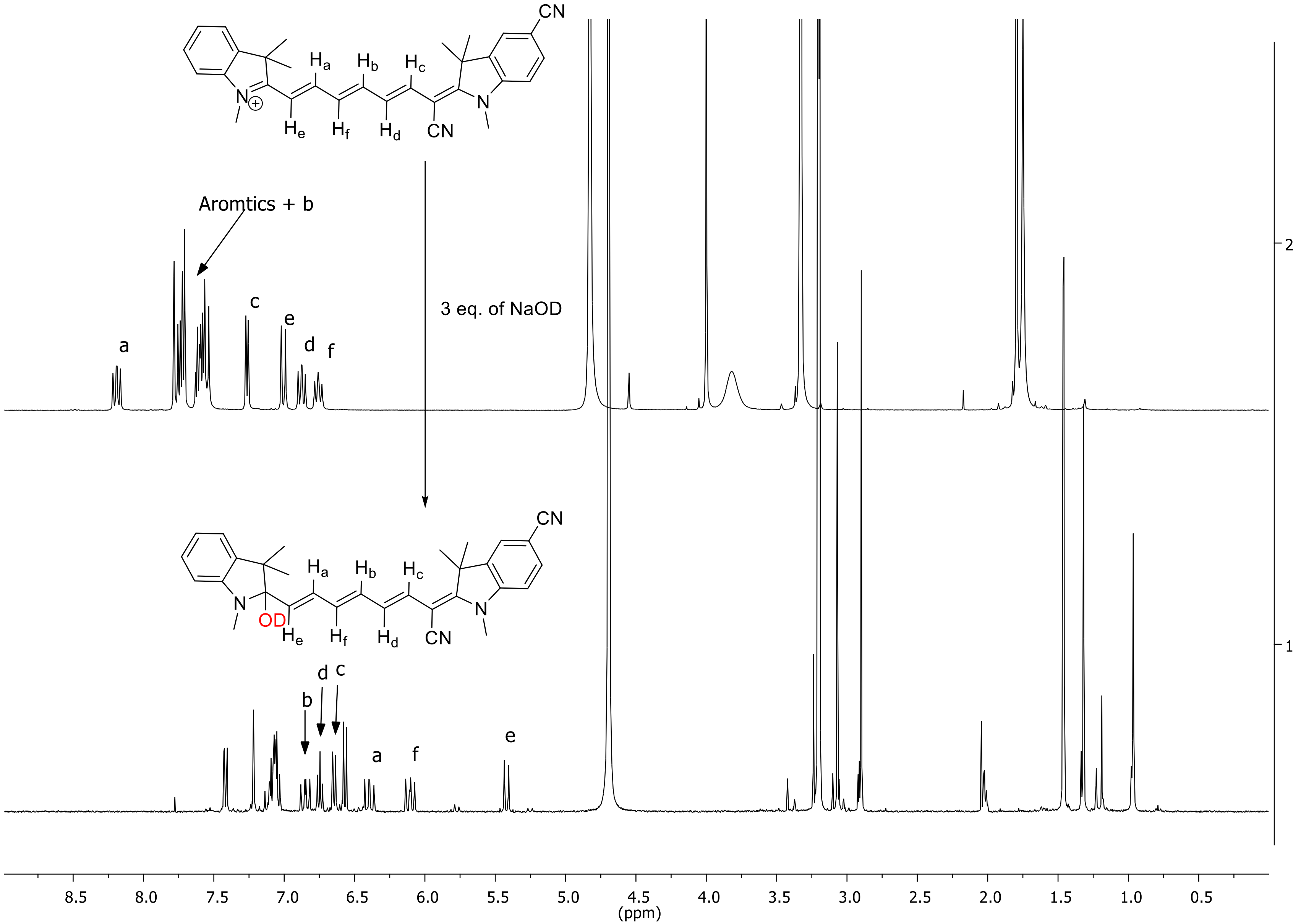

Figure S137. ^{1}H NMR spectra (400 MHz, d_4–CD_3OD) of a **Cy7-17** solution (c ~2.0×10^{-2} mol L^{-1} in d_4–CD_3OD) before and after the addition of NaOD (3 eq, c ~2.0×10^{-1} mol L^{-1} in D_2O).

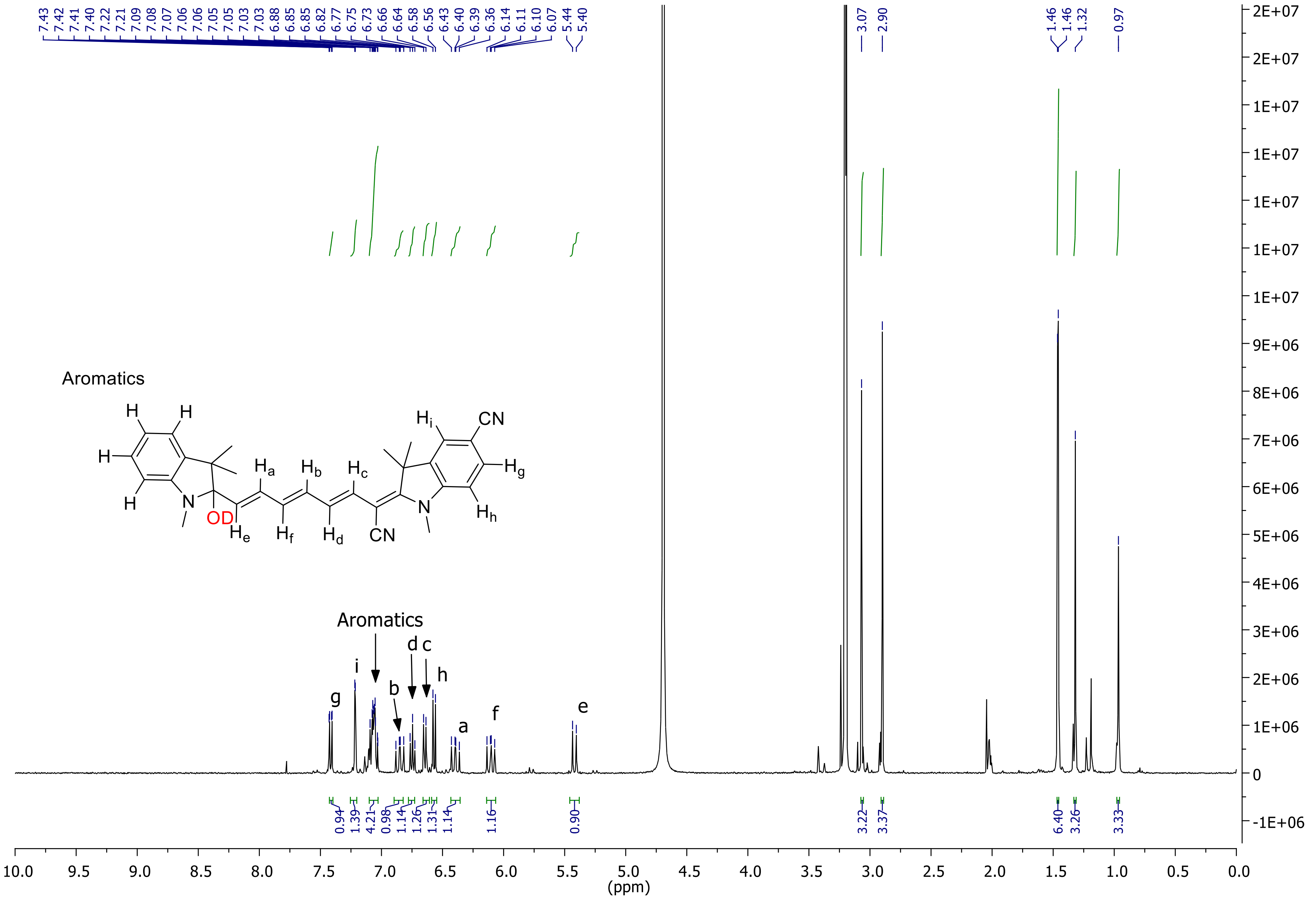

Figure S138. ^{1}H NMR (400 MHz, d_4–CD_3OD): a product of the NaOD addition (3 eq, c ~2.0×10^{-1} mol L^{-1} in D_2O) to **Cy7-17**.

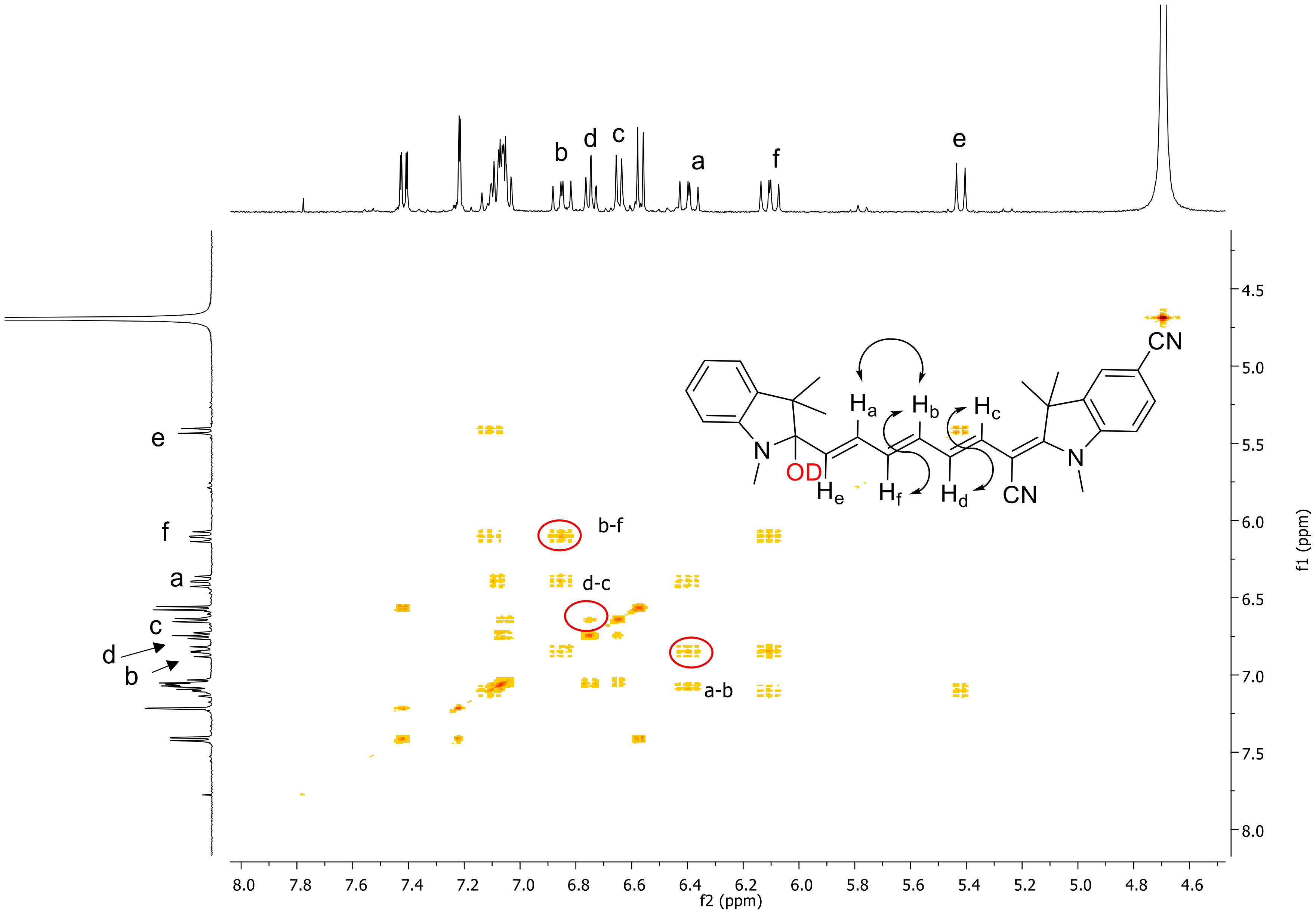

Figure S139. ^{1}H-{^{1}H} COSY (400 MHz, d_4–CD_3OD): a product of the NaOD addition (3 eq, c ~2.0 × 10^{-1} mol L^{-1} in D_2O) to **Cy7-17**.

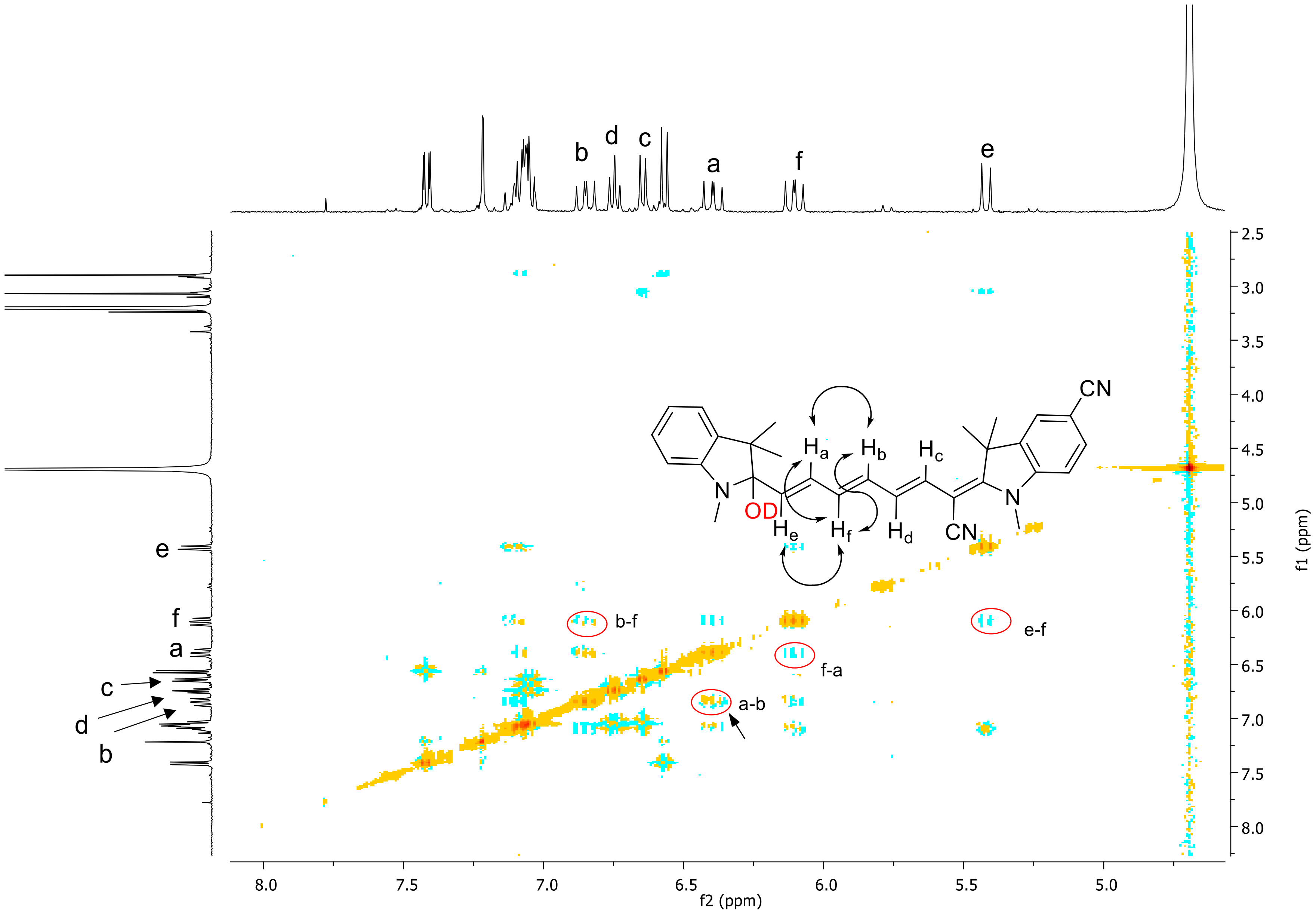

Figure S140. ^1H-{^1H} NOESY (400 MHz, d_4–CD_3OD): a product of the addition of NaOD (3 eq, c ~2.0×10^{-1} mol L^{-1} in D_2O) to **Cy7-17**.

Sensing of Cy7-13 by NaCN

Figure S141 shows the ^{1}H NMR spectra of **Cy7-13** in *d*$_4$–CD_3OD before (upper spectra) and after (bottom spectra) the additon of NaCN (3 equiv, c ~2.0×10^{-1} mol L^{-1} in D_2O) in the presence of DCl (2 equiv, c ~4.0×10^{-1} mol L^{-1} in D_2O). The original symmetrical spectrum of **Cy7-13** is converted into a new set of signals. An upfield shift of the vinyl protons is observed, which is consistent with the loss of the positive charge from the heterocyclic nitrogen. This suggests a nucleophilic attack of the cyanide to the iminium carbon of **Cy7-13.**

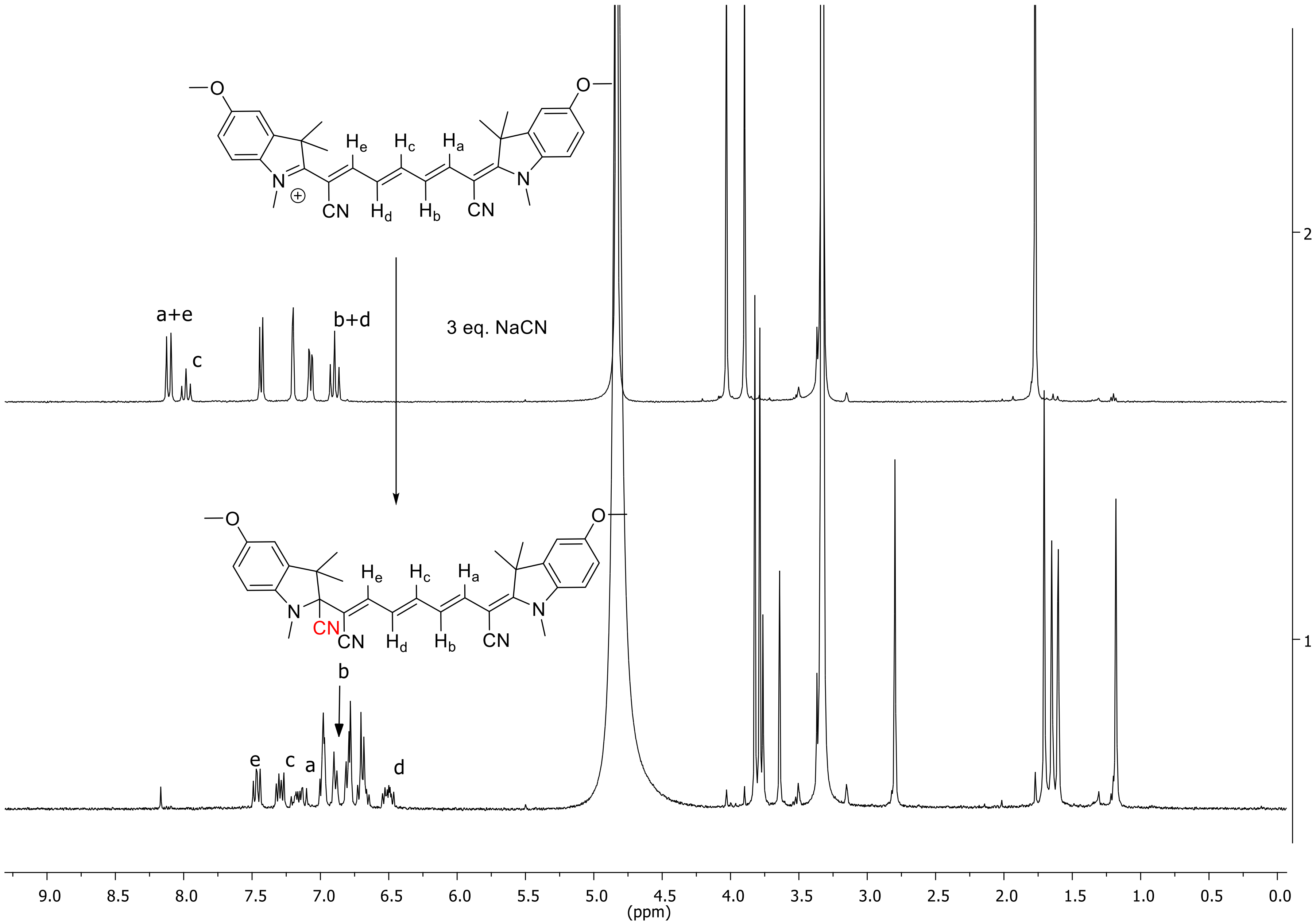

Figure S141. ^{1}H NMR spectra (400 MHz, d_4–CD_3OD) of **Cy7-13** (with the addition of DCl; 2 equiv, c ~4.0×10^{-1} mol L^{-1} in D_2O) before and after the addition of NaCN (3 equiv, c ~2.0×10^{-1} mol L^{-1} in D_2O).

Sensing of Cy7-13 by NaSMe

Figure 142 shows the ^{1}H NMR spectra before (upper spectra) and after (middle spectra) the additon of NaSMe (5 equiv, c ~2.0×10^{-1} mol L^{-1} in d_4–CD_3OD) to a methanolic solution of **Cy7-13** in the presence of DCl (2 equiv, c ~4.0×10^{-1} mol L^{-1} in D_2O). The original symmetrical spectrum of **Cy7-13** is converted into a new set of signals. Acidification of the solution with DCl (10 eq, bottom) restores the original spectrum of **Cy7-13**. Two signals significantly shifted upfield compared to the original spectrum of **Cy7-13** (red and green circle in Figure S143), which was attributed to the concomitant addition of MeS^{-} to the C2´ and C4´ of **Cy7-13** (Figure S143). Using ^{1}H-^{1}H NOESY and ^{1}H-^{1}H COSY measurements (Figure S144, Figure S145), we observed numerous interactions of the aforementioned signals with vinylic protons – specifically, with C2´-H and C6´-H in the case of the attack of MeS^{-} at the C4´-position (blue circle) and a cross-peak between C4´-H (orange circle) and C5´-H (magenta circle) in the case of the attack at the C2´-position (Figure S146A). In the latter adduct, the ^{1}H-^{1}H NOESY interaction between C2´-H and C3´-H was observed (brown circle, Figure S146B). In the ^{1}H-^{1}H NOESY measurements, the cross-peak derived from the interaction with the methyl moiety of the thiomethyl group and C3´-H was observed for both adducts (black circle, Figure S147).

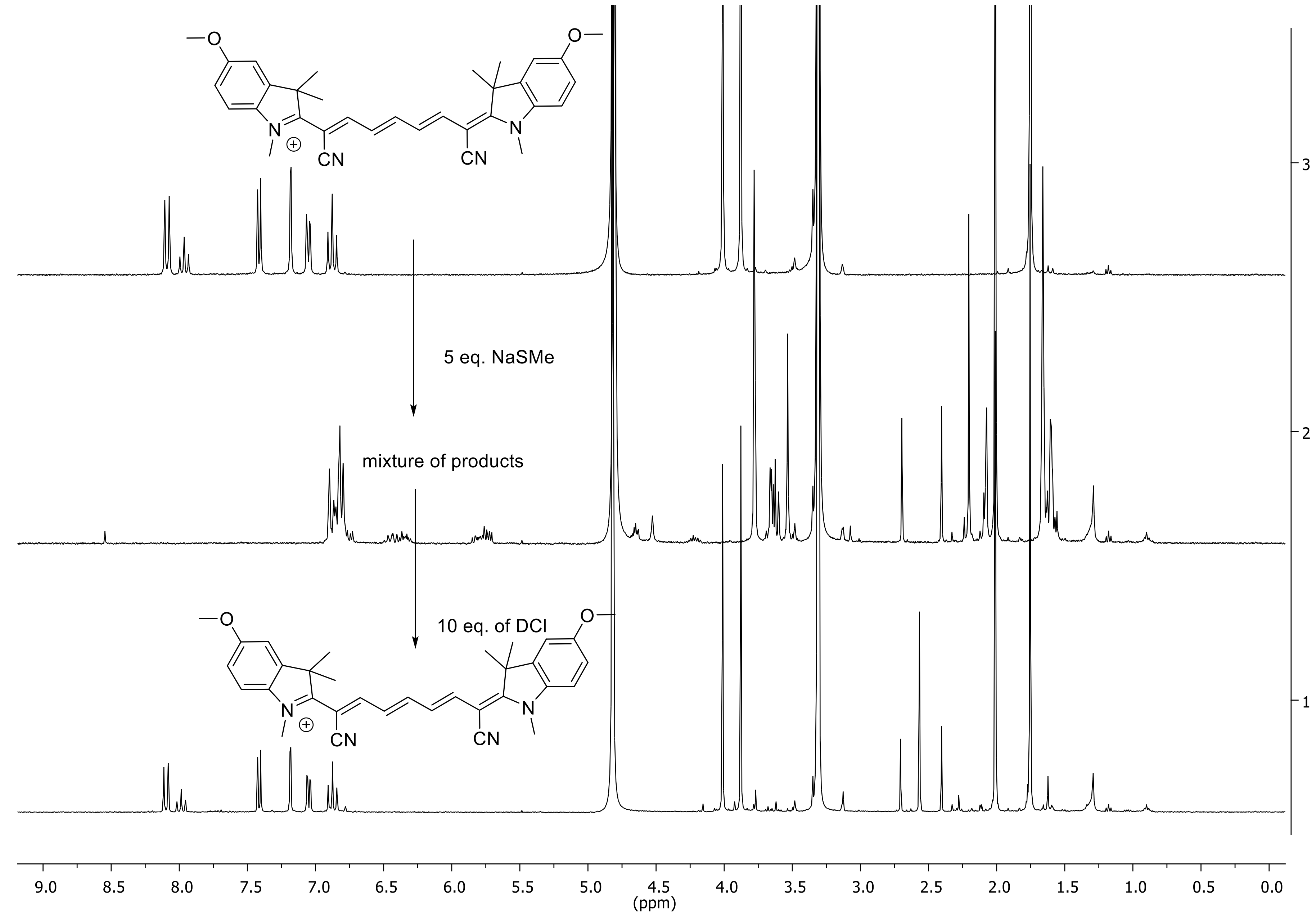

Figure S142.^{1}H NMR spectra (400 MHz, d_4–CD_3OD) of **Cy7-2** (top), after the addition of NaSMe (5 equiv, c ~2.0 × 10^{-1} mol L^{-1} in d_4–CD_3OD, middle), and after subsequent DCl addition (10 equiv, c ~4.0 × 10^{-1} mol L^{-1} in D_2O, bottom).

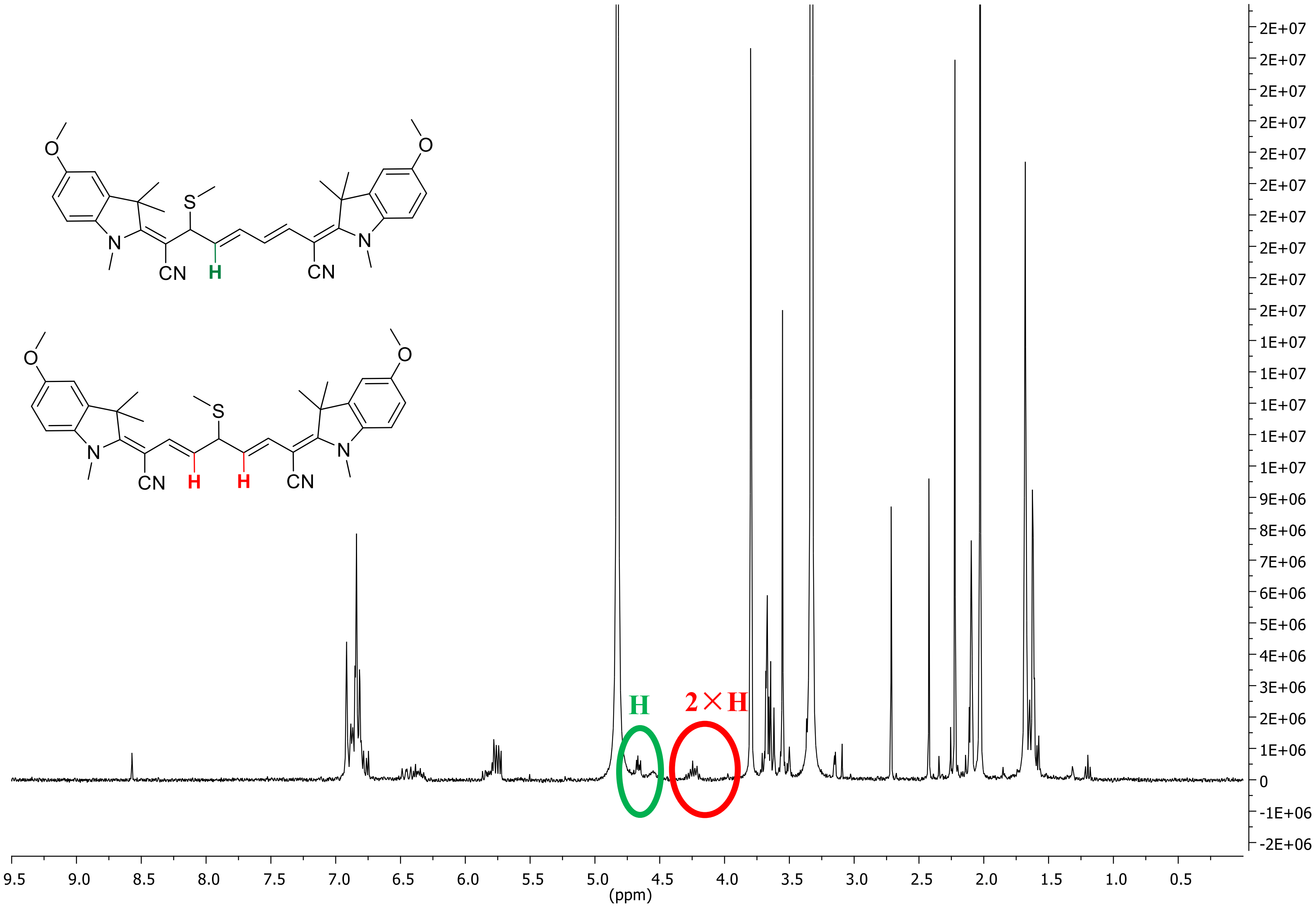

Figure S143. ^{1}H NMR (400 MHz, d_4–CD_3OD): products of the NaSMe addition to **Cy7-2**.

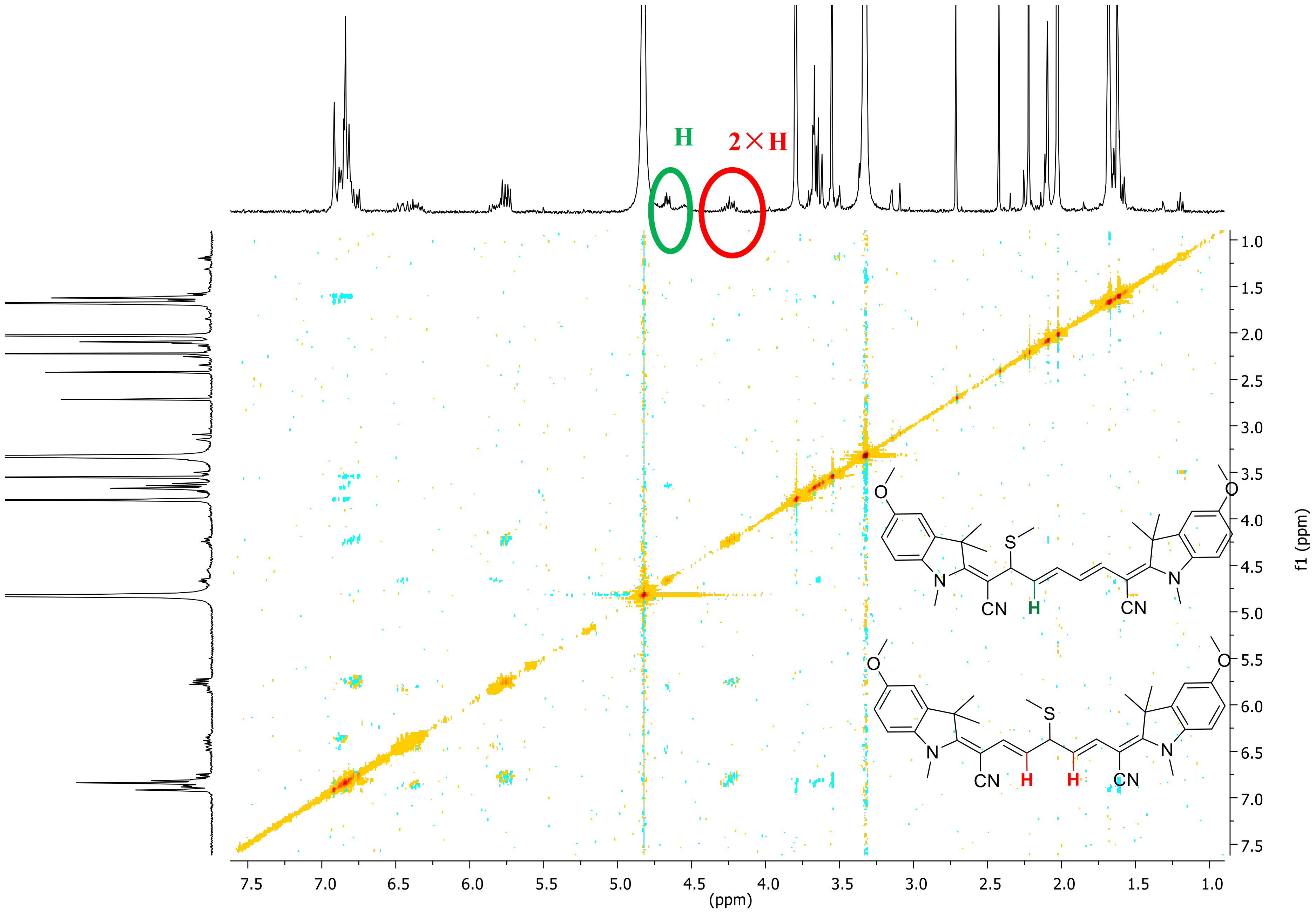

Figure S144. ^{1}H-{^{1}H} NOESY (400 MHz, d_4–CD_3OD): products of the NaSMe addition to **Cy7-2**.

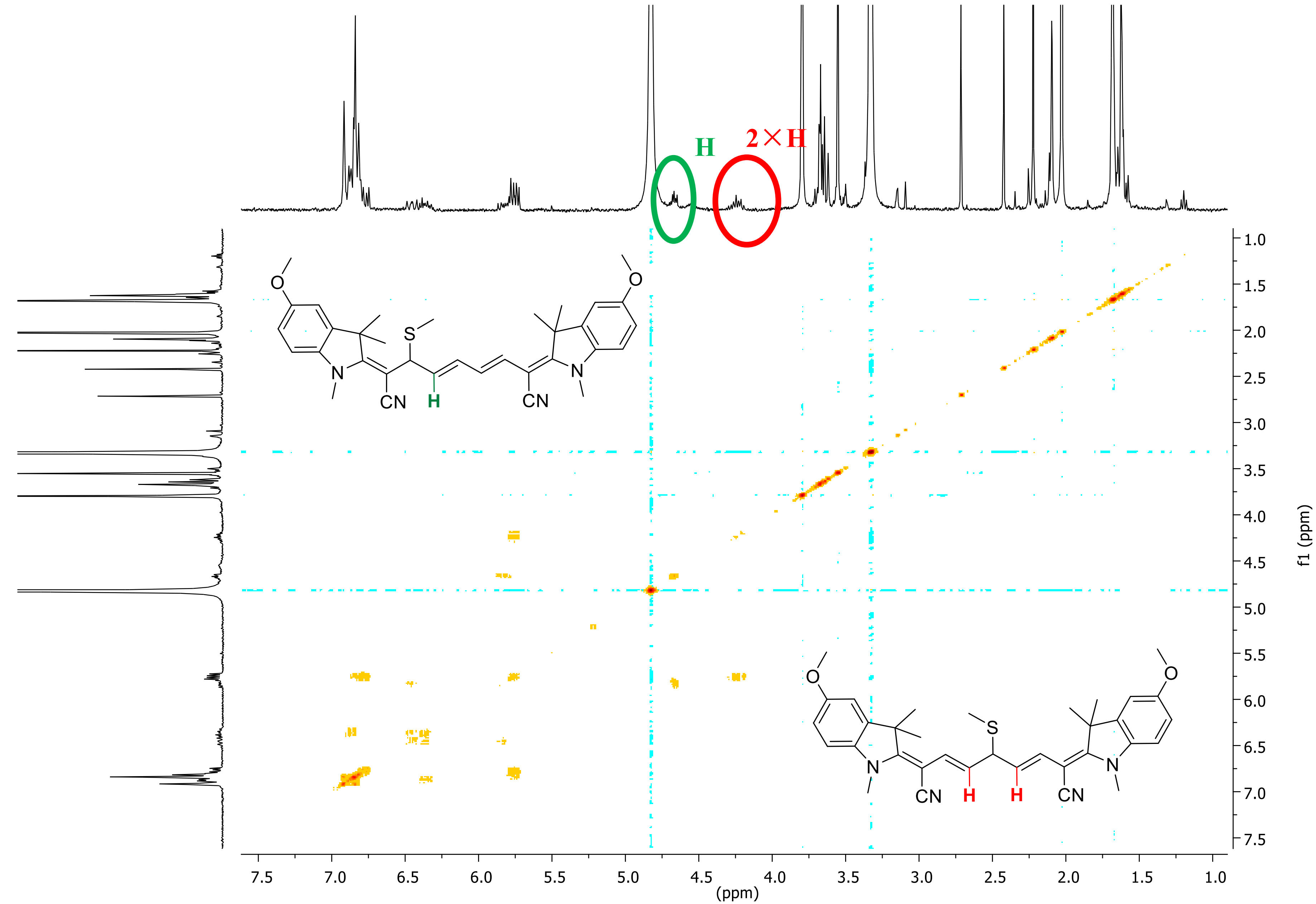

.

Figure S145. ^{1}H-{^{1}H} COSY (400 MHz, *d*4–CD3OD): products of the NaSMe addition to **Cy7-2**.

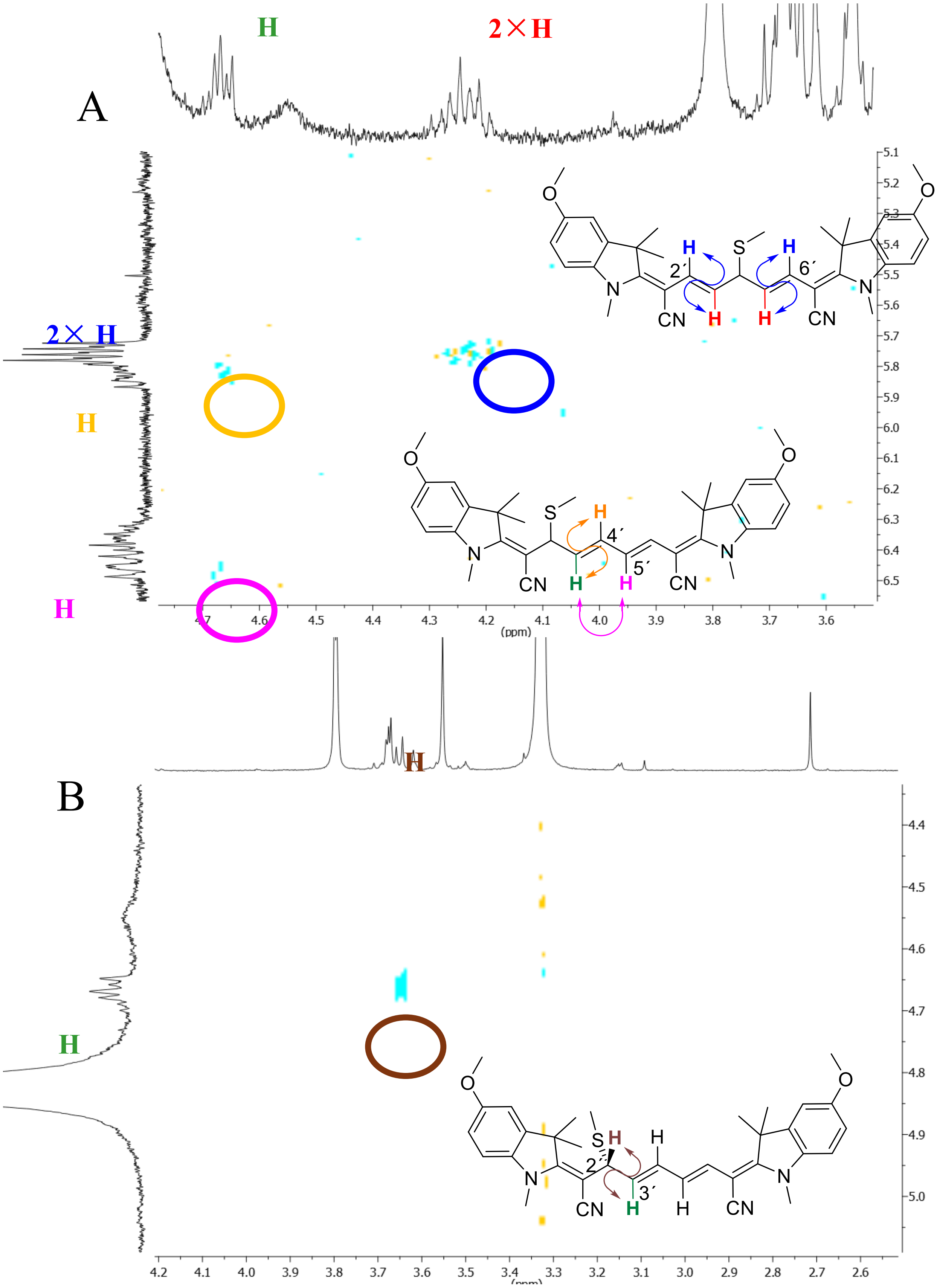

Figure S146. ^{1}H-{^{1}H} NOESY (400 MHz, d_4–CD_3OD): products of the NaSMe addition to **Cy7-2**.

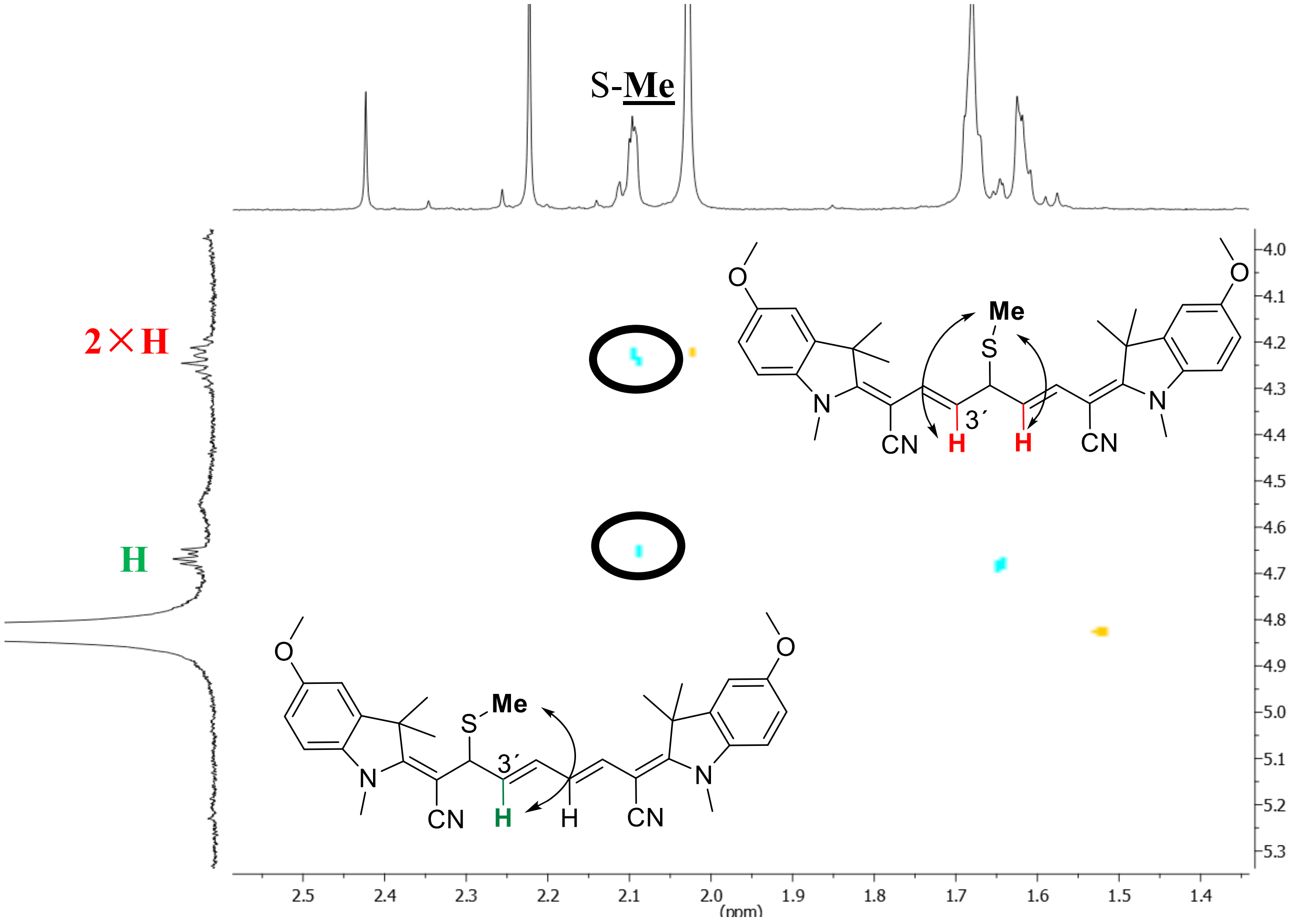

Figure S147. ^{1}H-{^{1}H} NOESY (400 MHz, d_4–CD_3OD): products of NaSMe addition to **Cy7-2**.

Fluorescence Lifetimes

The fluorescence lifetimes of several 1′-substituted Cy5 and Cy7 derivatives were obtained at λ_{ex} = 670 nm (Table S1). As was the case in our previous paper,[1] the biexponential lifetimes observed in some of the studied derivatives indicated the presence of two distinct emissive species contributing to the fluorescence emission (*E*- and *Z*-isomers). However, a direct correlation between the lifetimes and the variations in fluorescence quantum yields was not observed. Consequently, the efficiency ratios of competing deexcitation pathways remain undetermined.

Table S1. Fluorescence lifetimes of selected cyanines

Cy	Excitation/nm	τ_1 / ns	τ_2 / ns	Φ_F (λ_{ex} = 670 nm)
**Cy5-6**	635	0.09 ± 0.01	/	0.001
**Cy7-3**	670	0.24 ± 0.01	3.5 ± 0.2	0.022
**Cy7-5**	670	0.30 ± 0.01	4.7 ± 0.3	0.025
**Cy7-7**	670	0.23	3.60	0.003
**Cy7-8**	670	0.53 ± 0.01	6.9 ± 0.4	0.037
**Cy7-16**	635	0.51 ± 0.01	/	0.098

Fluorescence lifetime was measured in a methanolic solution and determined in a fluorimeter in 1.0 cm quartz fluorescence cuvettes at 23 ± 1 °C; sample concentrations with an absorbance below 0.1 at the excitation wavelength were used. Fluorescence lifetimes were measured three times and were averaged for each sample, except for **Cy7-7**. Fluorescence quantum yields were determined in methanol on a fluorimeter as absolute values using an integrating sphere. The solution concentrations were adjusted to have absorbance below 0.15

References

1. Bedocchi, O.; Polena, J.; Okorocenkova, J.; Slavicek, P.; Klan, P., Engineering the Photophysics of Cyanines by Chain C1′ Substituents. *J. Org. Chem.* **2025,** *90*, 17797-17813.
2. Stackova, L.; Muchova, E.; Russo, M.; Slavicek, P.; Stacko, P.; Klan, P., Deciphering the Structure–Property Relations in Substituted Heptamethine Cyanines. *J. Org. Chem.* **2020,** *85*, 9776-9790.
3. Wilkinson, F.; Helman, W. P.; Ross, A. B., Quantum Yields for the Photosensitized Formation of the Lowest Electronically Excited Singlet State of Molecular Oxygen in Solution. *J. Phys. Chem. Ref. Data* **1993,** *22*, 113-262.
4. Mongay, C.; Cerda, V., A Britton-Robinson Buffer of Known Ionic Strength. *Ann. Chimica* **1974,** *64*, 409-412.
